# Copper(I)-catalyzed asymmetric 1,3-dipolar cycloaddition of 1,3-enynes and azomethine ylides

**DOI:** 10.1038/s41467-023-40409-4

**Published:** 2023-08-04

**Authors:** Bo-Ran Wang, Yan-Bo Li, Qi Zhang, Dingding Gao, Ping Tian, Qinghua Li, Liang Yin

**Affiliations:** 1 The Research Center of Chiral Drugs, Shanghai Frontiers Science Center of TCM Chemical Biology, Innovation Research Institute of Traditional Chinese Medicine, Shanghai University of Traditional Chinese Medicine, 1200 Cailun Road, Shanghai, 201203 China; 2 CAS Key Laboratory of Synthetic Chemistry of Natural Substances, Center for Excellence in Molecular Synthesis, Shanghai Institute of Organic Chemistry, University of Chinese Academy of Sciences, Chinese Academy of Sciences, 345 Lingling Road, Shanghai, 200032 China

**Keywords:** Asymmetric catalysis, Synthetic chemistry methodology, Homogeneous catalysis

## Abstract

Herein, we report a copper(I)-catalyzed asymmetric 1,3-dipolar cycloaddition of azomethine ylides and 1,3-enynes, which provides a series of chiral poly-substituted pyrrolidines in high regio-, diastereo-, and enantioselectivities. Both 4-aryl-1,3-enynes and 4-silyl-1,3-enynes serve as suitable dipolarophiles while 4-alkyl-1,3-enynes are inert. Moreover, the method is successfully applied in the construction of both tetrasubstituted stereogenic carbon centers and chiral spiro pyrrolidines. The DFT calculations are also conducted, which imply a concerted mechanism rather than a stepwise mechanism. Finally, various transformations started from the pyrrolidine bearing a triethylsilylethynyl group and centered on the alkyne group are achieved, which compensates for the inertness of 4-alkyl-1,3-enynes in the present reaction.

## Abstract (web summary)

One of the most efficient method to access chiral poly-substituted pyrrolidines is the catalytic asymmetric 1,3-dipolar cycloaddition, but using this reaction with weakly activated alkenes remains elusive. Here, the authors disclose a copper(I)-catalyzed asymmetric 1,3-dipolar cycloaddition of azomethine ylides and 1,3-enynes.

## Introduction

**Fig. 1:**
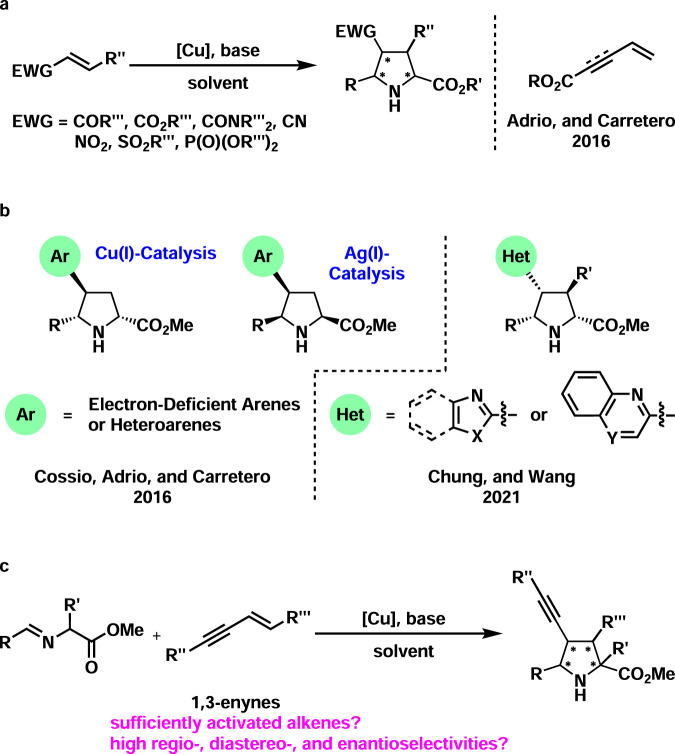
Introduction to catalytic asymmetric 1,3-dipolar cycloadditions and our working hypothesis. **a** Classical catalytic asymmetric 1,3-dipolar cycloaddition of α,β-unsaturated and α,β,γ,δ-unsaturated compounds. **b** Catalytic asymmetric 1,3-dipolar cycloaddition of electron-deficient vinylarenes and alkenyl heteroarenes. **c** Catalytic asymmetric 1,3-dipolar cycloaddition of 1,3-enynes: this work.

Chiral poly-substituted pyrrolidines are not only key structure units in both natural and man-made bioactive molecules but also versatile intermediates in organic synthesis^1–10^. One of the most efficient methods to access these compounds is the transition metal-catalyzed asymmetric 1,3-dipolar cycloaddition^11–18^. Among various metal catalysts, copper(I) catalysts have gained a prominent position, which exhibited admiring abilities to enable high reaction efficiency and wonderful asymmetric induction^19–34^. Various 1,3-dipoles, such as nitrones, azomethine ylides, and azomethine imines, serve as wonderful substrates. The dipolarophiles are generally limited to strongly activated alkenes, including α,β-unsaturated compounds and α,β,γ,δ-unsaturated compounds^35^ (Fig. 1a). The strong electron-withdrawing groups include aldehyde, ketone, ester, amide, cyano, nitro, sulfone, and phosphonate. However, catalytic asymmetric 1,3-dipolar cycloadditions with weakly activated alkenes are very uncommon and thus remain elusive^36–41^.

In 2016, Cossío, Adrio, and Carretero reported a copper(I)- or silver(I)-catalyzed asymmetric 1,3-dipolar cycloaddition of azomethine ylides and weakly activated alkenes (Fig. 1b, left). In this reaction, vinylarenes bearing electron-deficient aryl groups, as well as vinylheteroarenes, were used as satisfactory 1,3-dipoles^36^. In 2021, Chung, Wang, and co-workers successfully developed a copper(I)-catalyzed asymmetric 1,3-dipolar cycloaddition of β-substituted alkenyl heteroarenes by using a powerful chiral monodentate phosphoramidite ligand (Fig. 1b, right)^38^. Other notable examples are copper-catalyzed asymmetric 1,3-dipolar cycloadditions of fullerenes and cyclopropenes with azomethine ylides^39–43^. The highly strained nature of the olefin unit in fullerenes enabled the reaction. Despite these leading achievements, the scope of weakly activated alkenes is still significantly limited. Moreover, it is noted that the products in these reactions are structurally specific, which does not allow facile further transformations based on the electron-deficient aryls or heteroaryls. Thus research efforts on the reaction with weakly activated alkenes that are easily functionalized at a later stage are highly desirable^11–18^.

1,3-Enynes were initially used as important intermediates in the synthesis of highly substituted aromatic rings^44,45^. Then Krische pioneered the transition metal-catalyzed three-component coupling reactions with 1,3-enynes^46–55^. In 2004, Krische and co-workers uncovered a rhodium-catalyzed reductive coupling of 1,3-enynes and α-keto aldehydes under a hydrogenation atmosphere, which afforded (*E*)-2-hydroxy-3,5-dien-1-one products^46^. In 2008, the same group disclosed a ruthenium-catalyzed reductive coupling of 1,3-enynes and alcohols, providing synthetically versatile homopropargyl alcohols^52^. These instructive works stimulated further extensive investigations on the utilization of 1,3-enynes in three-component coupling reactions and other interesting transformations^56–58^, especially copper-catalyzed asymmetric syntheses of propargyl alcohols/amines^59–63^ and allenes^64–73^. Based on these seminar reports, it is envisioned that the presence of a conjugated carbon-carbon triple bond may lead to weak activation of the olefin group and thus may lower the LUMO energy (Fig. 1c). Thus, 1,3-enynes may serve as potential efficient dipolarophiles. Moreover, the presence of a carbon-carbon triple bond would allow further facile functionalization of the pyrrolidines produced via 1,3-dipolar cycloadditions.

Herein, we would like to disclose a copper(I)-catalyzed asymmetric 1,3-dipolar cycloaddition of azomethine ylides and 1,3-enynes. A series of chiral poly-substituted pyrrolidines were prepared in moderate to high yields with moderate to high diastereo- and enantioselectivities. Remarkably, 1,4-disubstituted 1,3-enynes also served as a suitable dipolarophile. Furthermore, the present methodology worked efficiently in the construction of both tetrasubstituted stereogenic carbon centers and chiral spiro pyrrolidines. The chemical calculations indicated that a concerted reaction pathway rather than a step-wise one was more reasonable for the present reaction. Moreover, the activation ability of the terminal olefin by several substituents was studied, which gave us a clear understanding of the reactivities of these weakly activated terminal olefins. At last, multiple transformations showcased the synthetic versatility of the triethylsilylethynyl group and thus complemented the substrate shortage of 4-alkyl-1,3-enynes.

## Results and discussion

**Fig. 2:**
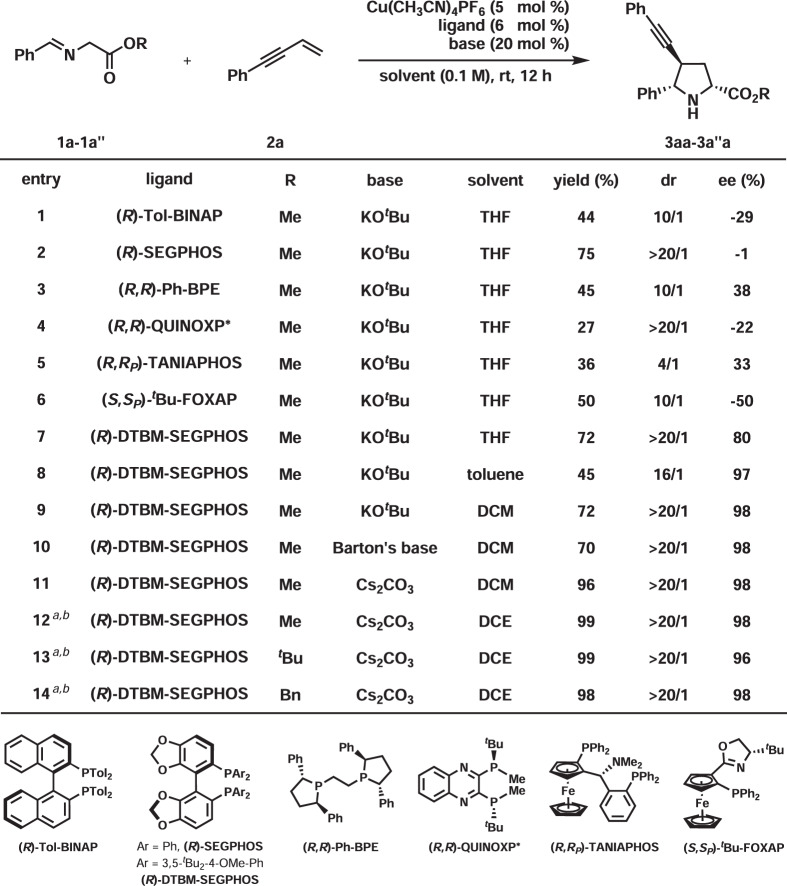
Optimization of reaction conditions. Reaction conditions: **1a–1a”** (0.13 mmol), **2a** (0.10 mmol). Yields were determined by ^1^H NMR analysis of the reaction crude mixture using CH_2_Br_2_ as an internal standard. Ee and dr were determined by chiral-stationary-phase HPLC analysis. THF = tetrahydrofuran, DCM = dichloromethane, DCE = ClCH_2_CH_2_Cl. Barton’s base = 2-^*t*^butyl-1,1,3,3-tetramethylguanidine. ^*a*^0.4 M. ^*b*^Isolated yield.

Initially, the reaction between iminoester **1a** and 1,3-enyne **2a** was investigated for the optimization of reaction conditions (Fig. 2). In the presence of 5 mol% Cu(CH_3_CN)_4_PF_6_, 6 mol% (*R*)-Tol-BINAP, and 20 mol% KO^*t*^Bu, **3aa** was produced in 44% yield with 10/1 dr and 29% ee (entry 1). Several bisphosphines and (*R*,*R*_*P*_)-^*t*^Bu-FOXAP were screened (entries 2–7), which identified (*R*)-DTBM-SEGPHOS as the best in terms of yield, dr, and ee (entry 7, 72% yield, >20/1 dr, 80% ee). Switching the solvent from THF to toluene led to increased enantioselectivity but with decreased both yield and dr (entry 8, 45% yield, 16/1 dr, 97% ee). DCM was found to be the most suitable solvent as **3aa** was obtained in 72% yield with >20/1 dr and 98% ee (entry 9). Barton’s base worked with similar efficiency (entry 10). However, weaker organic bases, such as Et_3_N and Cy_2_NMe, were found to be ineffective. The reaction with Cs_2_CO_3_ as the base afforded **3aa** in excellent yield together with excellent diastereo- and enantioselectivities (entry 11, 96% yield, >20/1 dr, 98% ee). The yield was further enhanced in DCE by increasing the substrate concentration from 0.1 to 0.4 M (entry 12, 99% yield, >20/1 dr, 98% ee). The reaction results of both ^*t*^butyl and benzyl esters (**1a′** and **1a′′**) were also satisfactory (entry 13, 99% yield, >20/1 dr, 96% ee; entry 14, 98% yield, >20/1 dr, 98% ee). It should be noted that only one type of regioisomers was observed in these reactions.

**Fig. 3:**
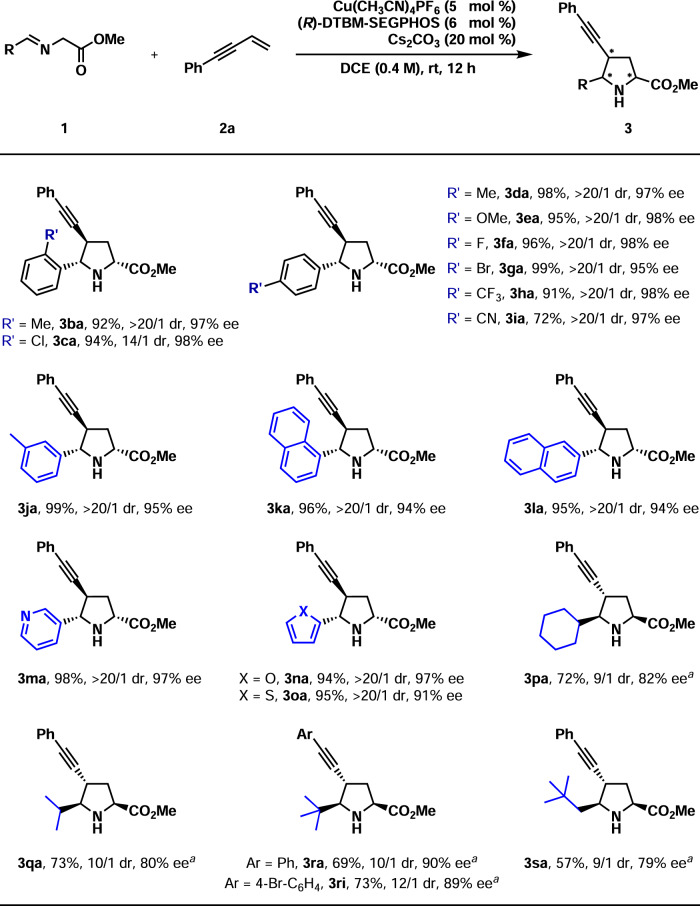
Substrate scope of azomethine ylides 1. Reaction conditions: **1** (0.26 mmol), **2a** (0.20 mmol). Isolated yields were reported. Ee and dr were determined by chiral-stationary-phase HPLC analysis. ^*a*^(*S*,S_P_)-^*t*^Bu-FOXAP was used instead of (*R*)-DTBM-SEGPHOS in DCE (1.0 M). 18 h.

Under the optimum reaction conditions, the substrate scope of iminoester (**1**) was studied with **2a** as the dipolarophile (Fig. 3). The reaction results were not very sensitive to both the substitution pattern and the electronic nature of the aryl groups as the corresponding products were isolated in generally high yields with excellent diastereo- and enantioselectivities (**3ba**–**3ja**, 72–99%, 14/1–20/1 dr, 95–98% ee). However, moderate dr (14/1) was observed in the case of **3ca** and moderate yield (72%) was obtained in the reaction of **3ia**. Moreover, 1-naphthyl and 2-naphthyl were well accepted (**3ka**–**3la**, 95–96%, >20/1 dr, 94% ee). Heteroaryl substituents were also well tolerated, and the corresponding products were obtained with excellent results (**3ma**–**3oa**, 94–98%, >20/1 dr, 91–97% ee). As for the aliphatic iminoesters (**1p**–**1s**), (*S*,S_P_)-^*t*^Bu-FOXAP was found as a better ligand than (*R*)-DTBM-SEGPHOS. Under higher substrate concentration (1.0 M), several aliphatic iminoesters reacted with **2a** smoothly to provide the corresponding products in acceptable results (**3pa**–**3sa** and **3ri**, 57–73%, 9/1–12/1 dr, 79–90% ee). However, the results of aliphatic iminoesters were inferior to the results of aromatic ones. The absolute configuration of **3ri** was determined by X-ray analysis of its derivative’s single crystals (for the details, see SI), which led to the assignment of the configurations of **3pa**–**3sa**.

**Fig. 4:**
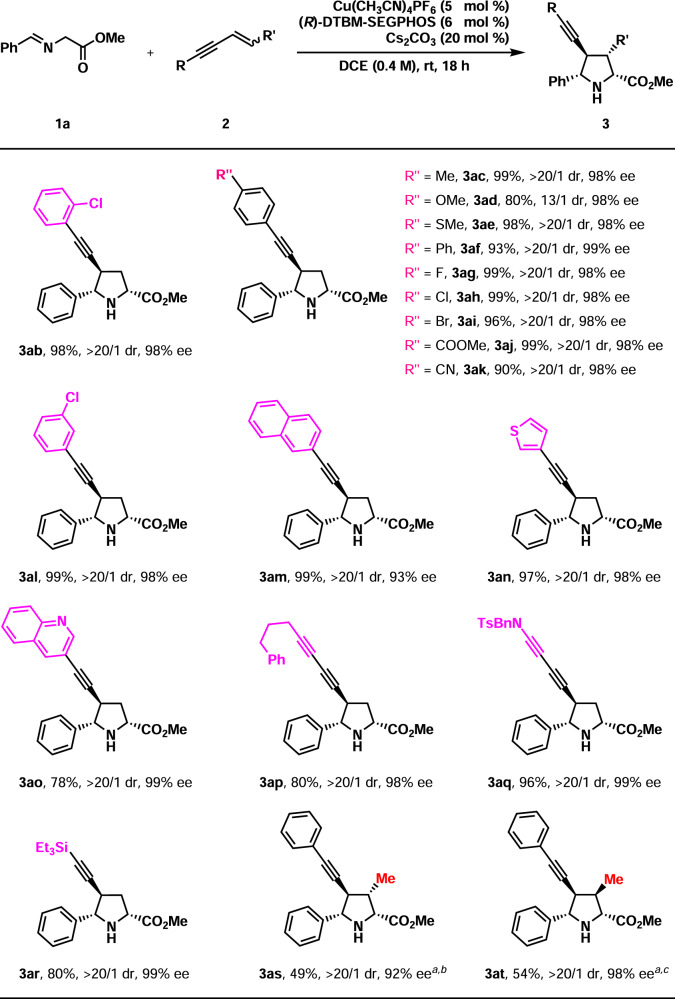
Substrate scope of 1,3-enynes 2. Reaction conditions: **1a** (0.26 mmol), **2** (0.20 mmol). Isolated yields were reported. Ee and dr were determined by chiral-stationary-phase HPLC analysis. ^*a*^10 mol% catalyst and 40 mol% Cs_2_CO_3_ were used. 1.0 M. 48 h. ^*b*^*trans*-1,3-enyne used. ^*c*^*cis*-1,3-enyne used.

Then the substrate scope of 1,3-enynes (**2**) was investigated with **1a** as the 1,3-dipole precursor (Fig. 4). At first, 1,3-enynes bearing a terminal olefin were evaluated (R′ = H). The R group could be various aromatic substituents (**3ab**–**3am**, 80–99%, 13/1–20/1 dr, 93–99% ee). Clearly, both the substitution pattern and the electronic nature of the aryl groups did not have a significant effect on yield, diastereo-, or enantioselectivities. However, the electron-donating group led to slightly decreased both yield and diastereoselectivity (**3ad**, 80%, 13/1 dr). Heteroaromatic substituents were well accepted in the R group (**3an**–**3ao**, 78–97%, >20/1 dr, 98–99% ee). When the R group was an aliphatic substituent (such as PhCH_2_CH_2_-), the reaction did not proceed at all. Fortunately, the R group could be either an additional alkynyl group or an *N*-Bn-sulfamide, and the products were isolated with satisfying results (**3ap**–**3aq**, 80–96%, >20/1 dr, 98–99% ee). Furthermore, 1,3-enyne **2r** containing a triethylsilyl group served as a satisfactory 1,3-dipolarophile, which was transformed to **3ar** in 80% yield with >20/1 dr and 99% ee. Evidently, the synthetically versatile triethylsilylethynyl group allows facile late-stage functionalization. Then *trans*-1,4-disubsituted 1,3-enyne **2s** (R = Ph, R′ = Me) was tried in the present reaction. Although the yield was moderate in the reaction with 10 mol% copper(I) catalyst and 40 mol% Cs_2_CO_3_, both the diastereo-, and enantioselectivities were very high (**3as**, 49%, >20/1 dr, 92% ee). Interestingly, *cis*-1,3-enyne **2t** (R = Ph, R′ = Me) exhibited similar performances in the present catalytic system (**3at**, 54%, >20/1 dr, 98% ee). It should be noted that **3as** and **3at** are diastereoisomers. The absolute configuration of **3ai** was determined unambiguously by X-ray crystal diffraction. The stereochemistry of other products (**3aa**–**3oa** and **3ab**–**3at**) was deduced by structural analogy. It should be pointed out that the relative configuration of **3as** was further confirmed by the X-ray analysis of its single crystals.

**Fig. 5:**
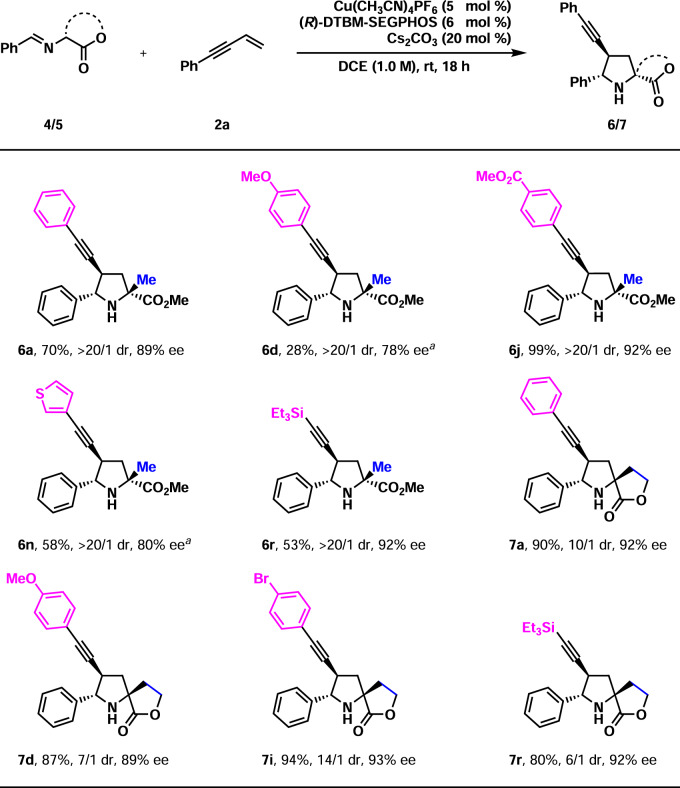
Preliminary investigation of the catalytic asymmetric construction of tetrasubstituted carbon. Reaction conditions: **4/5** (0.26 mmol), **2** (0.20 mmol). Isolated yields were reported. Ee and dr were determined by chiral-stationary-phase HPLC analysis. ^*a*^60 °C.

Subsequently, the present method was applied in the construction of tetrasubstituted stereocenters (Fig. 5). By introducing a methyl group at the α-position in the iminoester, substrate **4** was obtained for this purpose. The reactions of **4** and several 1,3-enynes proceeded nicely with excellent diastereo- and high enantioselectivities (**6a,** **6d,** **6j,** **6n**, and **6r**, >20/1 dr, 78–92% ee). However, yields varied considerably (28–99%). At 60 °C, as low as 28% yield was obtained for **6d**, and 58% yield was observed for **6n**. Undoubtedly, the yield increased significantly with the decrease in the electronic density of the 1,3-enynes. The method was further applied in the synthesis of chiral spiro compounds. Remarkably, four chiral spiro pyrrolidines were prepared in uniformly high yields and high enantioselectivity (**7a,** **7d,** **7i**, and **7r**, 80–94%, 89–93% ee). However, the diastereoselectivity was moderate (6/1–14/1 dr). The absolute configuration of **7i** was established by X-ray crystallographic analysis of its single crystals. The stereochemistry of other products (**6** and **7**) was assigned analogically.

**Fig. 6:**
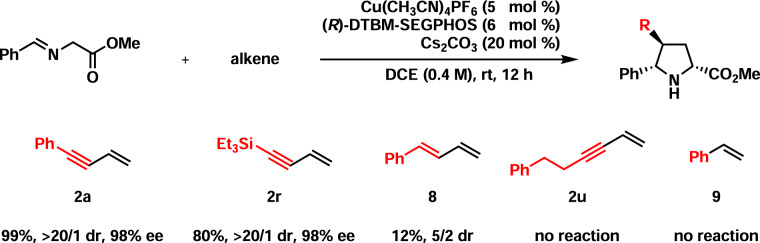
Cu(I)-catalyzed asymmetric 1,3-dipolar cycloadditions with several alkenes. Reaction conditions: **1a** (0.26 mmol), alkene (0.20 mmol). Isolated yields were reported. Ee and dr were determined by chiral-stationary-phase HPLC analysis.

With the present copper(I)-catalyzed asymmetric 1,3-dipolar cycloaddition as a tool, several olefins, including but-3-en-1-yn-1-ylbenzene (**2a**), but-3-en-1-yn-1-yltriethylsilane (**2r**), (*E*)-buta-1,3-dien-1-ylbenzene (**8**), hex-5-en-3-yn-1-ylbenzene (**2u**), and styrene (**9**), were evaluated. As shown in Fig. 6, **2a** exhibited the highest reactivity, possibly due to the double activation of the olefin by both the conjugated alkyne and the conjugated phenyl groups. **2r** displayed the second-highest reactivity as both the conjugated alkyne group and the triethylsilyl group activated the olefin. Clearly, the phenyl group had a stronger activation effect on the conjugated terminal olefin than the triethylsilyl group. The reaction with **8** was very slow and led to a low yield (12%), indicating that the activation effect of the conjugated alkene group on the terminal olefin is much weaker than the conjugated alkyne group. Such an experimental fact could be rationalized by the realization that two π-bonds are present in the alkyne while only one π-bond is present in the alkene, which leads to superior activation of the terminal olefin in **2a** to the one in **8**. **2u** and **9** remained intact, suggesting that the activation of the olefin singly by the alkyne or the phenyl is not enough for the present 1,3-dipolar cycloaddition.

**Fig. 7:**
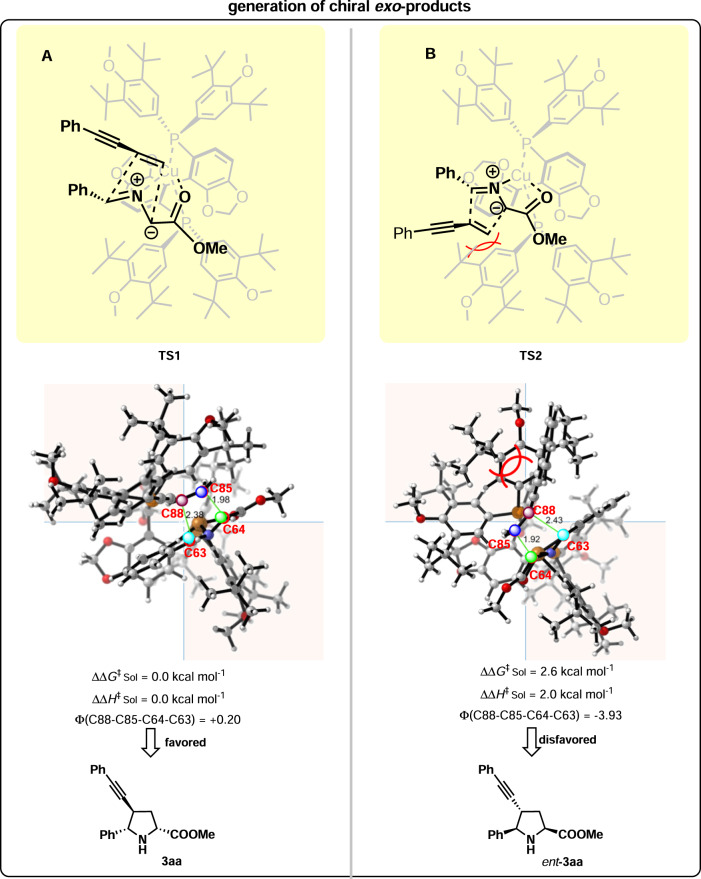
DFT Calculation results: generation of chiral *exo*-products. **A** Generation of **3aa**. **B** Generation of *ent*-**3aa**.

In literature^36^, it was proposed that the copper(I)-catalyzed 1,3-dipolar cycloaddition of strongly activated olefins prefers a stepwise mechanism while the reaction of weakly activated olefins prefers a concerted mechanism. Thus it is believed that the present reaction proceeds preferentially in a concerted mechanism. To elucidate the mechanistic details, DFT calculations were performed by employing iminoester **1a**, 1,3-enyne **2a**, ligand (*R*)-DTBM-SEGPHOS, and Cu(CH_3_CN)_4_PF_6_ as depicted in Fig. 7. The computational results showed that the present reaction proceeded preferentially in a concerted mechanism rather than the stepwise mechanism (for details, see SI). Based on the concerted mechanism, the stereoselectivity-determining cyclization step was revealed, and the relative free energies of the enantio-isomeric transition states and their structures were obtained, which are depicted in Fig. 7. **TS1** (leading to the desired product **3aa**) is more favorable by 2.6 kcal mol^−1^ than **TS2** (leading to *ent*-**3aa**). Evidently, the calculation results are in good agreement with the experimental enantioselectivity (98% ee), which validates the calculated concerted reaction pathway. Through analysis of the structures of the competing transition states **TS1** and **TS2**, the close distance between one of the ^*t*^Bu groups in (*R*)-DTBM-SEGPHOS and the alkynyl and phenyl groups in 1,3-enyne **2a** in the disfavored **TS2** leads to more van der Waals interactions (for details, see SI) and bigger dihedral angle of C88–C85–C64–C63 in the relatively unfavored cyclization transition state, which results in the observed absolute stereochemistry.

**Fig. 8:**
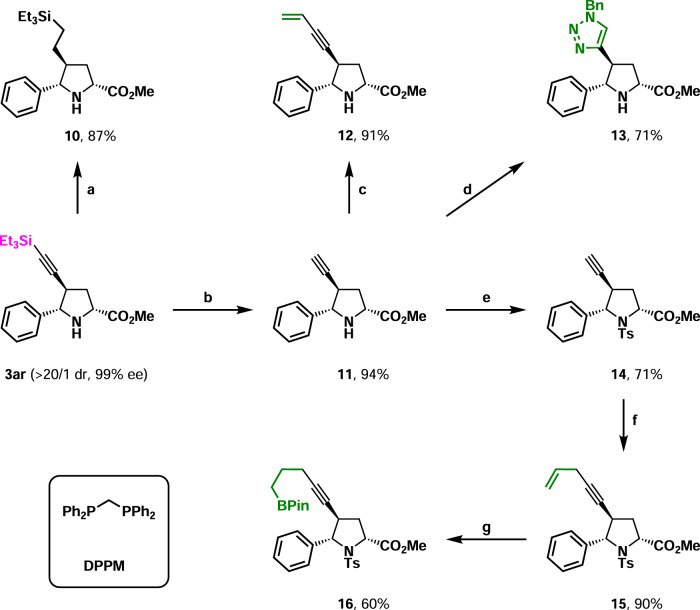
Transformations of product 3p. **a** Reduction. Pd/C, H_2_ (1 atm), MeOH, rt. **b** Desilylation. TBAF, THF, 0 °C. **c** Cross-coupling. Vinylbromide, Pd(PPh_3_)_2_Cl_2_, CuI, PPh_3_, NEt_3_, THF, 50 °C. **d** Click reaction. BnN_3_, NaAsc, CuSO_4_, H_2_O/DMF, 40 °C. **e** Protection. TsCl, Et_3_N, DMAP, DCM, 0 °C to rt. **f** Cross-coupling. Allylbromide, CuI, TBAI, K_2_CO_3_, DMF, rt. **g** Ir-catalyzed hydroboration. [Ir(COD)_2_Cl_2_]_2_, DPPM, HBPin, DCM, rt.

At last, transformations were performed with **3ar** as a model substrate (Fig. 8). The catalytic hydrogenation of **3ar** at room temperature provided silane **10** in 87% yield. The deprotection of **3ar** afforded terminal alkyne **11** in 94% yield. The Sonogashira coupling of **11** with vinylbromide furnished 1,3-enyne **12** in 91% yield. Notably, the coupling proceeded smoothly in the presence of a free secondary amine moiety. Moreover, the click reaction of **11** and benzyl azide occurred nicely to deliver triazole **13** in 71% yield. The protection of the secondary amine moiety underwent easily gave terminal alkyne **14**, which was ready for the CuI-catalyzed cross-coupling reaction between terminal alkyne and allyl bromide^74^. 1,4-Enyne **15** was synthesized in 90% yield. The hydroboration of the terminal olefin^75^ in **15** was readily performed to offer boronate **16** in 60% yield. It should be mentioned that further transformations based on silane, 1,3-enyne, terminal alkyne, terminal olefin, and boronate were straightforward. Moreover, some of the above transformations compensated for the inertness of 4-alkyl-1,3-enynes in the present 1,3-dipolar cycloaddition.

## Methods

General procedures for copper(I)-catalyzed asymmetric 1,3-dipolar cycloaddition of 1,3-enynes: a dried 25 ml schlenk tube equipped with a magnetic stirring bar was charged with [Cu(MeCN)_4_]PF_6_ (3.7 mg, 0.01 mmol, 0.05 equiv), Cs_2_CO_3_ (13.0 mg, 0.04 mmol, 0.2 equiv), and (*R*)-DTBM-SEGPHOS (14.0 mg, 0.012 mmol, 0.06 equiv) in a glove box under Ar atmosphere. Anhydrous DCE (0.5 ml) was added via a syringe. The mixture was stirred for 30 min at room temperature. Then imine esters (0.26 mmol, 1.3 equiv) and 1,3-enynes (0.2 mmol, 1.0 equiv) were added sequentially. The resulting mixture was stirred at room temperature for 12 or 18 h. Then the reaction mixture was directly purified by silica gel column chromatography to give the desired product.

### Supplementary information

Supplementary Information

Description of additional supplementary files

Supplementary Data 1

## Data Availability

All data are available from the authors upon request. Supplementary information and chemical compound information are available along with the online version of the paper. The X-ray crystallographic coordinates for structures reported in this study have been deposited at the Cambridge Crystallographic Data Center (CCDC) under deposition numbers 2093924, 2111662, 2106096, and 2111496. These data can be obtained free of charge from The Cambridge Crystallographic Data Centre via www.ccdc.cam.ac.uk/data_request/cif. Cartesian coordinates of the optimized structures are provided in a supplementary data file.
