# Supplementary material for: Copper(I)-catalyzed asymmetric 1,3-dipolar cycloaddition of 1,3-enynes and azomethine ylides
Source: Nat Commun. 2023 Aug 4;14:4688. doi: 10.1038/s41467-023-40409-4 (PMC10403559; doi:10.1038/s41467-023-40409-4)
Supplement: Supplementary file 1 — Supplementary Information [file 41467_2023_40409_MOESM1_ESM.pdf]

Supplementary Information

of

Copper(I)-Catalyzed Asymmetric 1,3-Dipolar Cycloaddition of 1,3-Enynes and Azomethine Ylides

Bo-Ran Wang[1,2], Yan-Bo Li[2], Qi Zhang[2], Dingding Gao[1], Ping Tian[1,*], Qinghua Li[1,*], and Liang Yin[1,2,*]

[1]*The Research Center of Chiral Drugs, Innovation Research Institute of Traditional Chinese Medicine and China-Thailand Joint Research Institute of Natural Medicine, Shanghai University of Traditional Chinese Medicine, 1200 Cailun Road, shanghai 201203, China*

[2]*CAS Key Laboratory of Synthetic Chemistry of Natural Substances, Centre for Excellence in Molecular Synthesis, Shanghai Institute of Organic Chemistry, University of Chinese Academy of Sciences, Chinese Academy of Sciences, 345 Lingling Road, Shanghai 200032, China.*

liangyin@sioc.ac.cn

Table of Contents

1. Supplementary Methods 1

1.1 General Information 1

1.2 Preparation of 1,3-Enynes 1

1.3 General Procedures for Copper(I)-Catalyzed Asymmetric 1,3-Dipolar Cycloaddition of 1,3-Enynes 2

2. Supplementary Discussion 54

2.1 Computational Methods 54

2.2 Determination of the Absolute Configurations of Products 56

2.3 Transformations of the Product 63

3. Supplementary Figures 67

3.1 ^{1}H, ^{13}C, and ^{19}F NMR Spectra of New Compounds 67

4. Supplementary References 191

1. Supplementary Methods

1.1 General Information

All reagents were obtained commercially unless otherwise noted. Nuclear Magnetic Resonance (NMR) spectra were acquired on Agilent 400, Bruker 400 or Bruker 500 instrument operating at 400 or 500, 101 or 126, and 376 MHz for ^{1}H, ^{13}C, and ^{19}F, respectively. For ^{1}H NMR, chemical shifts were reported in δ ppm referenced to an internal $SiMe_4$ standard. For ^{19}F NMR, $CFCl_3$ was used as the reference with chemical shift at 0 ppm. For ^{13}C NMR, chemical shifts were reported in the scale relative to NMR solvent ($CDCl_3$: δ 77.0 ppm) as an internal reference. Multiplicities are reported using the following abbreviations: s = singlet, d = doublet, t = triplet, q = quartet, m = multiplet, br = broad signal. High-resolution mass spectra (ESI) were measured on Agilent 6200 Series TOF/6500 Series. Infrared (IR) spectra were recorded on Thermo Scientific Nicolet iS5 FT-IR. Optical rotation was measured on an Anton Paar Mcp 5500 polarimeter. HPLC analysis was conducted on a Shimadzu HPLC system equipped with Daicel chiral-stationary-phase columns (ϕ 4.6 mm × 250 mm).

1.2 Preparation of 1,3-Enynes

1,3-enynes **2a**[1], **2b**[4], **2c**[1], **2d**[1], **2e**[6], **2f**[3], **2g**[2], **2h**[2], **2i**[1], **2j**[5], **2k**[1], **2l**[3], **2m**[2], **2n**[2], **2q**[16], **2r**[7], **2s**[8], **2t**[5] , and **2u**[1] are known compounds.

Azomethine ylides **1a**[9], **1a'**[14], **1a''**[14], **1b**[9], **1c**[10], **1d**[9], **1e**[9], **1f**[9], **1g**[9], **1h**[10], **1i**[11], **1j**[9], **1k**[9], **1l**[9], **1m**[11], **1n**[9], **1o**[10], **1p**[10], **1q**[10], **1r**[13], **1s**[12], **4**[10], and **5**[15] are known compounds.

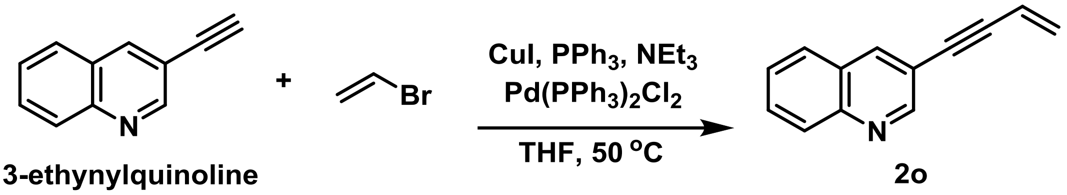

Supplementary Fig. 1. Preparation of **2o**

A 25 ml flask equipped with a magnetic stirring bar was charged with PPh_3 (67 mg, 0.26 mmol, 0.04 equiv), CuI (49 mg, 0.26 mmol, 0.04 equiv), and $Pd(PPh_3)_2Cl_2$ (119 mg, 0.156 mmol, 0.024equiv) in a glove box under Ar atmosphere. Vinyl bromide (1M in THF, 13 ml, 2.0 equiv), NEt_3 (3.28 g, 32.5 mmol, 5.0 equiv), and 3-ethynylquinoline (1 g, 6.5 mmol, 1.0 equiv) were added before the reaction mixture was heated to 50 °C and the reaction mixture was then stirred for 12 hours. The precipitate was filtered off and washed with EA. After removal of volatiles under reduced pressure, the residue was purified by silica gel column chromatography to afford **2o** (1.164 g, pale yellow oil, 99% yield).

^{1}H NMR (400 MHz, $CDCl_3$) δ 8.92 (d, *J* = 2.1 Hz, 1H), 8.23 (d, *J* = 2.1 Hz, 1H), 8.09 (dd, *J* = 8.4, 1.1 Hz, 1H), 7.78 (dd, *J* = 8.1, 1.5 Hz, 1H), 7.72 (ddd, *J* = 8.4, 6.9, 1.5 Hz, 1H), 7.56 (ddd, *J* = 8.1, 6.9, 1.2 Hz, 1H), 6.08 (dd, *J* = 17.5, 11.2 Hz, 1H), 5.84 (dd, *J* = 17.5, 2.0 Hz, 1H), 5.64 (dd, *J* = 11.2, 2.0 Hz, 1H) ppm.

^{13}**C NMR** (126 MHz, $CDCl_3$) δ 151.86, 146.62, 138.11, 129.94, 129.19, 127.98, 127.44, 127.12, 127.04, 117.14, 116.62, 91.13, 87.06 ppm.
HRMS (ESI) m/z [M+H]$^+$: calcd for $C_{13}H_{10}N^+$ 180.0808; found 180.0807.
IR (film): ν_{max} (cm^{-1}) 3063, 3009, 1608, 1566, 1416, 1341, 992, 908, 783, 751.

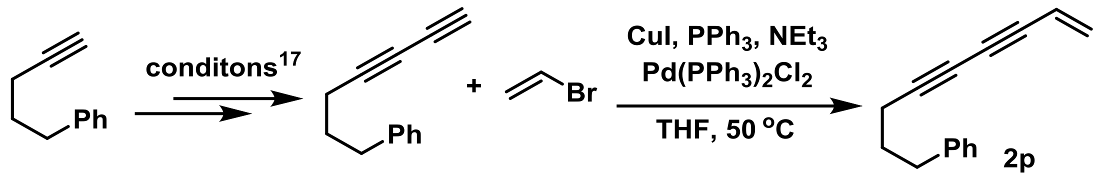

Supplementary Fig. 2. Preparation of **2p**

A 25 ml flask equipped with a magnetic stirring bar was charged with PPh_3 (20.9 mg, 0.08 mmol, 0.04 equiv), CuI (15.2 mg, 0.08 mmol, 0.04 equiv), and $Pd(PPh_3)_2Cl_2$ (33.7 mg, 0.048 mmol, 0.024equiv) in a glove box under Ar atmosphere. Vinyl bromide (1M in THF, 4 ml, 2.0 equiv), NEt_3 (1.01 g, 10 mmol, 5.0 equiv), and hepta-4,6-diyn-1-ylbenzene (336 mg, 2 mmol, 1.0 equiv) which is prepared according to the literature[17] from pent-4-yn-1-ylbenzene were added before the reaction mixture was heated to 50 °C and the reaction mixture was then stirred for 12 hours. The precipitate was filtered off and washed with EA. After removal of volatiles under reduced pressure, the residue was purified by silica gel column chromatography to afford **2p** (160 mg, pale yellow oil, 41% yield).
^{1}**H NMR** (400 MHz, $CDCl_3$) δ 7.32 – 7.26 (m, 2H), 7.22 – 7.16 (m, 3H), 5.88 – 5.70 (m, 2H), 5.59 (dd, J = 9.7, 3.5 Hz, 1H), 2.73 (t, J = 7.5 Hz, 2H), 2.33 (t, J = 7.0 Hz, 2H), 1.87 (p, J = 7.0 Hz, 2H) ppm.
^{13}**C NMR** (126 MHz, $CDCl_3$) δ 141.12, 129.74, 128.49, 128.37, 125.98, 116.26, 84.21, 74.96, 73.71, 65.42, 34.62, 29.72, 18.86 ppm.
HRMS (ESI) m/z [M+H]$^+$: calcd for $C_{15}H_{15}^+$ 195.1168; found 195.1168.
IR (film): ν_{max} (cm^{-1}) 3060, 2925, 2234, 1601, 1495, 922, 744, 698.

1.3 General Procedures for Copper(I)-Catalyzed Asymmetric 1,3-Dipolar Cycloaddition of 1,3-Enynes

General Procedure A:

A dried 25 ml schlenk tube equipped with a magnetic stirring bar was charged with $[Cu(MeCN)_4]PF_6$ (3.7 mg, 0.01 mmol, 0.05 equiv), Cs_2CO_3 (13.0 mg, 0.04 mmol, 0.2 equiv), and (*R*)-DTBM-SEGPHOS (14.0 mg, 0.012 mmol, 0.06 equiv) in a glove box under Ar atmosphere. Anhydrous DCE (0.5 ml) was added via a syringe. The mixture was stirred for 30 minutes at room temperature. Then imine esters (0.26 mmol, 1.3 equiv) and 1,3-enynes (0.2 mmol, 1.0 equiv) were added sequentially. The resulting nixture was stirring at room temperature for 12 or 18 hours. Then the reaction mixture was directly purified by silica gel column chromatography to give the desired product.

General Procedure B:

A dried 25 ml schlenk tube equipped with a magnetic stirring bar was charged with $[Cu(MeCN)_4]PF_6$ (3.7 mg, 0.01 mmol, 0.05 equiv), Cs_2CO_3 (13.0 mg, 0.04 mmol, 0.2 equiv), and

(*R*)-DTBM-SEGPHOS (14.0 mg, 0.012 mmol, 0.06 equiv) in a glove box under Ar atmosphere. Anhydrous DCE (0.2 ml) was added via a syringe. The mixture was stirred for 30 minutes. Then imine esters (0.26 mmol, 1.3 equiv) and 1,3-enynes (0.2 mmol, 1.0 equiv) were added sequentially. The resulting mixture was stirring at room temperature for 18 hours. Then the reaction mixture was directly purified by silica gel column chromatography to give the desired product.

General Procedure C:

A dried 25 ml schlenk tube equipped with a magnetic stirring bar was charged with $[Cu(MeCN)_4]PF_6$ (3.7 mg, 0.01 mmol, 0.05 equiv), Cs_2CO_3 (13.0 mg, 0.04 mmol, 0.2 equiv), and (S,S_p)-^{t}Bu-PHOXAP (5.9 mg, 0.012 mmol, 0.06 equiv) in a glove box under Ar atmosphere. Anhydrous DCE (0.2 ml) was added via a syringe. The mixture was stirred for 30 minutes. Then imine esters (0.26 mmol, 1.3 equiv) and 1,3-enynes (0.2 mmol, 1.0 equiv) were added sequentially. The resulting mixture was stirring at room temperature for 18 hours. Then the reaction mixture was cooled to room temperature and directly purified by silica gel column chromatography to give the desired product.

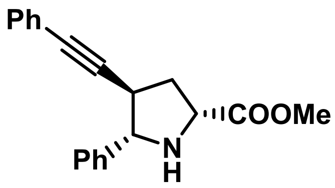

3aa

3aa: Procedure A, pale yellow oil, 60.4 mg, 99% yield, >20/1 dr (Diastereoselectivity was determined by ^{1}H NMR analysis of reaction crude mixture).

^{1}H NMR (400 MHz, $CDCl_3$) δ 7.60–7.53 (m, 2H), 7.42–7.33 (m, 4H), 7.33–7.29 (m, 1H), 7.29–7.24 (m, 3H), 4.22 (d, J = 9.1 Hz, 1H), 4.09 (dd, J = 9.3, 4.4 Hz, 1H), 3.80 (s, 3H), 2.94 (td, J = 9.5, 7.9 Hz, 1H), 2.54 (ddd, J = 12.6, 7.9, 4.5 Hz, 1H), 2.43 (dt, J = 13.0, 9.5 Hz, 1H) ppm.

^{13}C NMR (101 MHz, $CDCl_3$) δ 175.01, 140.88, 131.56, 128.49, 128.15, 127.85, 127.74, 126.83, 123.32, 88.99, 83.03, 69.47, 58.73, 52.31, 38.96, 37.53 ppm.

HRMS (ESI) m/z [M+H]$^{+}$: calcd $C_{20}H_{20}NO_2^{+}$ 306.1489; found 306.1491.

IR (film): ν_{max} (cm^{-1}) 3348, 3030, 2950, 1736, 1599, 1490, 1453, 1208, 756, 692.

Optical rotation: $[\alpha]_D^{25}$ = 160.46 (c = 1.00, $CHCl_3$, 98% ee).

HPLC: DAICEL CHIRALPAK IA-3, hexane/i-PrOH = 40/10, flow rate: 0.5 mL/min, λ = 254 nm, t_R(minor) = 12.9 min, t_R(major) = 11.5 min, 98% ee.

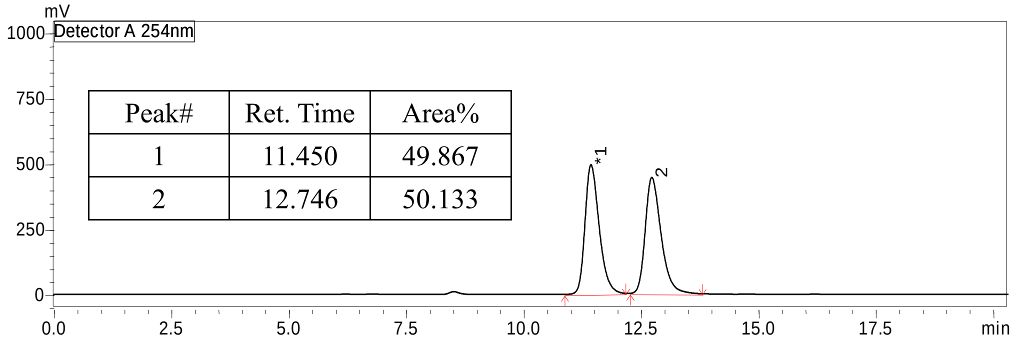

Peak#	Ret. Time	Area%
1	11.450	49.867
2	12.746	50.133

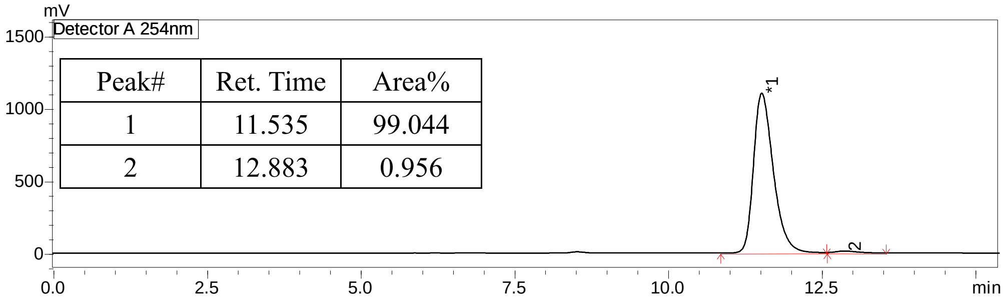

Peak#	Ret. Time	Area%
1	11.535	99.044
2	12.883	0.956

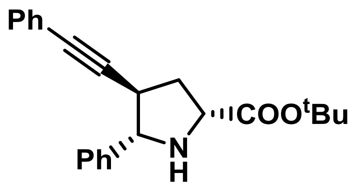

3a'a

3a'a: Procedure A, colorless oil, 69.7 mg, 99% yield, >20/1 dr (Diastereoselectivity was determined by ^{1}H NMR analysis of reaction crude mixture).

^{1}H NMR (400 MHz, $CDCl_3$) δ 7.57 (d, *J* = 7.3 Hz, 2H), 7.42–7.33 (m, 4H), 7.31 (dd, *J* = 7.2, 1.3 Hz, 1H), 7.29–7.24 (m, 3H), 4.19 (d, *J* = 9.2 Hz, 1H), 4.05–3.84 (m, 1H), 2.89 (q, *J* = 9.1 Hz, 1H), 2.53–2.37 (m, 2H), 1.52 (s, 9H) ppm.

^{13}C NMR (101 MHz, $CDCl_3$) δ 173.89, 140.86, 131.53, 128.46, 128.10, 127.78, 127.69, 126.81, 123.33, 89.11, 83.00, 81.50, 69.60, 59.60, 38.87, 38.11, 28.00 ppm.

HRMS (ESI) m/z [M+H]$^+$: calcd $C_{23}H_{26}NO_2^+$ 348.1958; found 348.1958.

IR (film): ν_{max} (cm^{-1}) 3353, 3030, 2977, 1727, 1599, 1490, 1455, 1152, 847, 756, 692.

Optical rotation: $[\alpha]_D^{25}$ = 139.78 (*c* = 2.76, $CHCl_3$, 96% ee).

HPLC: DAICEL CHIRALPAK IE, hexane/*i*-PrOH = 35/15, flow rate: 0.5 mL/min, λ = 254 nm, t_R(minor) = 10.5 min, t_R(major) = 9.3 min, 96% ee.

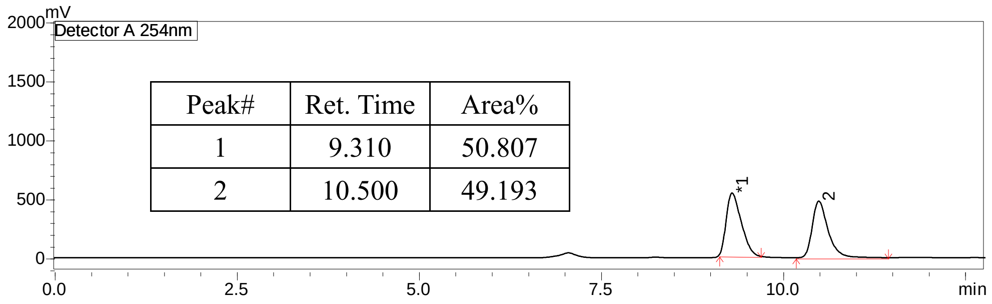

Peak#	Ret. Time	Area%
1	9.310	50.807
2	10.500	49.193

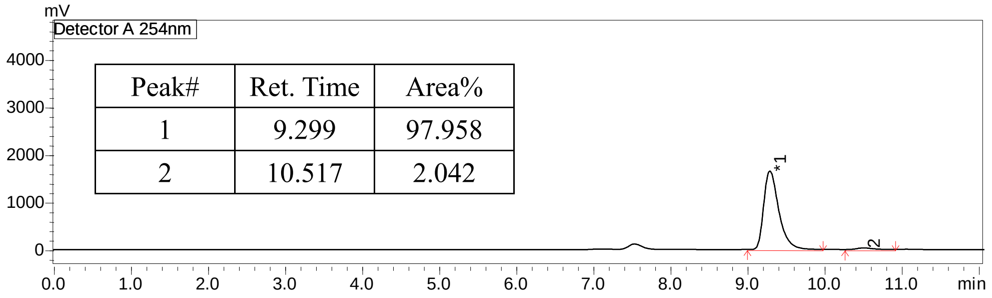

Peak#	Ret. Time	Area%
1	9.299	97.958
2	10.517	2.042

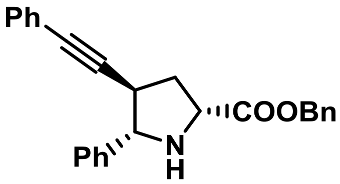

3a"a

3a"a: Procedure A, colorless oil, 75.1 mg, 98% yield, >20/1 dr (Diastereoselectivity was determined by ^{1}H NMR analysis of reaction crude mixture).

^{1}H NMR (400 MHz, $CDCl_3$) δ 7.59–7.51 (m, 2H), 7.43–7.32 (m, 9H), 7.32–7.28 (m, 1H), 7.28–7.23 (m, 3H), 5.23 (s, 2H), 4.23 (d, J = 9.1 Hz, 1H), 4.18–4.06 (m, 1H), 2.93 (td, J = 9.6, 7.9 Hz, 1H), 2.55 (ddd, J = 12.3, 7.8, 4.2 Hz, 1H), 2.44 (dt, J = 13.0, 9.6 Hz, 1H) ppm.

^{13}C NMR (126 MHz, $CDCl_3$) δ 174.32, 140.81, 135.46, 131.50, 128.58, 128.44, 128.37, 128.20, 128.11, 127.81, 127.69, 126.79, 123.22, 88.87, 83.02, 69.37, 66.93, 58.75, 38.95, 37.47 ppm.

HRMS (ESI) m/z [M+H]$^{+}$: calcd $C_{26}H_{24}NO_2^{+}$ 382.1802; found 382.1801.

IR (film): ν_{max} (cm^{-1}) 3369, 3062, 3031, 2951, 1735, 1598, 1490, 1454, 1193, 1026, 755, 697.

Optical rotation: $[\alpha]_D^{25}$ = 146.13 (c = 2.32, $CHCl_3$, 98% ee).

HPLC: DAICEL CHIRALPAK IA, hexane/i-PrOH = 44/6, flow rate: 0.5 mL/min, λ = 254 nm, t_R(minor) = 19.4 min, t_R(major) = 16.7 min, 98% ee.

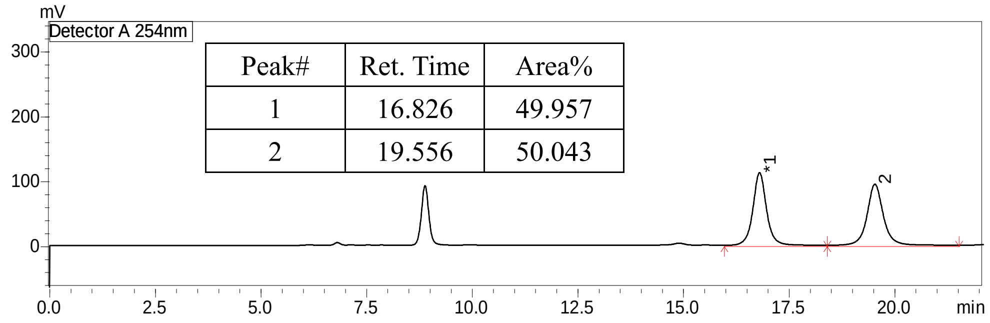

Peak#	Ret. Time	Area%
1	16.826	49.957
2	19.556	50.043

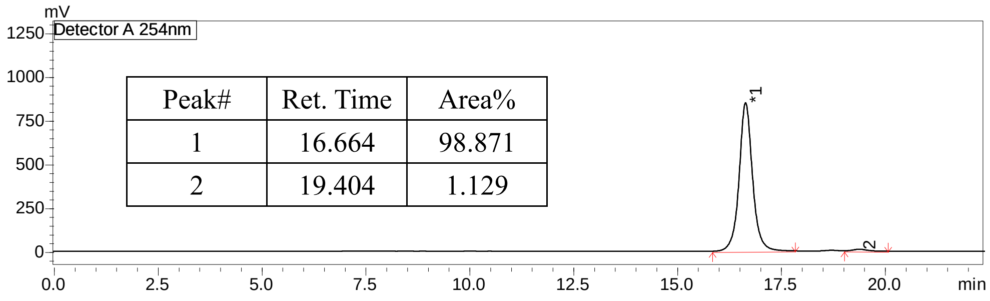

Peak#	Ret. Time	Area%
1	16.664	98.871
2	19.404	1.129

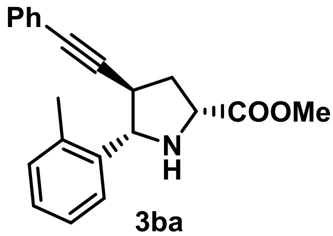

3ba

3ba: Procedure A, colorless oil, 58.8 mg, 92% yield, >20/1 dr (Diastereoselectivity was determined by ^{1}H NMR analysis of reaction crude mixture).

^{1}H NMR (400 MHz, $CDCl_3$) δ 7.66 (d, J = 7.1 Hz, 1H), 7.36–7.30 (m, 2H), 7.28–7.23 (m, 4H), 7.21–7.14 (m, 2H), 4.58 (d, J = 8.5 Hz, 1H), 4.12 (dd, J = 8.9, 4.9 Hz, 1H), 3.81 (s, 3H), 2.98 (q, J = 8.5 Hz, 1H), 2.51 (s, 4H), 2.49–2.42 (m, 1H) ppm.

^{13}C NMR (126 MHz, $CDCl_3$) δ 173.95, 139.20, 136.58, 131.43, 130.30, 128.51, 127.79, 127.28, 126.40, 125.55, 124.13, 89.15, 84.03, 65.35, 57.70, 54.40, 41.83, 37.46, 22.24 ppm.

HRMS (ESI) m/z [M+H]$^+$: calcd $C_{21}H_{22}NO_2^+$ 320.1645; found 320.1645.

IR (film): ν_{max} (cm^{-1}) 3353, 3053, 2950, 1736, 1598, 1490, 1208, 755, 691.

Optical rotation: $[\alpha]_D^{25}$ = 101.59 (c = 2.21, $CHCl_3$, 97% ee).

HPLC: DAICEL CHIRALPAK IE, hexane/i-PrOH = 46/4, flow rate: 0.5 mL/min, λ = 254 nm, t_R(minor) = 20.0 min, t_R(major) = 17.7 min, 97% ee.

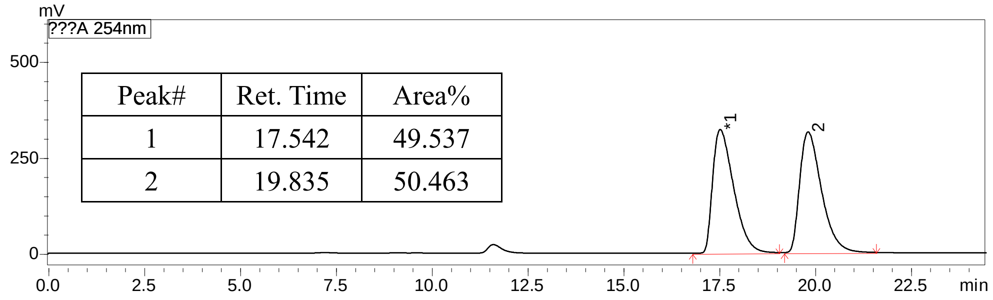

Peak#	Ret. Time	Area%
1	17.542	49.537
2	19.835	50.463

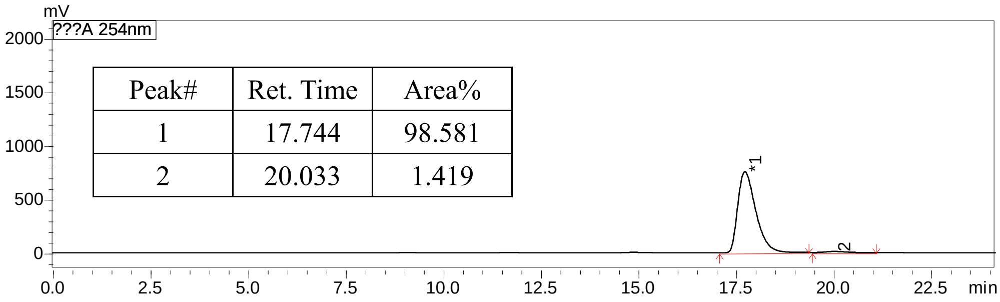

Peak#	Ret. Time	Area%
1	17.744	98.581
2	20.033	1.419

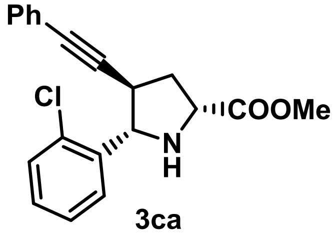

3ca

3ca: Procedure A, colorless oil, 64.1 mg, 94% yield, 14/1 dr (Diastereoselectivity was determined by ^{1}H NMR analysis of reaction crude mixture).

^{1}H NMR (400 MHz, $CDCl_3$) δ 7.81 (dd, *J* = 7.8, 1.7 Hz, 1H), 7.41–7.34 (m, 3H), 7.34–7.26 (m, 4H), 7.21 (td, *J* = 7.6, 1.7 Hz, 1H), 4.86 (d, *J* = 7.0 Hz, 1H), 4.21 (t, *J* = 7.3 Hz, 1H), 3.80 (s, 3H), 3.10 (q, *J* = 7.3 Hz, 1H), 2.45–2.37 (m, 2H) ppm.

^{13}C NMR (126 MHz, $CDCl_3$) δ 174.23, 139.93, 133.67, 131.54, 129.42, 128.55, 128.25, 128.11, 127.82, 127.10, 121.30, 90.44, 84.12, 67.83, 59.41, 53.58, 39.68, 36.80 ppm.

HRMS (ESI) m/z [M+H]$^+$: calcd $C_{20}H_{19}ClNO_2^+$ 340.1099; found 340.1099.

IR (film): ν_{max} (cm^{-1}) 3377, 3061, 2950, 1739, 1597, 1441, 1205, 755, 691.

Optical rotation: $[\alpha]_D^{25}$ = 57.06 (*c* = 2.00, $CHCl_3$, 98% ee).

HPLC: DAICEL CHIRALPAK IA-3, hexane/*i*-PrOH = 43/7, flow rate: 0.5 mL/min, λ = 254 nm, t_R(minor) = 16.4 min, t_R(major) = 14.1 min, 98% ee.

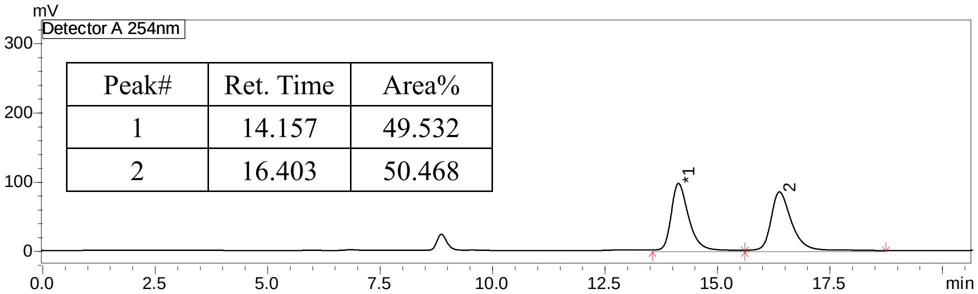

Peak#	Ret. Time	Area%
1	14.157	49.532
2	16.403	50.468

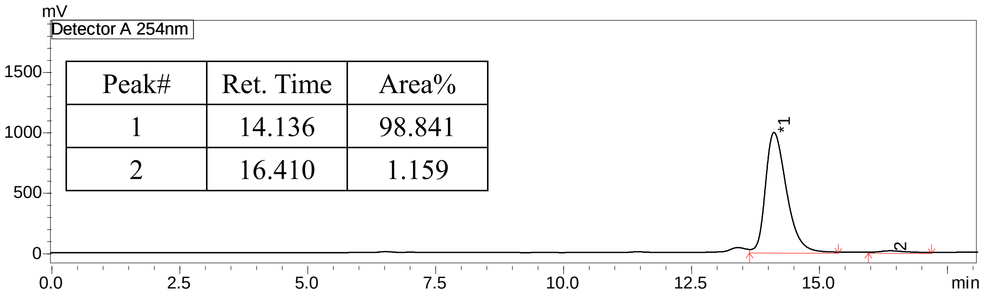

Peak#	Ret. Time	Area%
1	14.136	98.841
2	16.410	1.159

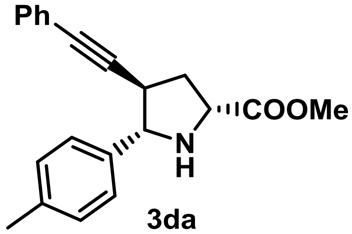

3da

3da: Procedure A, colorless oil, 62.6 mg, 98% yield, >20/1 dr (Diastereoselectivity was determined by ^{1}H NMR analysis of reaction crude mixture).

^{1}H NMR (400 MHz, $CDCl_3$) δ 7.48–7.40 (m, 2H), 7.38–7.32 (m, 2H), 7.26 (m, 3H), 7.18 (d, *J* = 7.8 Hz, 2H), 4.18 (d, *J* = 9.1 Hz, 1H), 4.07 (dd, *J* = 9.3, 4.4 Hz, 1H), 3.80 (s, 3H), 2.91 (td, *J* = 9.7, 8.0 Hz, 1H), 2.53 (ddd, *J* = 12.5, 7.9, 4.4 Hz, 1H), 2.49–2.39 (m, 1H), 2.35 (s, 3H) ppm.

^{13}C NMR (101 MHz, $CDCl_3$) δ 174.96, 137.64, 137.32, 131.49, 129.13, 128.07, 127.75, 126.67, 123.28, 88.97, 82.90, 69.21, 58.61, 52.23, 38.84, 37.50, 21.03 ppm.

HRMS (ESI) m/z [M+H]$^+$: calcd $C_{21}H_{22}NO_2^+$ 320.1645; found 320.1645.

IR (film): ν_{max} (cm^{-1}) 3349, 2950, 2922, 1735, 1653, 1515, 1436, 1209, 1111, 812, 757, 691.

Optical rotation: $[\alpha]_D^{25}$ = 180.31 (*c* = 2.51, $CHCl_3$, 97% ee).

HPLC: DAICEL CHIRALPAK IA, hexane/*i*-PrOH = 44/6, flow rate: 0.5 mL/min, λ = 254 nm, t_R(minor) = 14.7 min, t_R(major) = 12.9 min, 97% ee.

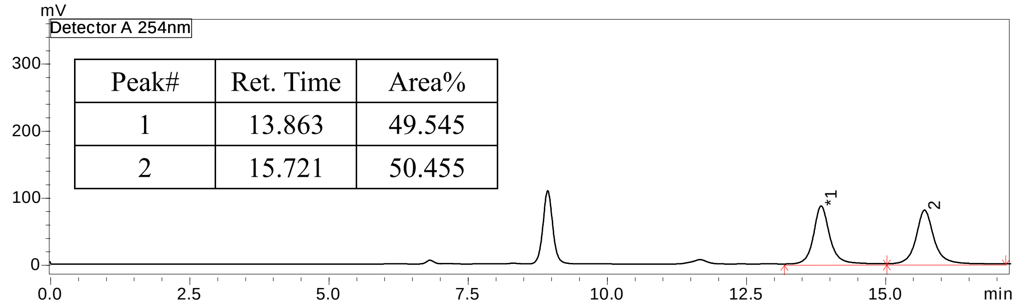

Peak#	Ret. Time	Area%
1	13.863	49.545
2	15.721	50.455

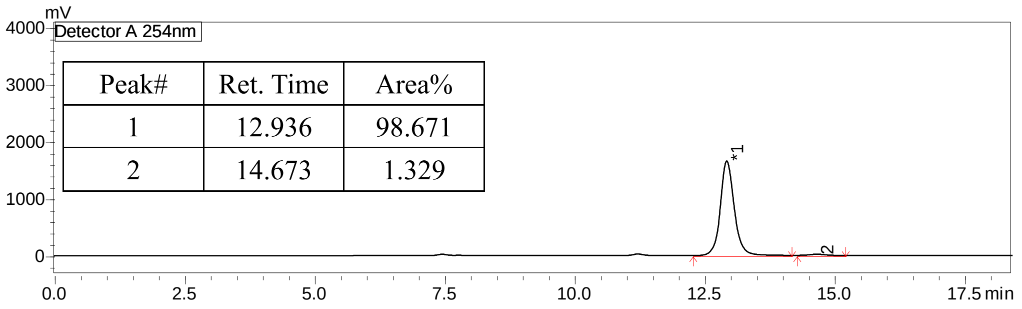

Peak#	Ret. Time	Area%
1	12.936	98.671
2	14.673	1.329

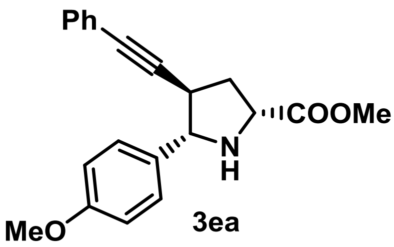

3ea

3ea: Procedure A, colorless oil, 63.8 mg, 95% yield, >20/1 dr (Diastereoselectivity was determined by ^{1}H NMR analysis of reaction crude mixture).

^{1}H NMR (400 MHz, $CDCl_3$) δ 7.51–7.43 (m, 2H), 7.38–7.32 (m, 2H), 7.28–7.22 (m, 3H), 6.93–6.87 (m, 2H), 4.16 (d, *J* = 9.2 Hz, 1H), 4.05 (dd, *J* = 9.3, 4.3 Hz, 1H), 3.79 (s, 3H), 3.78 (s, 3H), 2.89 (td, *J* = 9.7, 7.8 Hz, 1H), 2.53 (ddd, *J* = 12.4, 7.9, 4.3 Hz, 1H), 2.41 (dt, *J* = 13.0, 9.7 Hz, 1H) ppm.

^{13}C NMR (126 MHz, $CDCl_3$) δ 175.11, 159.17, 132.79, 131.56, 128.14, 127.96, 127.82, 123.33, 113.87, 89.02, 82.94, 69.02, 58.63, 55.21, 52.32, 38.90, 37.53 ppm.

HRMS (ESI) m/z [M+H]$^+$: calcd $C_{21}H_{22}NO_3^+$ 336.1594; found 336.1597.

IR (film): ν_{max} (cm^{-1}) 3349, 2952, 2836, 1734, 1611, 1490, 1247, 1032, 757, 692.

Optical rotation: $[\alpha]_D^{25}$ = 193.27 (*c* = 2.00, $CHCl_3$, 98% ee).

HPLC: DAICEL CHIRALPAK IE, hexane/*i*-PrOH = 43/7, flow rate: 0.5 mL/min, λ = 254 nm, t_R(minor) = 30.9 min, t_R(major) = 27.0 min, 98% ee.

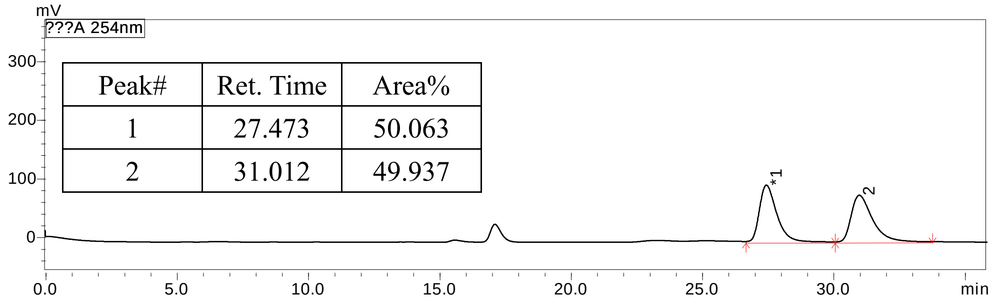

Peak#	Ret. Time	Area%
1	27.473	50.063
2	31.012	49.937

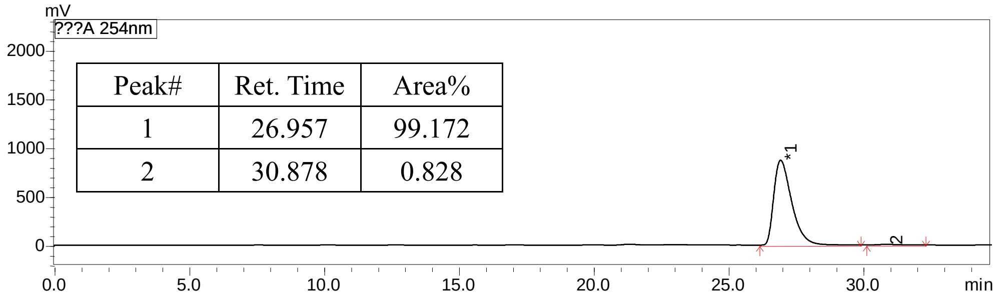

Peak#	Ret. Time	Area%
1	26.957	99.172
2	30.878	0.828

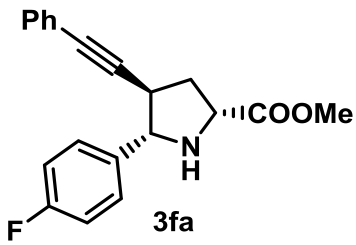

3fa

3fa: Procedure A, colorless oil, 62.3 mg, 96% yield, >20/1 dr (Diastereoselectivity was determined by ^{1}H NMR analysis of reaction crude mixture).

^{1}H NMR (400 MHz, $CDCl_3$) δ 7.59–7.48 (m, 2H), 7.38–7.32 (m, 2H), 7.31–7.24 (m, 3H), 7.10–7.00 (m, 2H), 4.20 (d, J = 9.1 Hz, 1H), 4.08 (dd, J = 9.3, 4.2 Hz, 1H), 3.80 (s, 3H), 2.89 (td, J = 9.8, 7.9 Hz, 1H), 2.54 (ddd, J = 12.4, 7.8, 4.2 Hz, 1H), 2.42 (dt, J = 13.1, 9.7 Hz, 1H) ppm.

^{13}C NMR (101 MHz, $CDCl_3$) δ 175.03, 162.35 (d, J = 245.5 Hz), 136.61 (d, J = 3.1 Hz), 131.53, 128.44 (d, J = 8.1 Hz), 128.18, 127.94, 123.14, 115.29 (d, J = 21.3 Hz), 88.57, 83.17, 68.69, 58.49, 52.34, 39.05, 37.30 ppm.

^{19}F NMR (376 MHz, $CDCl_3$) δ -114.93 ppm.

HRMS (ESI) m/z [M+H]$^+$: calcd for $C_{20}H_{19}FNO_2^+$ 324.1394; found 324.1396.

IR (film): ν_{max} (cm^{-1}) 3366, 2951, 1735, 1676, 1490, 1223, 757, 691.

Optical rotation: $[\alpha]_D^{25}$ = 156.35 (c = 2.00, $CHCl_3$, 98% ee).

HPLC: DAICEL CHIRALPAK IA-3, hexane/i-PrOH = 44/6, flow rate: 0.5 mL/min, λ = 254 nm, t_R(minor) = 15.1 min, t_R(major) = 14.0 min, 98% ee.

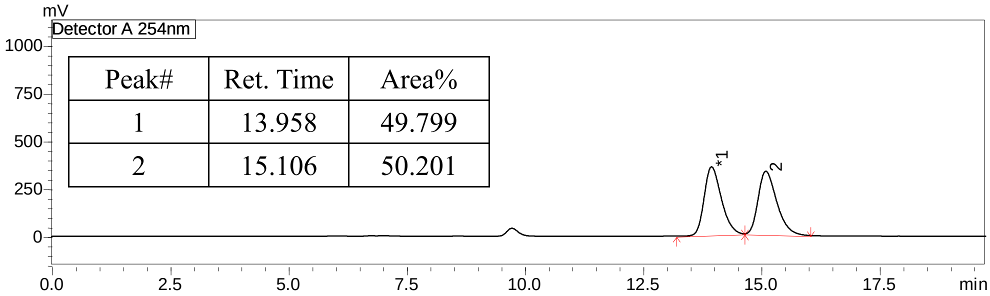

Peak#	Ret. Time	Area%
1	13.958	49.799
2	15.106	50.201

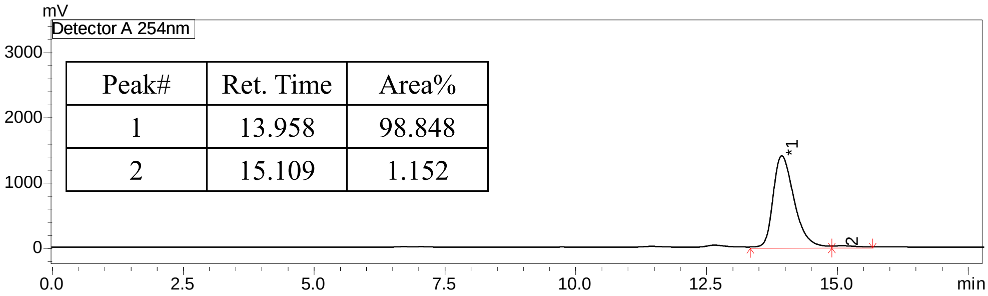

Peak#	Ret. Time	Area%
1	13.958	98.848
2	15.109	1.152

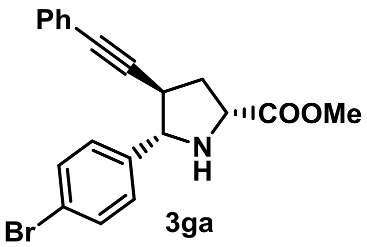

3ga

3ga: Procedure A, colorless oil, 76.4 mg, 99% yield, >20/1 dr (Diastereoselectivity was determined by ^{1}H NMR analysis of reaction crude mixture).

^{1}H NMR (400 MHz, $CDCl_3$) δ 7.53–7.43 (m, 4H), 7.39–7.32 (m, 2H), 7.32–7.25 (m, 3H), 4.18 (d, *J* = 9.1 Hz, 1H), 4.08 (dd, *J* = 9.3, 4.2 Hz, 1H), 3.80 (s, 3H), 2.88 (td, *J* = 9.7, 7.9 Hz, 1H), 2.53 (ddd, *J* = 12.3, 7.8, 4.3 Hz, 1H), 2.41 (dt, *J* = 13.0, 9.5 Hz, 1H) ppm.

^{13}C NMR (101 MHz, $CDCl_3$) δ 174.91, 140.04, 131.52, 128.56, 128.18, 127.96, 123.06, 121.52, 88.41, 83.26, 68.68, 58.47, 52.34, 39.01, 37.20 ppm.

HRMS (ESI) m/z [M+H]$^{+}$: calcd $C_{20}H_{19}BrNO_2^{+}$ 384.0594; found 384.0593.

IR (film): ν_{max} (cm^{-1}) 3358, 2950, 1735, 1489, 1435, 1010, 756, 691, 529.

Optical rotation: $[\alpha]_D^{25}$ = 199.33 (*c* = 2.00, $CHCl_3$, 95% ee).

HPLC: DAICEL CHIRALPAK IE, hexane/*i*-PrOH = 3/2, flow rate: 0.5 mL/min, λ = 254 nm, t_R(minor) = 11.9 min, t_R(major) = 10.9 min, 95% ee.

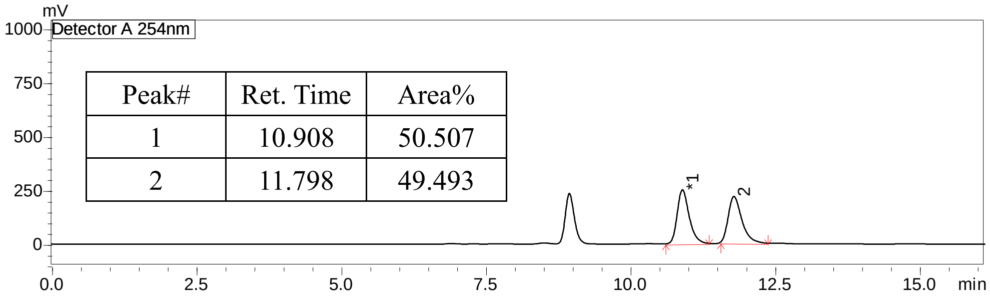

Peak#	Ret. Time	Area%
1	10.908	50.507
2	11.798	49.493

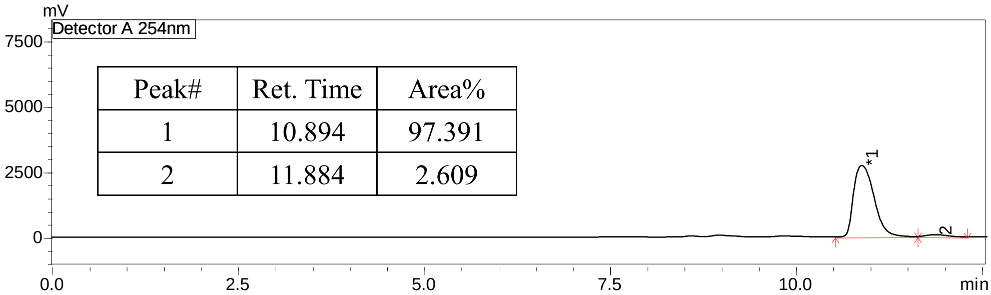

Peak#	Ret. Time	Area%
1	10.894	97.391
2	11.884	2.609

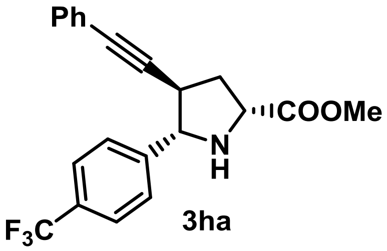

3ha

3ha: Procedure A, yellow oil, 67.7 mg, 91% yield, >20:1 dr (Diastereoselectivity was determined by ^{1}H NMR analysis of reaction crude mixture).

^{1}H NMR (400 MHz, $CDCl_3$) δ 7.71 (d, J = 8.1 Hz, 2H), 7.62 (d, J = 8.1 Hz, 2H), 7.40–7.32 (m, 2H), 7.31–7.25 (m, 3H), 4.30 (d, J = 9.0 Hz, 1H), 4.12 (dd, J = 9.3, 4.3 Hz, 1H), 3.81 (s, 3H), 2.93 (td, J = 9.6, 7.9 Hz, 1H), 2.56 (ddd, J = 12.4, 7.8, 4.3 Hz, 1H), 2.44 (dt, J = 13.0, 9.6 Hz, 1H) ppm.

^{13}C NMR (101 MHz, $CDCl_3$) δ 174.79, 145.26, 131.51, 129.84 (q, J = 32.6 Hz), 128.19, 128.02, 127.16, 126.90 (q, J = 272.0 Hz) 125.34 (q, J = 4.1 Hz), 123.00, 88.23, 83.41, 68.67, 58.48, 52.27, 39.07, 37.16 ppm.

^{19}F NMR (376 MHz, $CDCl_3$) δ -62.45 ppm.

HRMS (ESI) m/z [M+H]$^{+}$: calcd $C_{21}H_{19}F_3NO_2^{+}$ 374.1362; found 374.1362.

IR (film): ν_{max} (cm^{-1}) 3353, 2953, 1739, 1619, 1325, 1208, 1122, 757, 691.

Optical rotation: $[\alpha]_D^{25}$ = 161.61 (c = 2.50, $CHCl_3$, 98% ee).

HPLC: DAICEL CHIRALPAK IE, hexane/i-PrOH = 3/2, flow rate: 0.5 mL/min, λ = 254 nm, t_R(minor) = 9.0 min, t_R(major) = 8.5 min, 98% ee.

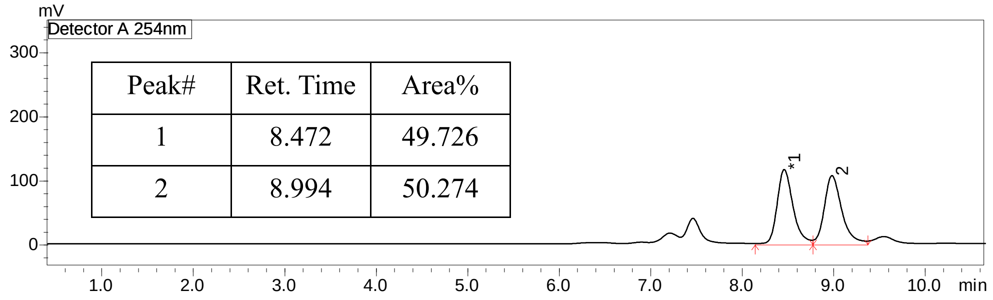

Peak#	Ret. Time	Area%
1	8.472	49.726
2	8.994	50.274

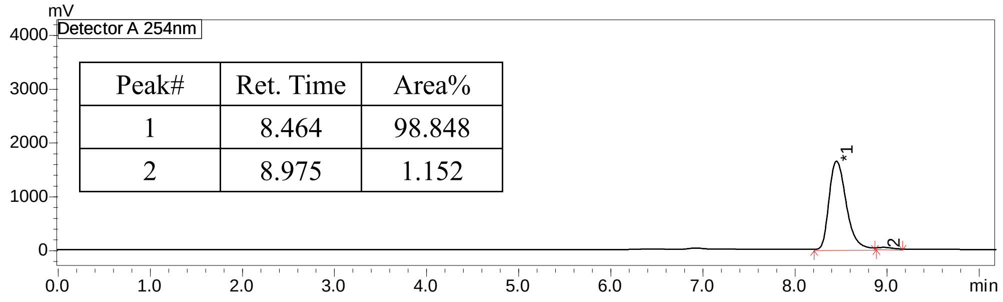

Peak#	Ret. Time	Area%
1	8.464	98.848
2	8.975	1.152

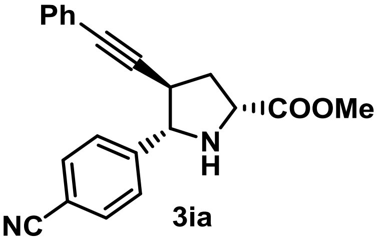

3ia

3ia: Procedure A, white solid, 47.3 mg, 72% yield, >20/1 dr (Diastereoselectivity was determined by ^{1}H NMR analysis of reaction crude mixture).

^{1}H NMR (500 MHz, $CDCl_3$) δ 7.72 (d, J = 8.3 Hz, 2H), 7.65 (d, J = 8.3 Hz, 2H), 7.37–7.33 (m, 2H), 7.30–7.27 (m, 3H), 4.30 (d, J = 9.0 Hz, 1H), 4.13 (dd, J = 9.2, 4.2 Hz, 1H), 3.80 (s, 3H), 2.95–2.86 (m, 1H), 2.55 (ddd, J = 13.0, 7.7, 4.1 Hz, 1H), 2.42 (dt, J = 13.0, 9.6 Hz, 1H) ppm.

^{13}C NMR (101 MHz, $CDCl_3$) δ 174.78, 146.95, 132.24, 131.50, 128.23, 128.12, 127.58, 122.84, 118.86, 111.40, 87.91, 83.59, 68.61, 58.40, 52.34, 39.20, 37.05 ppm.

HRMS (ESI) m/z [M+H]$^+$: calcd $C_{21}H_{19}N_2O_2^+$ 331.1441; found 331.1440.

IR (film): ν_{max} (cm^{-1}) 3366, 2915,2227, 1734, 1654, 1499, 1205, 757, 692.

Optical rotation: $[\alpha]_D^{25}$ = 248.60 (c = 2.00, $CHCl_3$, 97% ee).

HPLC: DAICEL CHIRALPAK IE, hexane/i-PrOH = 3/2, flow rate: 0.5 mL/min, λ = 254 nm, t_R(minor) = 17.5 min, t_R(major) = 15.5 min, 97% ee.

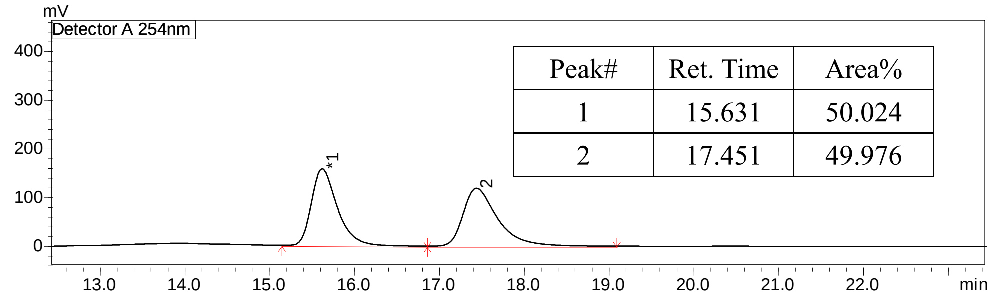

Peak#	Ret. Time	Area%
1	15.631	50.024
2	17.451	49.976

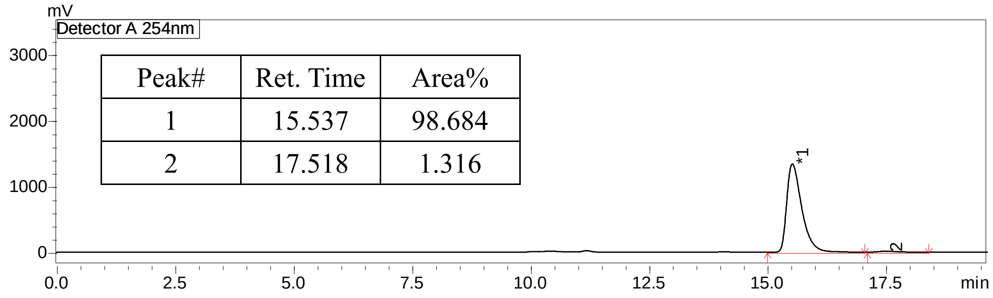

Peak#	Ret. Time	Area%
1	15.537	98.684
2	17.518	1.316

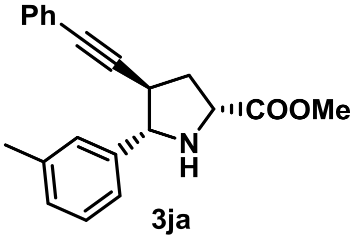

3ja

3ja: Procedure A, colorless oil, 63.8 mg, 99% yield, >20/1 dr (Diastereoselectivity was determined by ^{1}H NMR analysis of reaction crude mixture).

^{1}H NMR (500 MHz, $CDCl_3$) δ 7.39–7.33 (m, 4H), 7.30–7.23 (m, 4H), 7.12 (d, *J* = 7.5 Hz, 1H), 4.18 (d, *J* = 9.1 Hz, 1H), 4.08 (dd, *J* = 9.2, 4.5 Hz, 1H), 3.80 (s, 3H), 2.93 (td, *J* = 9.5, 8.0 Hz, 1H), 2.53 (ddd, *J* = 12.7, 8.0, 4.6 Hz, 1H), 2.48–2.40 (m, 1H), 2.38 (s, 3H) ppm.

^{13}C NMR (101 MHz, $CDCl_3$) δ 174.99, 140.60, 138.09, 131.54, 128.53, 128.38, 128.14, 127.82, 127.47, 123.91, 123.32, 89.06, 82.97, 69.44, 58.69, 52.33, 38.79, 37.55, 21.48 ppm.

HRMS (ESI) m/z [M+H]$^{+}$: calcd $C_{21}H_{22}NO_2^{+}$ 320.1645; found 320.1645.

IR (film): ν_{max} (cm^{-1}) 3356, 2950, 2922,1734,1654, 1458, 1207, 1070, 756, 691.

Optical rotation: $[\alpha]_D^{25}$ = 164.32 (*c* = 2.00, $CHCl_3$, 95% ee).

HPLC: DAICEL CHIRALPAK IA-3, hexane/*i*-PrOH = 4/1, flow rate: 0.5 mL/min, λ = 254 nm, t_R(minor) = 12.7 min, t_R(major) = 11.0 min, 95% ee.

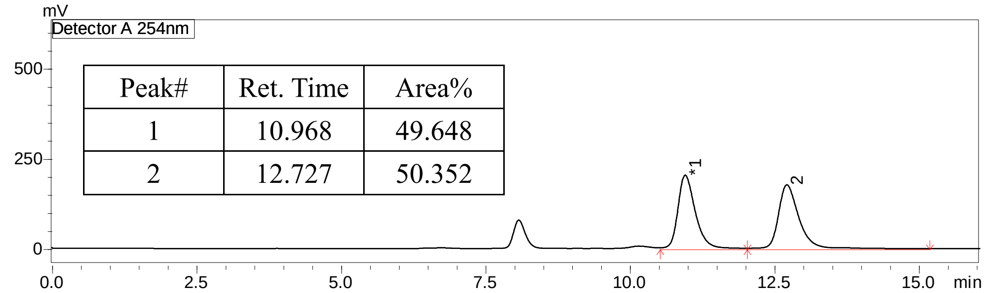

Peak#	Ret. Time	Area%
1	10.968	49.648
2	12.727	50.352

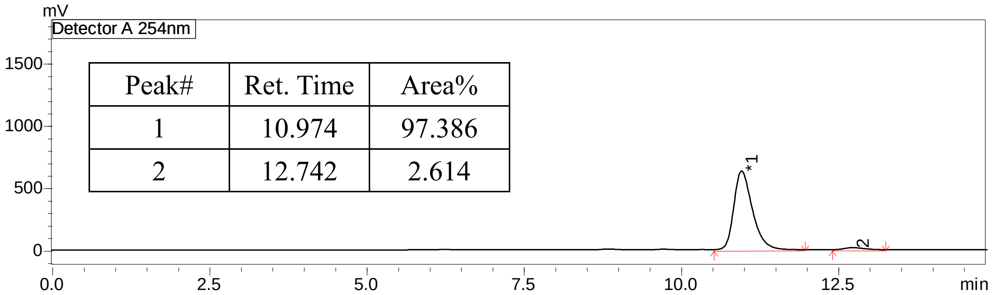

Peak#	Ret. Time	Area%
1	10.974	97.386
2	12.742	2.614

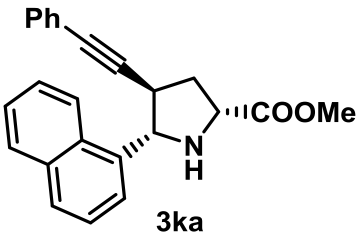

3ka

3ka: Procedure A, colorless oil, 68.2 mg, 96% yield, >20/1 dr (Diastereoselectivity was determined by ^{1}H NMR analysis of reaction crude mixture).

^{1}H NMR (400 MHz, $CDCl_3$) δ 8.44–8.32 (m, 1H), 7.94–7.84 (m, 2H), 7.80 (d, J = 8.2 Hz, 1H), 7.58–7.45 (m, 3H), 7.38–7.29 (m, 2H), 7.28–7.23 (m, 3H), 5.13 (d, J = 7.2 Hz, 1H), 4.28 (t, J = 7.4 Hz, 1H), 3.81 (s, 3H), 3.25 (q, J = 7.4 Hz, 1H), 2.55–2.42 (m, 2H) ppm.

^{13}C NMR (126 MHz, $CDCl_3$) δ 174.92, 137.17, 133.75, 131.76, 131.49, 128.58, 128.11, 128.03, 127.84, 125.90, 125.51, 123.92, 123.48, 123.19, 89.62, 83.04 , 64.88, 58.96, 52.24, 37.74, 36.79 ppm.

HRMS (ESI) m/z [M+H]$^{+}$: calcd $C_{24}H_{22}NO_2^{+}$ 356.1645; found 356.1646.

IR (film): ν_{max} (cm^{-1}) 3375, 3051, 2950, 1737, 1597, 1489, 1270, 1113, 800, 779, 757, 691.

Optical rotation: $[\alpha]_D^{25}$ = 33.80 (c = 2.22, $CHCl_3$, 94% ee).

HPLC: DAICEL CHIRALPAK IA, hexane/i-PrOH = 44/6, flow rate: 0.5 mL/min, λ = 254 nm, t_R(minor) = 29.4 min, t_R(major) = 13.0 min, 94% ee.

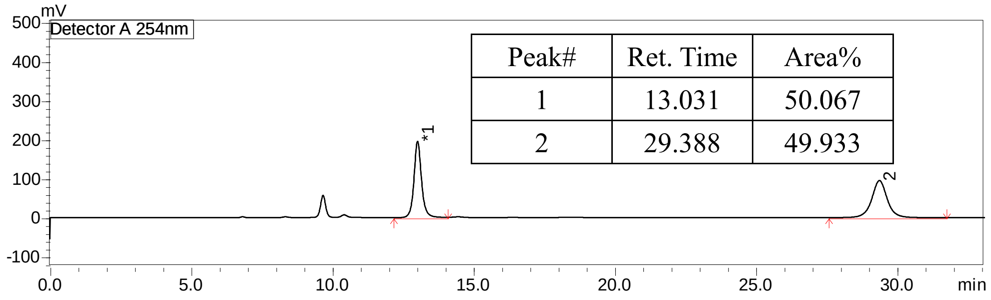

Peak#	Ret. Time	Area%
1	13.031	50.067
2	29.388	49.933

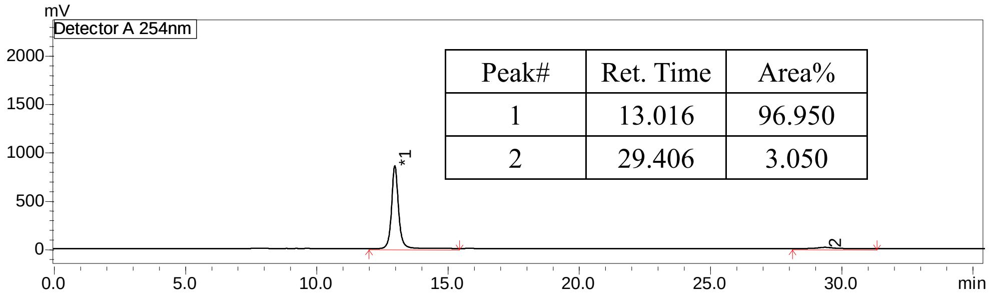

Peak#	Ret. Time	Area%
1	13.016	96.950
2	29.406	3.050

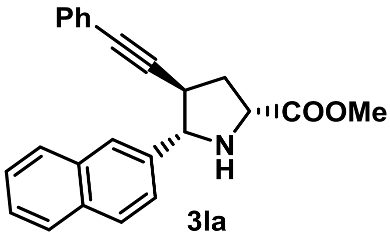

3la

3la: Procedure A, colorless oil, 67.1 mg, 95% yield, >20/1 dr (Diastereoselectivity was determined by ^{1}H NMR analysis of reaction crude mixture).

^{1}H NMR (400 MHz, $CDCl_3$) δ 7.99 (d, *J* = 1.8 Hz, 1H), 7.85 (td, *J* = 9.3, 8.7, 3.7 Hz, 3H), 7.71 (dd, *J* = 8.5, 1.8 Hz, 1H), 7.52–7.41 (m, 2H), 7.35 (dd, *J* = 6.7, 3.1 Hz, 2H), 7.30–7.23 (m, 3H), 4.40 (d, *J* = 9.1 Hz, 1H), 4.15 (dd, *J* = 9.2, 4.5 Hz, 1H), 3.83 (s, 3H), 3.05 (td, *J* = 9.5, 8.0 Hz, 1H), 2.59 (ddd, *J* = 12.6, 7.9, 4.5 Hz, 1H), 2.48 (dt, *J* = 13.0, 9.6 Hz, 2H) ppm.

^{13}C NMR (126 MHz, $CDCl_3$) δ 174.95, 138.11, 133.28, 133.11, 131.54, 128.29, 128.13, 127.95, 127.85, 127.59, 126.02, 125.80, 125.79, 124.80, 123.22, 88.86, 83.14, 69.45, 58.66, 52.35, 38.87, 37.46 ppm.

HRMS (ESI) m/z [M+H]$^{+}$: calcd $C_{24}H_{22}NO_2^{+}$ 356.1645; found 356.1645.

IR (film): ν_{max} (cm^{-1}) 3348, 3054, 2950, 1734, 1599, 1490, 1206, 756, 691.

Optical rotation: $[\alpha]_D^{25}$ = 224.98 (*c* = 2.000, $CHCl_3$, 94% ee).

HPLC: DAICEL CHIRALPAK IBN-3, hexane/*i*-PrOH = 45/5, flow rate: 0.5 mL/min, λ = 254 nm, t_R(minor) = 27.4 min, t_R(major) = 31.0 min, 94% ee.

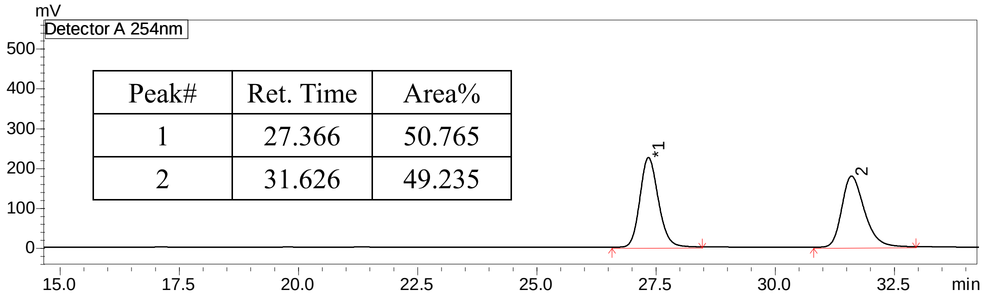

Peak#	Ret. Time	Area%
1	27.366	50.765
2	31.626	49.235

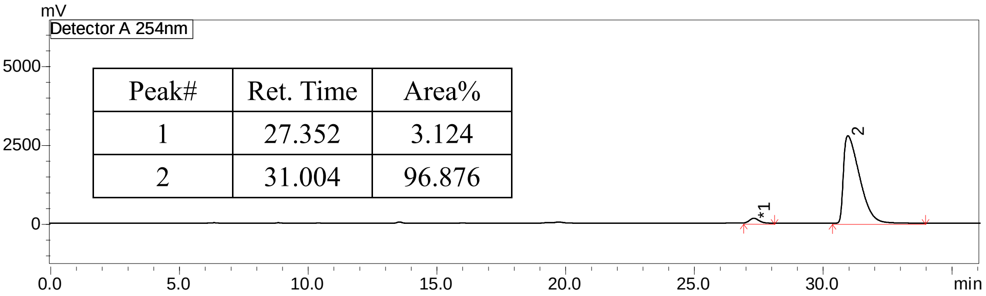

Peak#	Ret. Time	Area%
1	27.352	3.124
2	31.004	96.876

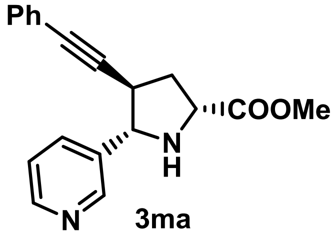

3ma

3ma: Procedure A, yellow oil, 60.5 mg, 98% yield, >20/1 dr (Diastereoselectivity was determined by ^{1}H NMR analysis of reaction crude mixture).

^{1}H NMR (500 MHz, $CDCl_3$) δ 8.77 (d, *J* = 2.3 Hz, 1H), 8.55 (dd, *J* = 4.8, 1.7 Hz, 1H), 7.97 (dt, *J* = 8.0, 2.0 Hz, 1H), 7.41–7.36 (m, 2H), 7.36–7.32 (m, 1H), 7.32–7.28 (m, 4H), 4.29 (d, *J* = 9.2 Hz, 1H), 4.14 (dd, *J* = 9.4, 4.1 Hz, 1H), 3.80 (s, 3H), 2.98 (td, *J* = 9.7, 7.8 Hz, 1H), 2.59 (ddd, *J* = 13.1, 7.7, 4.1 Hz, 1H), 2.44 (dt, *J* = 13.1, 9.7 Hz, 1H) ppm.

^{13}C NMR (101 MHz, $CDCl_3$) δ 174.78, 149.10, 148.75, 136.58, 134.28, 131.52, 128.17, 128.02, 123.55, 122.88, 87.88, 83.50, 66.82, 58.45, 52.33, 38.95, 37.11 ppm.

HRMS (ESI) m/z [M+H]$^{+}$: calcd $C_{19}H_{19}N2O_2^{+}$ 307.1441; found 307.1441.

IR (film): ν_{max} (cm^{-1}) 3366, 2915, 1734, 1636,1507, 1206, 757, 714, 692.

Optical rotation: $[\alpha]_D^{25}$ = 147.85 (*c* = 2.00, $CHCl_3$, 97% ee).

HPLC: DAICEL CHIRALPAK IE, hexane/*i*-PrOH = 3/2, flow rate: 0.5 mL/min, λ = 254 nm, t_R(minor) = 23.6 min, t_R(major) = 25.6 min, 97% ee.

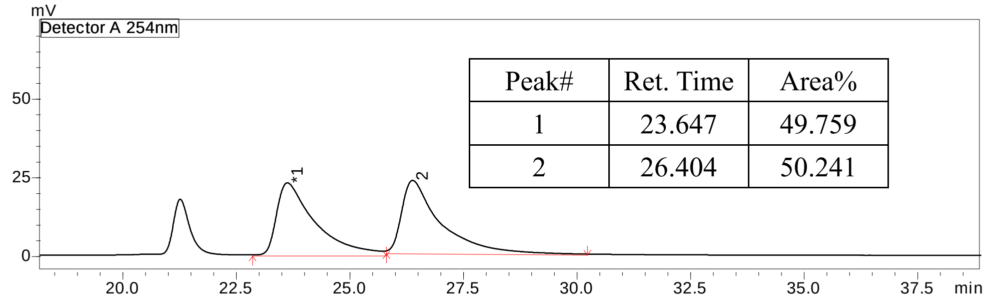

Peak#	Ret. Time	Area%
1	23.647	49.759
2	26.404	50.241

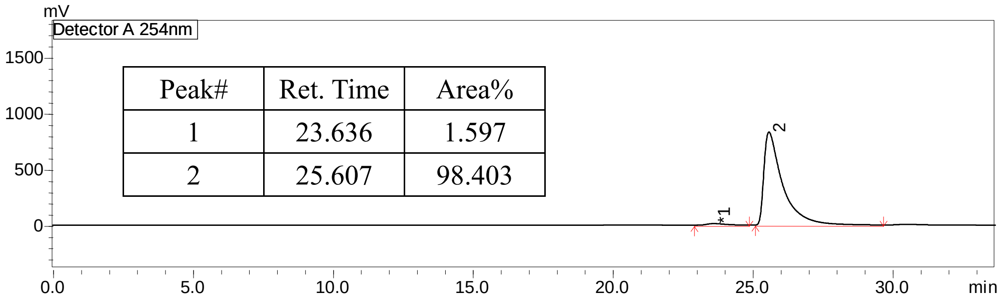

Peak#	Ret. Time	Area%
1	23.636	1.597
2	25.607	98.403

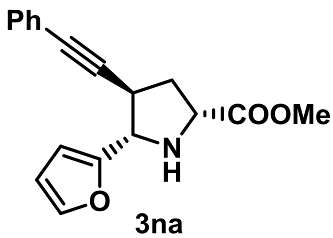

3na

3na: Procedure A, yellow oil, 55.2 mg, 94% yield, >20/1 dr (Diastereoselectivity was determined by ^{1}H NMR analysis of reaction crude mixture).

^{1}H NMR (500 MHz, $CDCl_3$) δ 7.43–7.42 (m, 1H), 7.42–7.36 (m, 2H), 7.31–7.27 (m, 3H), 6.38 (d, *J* = 3.2 Hz, 1H), 6.34 (dd, *J* = 3.3, 1.8 Hz, 1H), 4.38 (d, *J* = 8.0 Hz, 1H), 4.07 (dd, *J* = 9.0, 5.5 Hz, 1H), 3.78 (s, 3H), 3.20 (q, *J* = 8.2 Hz, 1H), 2.51 (ddd, *J* = 13.4, 8.1, 5.5 Hz, 1H), 2.40 (dt, *J* = 13.0, 8.8 Hz, 1H) ppm.

^{13}C NMR (101 MHz, $CDCl_3$) δ 174.47, 153.22, 142.20, 131.59, 128.14, 127.92, 123.12, 110.22, 107.11, 88.74, 82.66, 63.14, 58.92, 52.38, 37.26, 36.25 ppm.

HRMS (ESI) m/z [M+H]$^{+}$: calcd $C_{18}H_{18}NO_3^{+}$ 296.1281; found 296.1282.

IR (film): ν_{max} (cm^{-1}) 3341, 2951, 1736, 1597, 1490, 1208, 756, 692.

Optical rotation: $[\alpha]_D^{25}$ = 162.41 (*c* = 2.00, $CHCl_3$, 97% ee).

HPLC: DAICEL CHIRALPAK IE, hexane/*i*-PrOH = 3/2, flow rate: 0.5 mL/min, λ = 254 nm, t_R(minor) = 13.9 min, t_R(major) = 13.0 min, 97% ee.

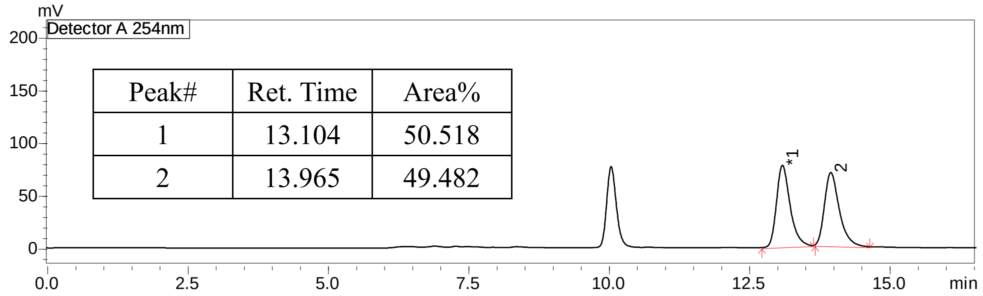

Peak#	Ret. Time	Area%
1	13.104	50.518
2	13.965	49.482

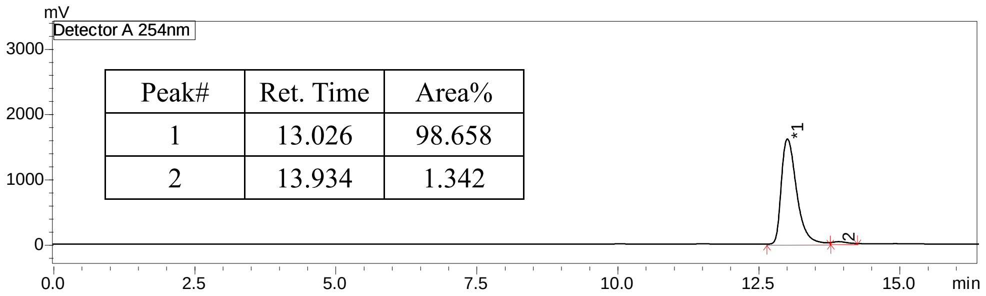

Peak#	Ret. Time	Area%
1	13.026	98.658
2	13.934	1.342

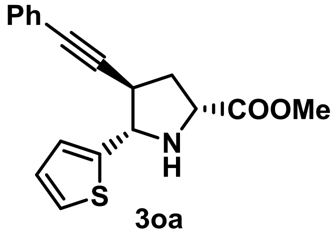

3oa

3oa: Procedure A, colorless oil, 59.0 mg, 95% yield, >20/1 dr (Diastereoselectivity was determined by ^{1}H NMR analysis of reaction crude mixture).

^{1}H NMR (400 MHz, $CDCl_3$) δ 7.42–7.36 (m, 2H), 7.35–7.22 (m, 4H), 7.18 (dt, *J* = 3.6, 1.1 Hz, 1H), 6.99 (dd, *J* = 5.1, 3.5 Hz, 1H), 4.48 (dd, *J* = 8.7, 0.9 Hz, 1H), 4.08 (dd, *J* = 9.2, 4.8 Hz, 1H), 3.79 (s, 3H), 3.02 (dt, *J* = 9.7, 8.3 Hz, 1H), 2.54 (ddd, *J* = 12.8, 8.0, 4.8 Hz, 1H), 2.42 (dt, *J* = 13.1, 9.4 Hz, 1H) ppm.

^{13}C NMR (101 MHz, $CDCl_3$) δ 174.33, 144.65, 131.50, 128.11, 127.89, 126.70, 124.33, 124.23, 123.08, 88.52, 83.22, 64.96, 58.57, 52.26, 39.47, 37.18 ppm.

HRMS (ESI) m/z [M+H]$^{+}$: calcd $C_{18}H_{22}NO_2S^{+}$ 312.1053; found 312.1053.

IR (film): ν_{max} (cm^{-1}) 3353, 2951, 1734, 1646, 1506, 1208, 757, 692.

Optical rotation: $[\alpha]_D^{25}$ = 146.18 (*c* = 2.000, $CHCl_3$, 91% ee).

HPLC: DAICEL CHIRALPAK IE, hexane/*i*-PrOH = 43/7, flow rate: 0.5 mL/min, λ = 254 nm, t_R(minor) = 20.8 min, t_R(major) = 17.7 min, 91% ee.

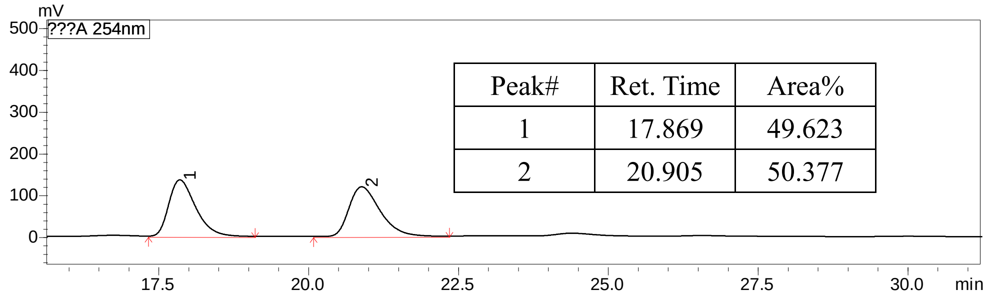

Peak#	Ret. Time	Area%
1	17.869	49.623
2	20.905	50.377

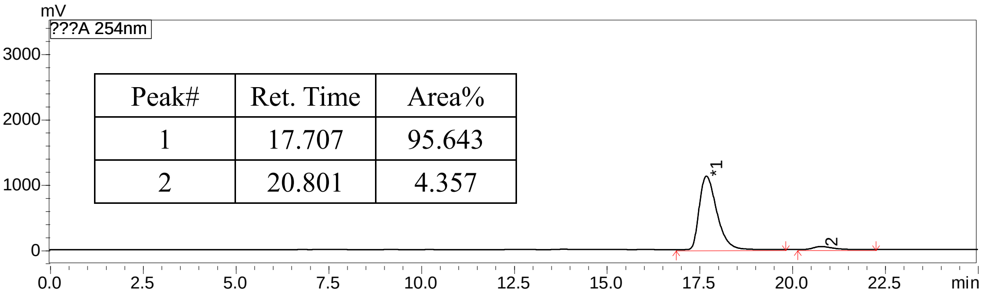

Peak#	Ret. Time	Area%
1	17.707	95.643
2	20.801	4.357

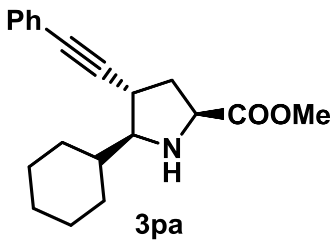

3pa

3pa: Procedure C, colorless oil, 44.6 mg, 72% yield, 9/1 dr (Diastereoselectivity was determined by ^{1}H NMR analysis of reaction crude mixture).

^{1}H NMR (400 MHz, $CDCl_3$) δ 7.40–7.34 (m, 2H), 7.30–7.25 (m, 3H), 3.96–3.84 (m, 1H), 3.75 (s, 3H), 2.97 (t, J = 7.8 Hz, 1H), 2.70 (q, J = 8.5 Hz, 1H), 2.30 (t, J = 7.6 Hz, 2H), 1.96 (d, J = 10.7 Hz, 1H), 1.88 (d, J = 11.8 Hz, 1H), 1.81–1.73 (m, 2H), 1.70–1.63 (m, 1H), 1.59–1.48 (m, 1H), 1.33–1.15 (m, 6H) ppm.

^{13}C NMR (126 MHz, $CDCl_3$) δ 177.65, 130.71, 128.20, 127.76, 123.57, 90.31, 83.71, 70.96, 59.97, 54.22, 42.21, 36.87, 32.87, 30.89, 29.86, 26.38, 26.23, 26.06 ppm.

HRMS (ESI) m/z [M+H]$^{+}$: calcd $C_{20}H_{26}NO_2^{+}$ 312.1958; found 312.1961.

IR (film): ν_{max} (cm^{-1}) 3346, 2924, 2852, 1738, 1598, 1490, 1443, 1205, 1003, 755, 691 .

Optical rotation: $[\alpha]_D^{25}$ = -13.23 (c = 2.22, $CHCl_3$, 82% ee).

HPLC: DAICEL CHIRALPAK IE, hexane/i-PrOH = 47/3, flow rate: 0.5 mL/min, λ = 254 nm, t_R(minor) = 17.9 min, t_R(major) = 19.8 min, 82% ee.

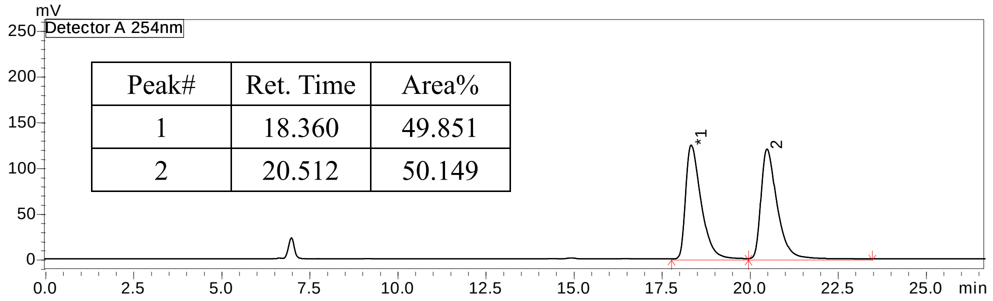

Peak#	Ret. Time	Area%
1	18.360	49.851
2	20.512	50.149

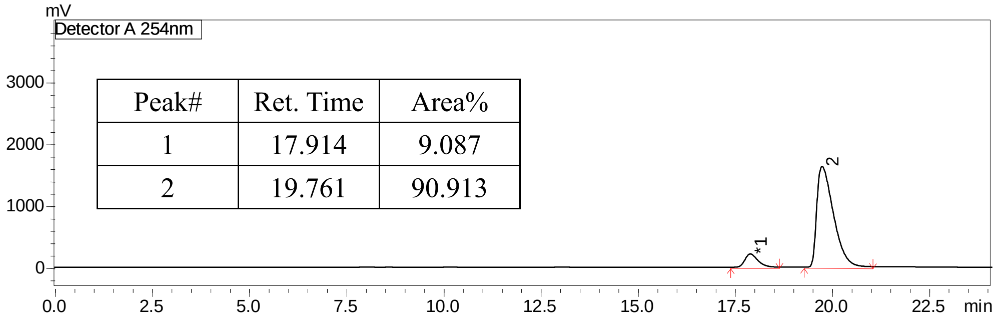

Peak#	Ret. Time	Area%
1	17.914	9.087
2	19.761	90.913

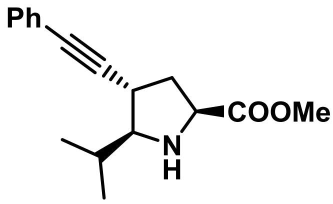

3qa

3qa: Procedure C, colorless oil, 39.5 mg, 73% yield, 10/1 dr (Diastereoselectivity was determined by ^{1}H NMR analysis of reaction crude mixture).

^{1}H NMR (400 MHz, $CDCl_3$) δ 7.40–7.34 (m, 2H), 7.31–7.25 (m, 3H), 3.91 (dd, J = 8.1, 6.7 Hz, 1H), 3.75 (s, 3H), 2.95 (dd, J = 8.6, 6.5 Hz, 1H), 2.68 (q, J = 8.5 Hz, 1H), 2.37–2.27 (m, 2H), 1.86 (hept, J = 6.7 Hz, 1H), 1.12 (d, J = 6.8 Hz, 3H), 1.06 (d, J = 6.7 Hz, 3H) ppm.

^{13}C NMR (126 MHz, $CDCl_3$) δ 174.80, 131.44, 128.18, 127.74, 123.55, 90.91, 82.14, 71.88, 58.79, 52.23, 38.21, 33.80, 32.16, 20.10, 19.32 ppm.

HRMS (ESI) m/z [M+H]$^+$: calcd $C_{17}H_{22}NO_2^+$ 272.1645; found 272.1645.

IR (film): ν_{max} (cm^{-1}) 3347, 2957, 1734, 1597, 1489, 1210, 1023, 757, 692, .

Optical rotation: $[\alpha]_D^{25}$ = 22.27 (c = 1.59, $CHCl_3$, 80% ee).

HPLC: DAICEL CHIRALPAK IA, hexane/i-PrOH = 48/2, flow rate: 0.5 mL/min, λ = 254 nm, t_R(minor) = 11.9 min, t_R(major) = 13.4 min, 80% ee.

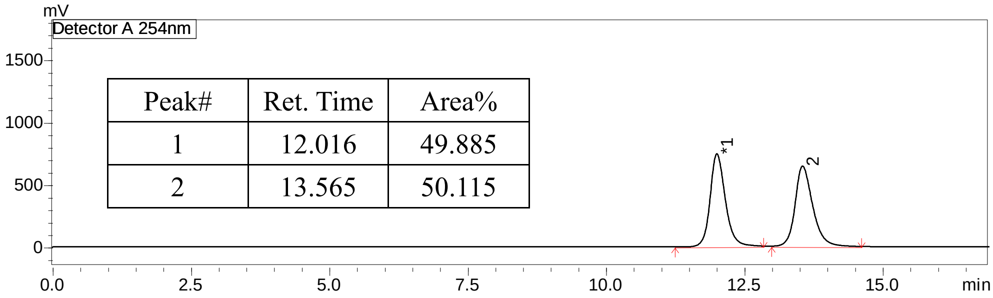

Peak#	Ret. Time	Area%
1	12.016	49.885
2	13.565	50.115

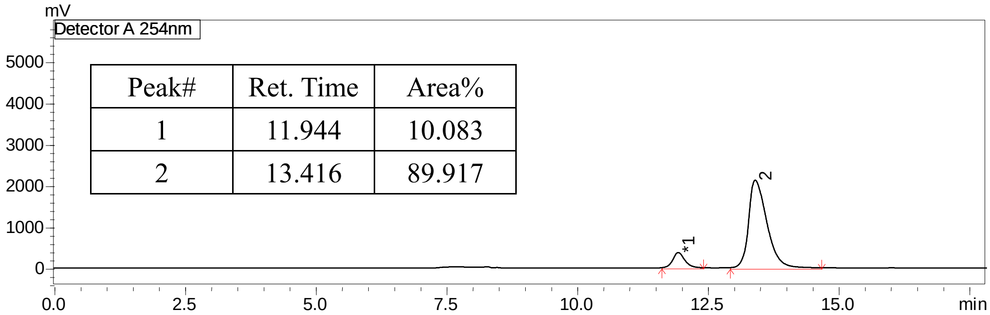

Peak#	Ret. Time	Area%
1	11.944	10.083
2	13.416	89.917

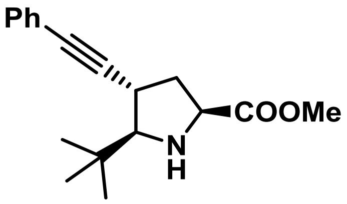

3ra

3ra: Procedure C, colorless oil, 39.4 mg, 69% yield, 10/1 dr (Diastereoselectivity was determined by ^{1}H NMR analysis of reaction crude mixture).

^{1}H NMR (400 MHz, $CDCl_3$) δ 7.39–7.34 (m, 2H), 7.30–7.25 (m, 3H), 3.91 (t, J = 7.5 Hz, 1H), 3.74 (s, 3H), 3.05 (d, J = 7.9 Hz, 1H), 2.80 (q, J = 7.7 Hz, 1H), 2.35–2.17 (m, 2H), 1.05 (s, 9H) ppm.

^{13}C NMR (126 MHz, $CDCl_3$) δ 174.45, 131.33, 128.16, 127.66, 123.62, 92.21, 81.94, 75.03, 58.82, 52.09, 38.62, 33.45, 31.19, 26.76 ppm.

HRMS (ESI) m/z [M+H]$^+$: calcd $C_{18}H_{24}NO_2^+$ 286.1802; found 286.1802.

IR (film): ν_{max} (cm^{-1}) 3372, 3053, 2952, 2225, 1738, 1597, 1504, 1200, 756, 691.

Optical rotation: $[\alpha]_D^{25}$ = 38.08 (*c* = 1.83, $CHCl_3$, 90% ee).

HPLC: DAICEL CHIRALPAK IC-3, hexane/*i*-PrOH = 43/7, flow rate: 0.5 mL/min, λ = 254 nm, t_R(minor) = 9.5 min, t_R(major) = 9.9 min, 90% ee.

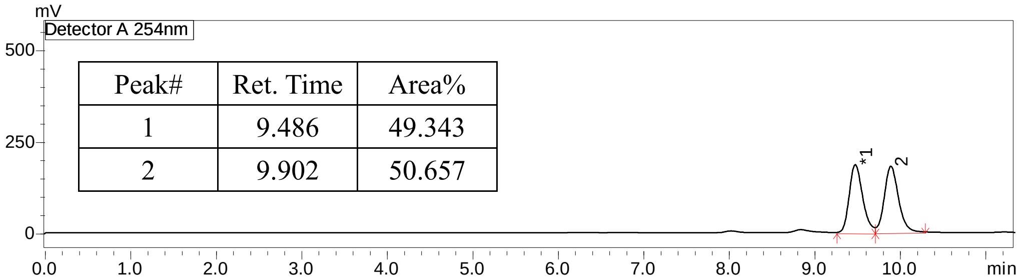

Peak#	Ret. Time	Area%
1	9.486	49.343
2	9.902	50.657

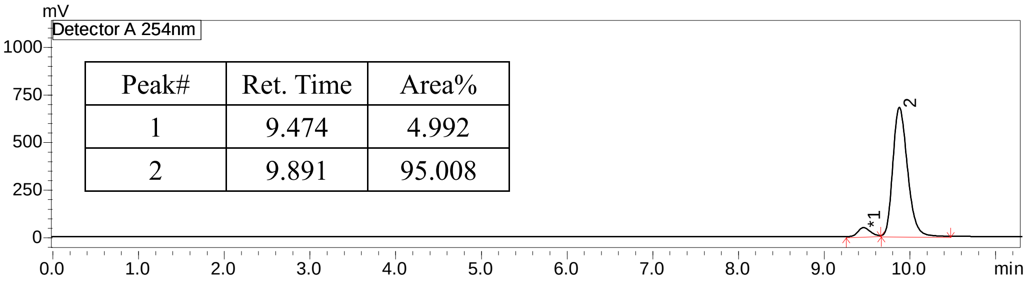

Peak#	Ret. Time	Area%
1	9.474	4.992
2	9.891	95.008

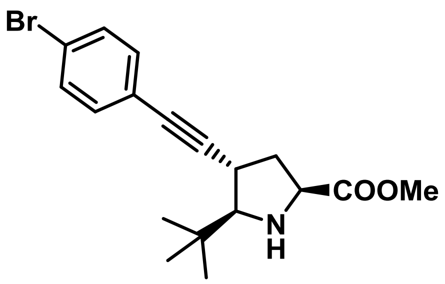

3ri

3ri: Procedure C, colorless oil, 52.8 mg, 73% yield, 12/1 dr (Diastereoselectivity was determined by ^{1}H NMR analysis of reaction crude mixture).

^{1}H NMR (400 MHz, $CDCl_3$) δ 7.41 (d, *J* = 8.4 Hz, 2H), 7.22 (d, *J* = 8.4 Hz, 2H), 3.90 (t, *J* = 7.5 Hz, 1H), 3.74 (s, 3H), 3.04 (d, *J* = 7.9 Hz, 1H), 2.78 (q, *J* = 7.9 Hz, 1H), 2.34–2.15 (m, 2H), 1.04 (s, 9H) ppm.

^{13}C NMR (126 MHz, $CDCl_3$) δ 174.38, 132.85, 131.42, 122.61, 121.81, 93.48, 81.02, 74.98, 58.84, 52.16, 38.50, 33.49, 31.23, 26.77 ppm.

HRMS (ESI) m/z [M+H]$^+$: calcd $C_{18}H_{23}BrNO_2^+$ 364.0907; found 364.0907.

IR (film): ν_{max} (cm^{-1}) 3357, 2951, 2865, 2225, 1738, 1504, 1434, 1070, 822, 524.

Optical rotation: $[\alpha]_D^{25}$ = 41.93 (*c* = 0.36, $CHCl_3$, 89% ee).

HPLC: DAICEL CHIRALPAK OX-3, hexane/*i*-PrOH = 49/1, flow rate: 0.5 mL/min, λ = 254 nm, t_R(minor) = 15.1 min, t_R(major) = 13.9 min, 89% ee.

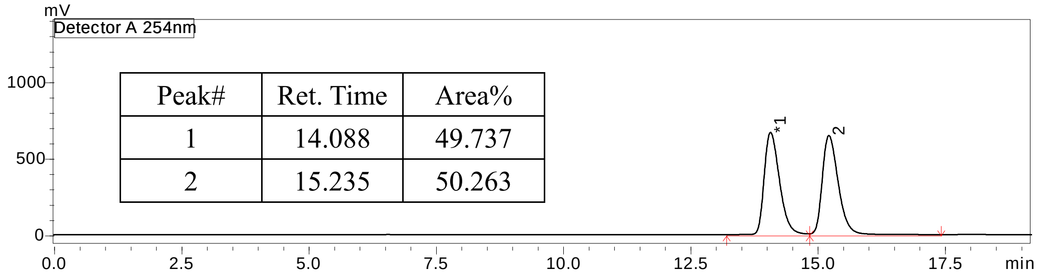

Peak#	Ret. Time	Area%
1	14.088	49.737
2	15.235	50.263

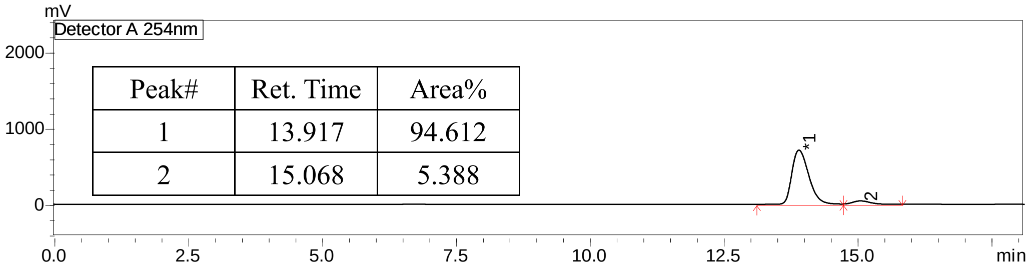

Peak#	Ret. Time	Area%
1	13.917	94.612
2	15.068	5.388

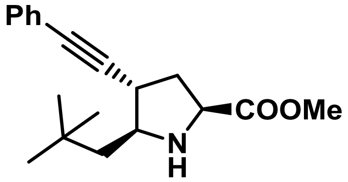

3sa

3sa: Procedure C, colorless oil, 34.3 mg, 57% yield, 9/1 dr (Diastereoselectivity was determined by ^{1}H NMR analysis of reaction crude mixture).

^{1}H NMR (400 MHz, $CDCl_3$) δ 7.41–7.35 (m, 2H), 7.31–7.24 (m, 3H), 3.92 (dd, J = 9.1, 4.9 Hz, 1H), 3.75 (s, 3H), 3.09 (td, J = 9.0, 2.1 Hz, 1H), 2.52–2.37 (m, 2H), 2.34–2.19 (m, 2H), 1.86 (dd, J = 14.3, 2.1 Hz, 1H), 1.39 (dd, J = 14.3, 8.7 Hz, 1H), 1.01 (s, 9H) ppm.

^{13}C NMR (126 MHz, $CDCl_3$) δ 175.14, 131.55, 128.19, 127.79, 123.44, 89.60, 82.26, 63.68, 59.31, 52.32, 48.76, 38.16, 36.84, 30.43, 30.00 ppm.

HRMS (ESI) m/z [M+H]$^+$: calcd $C_{19}H_{26}NO_2^+$ 300.1958; found 300.1960.

IR (film): ν_{max} (cm^{-1}) 3296, 3708, 2951, 2224, 1737, 1598, 1435, 1217, 756, 691.

Optical rotation: $[\alpha]_D^{25}$ = -33.89 (c = 0.81, $CHCl_3$, 79% ee).

HPLC: DAICEL CHIRALPAK IA-3, hexane/i-PrOH = 49/1, flow rate: 0.5 mL/min, λ = 254 nm, t_R(minor) = 10.7 min, t_R(major) = 11.4 min, 79% ee.

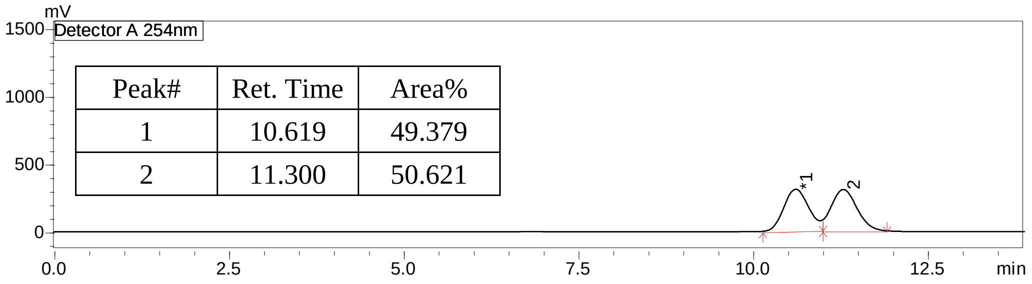

Peak#	Ret. Time	Area%
1	10.619	49.379
2	11.300	50.621

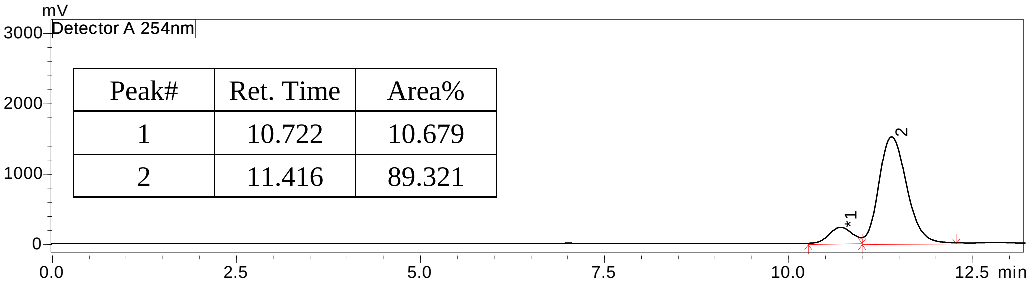

Peak#	Ret. Time	Area%
1	10.722	10.679
2	11.416	89.321

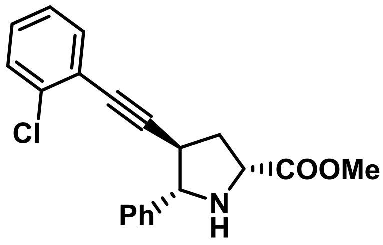

3ab

3ab: Procedure A, colorless oil, 66.9 mg, 98% yield, >20/1 dr (Diastereoselectivity was determined by ^{1}H NMR analysis of reaction crude mixture).

^{1}H NMR (400 MHz, $CDCl_3$) δ 7.63–7.55 (m, 2H), 7.41–7.33 (m, 4H), 7.33–7.27 (m, 1H), 7.23–7.13 (m, 2H), 4.28 (d, *J* = 9.0 Hz, 1H), 4.11 (dd, *J* = 9.2, 4.6 Hz, 1H), 3.80 (s, 3H), 2.99 (q, *J* = 9.2 Hz, 1H), 2.57 (ddd, *J* = 12.7, 8.0, 4.6 Hz, 1H), 2.47 (dt, *J* = 13.1, 9.4 Hz, 1H) ppm.

^{13}C NMR (126 MHz, $CDCl_3$) δ 174.90, 140.73, 135.84, 133.18, 129.08, 128.85, 128.45, 127.74, 126.85, 126.26, 123.11, 94.64, 79.87, 69.35, 58.73, 52.29, 39.15, 37.40 ppm.

HRMS (ESI) m/z [M+H]$^{+}$: calcd $C_{20}H_{19}ClNO_2^{+}$ 340.1099; found 340.1101.

IR (film): ν_{max} (cm^{-1}) 3347, 3029, 2951, 1735, 1473, 1436, 1207, 1033, 753, 700, 678.

Optical rotation: $[\alpha]_D^{25}$ = 154.48 (*c* = 2.00, $CHCl_3$, 98% ee).

HPLC: DAICEL CHIRALPAK IA, hexane/*i*-PrOH = 44/6, flow rate: 0.5 mL/min, λ = 254 nm, t_R(minor) = 15.0 min, t_R(major) = 13.5 min, 98% ee.

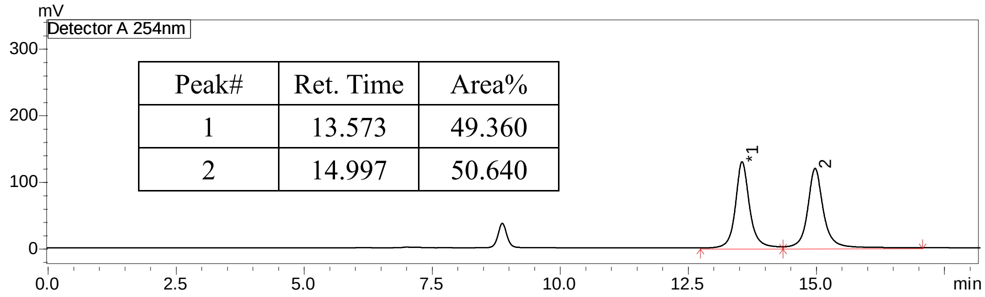

Peak#	Ret. Time	Area%
1	13.573	49.360
2	14.997	50.640

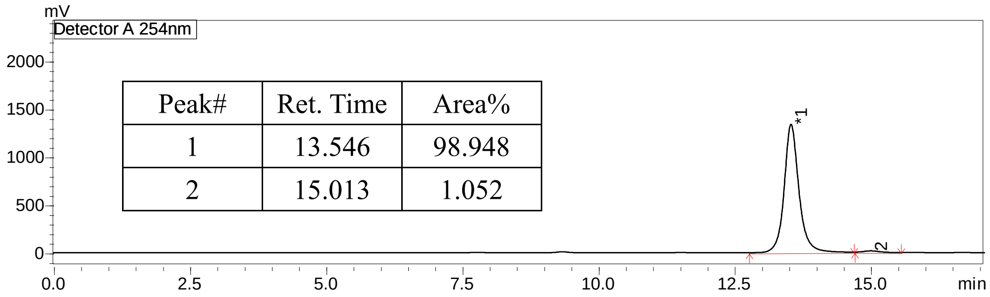

Peak#	Ret. Time	Area%
1	13.546	98.948
2	15.013	1.052

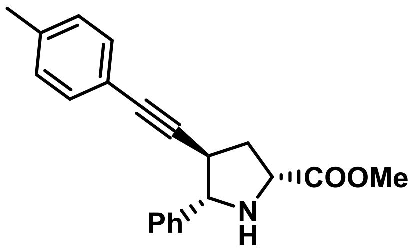

3ac

3ac: Procedure A, white solid, 63.1 mg, 99% yield, >20/1 dr (Diastereoselectivity was determined by ^{1}H NMR analysis of reaction crude mixture).

^{1}H NMR (400 MHz, $CDCl_3$) δ 7.56 (d, J = 7.4 Hz, 2H), 7.37 (t, J = 7.3 Hz, 2H), 7.30 (d, J = 7.1 Hz, 1H), 7.26 (d, J = 7.7 Hz, 2H), 7.07 (d, J = 7.7 Hz, 2H), 4.42–4.14 (m, 1H), 4.14–3.89 (m, 1H), 3.80 (s, 3H), 2.91 (q, J = 8.9 Hz, 1H), 2.60–2.47 (m, 1H), 2.47–2.39 (m, 1H), 2.32 (s, 3H) ppm.

^{13}C NMR (126 MHz, $CDCl_3$) δ 175.04, 140.87, 137.82, 131.37, 128.85, 128.41, 127.65, 126.77, 120.16, 88.18, 83.06, 69.31, 59.16, 52.23, 39.05, 37.17, 21.30 ppm.

HRMS (ESI) m/z [M+H]$^{+}$: calcd $C_{21}H_{22}NO_2^{+}$ 320.1645; found 320.1644.

IR (film): ν_{max} (cm^{-1}) 3329, 3029, 2946, 1723, 1558, 1496, 1213, 818, 702.

Optical rotation: $[\alpha]_D^{25}$ = 194.12 (c = 2.00, $CHCl_3$, 98% ee).

HPLC: DAICEL CHIRALPAK IE, hexane/i-PrOH = 43/7, flow rate: 0.5 mL/min, λ = 254 nm, t_R(minor) = 18.6 min, t_R(major) = 15.4 min, 98% ee.

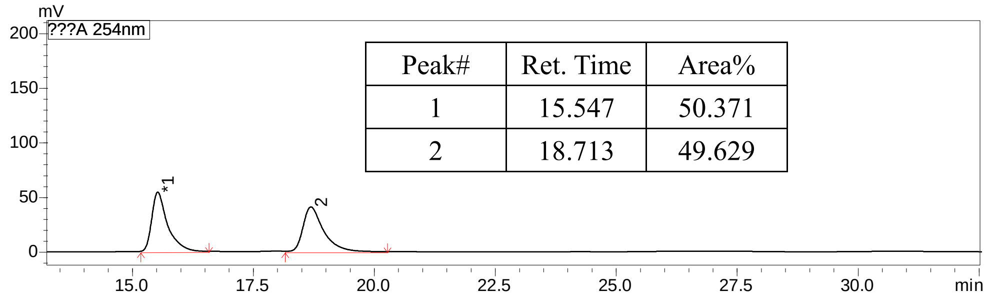

Peak#	Ret. Time	Area%
1	15.547	50.371
2	18.713	49.629

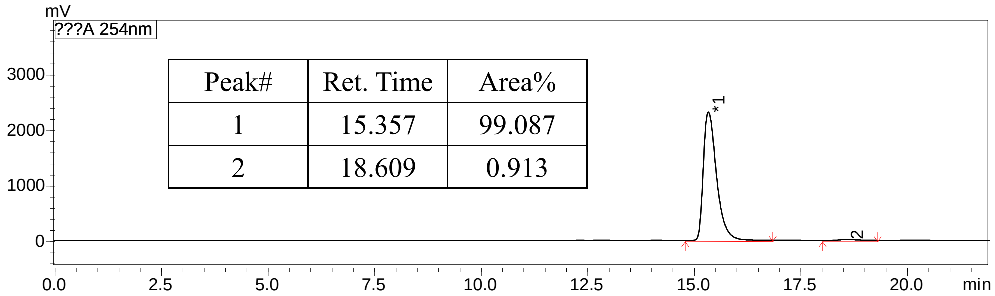

Peak#	Ret. Time	Area%
1	15.357	99.087
2	18.609	0.913

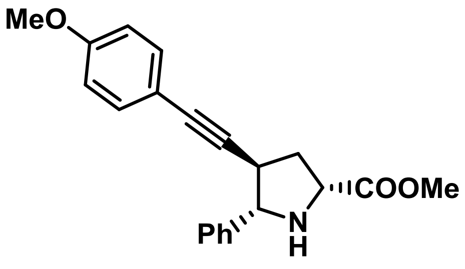

3ad

3ad: Procedure A, white solid, 53.9 mg, 80% yield, 13/1 dr (Diastereoselectivity was determined by ^{1}H NMR analysis of reaction crude mixture).

^{1}H NMR (400 MHz, $CDCl_3$) δ 7.59–7.50 (m, 2H), 7.40–7.34 (m, 2H), 7.32–7.26 (m, 3H), 6.79 (d, J = 8.8 Hz, 2H), 4.20 (d, J = 9.0 Hz, 1H), 4.07 (dd, J = 9.2, 4.5 Hz, 1H), 3.79 (s, 3H), 3.77 (s, 3H), 2.91 (td, J = 9.6, 8.0 Hz, 1H), 2.52 (ddd, J = 12.6, 7.9, 4.5 Hz, 1H), 2.41 (dt, J = 13.1, 9.5 Hz, 1H) ppm.

^{13}C NMR (126 MHz, $CDCl_3$) δ 175.07, 159.23, 140.96, 132.91, 128.46, 127.69, 126.83, 115.43, 113.76, 87.39, 82.78, 77.25, 69.49, 58.72, 55.20, 52.30, 38.97, 37.60 ppm.

HRMS (ESI) m/z [M+H]$^+$: calcd $C_{21}H_{22}NO_3^+$ 336.1594; found 336.1596.

IR (film): ν_{max} (cm^{-1}) 3331, 3031, 2984, 1721, 1608, 1509, 1217, 1106, 1030, 833, 762, 702.

Optical rotation: $[\alpha]_D^{25}$ = 176.88 (c = 2.00, $CHCl_3$, 98% ee).

HPLC: DAICEL CHIRALPAK IA-3, hexane/i-PrOH = 43/7, flow rate: 0.5 mL/min, λ = 254 nm, t_R(minor) = 21.7 min, t_R(major) = 17.1 min, 98% ee.

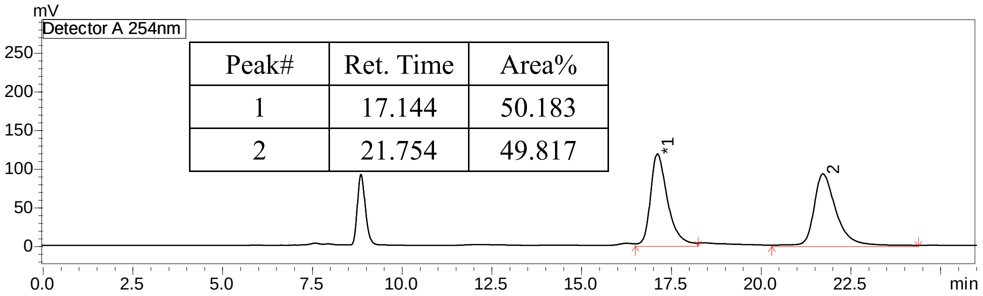

Peak#	Ret. Time	Area%
1	17.144	50.183
2	21.754	49.817

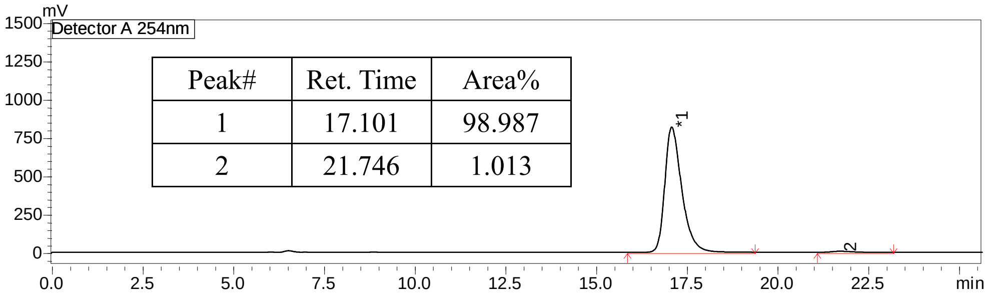

Peak#	Ret. Time	Area%
1	17.101	98.987
2	21.746	1.013

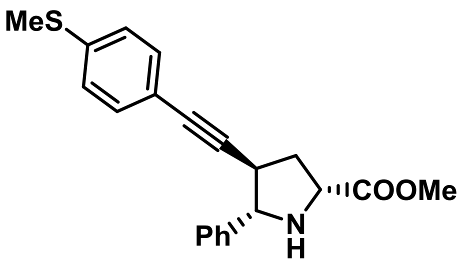

3ae

3ae: Procedure A, white solid, 69 mg, 98% yield, >20/1 dr (Diastereoselectivity was determined by ^{1}H NMR analysis of reaction crude mixture).

^{1}H NMR (400 MHz, $CDCl_3$) δ 7.60–7.50 (m, 2H), 7.41–7.34 (m, 2H), 7.33–7.23 (m, 3H), 7.20–7.09 (m, 2H), 4.31–4.15 (br, 1H), 4.15–4.00 (br, 1H), 3.80 (s, 3H), 2.92 (q, *J* = 8.9 Hz, 1H), 2.53 (ddd, *J* = 12.3, 7.9, 4.1 Hz, 1H), 2.49–2.37 (m, 4H) ppm.

^{13}C NMR (126 MHz, $CDCl_3$) δ 174.93, 140.79, 138.65, 131.75, 128.40, 127.66, 126.74, 125.71, 119.55, 88.97, 82.67, 69.31, 58.56, 52.22, 38.95, 37.44, 15.29 ppm.

HRMS (ESI) m/z [M+H]$^{+}$: calcd $C_{21}H_{22}NO_2S^{+}$ 352.1366; found 352.1366.

IR (film): ν_{max} (cm^{-1}) 3349, 3060, 2949, 1734, 1592, 1491, 1435, 1208, 1095, 819, 756, 700.

Optical rotation: $[\alpha]_D^{25}$ = 199.32 (*c* = 2.00, $CHCl_3$, 98% ee).

HPLC: DAICEL CHIRALPAK IE, hexane/*i*-PrOH = 45/5, flow rate: 0.5 mL/min, λ = 254 nm, t_R(minor) = 40.3 min, t_R(major) = 27.6 min, 98% ee.

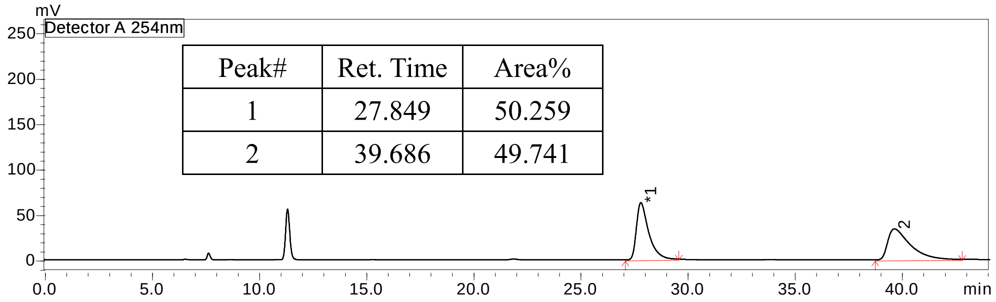

Peak#	Ret. Time	Area%
1	27.849	50.259
2	39.686	49.741

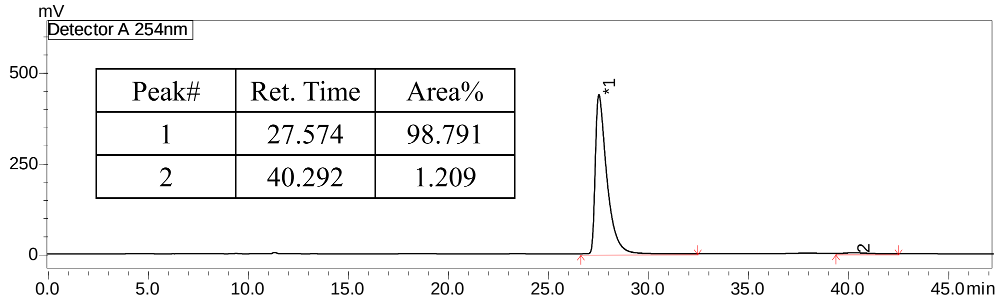

Peak#	Ret. Time	Area%
1	27.574	98.791
2	40.292	1.209

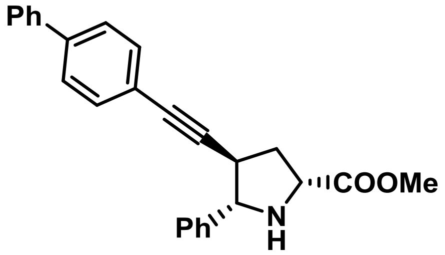

3af

3af: Procedure A, white solid, 71.3 mg, 93% yield, >20/1 dr (Diastereoselectivity was determined by ^{1}H NMR analysis of reaction crude mixture).

^{1}H NMR (400 MHz, $CDCl_3$) δ 7.62–7.54 (m, 4H), 7.54–7.49 (m, 2H), 7.47–7.39 (m, 4H), 7.39–7.35 (m, 2H), 7.35–7.27 (m, 2H), 4.24 (d, *J* = 9.0 Hz, 1H), 4.10 (dd, *J* = 9.3, 4.4 Hz, 1H), 3.81 (s, 3H), 2.96 (q, *J* = 9.3 Hz, 1H), 2.56 (ddd, *J* = 12.6, 7.9, 4.5 Hz, 1H), 2.45 (dt, *J* = 13.0, 9.5 Hz, 1H) ppm.

^{13}C NMR (101 MHz, $CDCl_3$) δ 175.04, 140.84, 140.60, 140.33, 131.98, 128.79, 128.52, 127.78, 127.52, 126.94, 126.85, 122.22, 89.66, 82.89, 69.49, 58.74, 52.36, 39.03, 37.55 ppm.

HRMS (ESI) m/z [M+H]$^+$: calcd $C_{26}H_{24}NO_2^+$ 382.1802; found 382.1803.

IR (film): ν_{max} (cm^{-1}) 3330, 3030, 2946, 1720, 1486, 1448, 1341, 1216, 841, 763, 699.

Optical rotation: $[\alpha]_D^{25}$ = 196 (*c* = 3.24, $CHCl_3$, 99% ee).

HPLC: DAICEL CHIRALPAK IBN-3, hexane/*i*-PrOH = 45/5, flow rate: 0.5 mL/min, λ = 254 nm, t_R(minor) = 29.5 min, t_R(major) = 49.8 min, 99% ee.

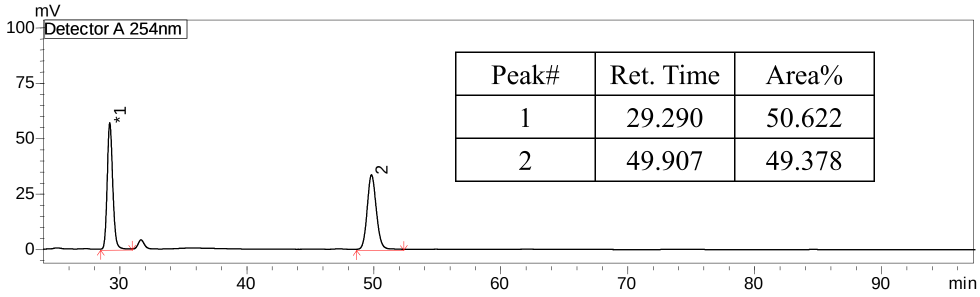

Peak#	Ret. Time	Area%
1	29.290	50.622
2	49.907	49.378

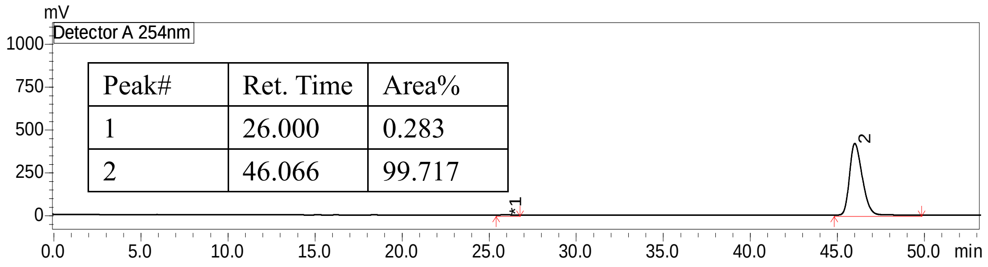

Peak#	Ret. Time	Area%
1	26.000	0.283
2	46.066	99.717

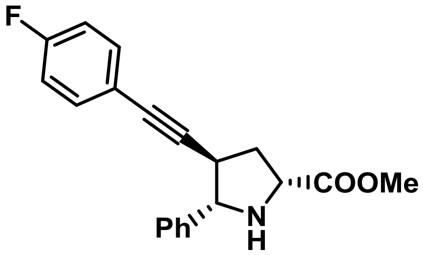

3ag

3ag: Procedure A, colorless oil, 64.1 mg, 99% yield, >20/1 dr (Diastereoselectivity was determined by ^{1}H NMR analysis of reaction crude mixture).

^{1}H NMR (400 MHz, $CDCl_3$) δ 7.59–7.49 (m, 2H), 7.41–7.22 (m, 5H), 7.01–6.89 (m, 2H), 4.20 (d, J = 9.1 Hz, 1H), 4.08 (dd, J = 9.4, 4.3 Hz, 1H), 3.79 (s, 3H), 2.92 (td, J = 9.6, 7.8 Hz, 1H), 2.53 (ddd, J = 12.5, 7.9, 4.3 Hz, 1H), 2.41 (dt, J = 13.0, 9.6 Hz, 1H) ppm.

^{13}C NMR (126 MHz, $CDCl_3$) δ 174.98, 162.18 (d, J = 248.9 Hz), 140.71, 133.37 (d, J = 8.3 Hz), 128.51, 127.80, 126.82, 119.33 (d, J = 3.6 Hz), 115.37 (d, J = 22.0 Hz), 88.54, 81.97, 77.25, 69.39, 58.65, 52.34, 38.83, 37.47 ppm.

^{19}F NMR (376 MHz, $CDCl_3$) δ -112.24 ppm.

HRMS (ESI) m/z [M+H]$^{+}$: calcd $C_{20}H_{19}FNO_2^{+}$ 324.1394; found 324.1394.

IR (film): ν_{max} (cm^{-1}) 3353, 3029, 2952, 1734, 1600, 1507, 1220, 1155, 836, 700.

Optical rotation: $[\alpha]_D^{25}$ = 158.98 (c = 2.13, $CHCl_3$, 98% ee).

HPLC: DAICEL CHIRALPAK IA-3, hexane/i-PrOH = 43/7, flow rate: 0.5 mL/min, λ = 254 nm, t_R(minor) = 16.4 min, t_R(major) = 14.1 min, 98% ee.

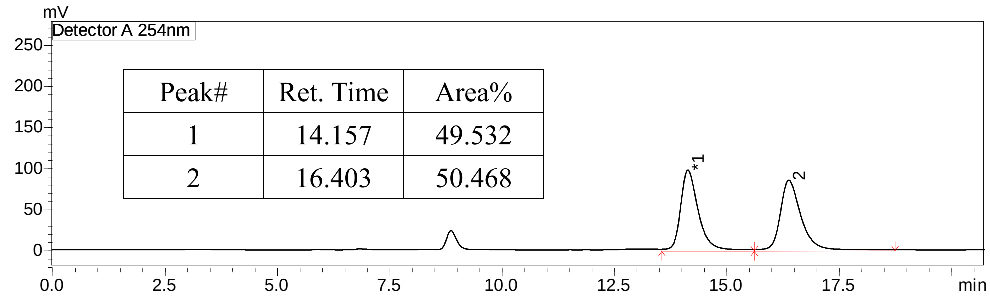

Peak#	Ret. Time	Area%
1	14.157	49.532
2	16.403	50.468

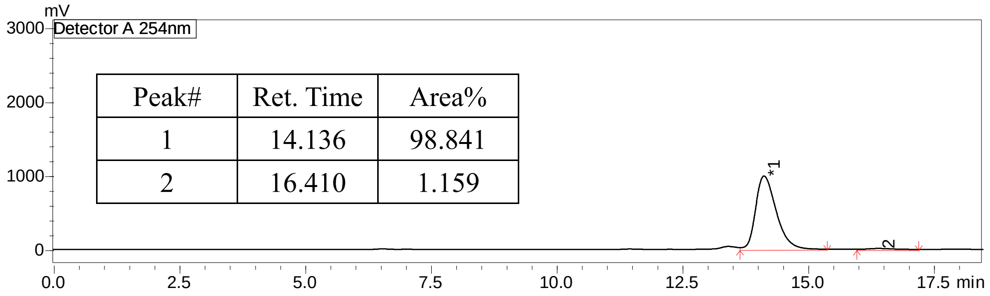

Peak#	Ret. Time	Area%
1	14.136	98.841
2	16.410	1.159

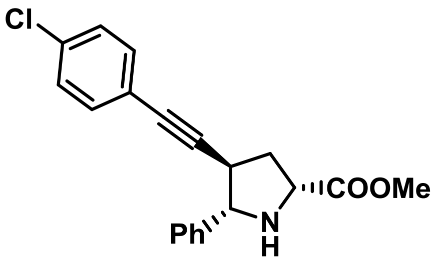

3ah

3ah: Procedure A, white solid, 67.5 mg, 99% yield, >20/1 dr (Diastereoselectivity was determined by ^{1}H NMR analysis of reaction crude mixture).

^{1}H NMR (400 MHz, $CDCl_3$) δ 7.60–7.50 (m, 2H), 7.37 (t, J = 7.3 Hz, 2H), 7.32 (d, J = 7.2 Hz, 1H), 7.30–7.21 (m, 5H), 4.32–4.15 (br, 1H), 4.15–3.98 (br, 1H), 3.80 (s, 3H), 2.98–2.82 (br, 1H), 2.59–2.48 (m, 1H), 2.48–2.36 (m, 1H) ppm.

^{13}C NMR (126 MHz, $CDCl_3$) δ 174.92, 140.69, 133.76, 132.74, 128.48, 128.41, 127.77, 126.77, 121.73, 90.00, 81.95, 69.29, 58.59, 52.28, 38.59, 37.38 ppm.

HRMS (ESI) m/z [M+H]$^{+}$: calcd $C_{20}H_{19}ClNO_2^{+}$ 340.1099; found 340.1099.

IR (film): ν_{max} (cm^{-1}) 3359, 3029, 2950, 1734, 1653, 1506, 1208, 1014, 750, 700, 668.

Optical rotation: $[\alpha]_D^{25}$ = 187.28 (c = 2.00, $CHCl_3$, 98% ee).

HPLC: DAICEL CHIRALPAK IE, hexane/i-PrOH = 35/15, flow rate: 0.5 mL/min, λ = 254 nm, t_R(minor) = 13.5 min, t_R(major) = 12.0 min, 98% ee.

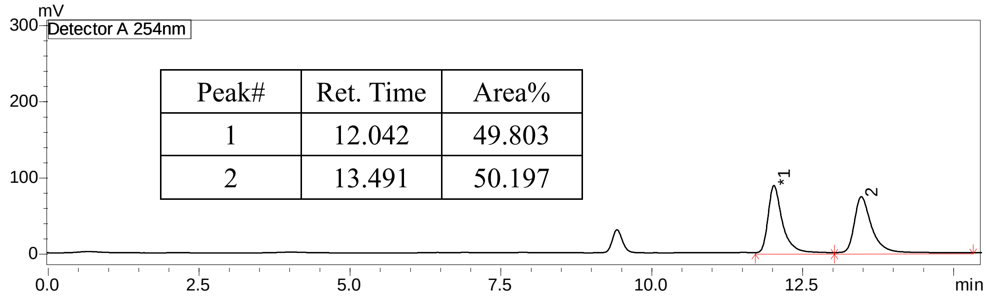

Peak#	Ret. Time	Area%
1	12.042	49.803
2	13.491	50.197

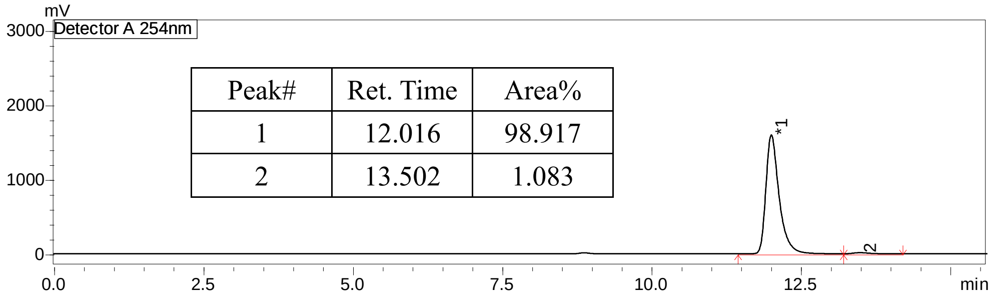

Peak#	Ret. Time	Area%
1	12.016	98.917
2	13.502	1.083

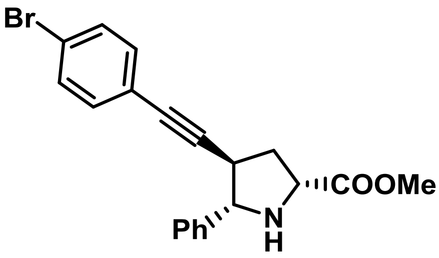

3ai

3ai: Procedure A, white solid, 74.1 mg, 96% yield, >20/1 dr (Diastereoselectivity was determined by ^{1}H NMR analysis of reaction crude mixture).

^{1}H NMR (400 MHz, $CDCl_3$) δ 7.58–7.51 (m, 2H), 7.44–7.34 (m, 4H), 7.33–7.27 (m, 1H), 7.24–7.16 (m, 2H), 4.21 (d, *J* = 9.0 Hz, 1H), 4.08 (dd, *J* = 9.2, 4.4 Hz, 1H), 3.80 (s, 3H), 2.92 (td, *J* = 9.6, 8.0 Hz, 1H), 2.53 (ddd, *J* = 12.6, 7.9, 4.4 Hz, 1H), 2.41 (dt, *J* = 13.0, 9.6 Hz, 1H) ppm.

^{13}C NMR (126 MHz, $CDCl_3$) δ 174.95, 140.75, 133.02, 131.39, 128.52, 127.81, 126.81, 122.25, 122.00, 90.25, 82.03, 69.41, 58.70, 52.35, 38.94, 37.39 ppm.

HRMS (ESI) m/z [M+H]$^+$: calcd $C_{20}H_{19}BrNO_2^+$ 384.0594; found 384.0594.

IR (film): ν_{max} (cm^{-1}) 3343, 3029, 2950, 1737, 1486, 1453, 1207, 1010, 823, 700, 525.

Optical rotation: $[\alpha]_D^{25}$ = 126.20 (*c* = 2.50, $CHCl_3$, 98% ee).

HPLC: DAICEL CHIRALPAK IA-3, hexane/*i*-PrOH = 43/7, flow rate: 0.5 mL/min, λ = 254 nm, t_R(minor) = 19.1 min, t_R(major) = 15.4 min, 98% ee.

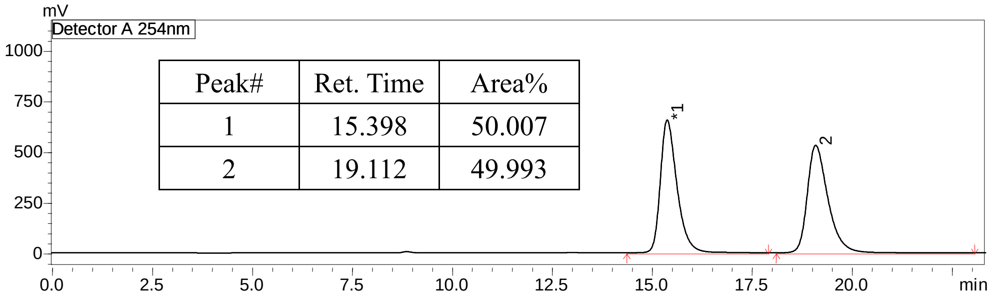

Peak#	Ret. Time	Area%
1	15.398	50.007
2	19.112	49.993

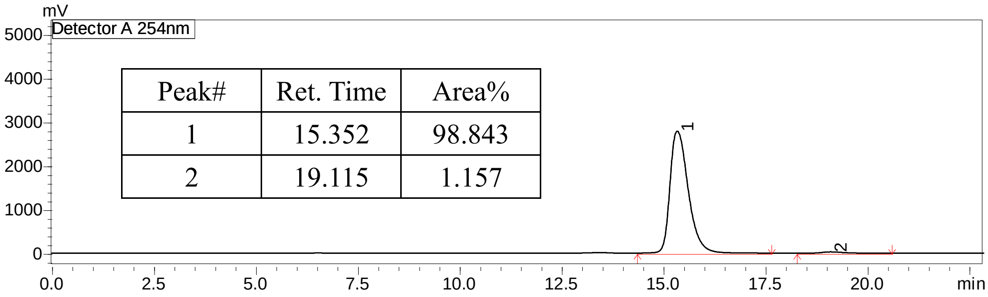

Peak#	Ret. Time	Area%
1	15.352	98.843
2	19.115	1.157

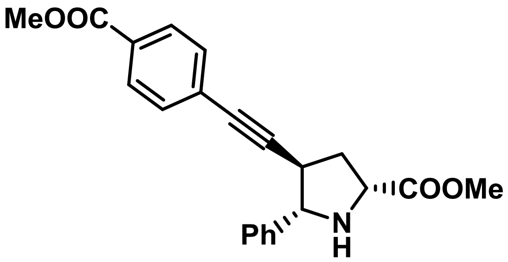

3aj

3aj: Procedure A, yellow oil, 72.8 mg, 99% yield, >20/1 dr (Diastereoselectivity was determined by ^{1}H NMR analysis of reaction crude mixture).

^{1}H NMR (400 MHz, $CDCl_3$) δ 7.99–7.91 (m, 2H), 7.60–7.51 (m, 2H), 7.46–7.34 (m, 4H), 7.34–7.28 (m, 1H), 4.23 (d, J = 9.1 Hz, 1H), 4.09 (dd, J = 9.2, 4.3 Hz, 1H), 3.90 (s, 3H), 3.80 (s, 3H), 2.97 (td, J = 9.6, 7.9 Hz, 1H), 2.56 (ddd, J = 12.5, 7.9, 4.4 Hz, 1H), 2.44 (dt, J = 13.0, 9.6 Hz, 2H) ppm.

^{13}C NMR (126 MHz, $CDCl_3$) δ 174.84, 166.44, 140.55, 131.45, 129.29, 129.12, 128.51, 127.99, 127.82, 126.77, 92.21, 82.43, 77.25, 69.30, 58.61, 52.31, 52.09, 38.90, 37.31 ppm.

HRMS (ESI) m/z [M+H]$^+$: calcd $C_{22}H_{22}NO_4^+$ 364.1544; found 364.1543.

IR (film): ν_{max} (cm^{-1}) 3348, 3028, 2951, 1723, 1605, 1435, 1275, 1108, 858, 769, 698.

Optical rotation: $[\alpha]_D^{25}$ = 169.15 (c = 2.00, $CHCl_3$, 98% ee).

HPLC: DAICEL CHIRALPAK IA, hexane/i-PrOH = 44/6, flow rate: 0.5 mL/min, λ = 254 nm, t_R(minor) = 26.4 min, t_R(major) = 20.8 min, 98% ee.

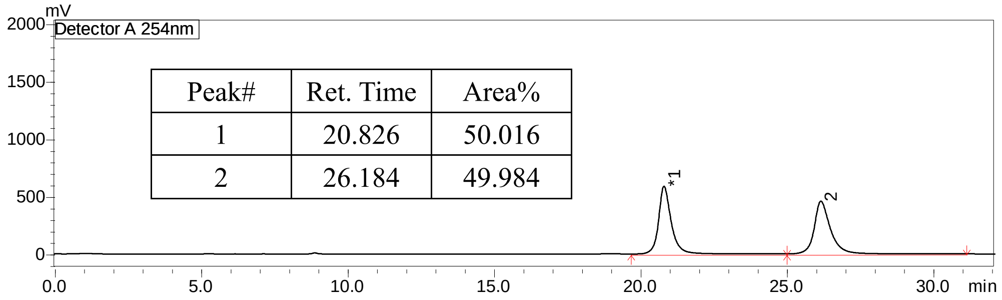

Peak#	Ret. Time	Area%
1	20.826	50.016
2	26.184	49.984

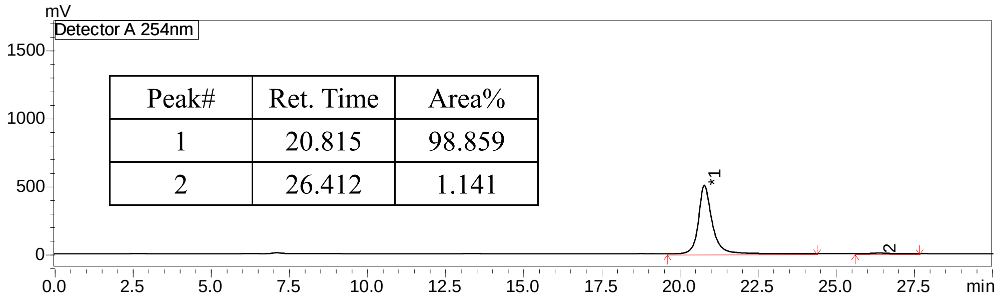

Peak#	Ret. Time	Area%
1	20.815	98.859
2	26.412	1.141

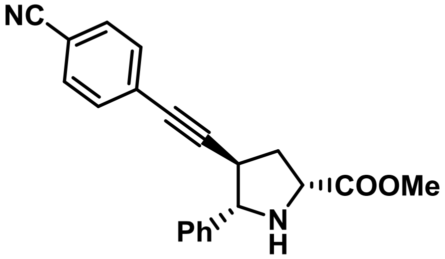

3ak

3ak: Procedure A, yellow oil, 59.5 mg, 90% yield, >20/1 dr (Diastereoselectivity was determined by ^{1}H NMR analysis of reaction crude mixture).

^{1}H NMR (500 MHz, $CDCl_3$) δ 7.61–7.53 (m, 4H), 7.47–7.43 (m, 2H), 7.43–7.38 (m, 2H), 7.37–7.32 (m, 1H), 4.22 (d, J = 9.2 Hz, 1H), 4.09 (dd, J = 9.3, 4.3 Hz, 1H), 3.81 (s, 3H), 2.99 (td, J = 9.6, 8.0 Hz, 1H), 2.59 (ddd, J = 12.5, 7.9, 4.3 Hz, 1H), 2.46 (dt, J = 13.0, 9.6 Hz, 1H) ppm.

^{13}C NMR (101 MHz, $CDCl_3$) δ 174.76, 140.40, 132.05, 131.81, 128.53, 128.16, 127.89, 126.74, 118.41, 111.09, 93.82, 81.67, 69.28, 58.58, 52.34, 38.86, 37.17 ppm.

HRMS (ESI) m/z [M+H]$^{+}$: calcd $C_{21}H_{19}N_2O_2^{+}$ 331.1441; found 331.1441.

IR (film): ν_{max} (cm^{-1}) 3366, 3030, 2951, 2226, 1735, 1603, 1457, 1208, 1106, 839, 701.

Optical rotation: $[\alpha]_D^{25}$ = 218.98 (c = 2.00, $CHCl_3$, 98% ee).

HPLC: DAICEL CHIRALPAK IE, hexane/i-PrOH = 35/15, flow rate: 0.5 mL/min, λ = 254 nm, t_R(minor) = 30.0 min, t_R(major) = 27.4 min, 98% ee.

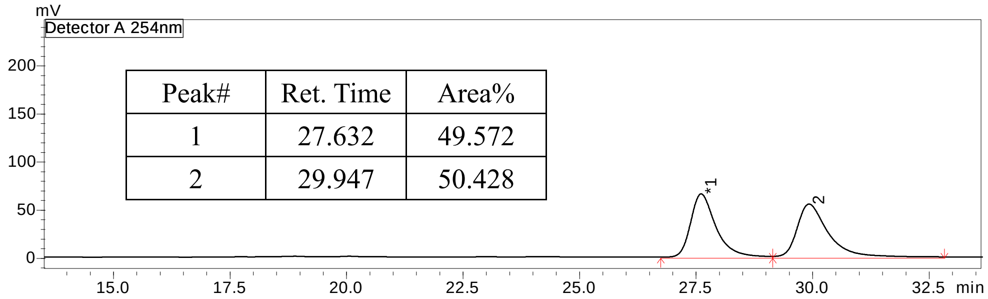

Peak#	Ret. Time	Area%
1	27.632	49.572
2	29.947	50.428

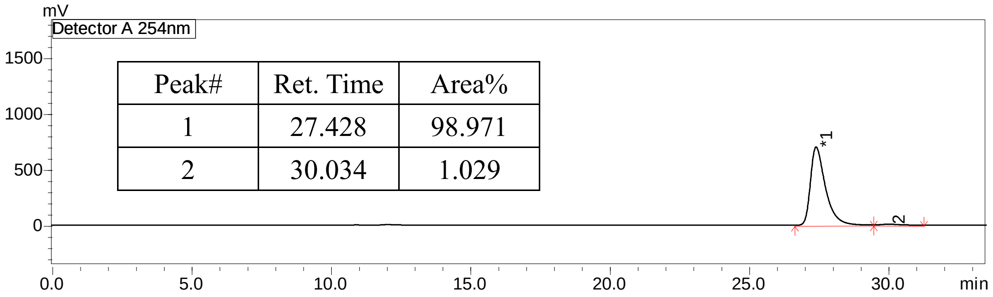

Peak#	Ret. Time	Area%
1	27.428	98.971
2	30.034	1.029

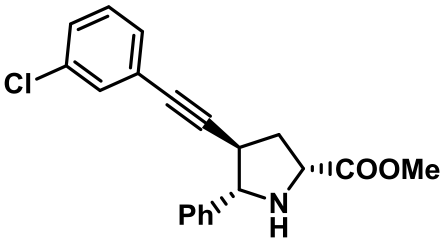

3al

3al: Procedure A, colorless oil, 67.2 mg, 99% yield, >20/1 dr (Diastereoselectivity was determined by ^{1}H NMR analysis of reaction crude mixture).

^{1}H NMR (500 MHz, $CDCl_3$) δ 7.60–7.53 (m, 2H), 7.43–7.38 (m, 2H), 7.38–7.31 (m, 2H), 7.29–7.20 (m, 3H), 4.21 (d, *J* = 9.1 Hz, 1H), 4.08 (dd, *J* = 9.4, 4.3 Hz, 1H), 3.80 (s, 3H), 2.93 (q, *J* = 9.0 Hz, 1H), 2.54 (ddd, *J* = 12.5, 7.9, 4.2 Hz, 1H), 2.42 (dt, *J* = 13.0, 9.6 Hz, 1H) ppm.

^{13}C NMR (101 MHz, $CDCl_3$) δ 174.93, 140.59, 133.93, 131.44, 129.70, 129.37, 128.55, 128.14, 127.86, 126.81, 124.97, 90.29, 81.74, 69.38, 58.67, 52.37, 38.84, 37.39 ppm.

HRMS (ESI) m/z [M+H]$^{+}$: calcd $C_{20}H_{19}ClNO_2^{+}$ 340.1099; found 340.1099.

IR (film): ν_{max} (cm^{-1}) 3346, 3029, 2950, 1736, 1592, 1493, 1208, 1077, 784, 700, 682.

Optical rotation: $[\alpha]_D^{25}$ = 161.51 (*c* = 2.00, $CHCl_3$, 98% ee).

HPLC: DAICEL CHIRALPAK IE, hexane/*i*-PrOH = 43/7, flow rate: 0.5 mL/min, λ = 254 nm, t_R(minor) = 18.1 min, t_R(major) = 15.9 min, 98% ee.

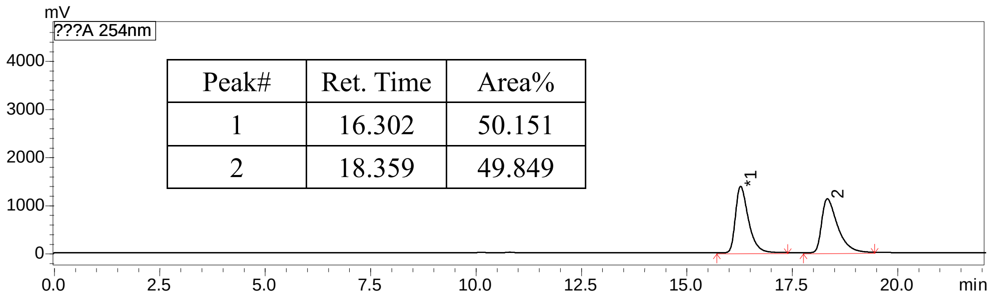

Peak#	Ret. Time	Area%
1	16.302	50.151
2	18.359	49.849

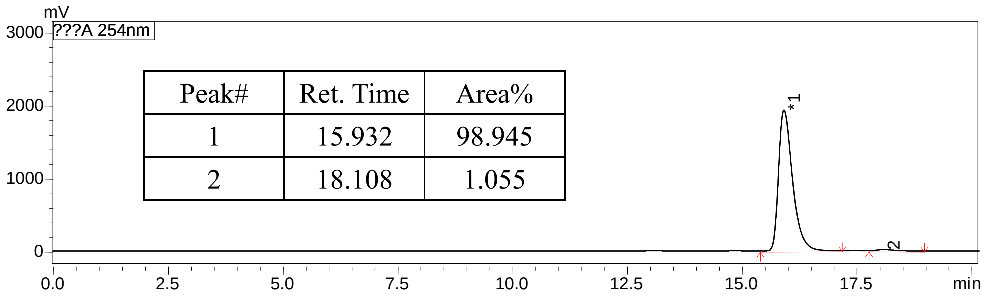

Peak#	Ret. Time	Area%
1	15.932	98.945
2	18.108	1.055

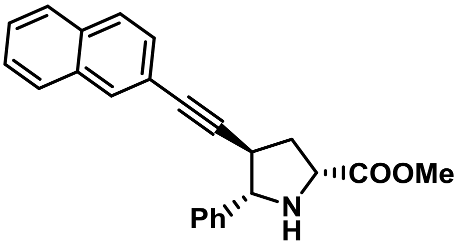

3am

3am: Procedure A, white solid, 70.9 mg, 99% yield, >20/1 dr (Diastereoselectivity was determined by ^{1}H NMR analysis of reaction crude mixture).

^{1}H NMR (400 MHz, $CDCl_3$) δ 7.87 (s, 1H), 7.83–7.71 (m, 3H), 7.60 (dd, J = 7.2, 1.8 Hz, 2H), 7.50–7.43 (m, 2H), 7.43–7.35 (m, 3H), 7.35–7.28 (m, 1H), 4.27 (d, J = 9.0 Hz, 1H), 4.12 (dd, J = 9.2, 4.5 Hz, 1H), 3.81 (s, 3H), 2.99 (td, J = 9.6, 8.0 Hz, 1H), 2.58 (ddd, J = 12.6, 7.9, 4.5 Hz, 1H), 2.48 (dt, J = 13.0, 9.5 Hz, 1H) ppm.

^{13}C NMR (126 MHz, $CDCl_3$) δ 174.96, 140.83, 132.86, 132.52, 131.19, 128.50, 127.76, 127.63, 127.53, 126.84, 126.38, 120.57, 89.32, 83.38, 69.45, 58.71, 52.30, 39.01, 37.52 ppm.

HRMS (ESI) m/z [M+H]$^{+}$: calcd $C_{24}H_{22}NO_2^{+}$ 356.1645; found 356.1645.

IR (film): ν_{max} (cm^{-1}) 3347, 3028, 2950, 1735, 1597, 1499, 1453, 1207, 817, 749, 700.

Optical rotation: $[\alpha]_D^{25}$ = 178.59 (c = 2.00, $CHCl_3$, 93% ee).

HPLC: DAICEL CHIRALPAK IA-3, hexane/i-PrOH = 43/7, flow rate: 0.5 mL/min, λ = 254 nm, t_R(minor) = 19.0 min, t_R(major) = 16.0 min, 93% ee.

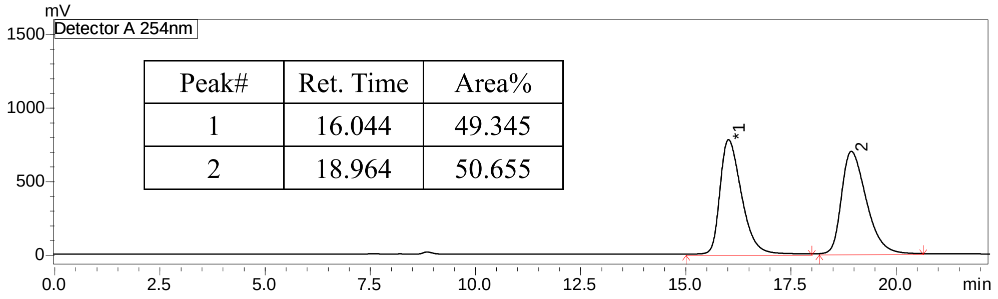

Peak#	Ret. Time	Area%
1	16.044	49.345
2	18.964	50.655

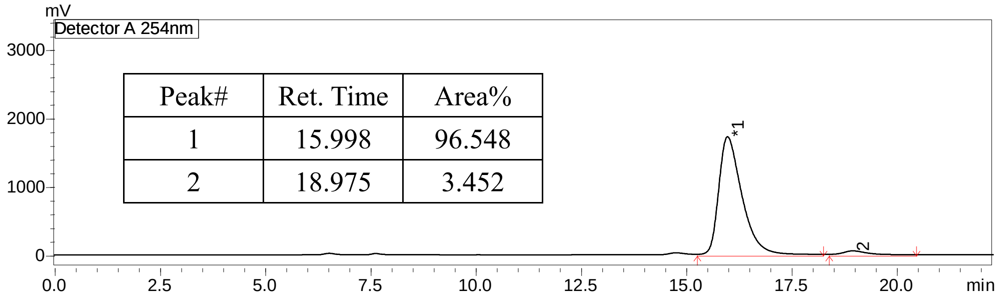

Peak#	Ret. Time	Area%
1	15.998	96.548
2	18.975	3.452

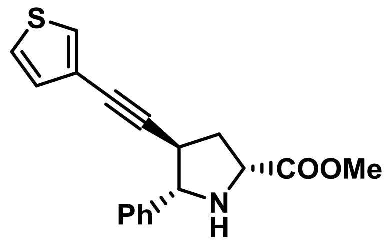

3an

3an: Procedure A, yellow solid, 60.8 mg, 97% yield, >20/1 dr (Diastereoselectivity was determined by ^{1}H NMR analysis of reaction crude mixture).

^{1}H NMR (400 MHz, $CDCl_3$) δ 7.55 (dd, *J* = 7.4, 1.8 Hz, 2H), 7.41–7.27 (m, 4H), 7.22 (dd, *J* = 5.1, 3.0 Hz, 1H), 7.03 (dd, *J* = 5.0, 1.1 Hz, 1H), 4.21 (d, *J* = 9.0 Hz, 1H), 4.08 (dd, *J* = 9.2, 4.5 Hz, 1H), 3.80 (s, 3H), 2.92 (td, *J* = 9.5, 8.0 Hz, 1H), 2.52 (ddd, *J* = 12.6, 7.9, 4.5 Hz, 1H), 2.42 (dt, *J* = 13.0, 9.5 Hz, 1H) ppm.

^{13}C NMR (126 MHz, $CDCl_3$) δ 174.96, 140.85, 129.87, 128.46, 128.07, 127.71, 126.81, 125.04, 122.24, 88.50, 78.03, 77.25, 69.35, 58.68, 52.27, 38.88, 37.45 ppm.

HRMS (ESI) m/z [M+H]$^{+}$: calcd $C_{18}H_{18}NO_2S^{+}$ 312.1053; found 312.1053.

IR (film): ν_{max} (cm^{-1}) 3367, 3029, 2950, 1734, 1507, 1451, 1208, 1073, 782, 753, 700.

Optical rotation: $[\alpha]_D^{25}$ = 180.48 (*c* = 2.00, $CHCl_3$, 98% ee).

HPLC: DAICEL CHIRALPAK IA-3, hexane/*i*-PrOH = 43/7, flow rate: 0.5 mL/min, λ = 254 nm, t_R(minor) = 18.4 min, t_R(major) = 15.6 min, 98% ee.

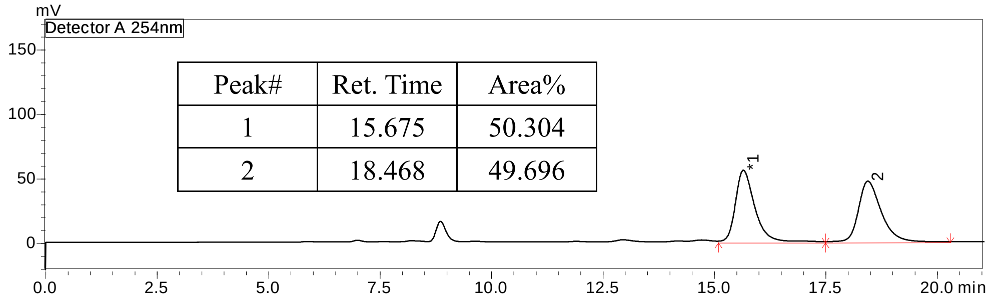

Peak#	Ret. Time	Area%
1	15.675	50.304
2	18.468	49.696

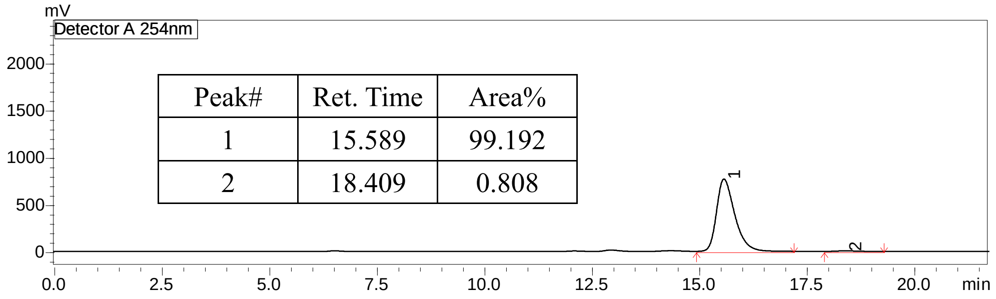

Peak#	Ret. Time	Area%
1	15.589	99.192
2	18.409	0.808

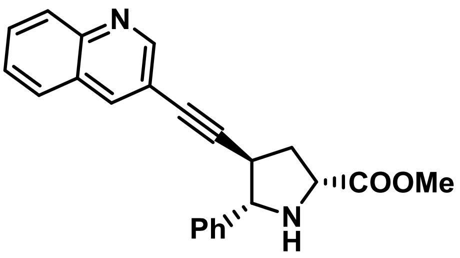

3ao

3ao: Procedure A, yellow oil, 55.6 mg, 78% yield, >20/1 dr (Diastereoselectivity was determined by ^{1}H NMR analysis of reaction crude mixture).

^{1}H NMR (400 MHz, $CDCl_3$) δ 8.88–8.77 (m, 1H), 8.14 (d, *J* = 1.9 Hz, 1H), 8.06 (d, *J* = 8.5 Hz, 1H), 7.79–7.65 (m, 2H), 7.64–7.50 (m, 3H), 7.44–7.36 (m, 2H), 7.36–7.29 (m, 1H), 4.28 (d, *J* = 9.1 Hz, 1H), 4.18–4.07 (m, 1H), 3.82 (s, 3H), 3.02 (q, *J* = 9.1 Hz, 1H), 2.60 (ddd, *J* = 12.5, 7.9, 4.3 Hz, 1H), 2.49 (dt, *J* = 13.0, 9.5 Hz, 1H) ppm.

^{13}C NMR (126 MHz, $CDCl_3$) δ 174.84, 152.16, 146.49, 140.53, 138.11, 129.78, 129.15, 128.53, 127.86, 127.34, 127.09, 126.78, 117.32, 92.48, 80.34, 69.37, 58.61, 52.31, 38.97, 37.32 ppm.

HRMS (ESI) m/z [M+H]$^{+}$: calcd $C_{23}H_{21}N_2O_2^{+}$ 357.1598; found 357.1597.

IR (film): ν_{max} (cm^{-1}) 3343, 3028, 2950, 1735,1566, 1488, 1207, 909, 751, 700.

Optical rotation: $[\alpha]_D^{25}$ = 200.93 (*c* = 2.23, $CHCl_3$, 99% ee).

HPLC: DAICEL CHIRALPAK IE, hexane/*i*-PrOH = 3/2, flow rate: 0.5 mL/min, λ = 254 nm, t_R(minor) = 32.8 min, t_R(major) = 23.4 min, 99% ee.

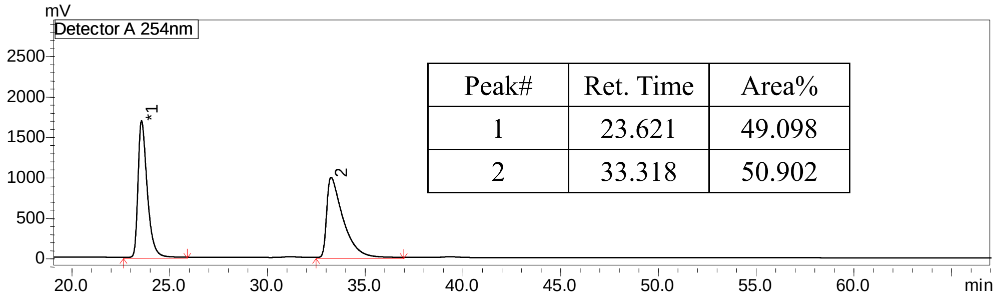

Peak#	Ret. Time	Area%
1	23.621	49.098
2	33.318	50.902

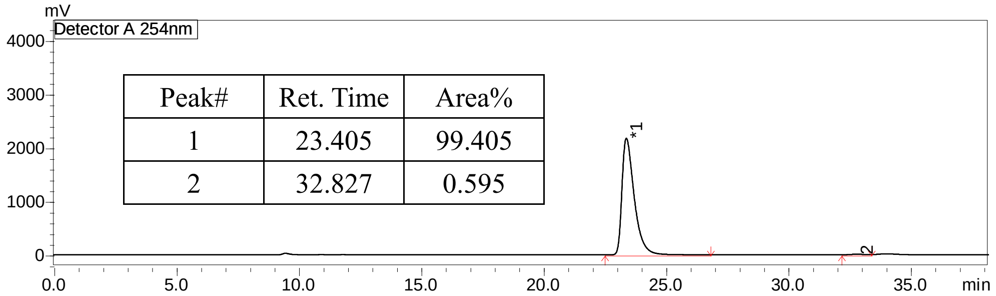

Peak#	Ret. Time	Area%
1	23.405	99.405
2	32.827	0.595

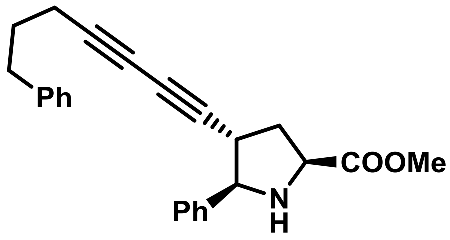

3ap

3ap: Procedure A, yellow oil, 59.4 mg, 80% yield, >20/1 dr (Diastereoselectivity was determined by ^{1}H NMR analysis of reaction crude mixture).

^{1}H NMR (400 MHz, $CDCl_3$) δ 7.50 (d, J = 7.3 Hz, 2H), 7.36 (t, J = 7.3 Hz, 2H), 7.32 – 7.25 (m, 3H), 7.21 – 7.14 (m, 3H), 4.35 – 4.08 (br, 1H), 4.08 – 3.87 (br, 1H), 3.77 (s, 3H), 2.87 – 2.73 (m, 1H), 2.70 (t, J = 7.6 Hz, 2H), 2.49 – 2.40 (m, 1H), 2.40 – 2.30 (m, 1H), 2.25 (t, J = 7.0 Hz, 2H), 1.82 (p, J = 7.1 Hz, 2H) ppm.

^{13}C NMR (126 MHz, $CDCl_3$) δ 174.93, 141.09, 140.42, 128.51, 128.40, 128.29, 127.82, 126.75, 125.90, 78.74, 67.81, 65.38, 52.29, 36.88, 34.58, 29.66, 18.50 ppm.

HRMS (ESI) m/z [M+H]$^{+}$: calcd $C_{25}H_{26}NO_2^{+}$372.1958; found 372.1959.

IR (film): ν_{max} (cm^{-1}) 3369, 3060, 2947, 1734, 1601, 1494, 1373, 1211, 746, 699.

Optical rotation: $[\alpha]_D^{25}$ = 157.37 (c = 2.75, $CHCl_3$, 98% ee).

HPLC: DAICEL CHIRALPAK IA, hexane/i-PrOH = 45/5, flow rate: 0.5 mL/min, λ = 254 nm, t_R(minor) = 16.3 min, t_R(major) = 15.1 min, 98% ee.

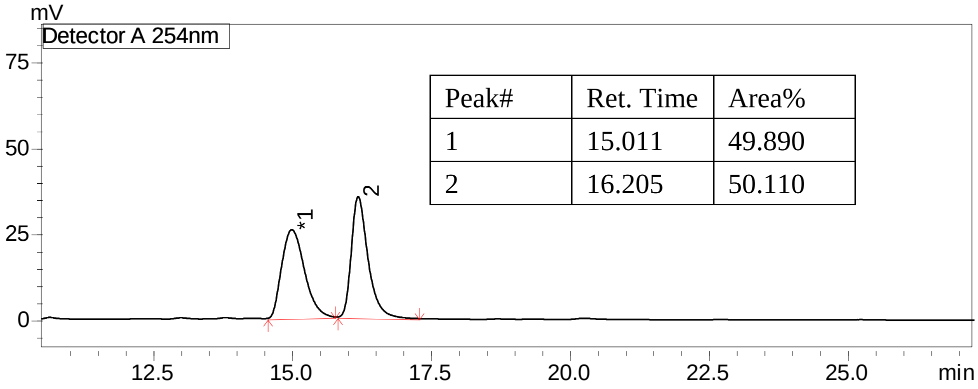

Peak#	Ret. Time	Area%
1	15.011	49.890
2	16.205	50.110

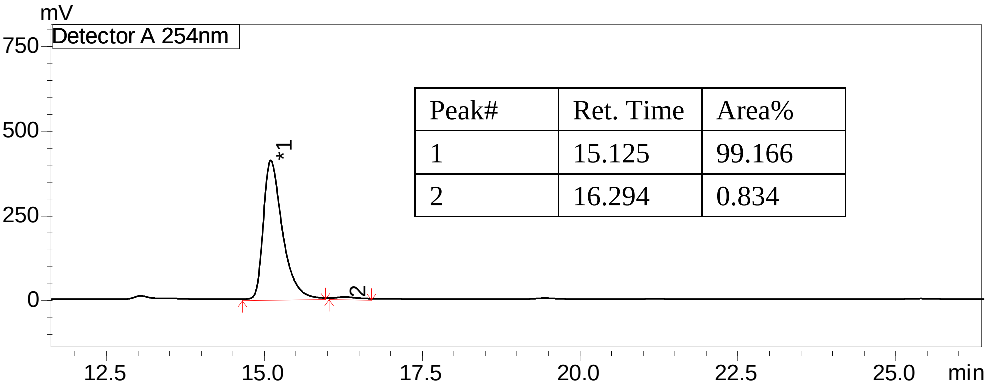

Peak#	Ret. Time	Area%
1	15.125	99.166
2	16.294	0.834

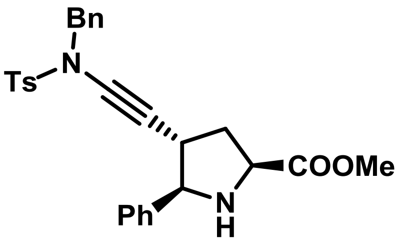

3aq

3ao: Procedure A, yellow oil, 93.8 mg, 96% yield, >20/1 dr (Diastereoselectivity was determined by ^{1}H NMR analysis of reaction crude mixture).

^{1}H NMR (400 MHz, $CDCl_3$) δ 7.60 (d, J = 8.3 Hz, 2H), 7.37 – 7.14 (m, 12H), 4.48 – 4.30 (m, 2H), 3.98 – 3.81 (m, 2H), 3.74 (s, 3H), 2.68 (q, J = 9.4 Hz, 1H), 2.40 (s, 3H), 2.33 (ddd, J = 12.1, 7.8, 4.1 Hz, 1H), 2.12 (dt, J = 12.9, 9.7 Hz, 1H) ppm.

^{13}C NMR (126 MHz, $CDCl_3$) δ 174.88, 144.28, 140.56, 134.31, 134.21, 129.44, 128.65, 128.26, 128.06, 127.49, 127.47, 126.72, 75.77, 70.01, 69.32, 58.41, 55.26, 52.16, 37.89, 37.32, 21.48 ppm.

HRMS (ESI) m/z [M+H]$^{+}$: calcd $C_{28}H_{29}N_2O_4S^{+}$ 489.1843; found 489.1843.

IR (film): ν_{max} (cm^{-1}) 3363, 3029, 2949, 2251, 1734, 1596, 1494, 1364, 813, 701, 544.

Optical rotation: $[\alpha]_D^{25}$ = 36.70 (c = 4.52, $CHCl_3$, 99% ee).

HPLC: DAICEL CHIRALPAK IA, hexane/i-PrOH = 3/2, flow rate: 0.5 mL/min, λ = 254 nm, t_R(minor) = 16.6 min, t_R(major) = 17.4 min, 99% ee.

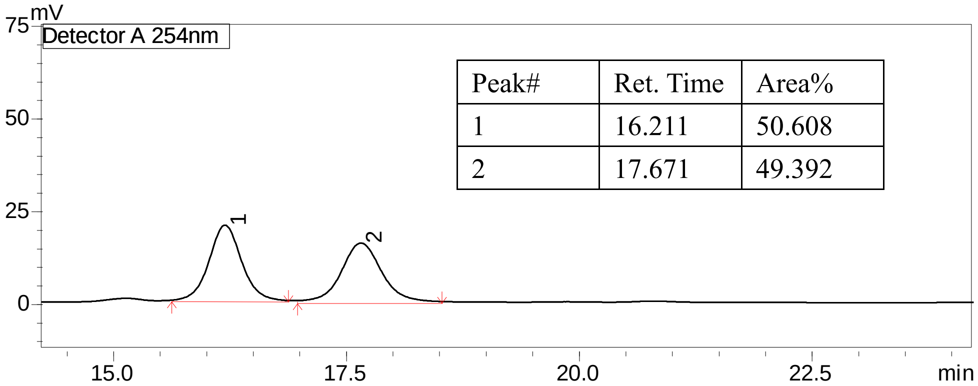

Peak#	Ret. Time	Area%
1	16.211	50.608
2	17.671	49.392

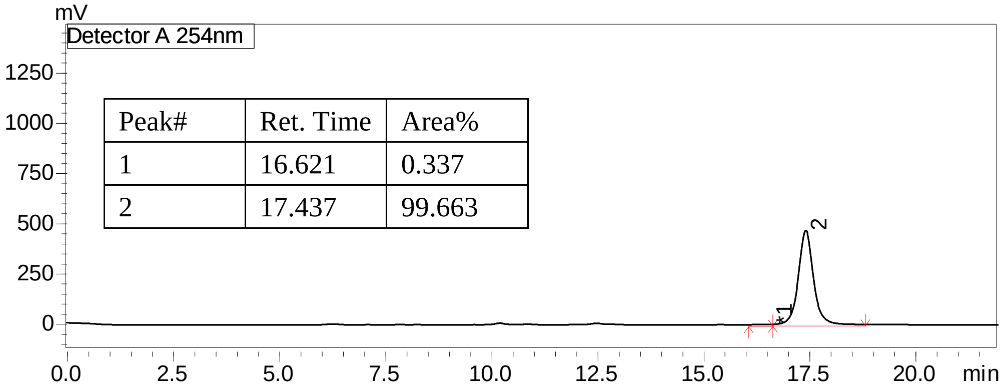

Peak#	Ret. Time	Area%
1	16.621	0.337
2	17.437	99.663

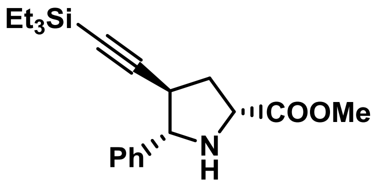

3ar

3ar: Procedure A, colorless oil, 55.3 mg, 80% yield, >20/1 dr (Diastereoselectivity was determined by ^{1}H NMR analysis of reaction crude mixture).

^{1}H NMR (500 MHz, $CDCl_3$) δ 7.51 (d, *J* = 7.0 Hz, 2H), 7.34 (t, *J* = 7.4 Hz, 2H), 7.33–7.30 (m, 1H), 4.13 (d, *J* = 9.1 Hz, 1H), 4.10–4.03 (m, 1H), 3.78 (s, 3H), 2.75 (q, *J* = 9.0 Hz, 1H), 2.49 (ddd, *J* = 12.5, 8.0, 4.4 Hz, 1H), 2.37 (dt, *J* = 12.9, 9.5 Hz, 1H), 0.94 (t, *J* = 7.9 Hz, 9H), 0.54 (q, *J* = 7.9 Hz, 6H) ppm.

^{13}C NMR (126 MHz, $CDCl_3$) δ 174.95, 140.73, 128.35, 127.66, 126.74, 107.19, 84.42, 69.59, 58.65, 52.27, 39.46, 37.69, 7.37, 4.39 ppm.

HRMS (ESI) m/z [M+H]$^{+}$: calcd $C_{20}H_{30}NO_2Si^{+}$ 344.2040; found 344.2041.

IR (film): ν_{max} (cm^{-1}) 3371, 3030, 2953, 2170, 1738, 1603, 1494, 1455, 1208, 726, 699.

Optical rotation: $[\alpha]_D^{25}$ = 97.44 (*c* = 0.60, $CHCl_3$, 99% ee).

HPLC: DAICEL CHIRALPAK IBN-3, hexane/*i*-PrOH = 45/5, flow rate: 0.5 mL/min, λ = 220 nm, t_R(minor) = 12.6 min, t_R(major) = 15.4 min, 99% ee.

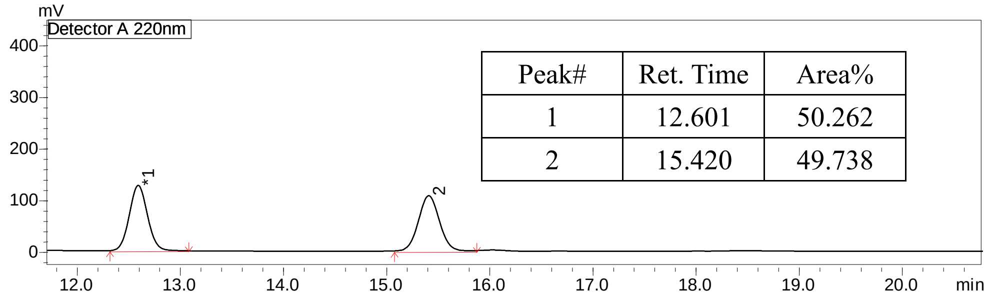

Peak#	Ret. Time	Area%
1	12.601	50.262
2	15.420	49.738

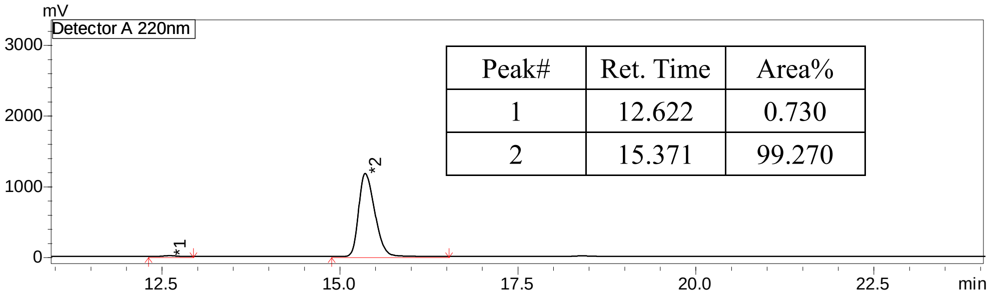

Peak#	Ret. Time	Area%
1	12.622	0.730
2	15.371	99.270

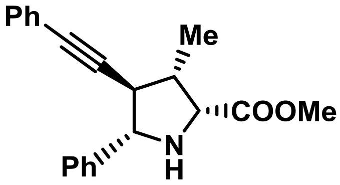

3as

3as: Procedure B, 10 mol % catalyst and 40 mol % Cs_2CO_3 was used, 1 M in DCE, 48 h. white solid, 31.3 mg, 49% yield, >20/1 dr (Diastereoselectivity was determined by ^{1}H NMR analysis of reaction crude mixture).

^{1}H NMR (400 MHz, $CDCl_3$) δ 7.66–7.59 (m, 3H), 7.41–7.33 (m, 4H), 7.33–7.29 (m, 1H), 7.29–7.23 (m, 3H), 4.22 (d, J = 8.3 Hz, 1H), 4.09 (d, J = 7.3 Hz, 1H), 3.79 (s, 3H), 2.80–2.63 (m, 2H), 1.18 (d, J = 6.5 Hz, 3H) ppm.

^{13}C NMR (126 MHz, $CDCl_3$) δ 174.88, 141.03, 131.64, 128.51, 128.14, 127.84, 127.76, 127.06, 123.36, 88.23, 83.59, 68.76, 63.18, 51.82, 46.45, 45.01, 14.32 ppm.

HRMS (ESI) m/z [M+H]$^+$: calcd $C_{21}H_{22}NO_2^+$ 320.1645; found 320.1647.

IR (film): ν_{max} (cm^{-1}) 3379, 3059, 2959, 1731, 1598, 1454, 1207, 756, 699.

Optical rotation: $[\alpha]_D^{25}$ = 73.95 (c = 1.32, $CHCl_3$, 92% ee).

HPLC: DAICEL CHIRALPAK IG-3, hexane/i-PrOH = 45/5, flow rate: 0.5 mL/min, λ = 254 nm, t_R(minor) = 21.1 min, t_R(major) = 17.6 min, 92% ee.

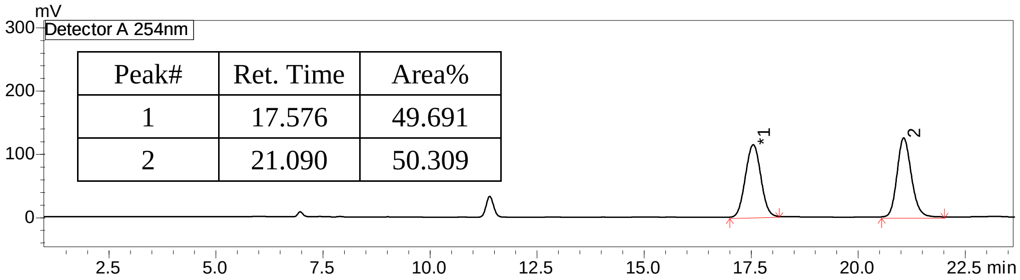

Peak#	Ret. Time	Area%
1	17.576	49.691
2	21.090	50.309

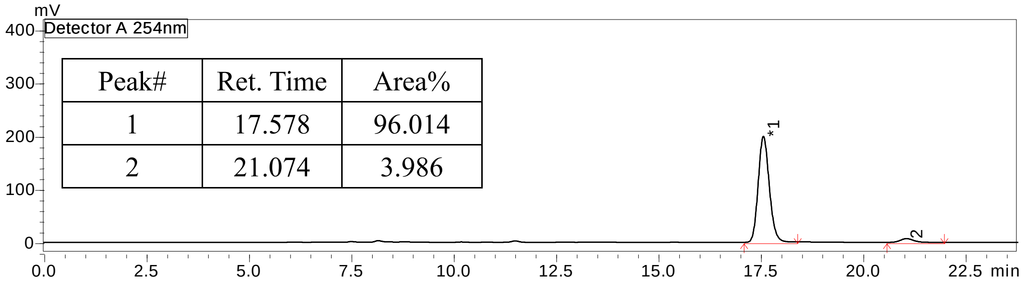

Peak#	Ret. Time	Area%
1	17.578	96.014
2	21.074	3.986

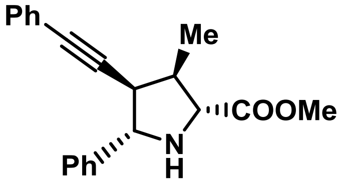

3at

3at: Procedure B, 10 mol % catalyst and 40 mol % Cs_2CO_3 was used, 1 M in DCE, 48 h. yellow solid, 34.7 mg, 54% yield, >20/1 dr (Diastereoselectivity was determined by ^{1}H NMR analysis of reaction crude mixture).

^{1}H NMR (400 MHz, $CDCl_3$) δ 7.59–7.55 (m, 2H), 7.40–7.34 (m, 4H), 7.32–7.24 (m, 4H), 4.34 (d, J = 8.4 Hz, 1H), 3.81 (s, 3H), 3.70 (d, J = 5.3 Hz, 1H), 3.07 (t, J = 8.2 Hz, 1H), 2.72–2.59 (m, 1H), 2.05 (broad, 1H), 1.37 (d, J = 7.1 Hz, 3H) ppm.

^{13}C NMR (101 MHz, $CDCl_3$) δ 174.82, 141.55, 131.57, 128.49, 128.19, 127.86, 127.63, 126.85, 123.45, 87.45, 85.24, 67.55, 66.58, 52.27, 44.58, 41.39, 16.47 ppm.

HRMS (ESI) m/z [M+H]$^+$: calcd $C_{21}H_{22}NO_2^+$ 320.1645; found 320.1645.

IR (film): ν_{max} (cm^{-1}) 2964, 1737, 1490, 1454, 1260, 1205, 1028, 756, 692.

Optical rotation: $[\alpha]_D^{25}$ = 119.47 (c = 0.89, $CHCl_3$, 98% ee).

HPLC: DAICEL CHIRALPAK IG-3, hexane/i-PrOH = 45/5, flow rate: 0.5 mL/min, λ = 254 nm, t_R(minor) = 20.0 min, t_R(major) = 18.5 min, 98% ee.

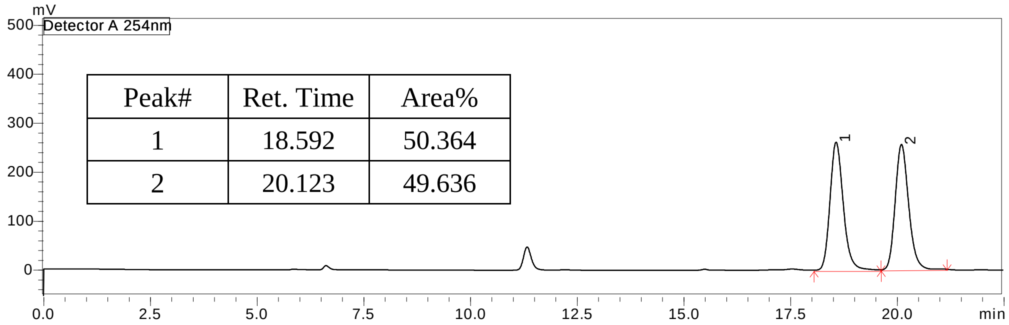

Peak#	Ret. Time	Area%
1	18.592	50.364
2	20.123	49.636

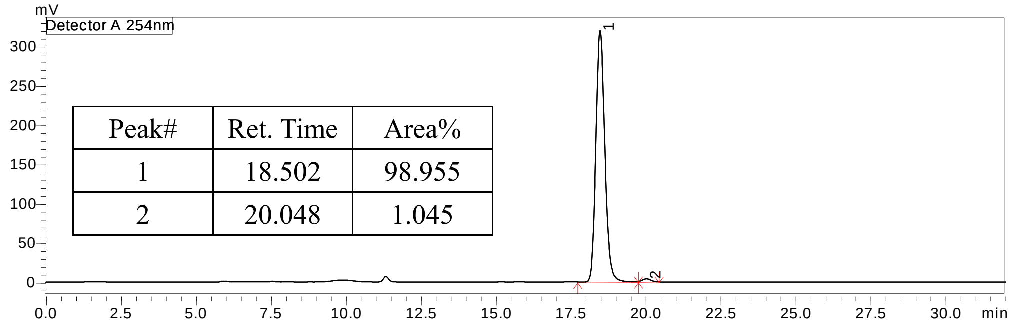

Peak#	Ret. Time	Area%
1	18.502	98.955
2	20.048	1.045

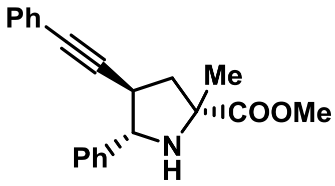

6a

6a: Procedure B, colorless oil, 44.8 mg, 70% yield, >20/1 dr (Diastereoselectivity was determined by ^{1}H NMR analysis of reaction crude mixture).

^{1}H NMR (500 MHz, $CDCl_3$) δ 7.59–7.52 (m, 2H), 7.42–7.34 (m, 4H), 7.34–7.31 (m, 1H), 7.31–7.26 (m, 4H), 4.30 (d, J = 9.7 Hz, 1H), 3.80 (s, 3H), 2.95 (ddd, J = 11.3, 9.7, 7.1 Hz, 1H), 2.86 (dd, J = 12.9, 7.2 Hz, 1H), 2.09 (dd, J = 12.9, 11.4 Hz, 1H), 1.56 (s, 3H) ppm.

^{13}C NMR (126 MHz, $CDCl_3$) δ 177.25, 140.89, 131.56, 128.49, 128.15, 127.83, 127.72, 126.85, 123.35, 88.73, 82.92, 68.78, 64.86, 52.64, 45.31, 40.34, 26.84 ppm.

HRMS (ESI) m/z [M+H]$^+$: calcd $C_{21}H_{22}NO_2^+$ 320.1645; found 320.1648.

IR (film): ν_{max} (cm^{-1}) 3354, 3030, 2951, 1733, 1599, 1490, 1447, 756, 692.

Optical rotation: $[\alpha]_D^{25}$ = 161.77 (c = 1.00, $CHCl_3$, 89% ee).

HPLC: DAICEL CHIRALPAK IBN-3, hexane/i-PrOH = 45/5, flow rate: 0.5 mL/min, λ = 254 nm, t_R(minor) = 12.0 min, t_R(major) = 10.9 min, 89% ee.

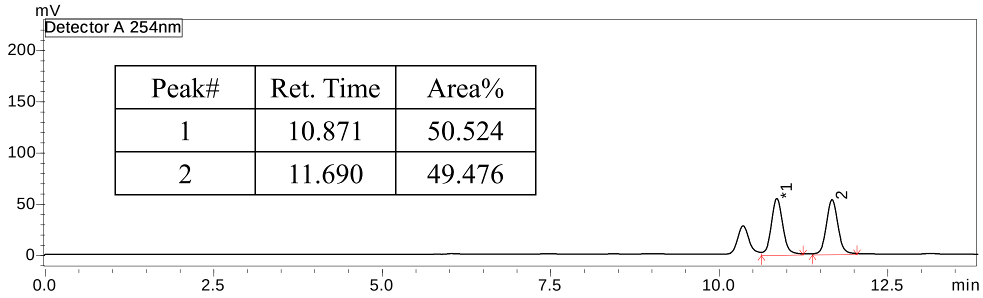

Peak#	Ret. Time	Area%
1	10.871	50.524
2	11.690	49.476

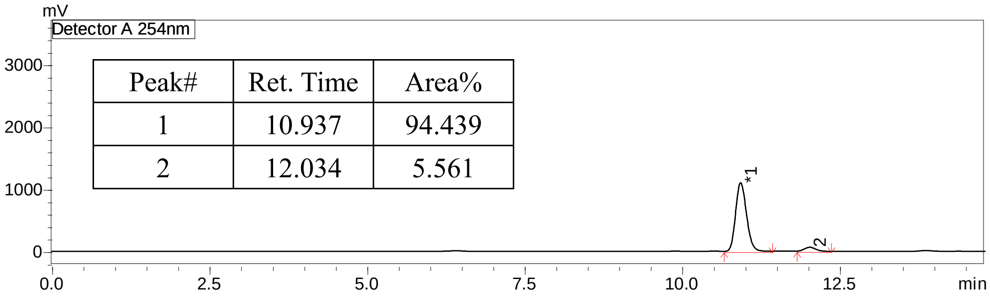

Peak#	Ret. Time	Area%
1	10.937	94.439
2	12.034	5.561

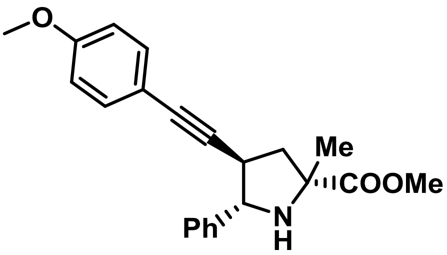

6d

6d: Procedure B, performed at 60 °C, colorless oil, 19.8 mg, 28% yield, >20/1 dr (Diastereoselectivity was determined by ^{1}H NMR analysis of reaction crude mixture).

^{1}H NMR (400 MHz, $CDCl_3$) δ 7.55–7.48 (m, 2H), 7.35 (t, J = 7.4 Hz, 2H), 7.31–7.24 (m, 3H), 6.82–6.74 (m, 2H), 4.28 (d, J = 9.6 Hz, 1H), 3.79 (s, 3H), 3.78 (s, 3H), 2.93 (ddd, J = 11.2, 9.6, 7.1 Hz, 1H), 2.84 (dd, J = 12.7, 7.2 Hz, 1H), 2.08 (dd, J = 12.8, 11.3 Hz, 1H), 1.55 (s, 3H) ppm.

^{13}C NMR (126 MHz, $CDCl_3$) δ 177.31, 159.21, 140.98, 132.92, 128.46, 127.67, 126.87, 115.48, 113.76, 87.13, 82.68, 68.83, 64.83, 55.23, 52.63, 45.40, 40.37, 26.88 ppm.

HRMS (ESI) m/z [M+H]$^+$: calcd $C_{22}H_{24}NO_3^+$ 350.1751; found 350.1751.

IR (film): ν_{max} (cm^{-1}) 3353, 2929, 1733, 1604, 1509, 1465, 1248, 832, 700.

Optical rotation: $[\alpha]_D^{25}$ = 160.09 (c = 0.96, $CHCl_3$, 78% ee).

HPLC: DAICEL CHIRALPAK IBN-3, hexane/i-PrOH = 47/3, flow rate: 0.5 mL/min, λ = 254 nm, t_R(minor) = 18.8 min, t_R(major) = 16.9 min, 78% ee.

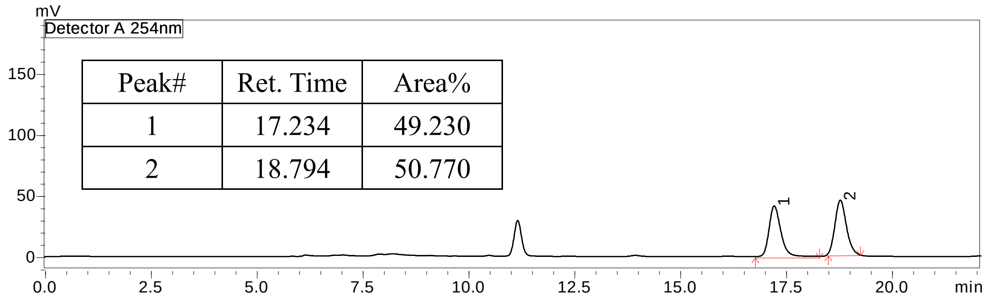

Peak#	Ret. Time	Area%
1	17.234	49.230
2	18.794	50.770

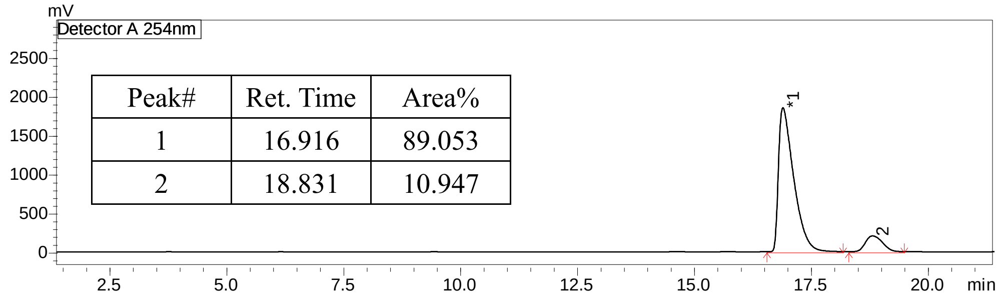

Peak#	Ret. Time	Area%
1	16.916	89.053
2	18.831	10.947

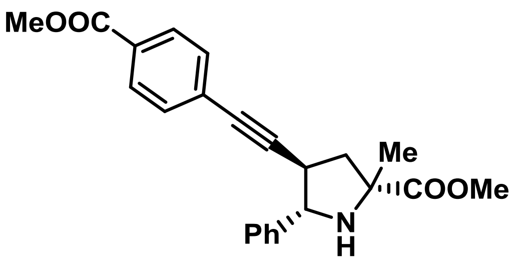

6j

6j: Procedure B, yellow oil, 75.2 mg, 99% yield, >20/1 dr (Diastereoselectivity was determined by ^{1}H NMR analysis of reaction crude mixture).

^{1}H NMR (400 MHz, $CDCl_3$) δ 8.00–7.88 (m, 2H), 7.51 (d, *J* = 7.0 Hz, 2H), 7.44–7.34 (m, 4H), 7.33–7.27 (m, 1H), 4.44–4.21 (br, 1H), 3.90 (s, 3H), 3.80 (s, 3H), 3.06–2.92 (m, 1H), 2.87 (dd, *J* = 12.8, 7.2 Hz, 1H), 2.14–2.02 (m, 1H), 1.56 (s, 3H) ppm.

^{13}C NMR (126 MHz, $CDCl_3$) δ 177.54, 164.62, 140.68, 131.40, 129.24, 129.04, 128.45, 127.99, 127.73, 126.72, 92.05, 83.31, 68.59, 64.72, 52.57, 52.05, 45.54, 30.53, 26.75 ppm.

HRMS (ESI) m/z [M+H]$^{+}$: calcd $C_{23}H_{24}NO_4^{+}$ 378.1700; found 378.1700.

IR (film): ν_{max} (cm^{-1}) 3353, 2952, 1724, 1605, 1506, 1435, 1278, 1107, 771, 697.

Optical rotation: $[\alpha]_D^{25}$ = 152.52 (*c* = 0.94, $CHCl_3$, 92% ee).

HPLC: DAICEL CHIRALPAK IBN-3, hexane/*i*-PrOH = 45/5, flow rate: 0.5 mL/min, λ = 254 nm, t_R(minor) = 17.5 min, t_R(major) = 15.2 min, 92% ee.

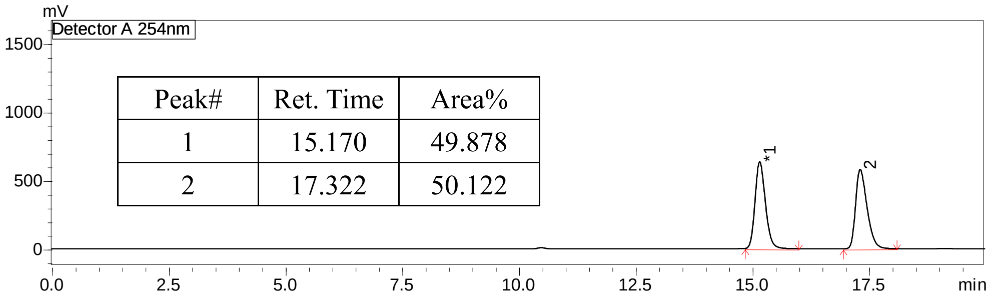

Peak#	Ret. Time	Area%
1	15.170	49.878
2	17.322	50.122

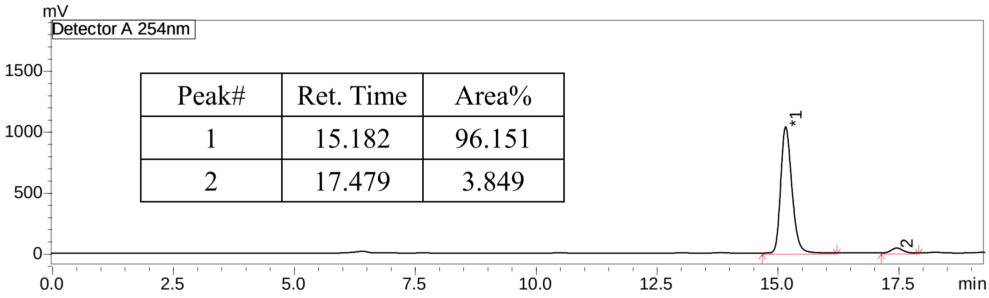

Peak#	Ret. Time	Area%
1	15.182	96.151
2	17.479	3.849

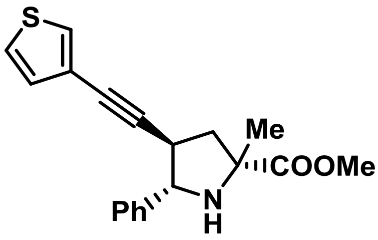

6n

6n: Procedure B, performed at 60 °C colorless oil, 37.7 mg, 58% yield, >20/1 dr (Diastereoselectivity was determined by ^{1}H NMR analysis of reaction crude mixture).

^{1}H NMR (400 MHz, $CDCl_3$) δ 7.54–7.48 (m, 2H), 7.39–7.32 (m, 2H), 7.32–7.24 (m, 2H), 7.20 (dd, J = 4.9, 3.0 Hz, 1H), 7.01 (dd, J = 4.9, 1.2 Hz, 1H), 4.28 (d, J = 9.7 Hz, 1H), 3.79 (s, 3H), 2.93 (ddd, J = 11.3, 9.6, 7.2 Hz, 1H), 2.84 (dd, J = 12.8, 7.2 Hz, 1H), 2.08 (dd, J = 12.8, 11.3 Hz, 1H), 1.54 (s, 3H) ppm.

^{13}C NMR (126 MHz, $CDCl_3$) δ 177.22, 140.84, 129.91, 128.48, 128.06, 127.72, 126.86, 125.03, 122.29, 88.20, 77.94, 68.69, 64.82, 52.63, 45.27, 40.27, 26.83 ppm.

HRMS (ESI) m/z [M+H]$^+$: calcd $C_{19}H_{20}NO_2S^+$ 326.1209; found 326.1210.

IR (film): ν_{max} (cm^{-1}) 3354, 3029, 2950, 1732, 1602, 1506, 1455, 1163, 757, 699.

Optical rotation: $[\alpha]_D^{25}$ = 152 (c = 1.50, $CHCl_3$, 80% ee).

HPLC: DAICEL CHIRALPAK IBN-3, hexane/i-PrOH = 47/3, flow rate: 0.5 mL/min, λ = 254 nm, t_R(minor) = 15.7 min, t_R(major) = 13.9 min, 80% ee.

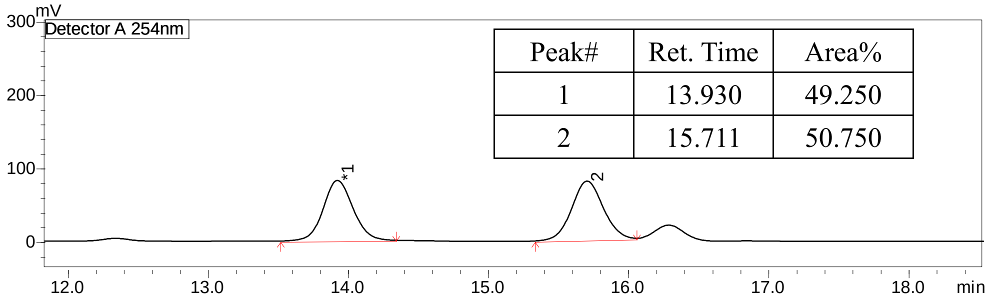

Peak#	Ret. Time	Area%
1	13.930	49.250
2	15.711	50.750

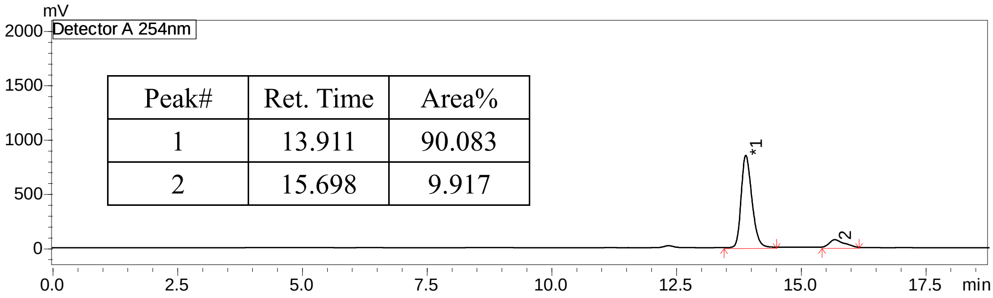

Peak#	Ret. Time	Area%
1	13.911	90.083
2	15.698	9.917

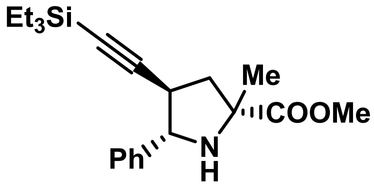

6r

6r: Procedure B, colorless oil, 38.1 mg, 53% yield, >20/1 dr (Diastereoselectivity was determined by ^{1}H NMR analysis of reaction crude mixture).

^{1}H NMR (400 MHz, $CDCl_3$) δ 7.51–7.44 (m, 2H), 7.37–7.30 (m, 2H), 7.30–7.24 (m, 1H), 4.24–4.17 (m, 1H), 3.77 (s, 3H), 2.81–2.69 (m, 2H), 2.05–1.93 (m, 1H), 1.52 (s, 3H), 0.93 (t, J = 7.9 Hz, 9H), 0.53 (q, J = 7.9 Hz, 6H) ppm.

^{13}C NMR (126 MHz, $CDCl_3$) δ 177.23, 140.93, 128.31, 127.58, 126.74, 107.01, 84.22, 68.97, 64.82, 52.55, 45.40, 40.92, 26.76, 7.38, 4.38 ppm.

HRMS (ESI) m/z [M+H]$^{+}$: calcd $C_{21}H_{32}NO_2Si^{+}$ 358.2197; found 358.2196.

IR (film): ν_{max} (cm^{-1}) 3359, 3030, 2954, 2170, 1732, 1496, 1456, 1162, 725, 699.

Optical rotation: $[\alpha]_D^{25}$ = 89.09 (c = 1.50, $CHCl_3$, 92% ee).

HPLC: DAICEL CHIRALPAK IC-3, hexane/i-PrOH = 475/25, flow rate: 0.5 mL/min, λ = 220 nm, t_R(minor) = 8.8 min, t_R(major) = 9.5 min, 92% ee.

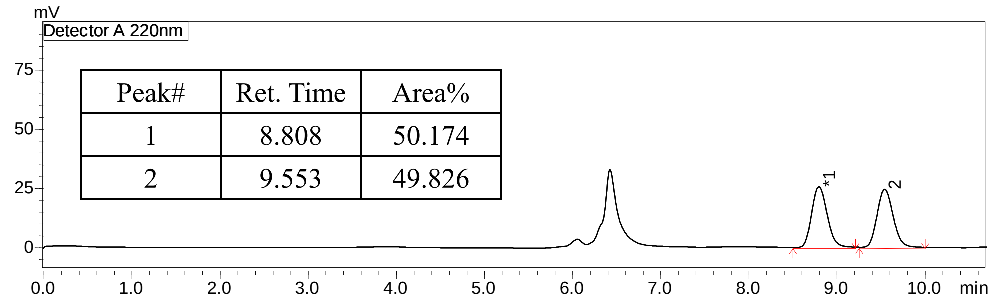

Peak#	Ret. Time	Area%
1	8.808	50.174
2	9.553	49.826

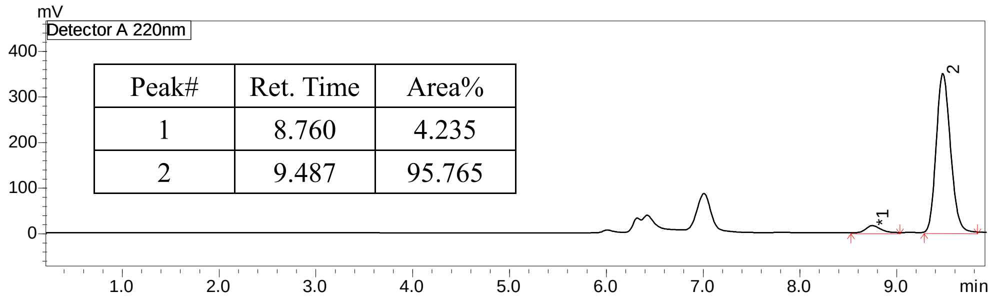

Peak#	Ret. Time	Area%
1	8.760	4.235
2	9.487	95.765

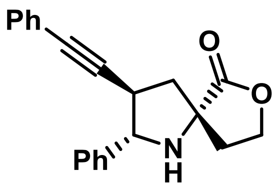

7a

7a: Procedure B, white solid, 57.2 mg, 90% yield, 10/1 dr (Diastereoselectivity was determined by ^{1}H NMR analysis of reaction crude mixture).

^{1}H NMR (400 MHz, $CDCl_3$) δ 7.59 (d, J = 7.4 Hz, 2H), 7.41–7.30 (m, 5H), 7.30–7.23 (m, 3H), 4.40 (dt, J = 9.1, 6.2 Hz, 1H), 4.34–4.23 (m, 2H), 3.31 (q, J = 9.4 Hz, 1H), 2.72 (dd, J = 12.9, 7.9 Hz, 1H), 2.51–2.40 (m, 2H), 2.28–2.08 (m, 2H) ppm.

^{13}C NMR (126 MHz, $CDCl_3$) δ 179.91, 140.18, 131.55, 128.64, 128.19, 127.99, 127.97, 126.95, 123.12, 88.52, 83.15, 69.62, 65.32, 64.58, 43.46, 39.91, 38.03 ppm.

HRMS (ESI) m/z [M+H]$^+$: calcd $C_{21}H_{20}NO_2^+$ 318.1489; found 318.1489.

IR (film): ν_{max} (cm^{-1}) 3352, 3060, 2962, 1771, 1598, 1490, 1443, 1139, 757, 693

Optical rotation: $[\alpha]_D^{25}$ = 163.73 (c = 1.78, $CHCl_3$, 92% ee).

HPLC: DAICEL CHIRALPAK IC, hexane/i-PrOH = 40/10, flow rate: 0.5 mL/min, λ = 254 nm, t_R(minor) = 22.5 min, t_R(major) = 21.7 min, 92% ee.

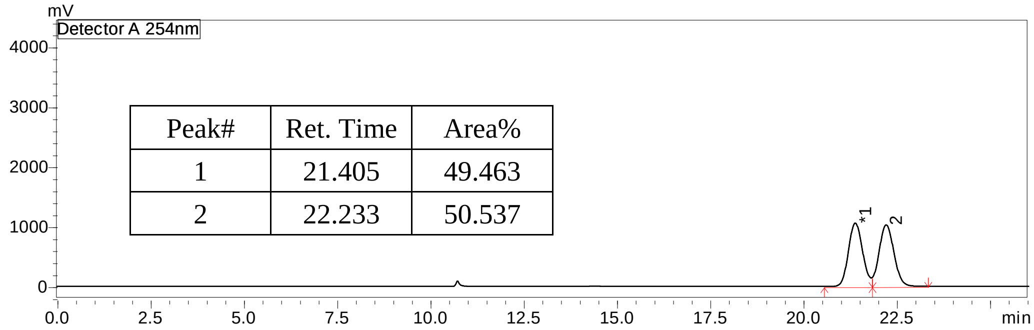

Peak#	Ret. Time	Area%
1	21.405	49.463
2	22.233	50.537

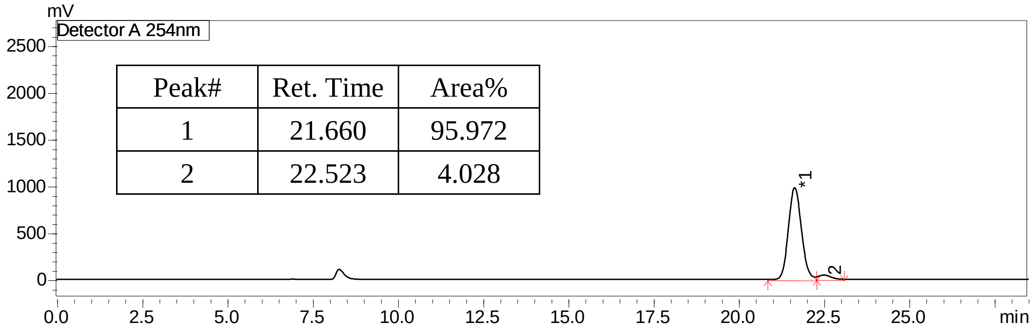

Peak#	Ret. Time	Area%
1	21.660	95.972
2	22.523	4.028

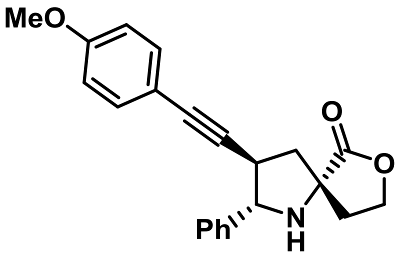

7d

7d: Procedure B, white solid, 60.7 mg, 87% yield, 7/1 dr (Diastereoselectivity was determined by ^{1}H NMR analysis of reaction crude mixture).

^{1}H NMR (400 MHz, $CDCl_3$) δ 7.59 (d, J = 7.3 Hz, 2H), 7.42–7.35 (m, 2H), 7.30 (dd, J = 14.6, 8.0 Hz, 3H), 6.80 (d, J = 8.8 Hz, 2H), 4.41 (dt, J = 9.2, 6.4 Hz, 1H), 4.34–4.24 (m, 2H), 3.79 (s, 3H), 3.30 (td, J = 9.5, 7.9 Hz, 1H), 2.71 (dd, J = 12.9, 7.9 Hz, 1H), 2.52–2.45 (m, 2H), 2.22 (dd, J = 12.9, 10.0 Hz, 1H) ppm.

^{13}C NMR (126 MHz, $CDCl_3$) δ 179.94, 159.29, 140.26, 132.91, 128.59, 127.93, 126.95, 115.22, 113.78, 86.94, 82.92, 69.62, 65.30, 64.54, 55.21, 43.52, 39.93, 38.03 ppm.

HRMS (ESI) m/z [M+H]$^+$: calcd $C_{22}H_{22}NO_3^+$ 348.1594; found 348.1595.

IR (film): ν_{max} (cm^{-1}) 3352, 2929, 1771, 1605, 1509, 1374, 1247, 1173, 833, 700.

Optical rotation: $[\alpha]_D^{25}$ = 151.39 (c = 1.54, $CHCl_3$, 89% ee).

HPLC: DAICEL CHIRALPAK IC, hexane/i-PrOH = 40/10, flow rate: 0.5 mL/min, λ = 254 nm, t_R(minor) = 34.9 min, t_R(major) = 31.8 min, 89% ee.

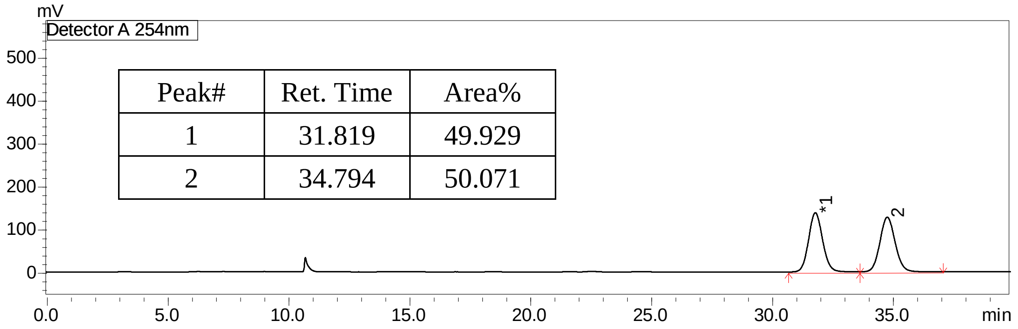

Peak#	Ret. Time	Area%
1	31.819	49.929
2	34.794	50.071

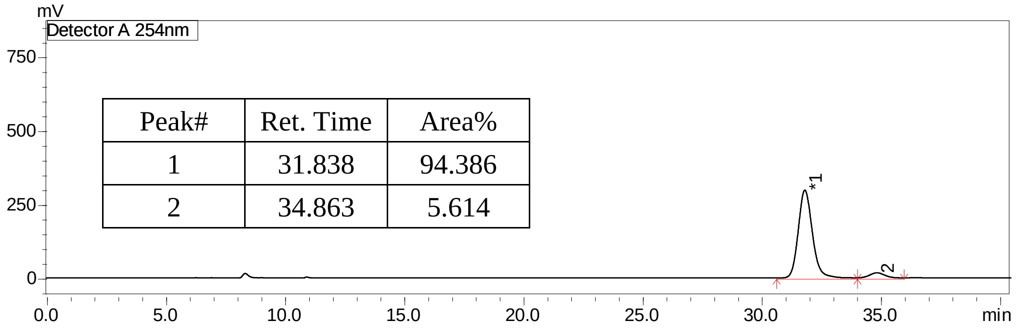

Peak#	Ret. Time	Area%
1	31.838	94.386
2	34.863	5.614

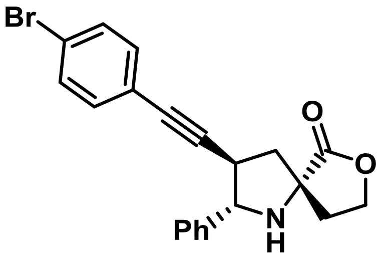

7i

7i: Procedure B, white solid, 74.3 mg, 94% yield, 14/1 dr (Diastereoselectivity was determined by ^{1}H NMR analysis of reaction crude mixture).

^{1}H NMR (400 MHz, $CDCl_3$) δ 7.57 (d, J = 7.1 Hz, 2H), 7.43–7.35 (m, 4H), 7.34–7.29 (m, 1H), 7.23–7.17 (m, 2H), 4.41 (dt, J = 9.2, 6.1 Hz, 1H), 4.34–4.24 (m, 2H), 3.39–3.24 (m, 1H), 2.72 (dd, J = 12.9, 7.8 Hz, 1H), 2.51–2.43 (m, 2H), 2.22 (dd, J = 12.9, 10.2 Hz, 1H) ppm.

^{13}C NMR (126 MHz, $CDCl_3$) δ 179.89, 140.02, 133.03, 131.44, 128.69, 128.10, 126.96, 122.16, 122.08, 89.70, 82.19, 69.55, 65.34, 64.58, 43.38, 39.90, 38.04 ppm.

HRMS (ESI) m/z [M+H]$^{+}$: calcd $C_{21}H_{19}BrNO_2^{+}$ 396.0594; found 396.0593.

IR (film): ν_{max} (cm^{-1}) 3353, 3061, 2924, 1770, 1600, 1486, 1374, 1171, 824, 700.

Optical rotation: $[\alpha]_D^{25}$ = 187.85 (c = 0.710, $CHCl_3$, 93% ee).

HPLC: DAICEL CHIRALPAK IC, hexane/i-PrOH = 40/10, flow rate: 0.5 mL/min, λ = 254 nm, t_R(minor) = 24.5 min, t_R(major) = 22.3 min, 93% ee.

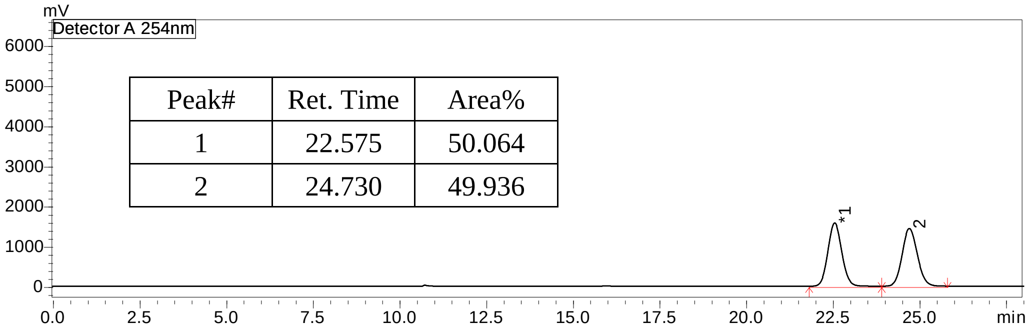

Peak#	Ret. Time	Area%
1	22.575	50.064
2	24.730	49.936

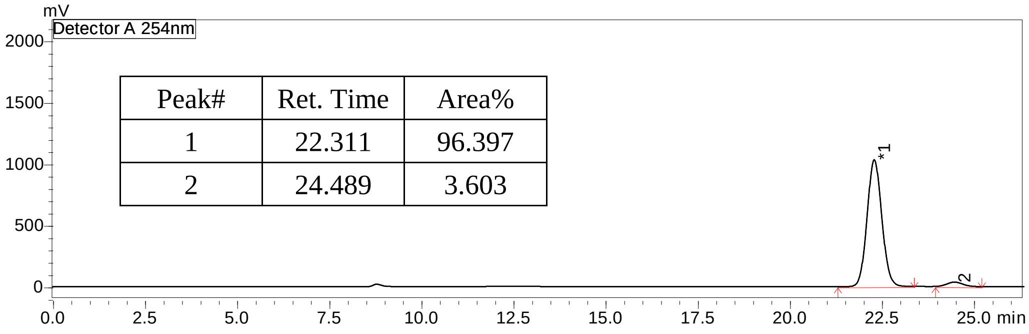

Peak#	Ret. Time	Area%
1	22.311	96.397
2	24.489	3.603

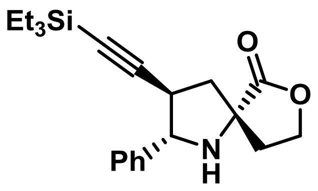

7r

7r: Procedure B, colorless oil, 56.8 mg, 80% yield, 6/1 dr (Diastereoselectivity was determined by ^{1}H NMR analysis of reaction crude mixture).

^{1}H NMR (400 MHz, $CDCl_3$) δ 7.53 (d, J = 7.2 Hz, 2H), 7.40 – 7.32 (m, 2H), 7.32 – 7.27 (m, 1H), 4.44 – 4.36 (m, 1H), 4.26 (dt, J = 18.0, 9.0 Hz, 2H), 3.10 (q, J = 9.3 Hz, 1H), 2.64 (dd, J = 12.9, 7.9 Hz, 1H), 2.49 – 2.40 (m, 2H), 2.13 (dd, J = 12.9, 9.9 Hz, 1H), 0.93 (t, J = 7.9 Hz, 9H), 0.54 (q, J = 7.9 Hz, 6H) ppm.

^{13}C NMR (126 MHz, $CDCl_3$) δ 179.82, 140.18, 128.47, 127.87, 126.83, 106.79, 84.64, 69.81, 65.28, 64.57, 43.56, 40.44, 37.88, 7.37, 4.34 ppm.

HRMS (ESI) m/z [M+H]$^{+}$: calcd $C_{21}H_{30}NO_2Si^{+}$ 356.2040; found 356.2040.

IR (film): ν_{max} (cm^{-1})3352, 3030, 2954, 2171, 1772, 1495, 1456, 1374, 1140, 734.

Optical rotation: $[\alpha]_D^{25}$ = 100.68 (c = 2.27, $CHCl_3$, 80% ee).

HPLC: DAICEL CHIRALPAK IBN-3, hexane/i-PrOH = 48/2, flow rate: 0.5 mL/min, λ = 220 nm, t_R(minor) = 36.3 min, t_R(major) = 27.6 min, 92% ee.

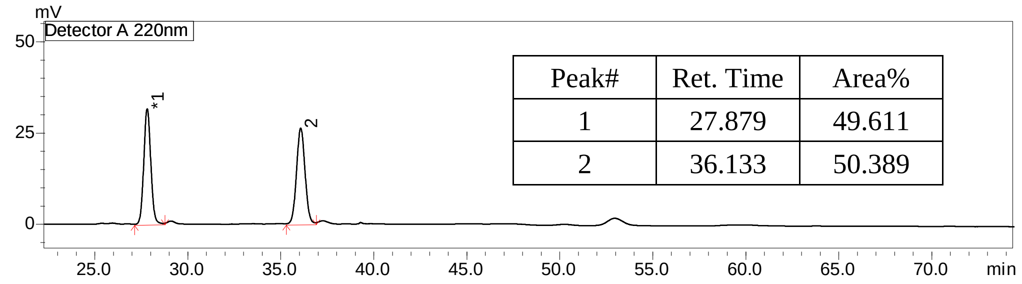

Peak#	Ret. Time	Area%
1	27.879	49.611
2	36.133	50.389

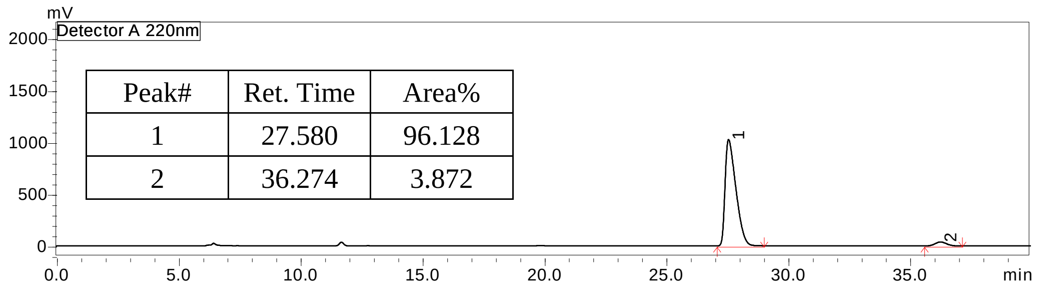

Peak#	Ret. Time	Area%
1	27.580	96.128
2	36.274	3.872

2. Supplementary Discussion

2.1 Computational Methods[18,19]

The DFT calculations were performed on the High-Performance Computing Clusters of Shanghai University of Traditional Chinese Medicine. Unless otherwise noted, geometry optimizations were performed with the B3LYP functional with Gaussian16 suite of programs. SDD and 6-31G(d) basis sets were used for Cu and all other atoms, respectively. Single-point energies were calculated with the B3LYP functional SDD and 6-311+G(d,p) basis sets were used for Cu and all other atoms, respectively. The SMD model was used for solvent corrections. Frequency calculations were also conducted with the optimized geometries to confirm that the stationary points were minima (zero imaginary frequencies) or transition states (one imaginary frequency). IRC calculations were conducted for important transition states to ensure that those were linked to proper starting materials and products. The 3D diagrams of molecules were generated using CYLView[20]. The independent gradient model based on Hirshfeld partition (IGMH） analysis was employed using Multiwfn software and VMD software, respectively[21,22].

To correct the Gibbs free energies under 1 atm to the standard state in solution (1 mol/L), a correction of RTln(cs/cg) is added to energies of all species. cs stands for the standard molar concentration in solution (1 mol/L), cg stands for the standard molar concentration in gas phase (0.040876 mol/L), and R is the gas constant. For calculated intermediates at the standard state of 1 mol/L at 298.15 K, the correction value equaling to 1.89 kcal/mol was used. We used the Conformer-Rotamer Ensemble Sampling Tool CREST (version 2.10.2)[23] in combination with the xTB package (version 6.1)[24] to sample low-energy conformations. The CREST conformational sampling was performed with GFN2-xTB in gas phase. Atoms in the forming/cleaving bonds were constrained by applying a force constant of 1.0 Hartree/Bohr2. An energy window of 6.0 kcal/mol and a RMSD threshold of 0.25 Å was used. Based on the conformers obtained by the CREST/xTB sampling, we optimized the geometries at the B3LYP-D3(BJ)/6-31G(d)/SDD(for Cu) level of theory, followed by single point energy calculations at the B3LYP-D3(BJ)/6-311+G(d,p)/SDD(for Cu) level of theory with SMD (solvent = dichloroethane) solvation model.

Mechanism study of the cyclization reaction process

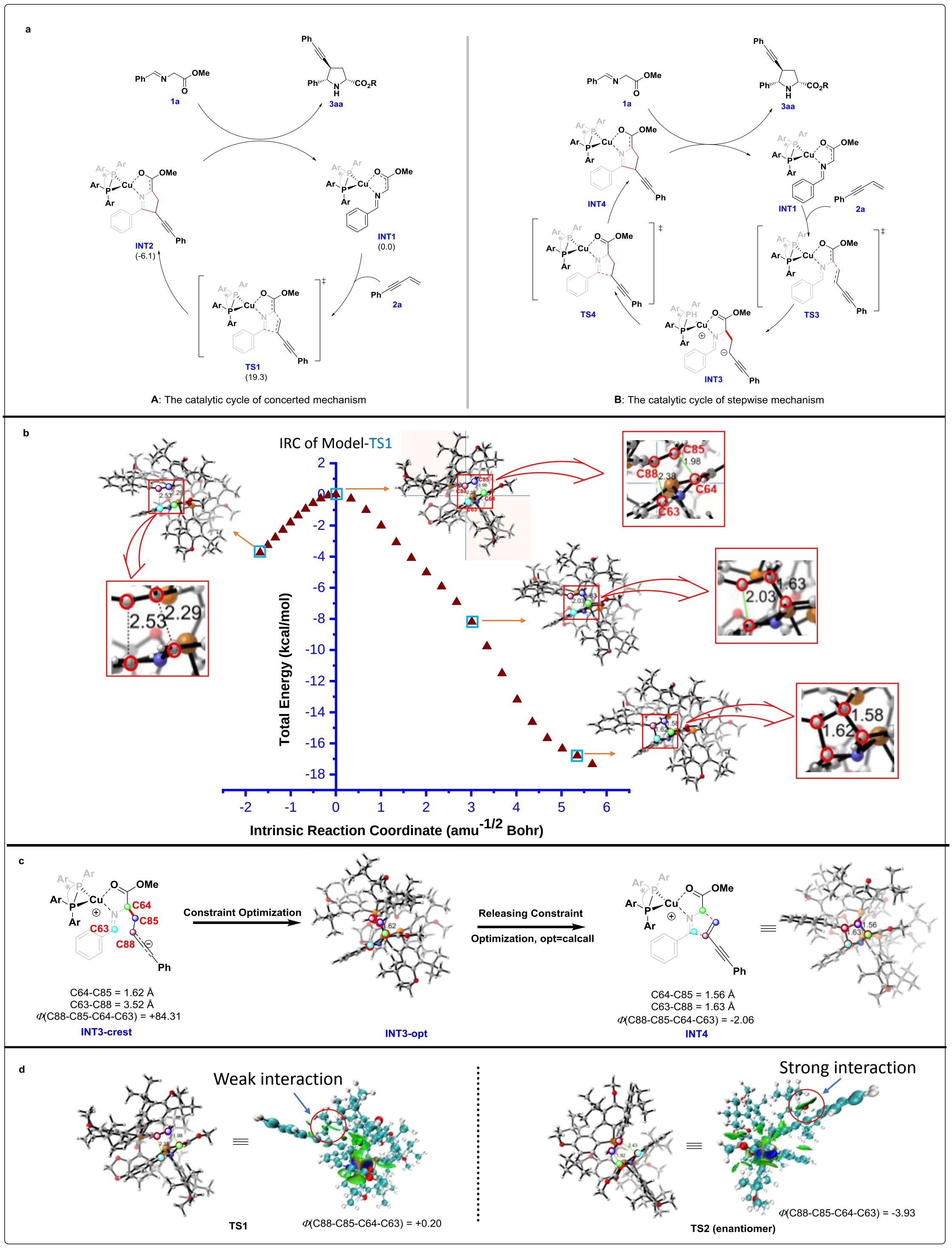

Supplementary Fig. 3. Mechanistic study of 1,3-dipolar cycloaddition of azomethine ylide and 1,3-enyne. a) Two possible pathways for the catalytic cycle that involves concerted mechanism (A) and stepwise mechanism (B); b) IRC calculation concerted reaction model of **TS1**. c) Attempt to locate the **INT3**. d) IGMH diagrams of **TS1** and **TS2**.

For the cyclization reaction process, we proposed two possible pathways (**Supplementary Fig. 1**) and studied them by DFT. Path A is the concerted mechanism, and the key transition state model-**TS1** directly connects to pre-intermediate **INT1** and the post-intermediate **INT2**. Path B is a stepwise dipolar cycloaddition mechanism and involves the zwitterionic intermediate **INT3**, which is *N*-metalated cycloadduct from **TS3**. We were able to locate the concerted transition state for Path A, which is confirmed by IRC calculations (**Supplementary Fig. 1-b**) and requires a barrier of 19.3 kcal/mol comparing with the pre-intermediate **INT1** (**Supplementary Fig. 1-a**). While for the Path B, the proposed the zwitterionic intermediate **INT3** could not be located. All the attempts to optimize the ionic intermediate **INT3** led to the cyclized **INT4** (**Supplementary Fig. 1-c**). These results indicate that the concerted pathway is a reasonable pathway in this copper(I)-catalyzed 1,3-dipolar cycloaddition of 1,3-enynes and azomethine ylides. In addition, through analysis of the structures of the competing transition states **TS1** and **TS2**, the close distance between one of the ^{t}Bu groups in (*R*)-DTBM-SEGPHOS and the alkynyl and phenyl groups in 1,3-enyne **2a** in the disfavored **TS2** leads to more van der Waals interactions and bigger dihedral angle of C88-C85-C64-C63 in the relatively unfavored cyclization transition state, which results in the observed absolute stereochemistry.

2.2 Determination of the Absolute Configurations of Products

Determination of the absolute configuration of 3ai

A solution of **3ai** in PE/DCM in a 25 ml flask was left at room temperature to grow single crystals. The absolute configuration of **3ai** was determined by X-ray crystallographic analysis. Other products (**3aa-3oa** and **3ab-3ap**) were deduced by analogy.

CCDC 2093924 contains the supplementary crystallographic data of **3ai**.

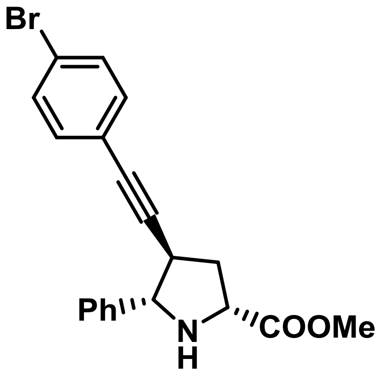

3ai
CCDC : 2093924

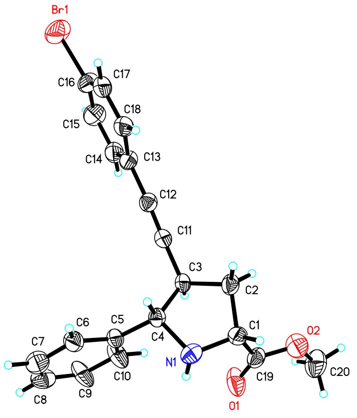

Supplementary Fig. 4. X-ray structure of **3ai** (CCDC 2093924, 30% ellipsoid contour probability)

Identification code	mo_d8v21295_0m
Empirical formula	C20 H18 Br N O2
Formula weight	384.26
Temperature	293(2) K

Wavelength	0.71073 Å	
Crystal system	Orthorhombic	
Space group	P 21 21 21	
Unit cell dimensions	a = 5.1496(2) Å	α= 90 °.
	b = 8.8621(4) Å	β= 90 °.
	c = 39.1115(18) Å	γ = 90 °.
Volume	1784.90(13) $Å^3$	
Z	4	
Density (calculated)	1.430 Mg/m^3	
Absorption coefficient	2.314 mm^{-1}	
F(000)	784	
Crystal size	0.180 x 0.150 x 0.120 mm^3	
Theta range for data collection	2.356 to 25.975 °.	
Index ranges	-5<=h<=6, -9<=k<=10, -47<=l<=48	
Reflections collected	8768	
Independent reflections	3473 [R(int) = 0.0280]	
Completeness to theta = 25.242 °	99.0 %	
Absorption correction	Semi-empirical from equivalents	
Max. and min. transmission	0.7456 and 0.3925	
Refinement method	Full-matrix least-squares on F^2	
Data / restraints / parameters	3473 / 0 / 223	
Goodness-of-fit on F^2	1.038	
Final R indices [I>2sigma(I)]	R1 = 0.0436, wR2 = 0.1030	
R indices (all data)	R1 = 0.0717, wR2 = 0.1154	
Absolute structure parameter	0.021(8)	
Extinction coefficient	0.011(4)	
Largest diff. peak and hole	0.466 and -0.508 e.$Å^{-3}$	

Determination of the absolute configuration of 3ri

Absolute configuration of **3ri** was determined by its transformation to **15** as shown below.

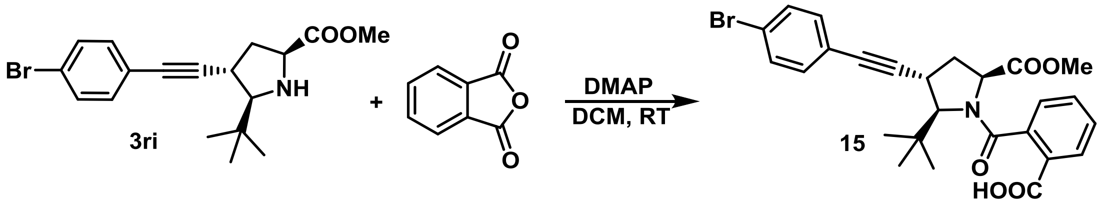

Supplementary Fig. 5. Preparation of **15** from product **3ri**

A 25 ml flask equipped with a magnetic stirring bar was charged with **3ri** (36.4 mg, 0.1 mmol, 1.0 equiv, 89% ee) and DCM (1.5 ml). Then DMAP (1.2 mg, 0.01 mmol, 0.1 equiv) and phthalic anhydride (22.2 mg, 0.15 mmol, 1.5 equiv) were added. The resulting reaction mixture was stirred

at room temperature for 12 h. After that, the reaction mixture was directly purified by silica gel column chromatography to give the product **15** (41.5 mg, white solid, 81% yield, 89% ee).

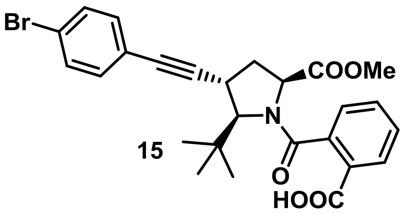

15

^{1}H NMR (400 MHz, $CDCl_3$) δ 7.74 (d, J = 7.7 Hz, 1H), 7.58 (t, J = 7.3 Hz, 1H), 7.47–7.33 (m, 4H), 7.29 (d, J = 8.4 Hz, 2H), 4.62 (d, J = 5.3 Hz, 1H), 4.31 (dd, J = 7.7, 4.8 Hz, 1H), 3.53 (s, 3H), 3.28 (q, J = 7.1 Hz, 1H), 2.61–2.47 (m, 1H), 2.36–2.18 (br, 1H), 1.09 (s, 9H) ppm.

^{13}C NMR (101 MHz, $CDCl_3$) δ 173.56, 171.77, 169.78, 139.29, 135.98, 133.16, 132.88, 131.43, 130.78, 128.94, 125.68, 122.53, 121.96, 92.57, 80.69, 72.43, 62.47, 52.10, 36.63, 35.77, 31.37, 27.46 ppm.

HRMS (ESI) m/z [M-H]$^{-}$: calcd $C_{26}H_{25}BrNO_5^{-}$ 510.0922; found 510.0922.

IR (film): ν_{max} (cm^{-1}) 2957, 1738, 1651, 1596, 1504, 1445, 1068, 823, 523.

Optical rotation: $[\alpha]_D^{25}$ = 41.75 (c = 0.51, $CHCl_3$, 89% ee).

A solution of **15** in PE/EA in a 25 ml flask was left at room temperature to grow single crystals. The absolute configuration of **15** was determined by X-ray crystallographic analysis. Products (**3ri** and **3pa-3sa**) were deduced by analogy.

CCDC 2111662 contains the supplementary crystallographic data of **15**.

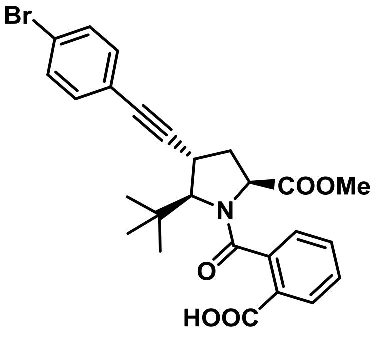

15
CCDC : 2111662

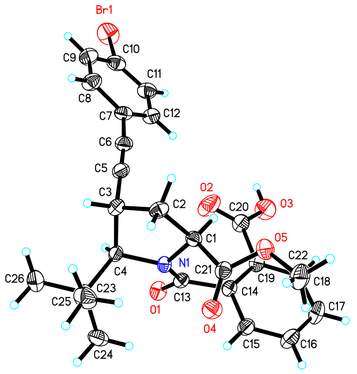

Supplementary Fig. 6. X-ray structure of **15** (CCDC 2111662, 30% ellipsoid contour probability)

Identification code	mo_d8v21739_0m	
Empirical formula	C26 H26 Br N O5	
Formula weight	512.39	
Temperature	293(2) K	
Wavelength	0.71073 Å	
Crystal system	Orthorhombic	
Space group	P 21 21 21	
Unit cell dimensions	a = 9.8009(4) Å	α= 90 °.
	b = 11.3882(4) Å	β= 90 °.

	c = 21.9917(8) Å	γ = 90°.
Volume	2454.60(16) $Å^3$	
Z	4	
Density (calculated)	1.387 Mg/m^3	
Absorption coefficient	1.711 mm^{-1}	
F(000)	1056	
Crystal size	0.170 x 0.110 x 0.070 mm^3	
Theta range for data collection	2.742 to 26.000°.	
Index ranges	-12<=h<=12, -14<=k<=11, -27<=l<=24	
Reflections collected	12155	
Independent reflections	4785 [R(int) = 0.0339]	
Completeness to theta = 25.242°	99.8 %	
Absorption correction	Semi-empirical from equivalents	
Max. and min. transmission	0.7456 and 0.5532	
Refinement method	Full-matrix least-squares on F^2	
Data / restraints / parameters	4785 / 0 / 303	
Goodness-of-fit on F^2	1.025	
Final R indices [I>2sigma(I)]	R1 = 0.0416, wR2 = 0.0800	
R indices (all data)	R1 = 0.0710, wR2 = 0.0915	
Absolute structure parameter	0.009(6)	
Extinction coefficient	n/a	
Largest diff. peak and hole	0.626 and -0.579 e.$Å^{-3}$	

Determination of the absolute configuration of 7i

A solution of **7i** in PE/DCM in a 25 ml flask was left at room temperature to grow single crystals. The absolute configuration of **7i** was determined by X-ray crystallographic analysis. Other products in **Table 4** were deduced by analogy.

CCDC 2106096 contains the supplementary crystallographic data of **7i**.

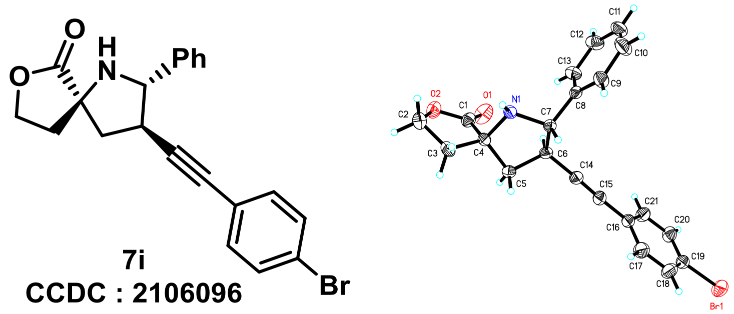

Supplementary Fig. 7. X-ray structure of **7i** (CCDC 2106096, 30% ellipsoid contour probability)

Identification code	mo_d8v21639_0m
Empirical formula	C21 H18 Br N O2

Formula weight	396.27
Temperature	293(2) K
Wavelength	0.71073 Å
Crystal system	Orthorhombic
Space group	P 21 21 21
Unit cell dimensions	a = 5.5373(2) Å α= 90°.
	b = 8.8572(3) Å β= 90°.
	c = 37.0747(12) Å γ = 90°.
Volume	1818.33(11) $Å^3$
Z	4
Density (calculated)	1.448 Mg/m^3
Absorption coefficient	2.274 mm^{-1}
F(000)	808
Crystal size	0.170 x 0.140 x 0.120 mm^3
Theta range for data collection	2.829 to 25.994°.
Index ranges	-6<=h<=6, -10<=k<=9, -45<=l<=43
Reflections collected	8847
Independent reflections	3505 [R(int) = 0.0266]
Completeness to theta = 25.242°	98.8 %
Absorption correction	Semi-empirical from equivalents
Max. and min. transmission	0.7456 and 0.3365
Refinement method	Full-matrix least-squares on F^2
Data / restraints / parameters	3505 / 0 / 231
Goodness-of-fit on F^2	1.055
Final R indices [I>2sigma(I)]	R1 = 0.0384, wR2 = 0.0950
R indices (all data)	R1 = 0.0497, wR2 = 0.1016
Absolute structure parameter	0.021(7)
Extinction coefficient	0.010(4)
Largest diff. peak and hole	0.569 and -0.550 e.$Å^{-3}$

Determination of the relative configuration of 3aq

A solution of **3aq** in PE/EA in a 25 ml flask was left at room temperature to grow single crystals. The relative configuration of **3aq** was determined by X-ray crystallographic analysis. CCDC 2111496 contains the supplementary crystallographic data of **3aq**.

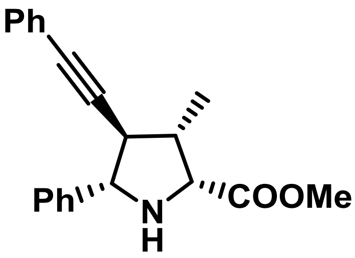

3aq
CCDC : 2111496

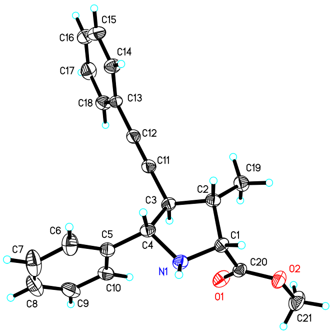

Supplementary Fig. 8. X-ray structure of **3aq** (CCDC 2111496, 30% ellipsoid contour probability)

Identification code	mo_d8v21673_0m	
Empirical formula	C21 H21 N O2	
Formula weight	319.39	
Temperature	213(2) K	
Wavelength	0.71073 Å	
Crystal system	Triclinic	
Space group	P 1	
Unit cell dimensions	a = 5.3673(5) Å	α= 100.874(3) °.
	b = 8.6715(9) Å	β= 96.733(3) °.
	c = 9.8581(10) Å	γ = 97.678(3) °.
Volume	441.73(8) Å^3	
Z	1	
Density (calculated)	1.201 Mg/m^3	
Absorption coefficient	0.077 mm^{-1}	
F(000)	170	
Crystal size	0.150 x 0.120 x 0.070 mm^3	
Theta range for data collection	2.873 to 25.995 °.	
Index ranges	-6<=h<=6, -10<=k<=10, -12<=l<=12	
Reflections collected	10815	
Independent reflections	3425 [R(int) = 0.0491]	
Completeness to theta = 25.242 °	99.8 %	
Absorption correction	Semi-empirical from equivalents	
Max. and min. transmission	0.7456 and 0.6849	
Refinement method	Full-matrix least-squares on F^2	
Data / restraints / parameters	3425 / 3 / 224	
Goodness-of-fit on F^2	1.096	
Final R indices [I>2sigma(I)]	R1 = 0.0456, wR2 = 0.0944	
R indices (all data)	R1 = 0.0697, wR2 = 0.1070	
Absolute structure parameter	0.2(8)	

Extinction coefficient	0.077(12)
Largest diff. peak and hole	0.183 and -0.152 e.Å^{-3}

2.3 Transformations of the Product

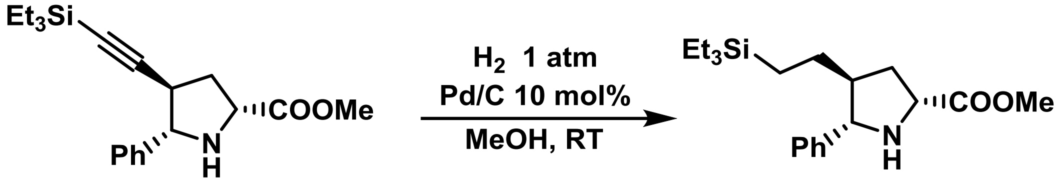

3ar **8**

Supplementary Fig. 9. Preparation of **8** from product **3ar**

A 25 ml flask equipped with a magnetic stirring bar was charged with **3ar** (34.3 mg, 0.1 mmol, 1.0 equiv, 99% ee, >20:1 dr), Pd/C (3.4 mg, 10%), and MeOH (1.0 ml). The resulting reaction mixture was stirred at room temperature under H_2 atmosphere (1 atm) for 17 hours. After that, the reaction mixture was directly purified by silica gel column chromatography to give the product **8** (30.2 mg, colorless oil, 87% yield).

^{1}H NMR (400 MHz, $CDCl_3$) δ 7.45–7.40 (m, 2H), 7.37–7.30 (m, 2H), 7.28–7.22 (m, 1H), 3.94 (dd, *J* = 8.5, 3.9 Hz, 1H), 3.78 (s, 3H), 3.71 (d, *J* = 8.2 Hz, 1H), 2.67–2.55 (br, 1H), 2.40–2.26 (m, 1H), 1.99–1.84 (m, 2H), 1.41 (tt, *J* = 13.3, 4.5 Hz, 1H), 1.11–1.23 (m, 1H), 0.88–0.83 (m, 8H), 0.54–0.31 (m, 9H) ppm.

^{13}C NMR (126 MHz, $CDCl_3$) δ 176.00, 142.52, 128.48, 127.37, 127.34, 69.25, 58.68, 52.18, 49.91, 36.54, 25.77, 9.45, 7.34, 3.08 ppm.

HRMS (ESI) m/z [M+H]$^+$: calcd $C_{20}H_{34}NO_2Si^+$ 348.2353; found 348.2353.

IR (film): ν_{max} (cm^{-1}) 3357, 3027, 2951, 1736, 1602, 1493, 1455, 1206, 1016, 754, 731, 701.

Optical rotation: $[\alpha]_D^{25}$ = 31.77 (*c* = 0.91, $CHCl_3$, 99% ee).

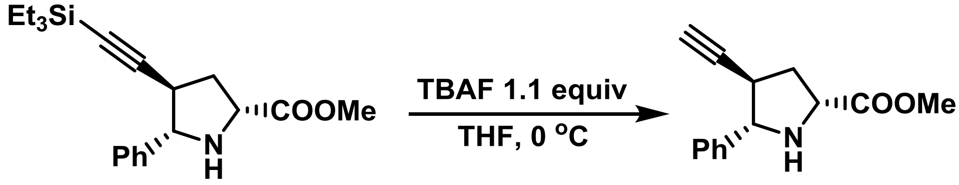

3ar **9**

Supplementary Fig. 10. Preparation of **9** from product **3ar**

A 25 ml flask equipped with a magnetic stirring bar was charged with **3ar** (94 mg, 0.274 mmol, 1.0 equiv, 99% ee, >20:1 dr) and THF (2.7 ml). The mixture was cooled to 0 °C before TBAF (1.0 M in THF, 0.3 ml, 1.1 equiv) was added via a syringe. The resulting reaction mixture was stirred at 0 °C for 15 min. After that, the reaction mixture was directly purified by silica gel column chromatography to give the product **9** (59.1 mg, colorless oil, 94% yield).

^{1}H NMR (400 MHz, $CDCl_3$) δ 7.51 (dd, *J* = 7.6, 1.7 Hz, 2H), 7.407.33 (m, 2H), 7.33–7.27 (m, 1H), 4.14 (d, *J* = 9.1 Hz, 1H), 4.03 (dd, *J* = 9.4, 4.4 Hz, 1H), 3.78 (s, 3H), 2.79–2.67 (m, 1H), 2.48 (ddd, *J* = 12.5, 7.9, 4.4 Hz, 1H), 2.35 (dt, *J* = 13.1, 9.6 Hz, 1H), 2.13 (d, *J* = 2.3 Hz, 1H) ppm.

^{13}C NMR (126 MHz, $CDCl_3$) δ 174.92, 140.54, 128.53, 127.86, 126.85, 83.60, 70.86, 69.33, 58.65, 52.34, 37.95, 37.32 ppm.

HRMS (ESI) m/z [M+H]$^+$: calcd $C_{14}H_{16}NO_2^+$ 230.1176; found 230.1177.

IR (film): ν_{max} (cm^{-1}) 3288, 3030, 2952, 1738, 1060, 1494, 1454, 1208, 1023, 757, 700

Optical rotation: $[\alpha]_D^{25}$ = 70.61 (*c* = 0.40, $CHCl_3$, 99% ee).

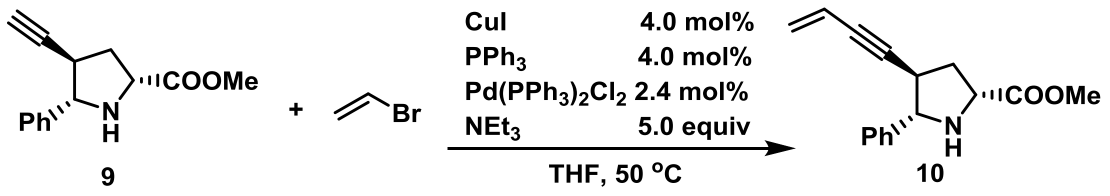

Supplementary Fig. 11. Preparation of **10** from product **9**

A 25 ml schlenk tube equipped with a magnetic stirring bar was charged with PPh_3 (1 mg, 0.0039 mmol, 0.04 equiv), CuI (0.7 mg, 0.0038 mmol, 0.04 equiv), and $Pd(PPh_3)_2Cl_2$ (1.7 mg, 0.0023 mmol, 0.024 equiv) in a glove box under Ar atmosphere. Vinyl bromide (1M in THF, 194 µl, 2.0 equiv), anhydrous THF (0.25 mL), and NEt_3 (49 mg, 0.485 mmol, 5.0 equiv) were added via syringes sequentially. The resulting mixture was stirred at room temperature. To Another dried schlenk tube was added **9** (22.3 mg, 0.097 mmol, 1.0 equiv) under N_2 atmosphere and the above reaction mixture was added via a syringe. The mixture was heated at 50 °C for 12 hours. After that, the reaction mixture was directly purified by silica gel column chromatography to give the product **10** (22.6 mg, colorless oil, 91% yield).

^{1}H NMR (400 MHz, $CDCl_3$) δ 7.55–7.46 (m, 2H), 7.40–7.33 (m, 2H), 7.32–7.27 (m, 1H), 5.75 (ddd, *J* = 17.6, 11.0, 1.9 Hz, 1H), 5.54 (dd, *J* = 17.5, 2.2 Hz, 1H), 5.39 (dd, *J* = 11.0, 2.3 Hz, 1H), 4.14 (d, *J* = 9.0 Hz, 1H), 4.04 (dd, *J* = 9.3, 4.5 Hz, 1H), 3.79 (s, 3H), 2.93–2.74 (m, 1H), 2.47 (ddd, *J* = 12.6, 7.9, 4.5 Hz, 1H), 2.35 (dt, *J* = 13.0, 9.5 Hz, 1H) ppm.

^{13}C NMR (126 MHz, $CDCl_3$) δ 174.97, 140.71, 128.52, 127.80, 126.86, 126.39, 117.14, 89.47, 81.78, 69.32, 58.71, 52.37, 38.78, 37.46 ppm.

HRMS (ESI) m/z [M+H]$^+$: calcd $C_{16}H_{18}NO_2^+$ 256.1332; found 256.1333.

IR (film): ν_{max} (cm^{-1}) 3301, 3062, 2952, 2925, 1746, 1660, 1494, 1454, 1206, 763, 702.

Optical rotation: $[\alpha]_D^{25}$ = 150.76 (*c* = 0.40, $CHCl_3$, 99% ee).

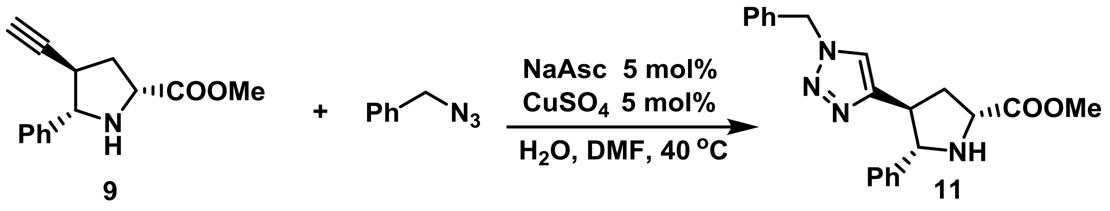

Supplementary Fig. 12. Preparation of **11** from product **9**

NaAsc (19.8 mg, 0.1 mmol, 1.0 equiv) was dissolved in 100 µl H_2O to get solution A. $CuSO_4 \cdot 5H_2O$ (1.25 mg, 0.005 mmol, 0.05 equiv) was dissolved in 100 µl H_2O to get solution B. Solution A and solution B were combined immediately to form suspension C, which was used in next step. A 25 ml flask equipped with a magnetic stirring bar was charged with **9** (22.9 mg, 0.1 mmol, 1.0 equiv), BnN_3 (13.3 mg, 0.1 mmol, 1.0 equiv), and DMF (0.5 ml). Then suspension C was added in one portion. The resulting reaction mixture was stirred at 40 °C for 3 hours and stirred at room temperature for additional 36 hours. After that, the reaction mixture was quenched by ammonia water (25-30%) and then extracted with EA, and dried over Na_2SO_4. After removal of the volatiles under reduced pressure, the crude was purified by silica gel column chromatography to give the product **11** (25.7 mg, colorless oil, 71% yield).

^{1}H NMR (400 MHz, $CDCl_3$) δ 7.38–7.33 (m, 5H), 7.30–7.22 (m, 3H), 7.21–7.15 (m, 2H), 6.95 (s, 1H), 5.43 (s, 2H), 4.30 (d, *J* = 9.4 Hz, 1H), 4.14 (dd, *J* = 9.6, 3.7 Hz, 1H), 3.79 (s, 3H), 3.29 (td, *J*

= 10.0, 8.0 Hz, 1H), 2.79–2.69 (m, 1H), 2.61–2.53 (m, 1H) ppm.

^{13}C NMR (126 MHz, $CDCl_3$) δ 175.52, 147.12, 141.06, 134.72, 128.97, 128.56, 128.41, 127.75, 127.60, 127.13, 121.01, 77.26, 69.29, 58.70, 53.89, 52.27, 44.02, 36.77 ppm.

HRMS (ESI) m/z [M+H]$^+$: calcd $C_{21}H_{23}N_4O_2^+$ 363.1816; found 363.1816.

IR (film): ν_{max} (cm^{-1}) 3343, 3030, 2950, 1736, 1603, 1496, 1455, 1211, 751, 702.

Optical rotation: $[\alpha]_D^{25}$ = 53.17 (*c* = 1.0, $CHCl_3$, 99% ee).

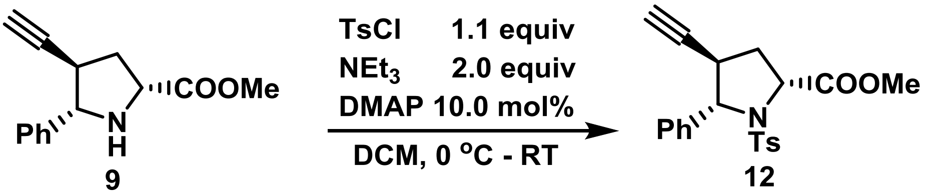

Supplementary Fig. 13. Preparation of **12** from product **9**

A 25 ml flask equipped with a magnetic stirring bar was charged with **9** (27.9 mg, 0.122 mmol, 1.0 equiv) and DCM (1.0 ml). NEt_3(24.6 mg, 0.224 mmol, 2.0 equiv) and DMAP (1.49 mg, 0.012 mmol, 0.1 equiv) were added sequentially. The mixture was cooled to 0 °C before TsCl (25.5 mg, 0.134 mmol, 1.1 equiv) was added. Then the resulting reaction mixture was stirred at room temperature for 24 hours. After that, the reaction mixture was directly purified by silica gel column chromatography to give the product **12** (33.3 mg, colorless oil, 71% yield).

^{1}H NMR (400 MHz, $CDCl_3$) δ 7.55 (d, *J* = 8.3 Hz, 2H), 7.51–7.43 (m, 2H), 7.28–7.21 (m, 3H), 7.18 (d, *J* = 8.0 Hz, 2H), 4.75–4.64 (m, 2H), 3.83 (s, 3H), 3.03–2.95 (m, 1H), 2.38 (s, 3H), 2.33 (ddd, *J* = 12.7, 6.2, 5.0 Hz, 1H), 2.17 (dt, *J* = 12.7, 8.3 Hz, 1H), 1.95 (d, *J* = 2.4 Hz, 1H) ppm.

^{13}C NMR (101 MHz, $CDCl_3$) δ 172.29, 143.57, 139.12, 135.10, 129.24, 128.26, 127.99, 127.80, 127.18, 81.29, 71.98, 70.08, 60.75, 52.62, 39.81, 35.37, 21.49 ppm.

HRMS (ESI) m/z [M+H]$^+$: calcd $C_{21}H_{22}NO_4S^+$ 384.1264; found 384.1265.

IR (film): ν_{max} (cm^{-1}) 3279, 3029, 2924, 1742, 1598, 1455, 1020, 766, 699.

Optical rotation: $[\alpha]_D^{25}$ = 48.26 (*c* = 0.36, $CHCl_3$, 99% ee).

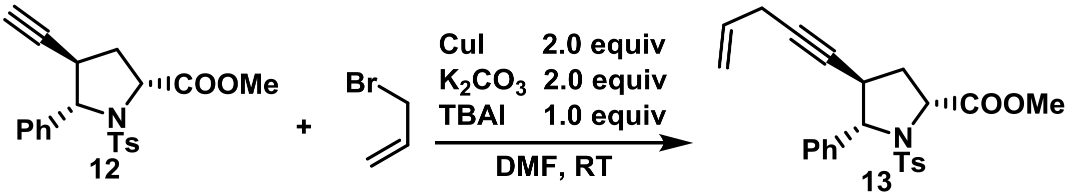

Supplementary Fig. 14. Preparation of **13** from product **12**

A 25 ml schlenk tube equipped with a magnetic stirring bar was charged with **12** (22.0 mg, 0.057 mmol, 1.0 equiv), K_2CO_3 (15.7 mg, 0.114 mmol, 2.0 equiv), TBAI (21.0 mg, 0.057 mmol, 1.0 equiv), and CuI (21.6 mg, 0,114 mmol, 2.0 equiv) under N_2 atmosphere. DMF (285 μl) and allyl bromide (10.4 mg, 0.086 mmol, 1.5 equiv) were added via syringes sequentially. The mixture was stirred at room temperature for 18 hours. After that, the reaction mixture was directly purified by silica gel column chromatography to give the product **13** (21.8 mg, pale green oil, 90% yield).

^{1}H NMR (400 MHz, $CDCl_3$) δ 7.57 (d, *J* = 8.3 Hz, 2H), 7.49 (dd, *J* = 7.8, 1.8 Hz, 2H), 7.28–7.21 (m, 3H), 7.19 (d, *J* = 7.8 Hz, 2H), 5.67 (ddt, *J* = 17.0, 10.2, 5.2 Hz, 1H), 5.15 (dq, *J* = 17.0, 1.9 Hz, 1H), 5.06 (dq, *J* = 10.0, 1.7 Hz, 1H), 4.71–4.62 (m, 2H), 3.83 (s, 3H), 3.04–2.95 (m, 1H), 2.72 (dq, *J* = 5.7, 2.0 Hz, 2H), 2.38 (s, 3H), 2.31 (dt, *J* = 12.6, 5.7 Hz, 1H), 2.14 (dt, *J* = 12.6, 7.9 Hz, 1H) ppm.

^{13}C NMR (126 MHz, $CDCl_3$) δ 172.45, 143.46, 139.61, 134.86, 132.17, 129.25, 128.18, 127.93, 127.61, 127.11, 115.99, 80.60, 79.82, 70.39, 60.87, 52.60, 40.19, 35.68, 22.80, 21.49 ppm.

HRMS (ESI) m/z [M+H]+: calcd $C_{24}H_{26}NO_4S^+$ 424.1577; found 424.1576.
IR (film): ν_{max} (cm^{-1}) 3029, 2953, 1752, 1598, 1494, 1354, 1206, 1162, 766, 701, 548.
Optical rotation: $[\alpha]_D^{25}$ = 69.87 (*c* = 1.00, $CHCl_3$, 99% ee).

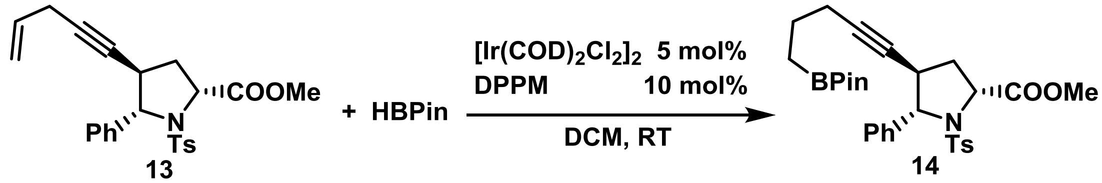

Supplementary Fig. 15. Preparation of **14** from product **13**

A 25 ml dried schlenk tube equipped with a magnetic stirring bar was charged with $[Ir(COD)_2Cl_2]_2$ (1.6 mg, 0.0024 mmol, 0.05 equiv) and DPPM (1.8 mg, 0.0048 mmol, 0.1 equiv) under N_2 atmosphere. Dried DCM (1 ml) was added via a syringe. Then the mixture was stirred at room temperature for 15 minutes. Another dried 25 ml schlenk tube was charged with **13** (20.5 mg, 0.048 mmol, 1.0 equiv), HBPin (6.8 mg, 0.053 mmol, 1.1 equiv), and the above catalyst solution. The reaction mixture was stirred at room temperature for 18 hours. After that, the mixture was directly purified by silica gel column chromatography to give the product **14** (15.9 mg, colorless oil, 60% yield).

^{1}H NMR (400 MHz, $CDCl_3$) δ 7.57 (d, *J* = 8.3 Hz, 2H), 7.49 (dd, *J* = 7.9, 1.7 Hz, 2H), 7.27–7.18 (m, 5H), 4.67–4.59 (m, 2H), 3.83 (s, 3H), 2.98–2.89 (m, 1H), 2.39 (s, 3H), 2.27 (dt, *J* = 12.6, 5.7 Hz, 1H), 2.14–2.07 (m, 1H), 1.97–1.86 (m, 2H), 1.52–1.39 (m, 2H), 1.24 (s, 12H), 0.81–0.71 (m, 2H) ppm.

^{13}C NMR (126 MHz, $CDCl_3$) δ 172.51, 143.45, 139.76, 134.84, 129.26, 128.15, 127.95, 127.53, 127.10, 84.15, 83.03, 70.51, 60.90, 52.58, 40.24, 35.80, 24.80, 23.34, 21.52, 20.98 ppm.

HRMS (ESI) m/z [M+H]+: calcd $C_{30}H_{39}BNO_6S^+$ 551.2622; found 551.2620.
IR (film): ν_{max} (cm^{-1}) 2977, 2928, 1754, 1598, 1495, 1372, 1206, 765, 701, 549.
Optical rotation: $[\alpha]_D^{25}$ = 39.37 (*c* = 0.70, $CHCl_3$, 99% ee).

3. Supplementary Figures

3.1 ^{1}H, ^{13}C, and ^{19}F NMR Spectra of New Compounds

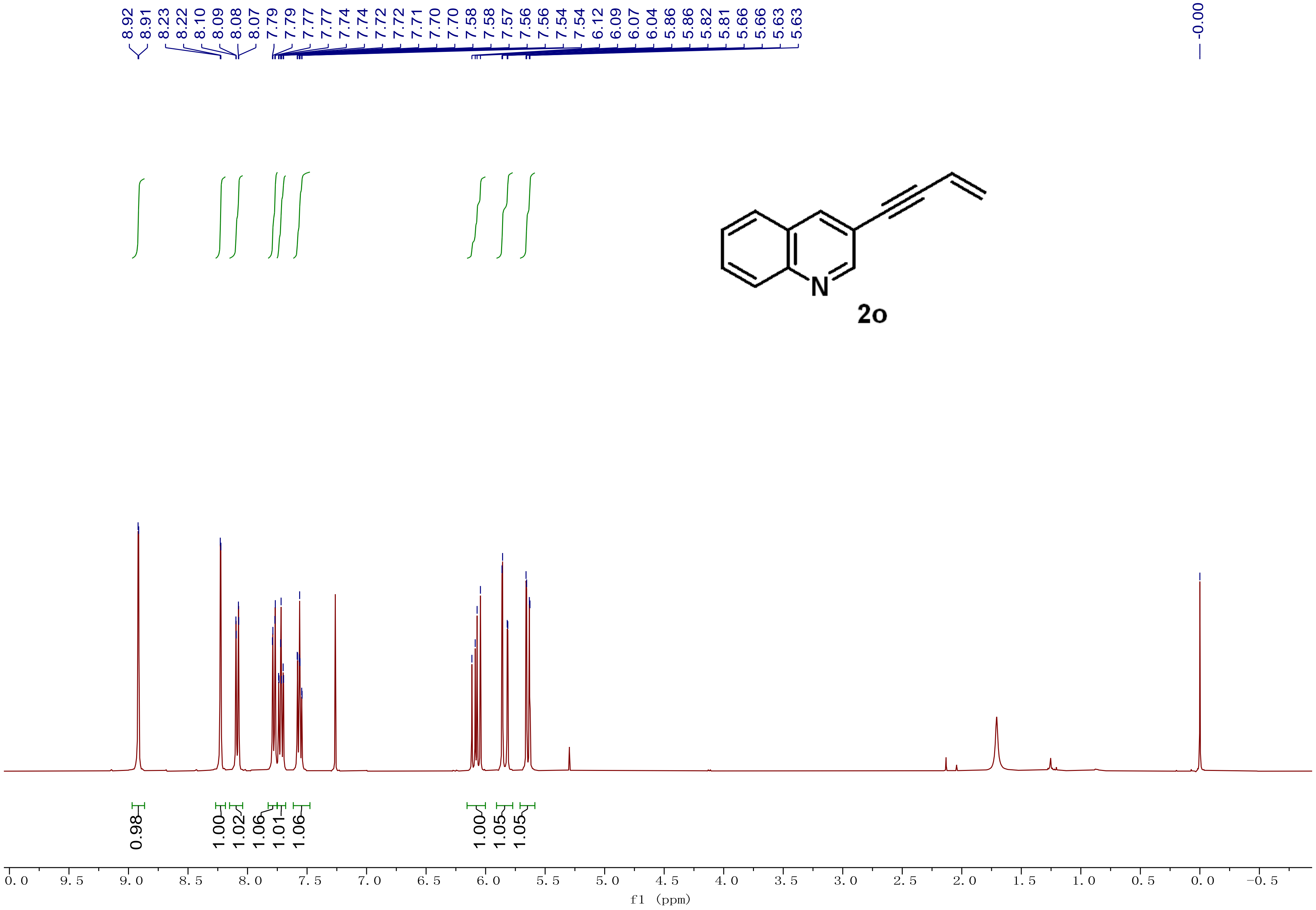
8.92
8.91
8.23
8.22
8.10
8.09
8.08
8.07
7.79
7.79
7.77
7.77
7.74
7.74
7.72
7.72
7.71
7.70
7.70
7.58
7.58
7.57
7.56
7.56
7.54
7.54
6.12
6.09
6.07
6.04
5.86
5.86
5.82
5.81
5.66
5.66
5.63
5.63
-0.00
N
2o
0.98
1.00
1.02
1.06
1.01
1.06
1.00
1.05
1.05
f1 (ppm)

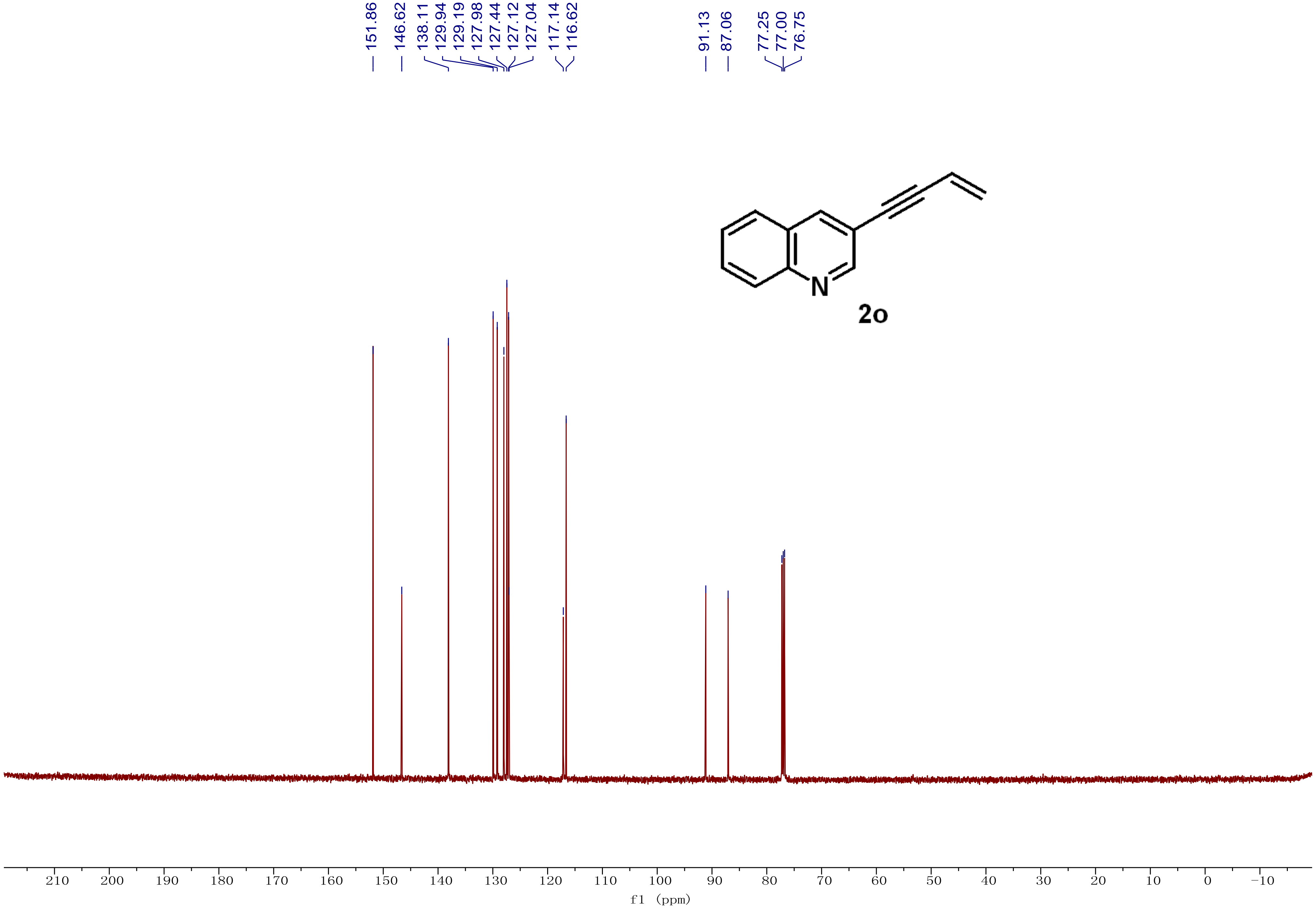
151.86
146.62
138.11
129.94
129.19
127.98
127.44
127.12
127.04
117.14
116.62
91.13
87.06
77.25
77.00
76.75
N
2o
210 200 190 180 170 160 150 140 130 120 110 100 90 80 70 60 50 40 30 20 10 0 −10
f1 (ppm)

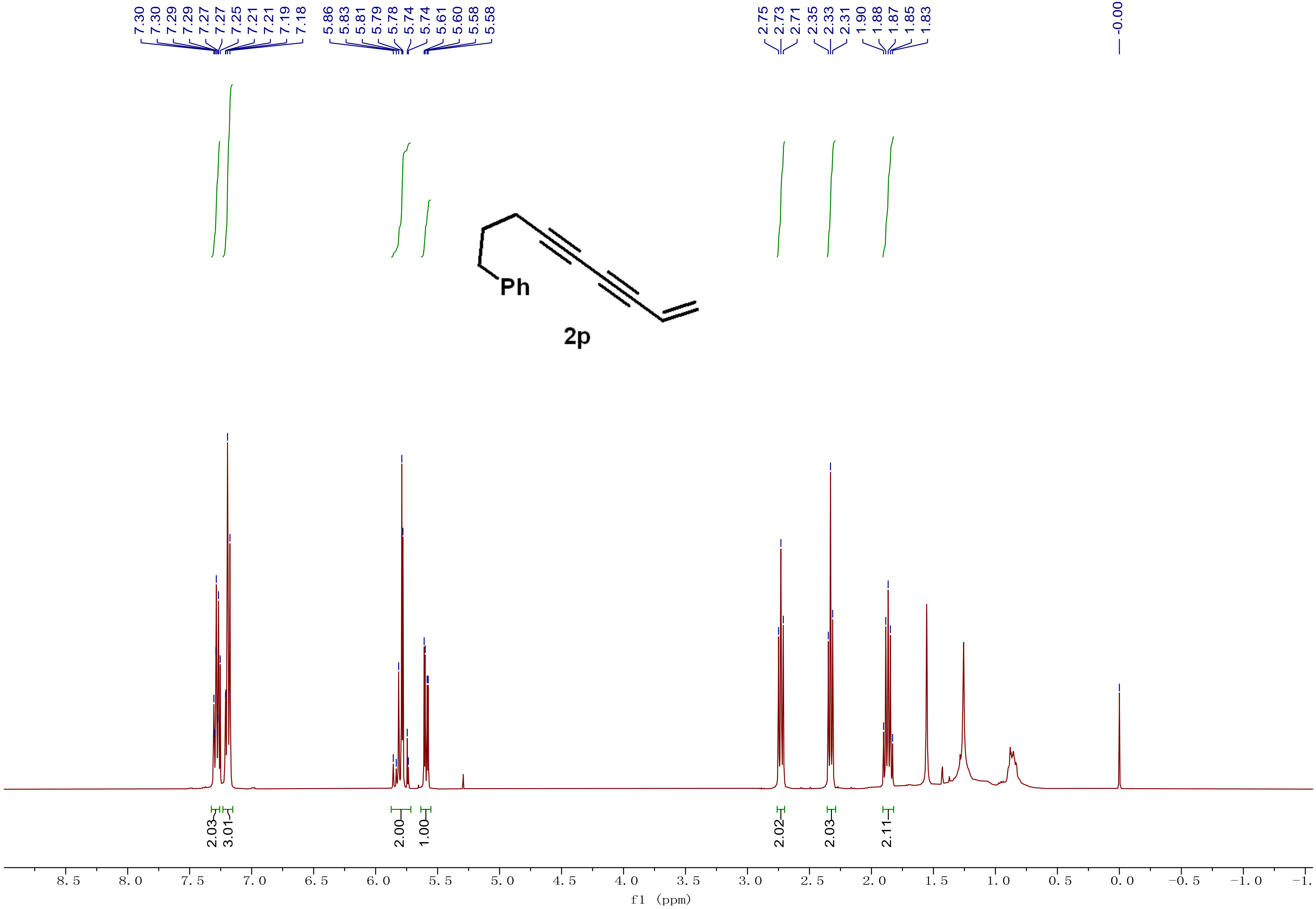

7.30 7.30 7.29 7.29 7.27 7.27 7.25 7.21 7.21 7.19 7.18
5.86 5.83 5.81 5.79 5.78 5.74 5.74 5.61 5.60 5.58 5.58
2.75 2.73 2.71 2.35 2.33 2.31 1.90 1.88 1.87 1.85 1.83
-0.00
Ph
2p
2.03 3.01 2.00 1.00 2.02 2.03 2.11
8.5 8.0 7.5 7.0 6.5 6.0 5.5 5.0 4.5 4.0 3.5 3.0 2.5 2.0 1.5 1.0 0.5 0.0 −0.5 −1.0
f1 (ppm)

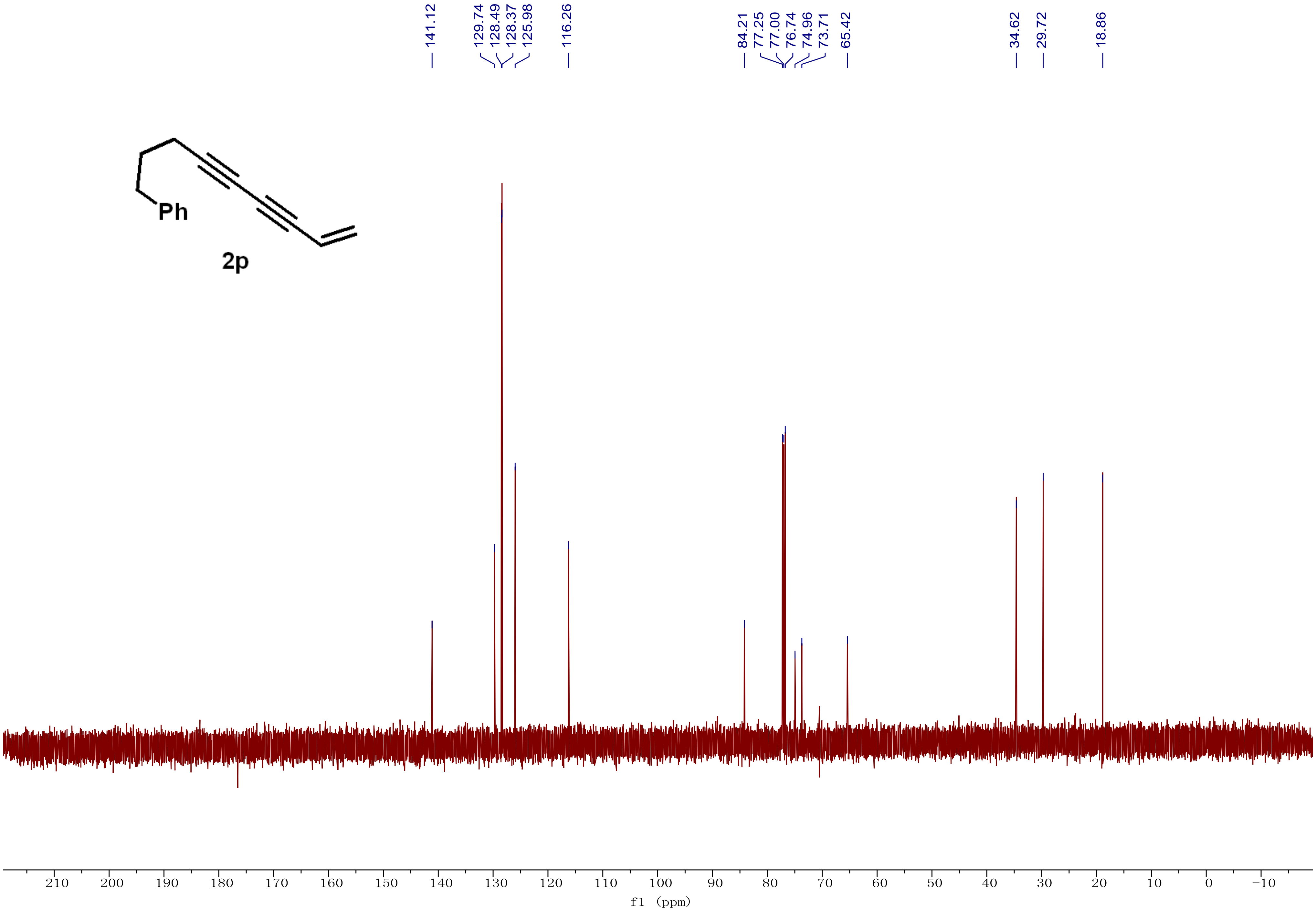
141.12
129.74
128.49
128.37
125.98
116.26
84.21
77.25
77.00
76.74
74.96
73.71
65.42
34.62
29.72
18.86
Ph
2p
210 200 190 180 170 160 150 140 130 120 110 100 90 80 70 60 50 40 30 20 10 0 −10
f1 (ppm)

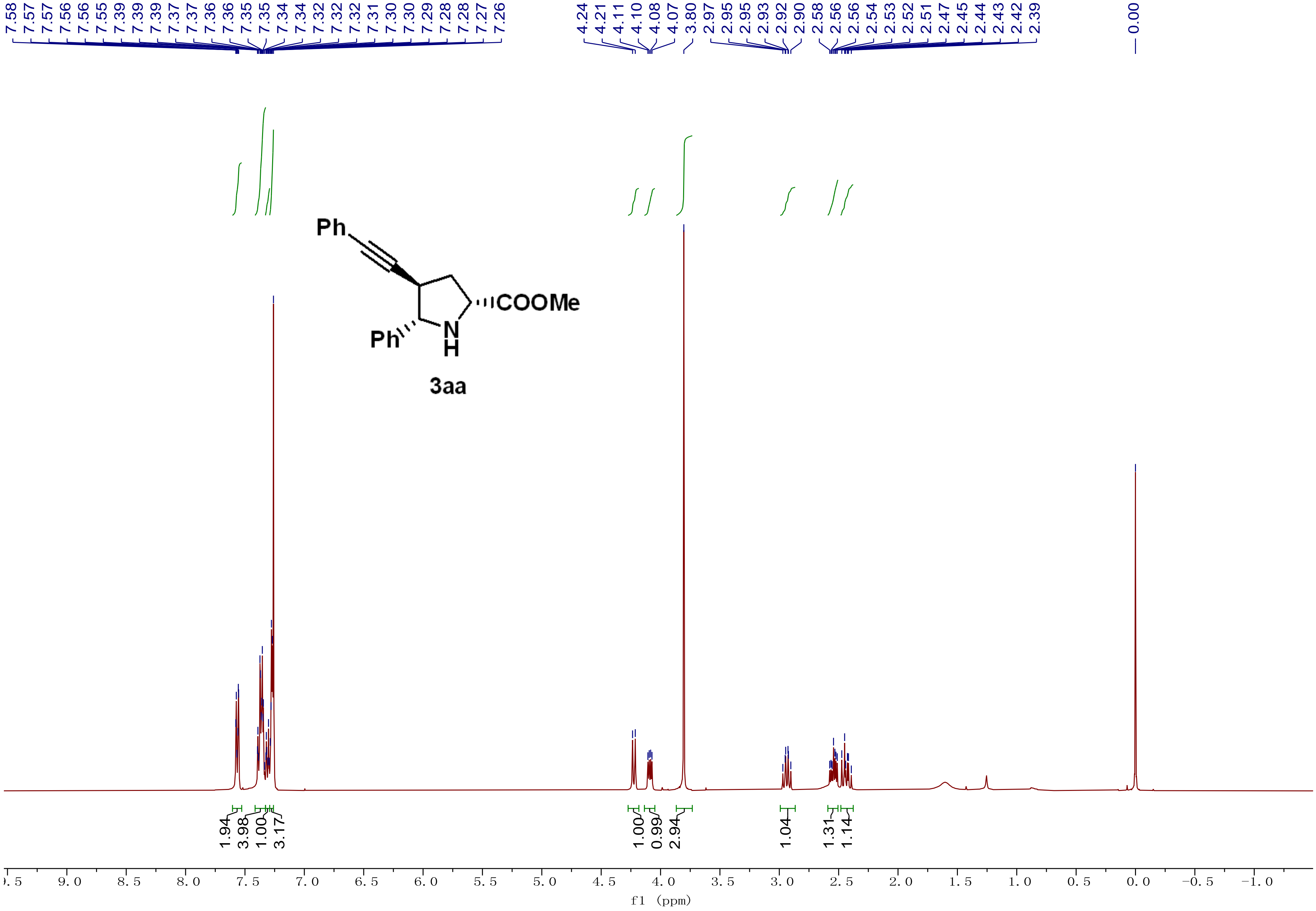
7.58 7.57 7.57 7.56 7.56 7.55 7.39 7.39 7.39 7.37 7.37 7.36 7.36 7.35 7.35 7.34 7.34 7.32 7.32 7.32 7.31 7.30 7.30 7.29 7.28 7.28 7.27 7.26
4.24 4.21 4.11 4.10 4.08 4.07 3.80 2.97 2.95 2.95 2.93 2.92 2.90 2.58 2.56 2.56 2.54 2.53 2.52 2.51 2.47 2.45 2.44 2.43 2.42 2.39
0.00
Ph
Ph
COOMe
N
H
3aa
1.94
3.98
1.00
3.17
1.00
0.99
2.94
1.04
1.31
1.14
9.0 8.5 8.0 7.5 7.0 6.5 6.0 5.5 5.0 4.5 4.0 3.5 3.0 2.5 2.0 1.5 1.0 0.5 0.0 -0.5 -1.0
f1 (ppm)

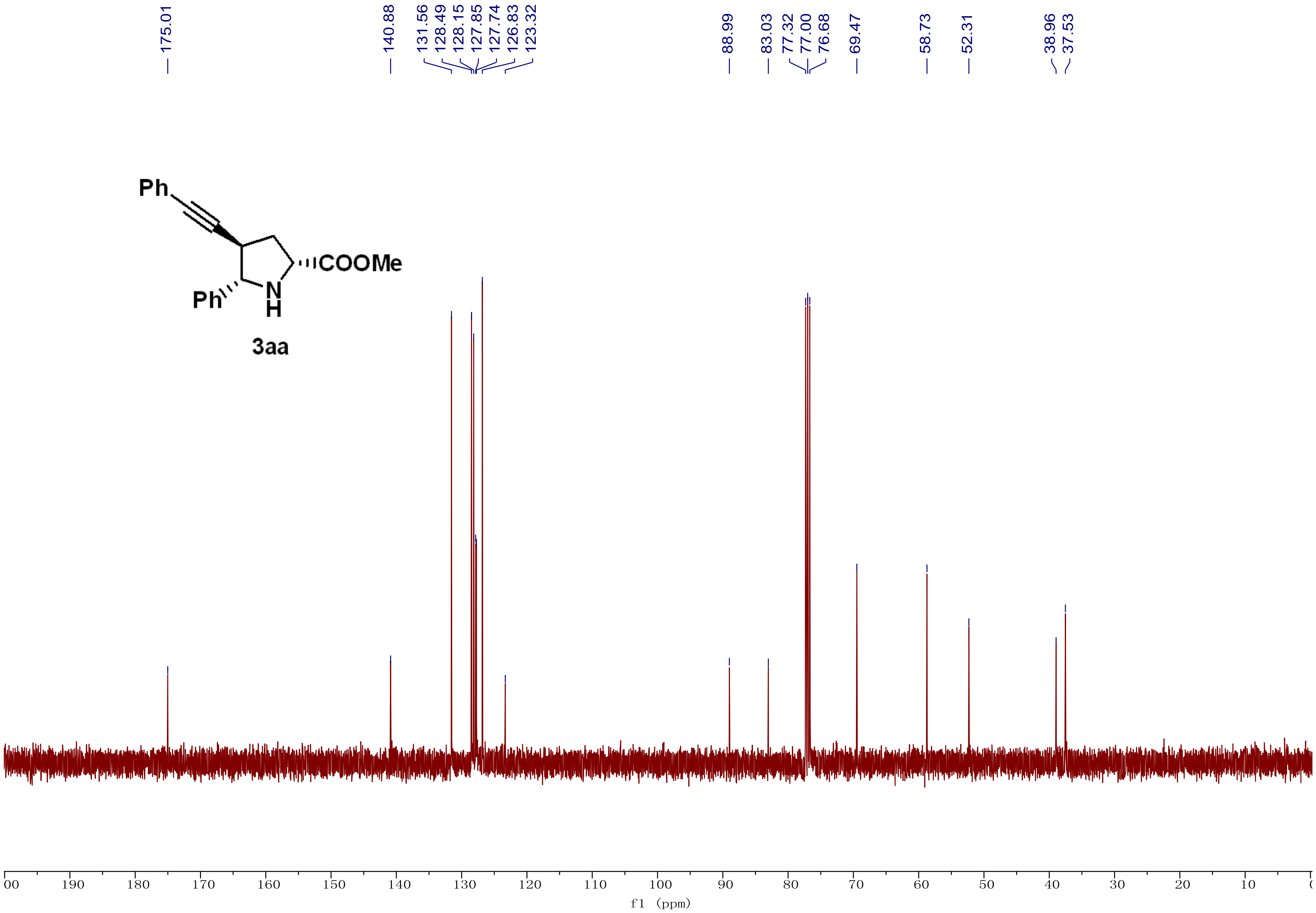

175.01
140.88
131.56
128.49
128.15
127.85
127.74
126.83
123.32
88.99
83.03
77.32
77.00
76.68
69.47
58.73
52.31
38.96
37.53
Ph
COOMe
Ph
N
H
3aa
00
190
180
170
160
150
140
130
120
110
100
90
80
70
60
50
40
30
20
10
f1 (ppm)

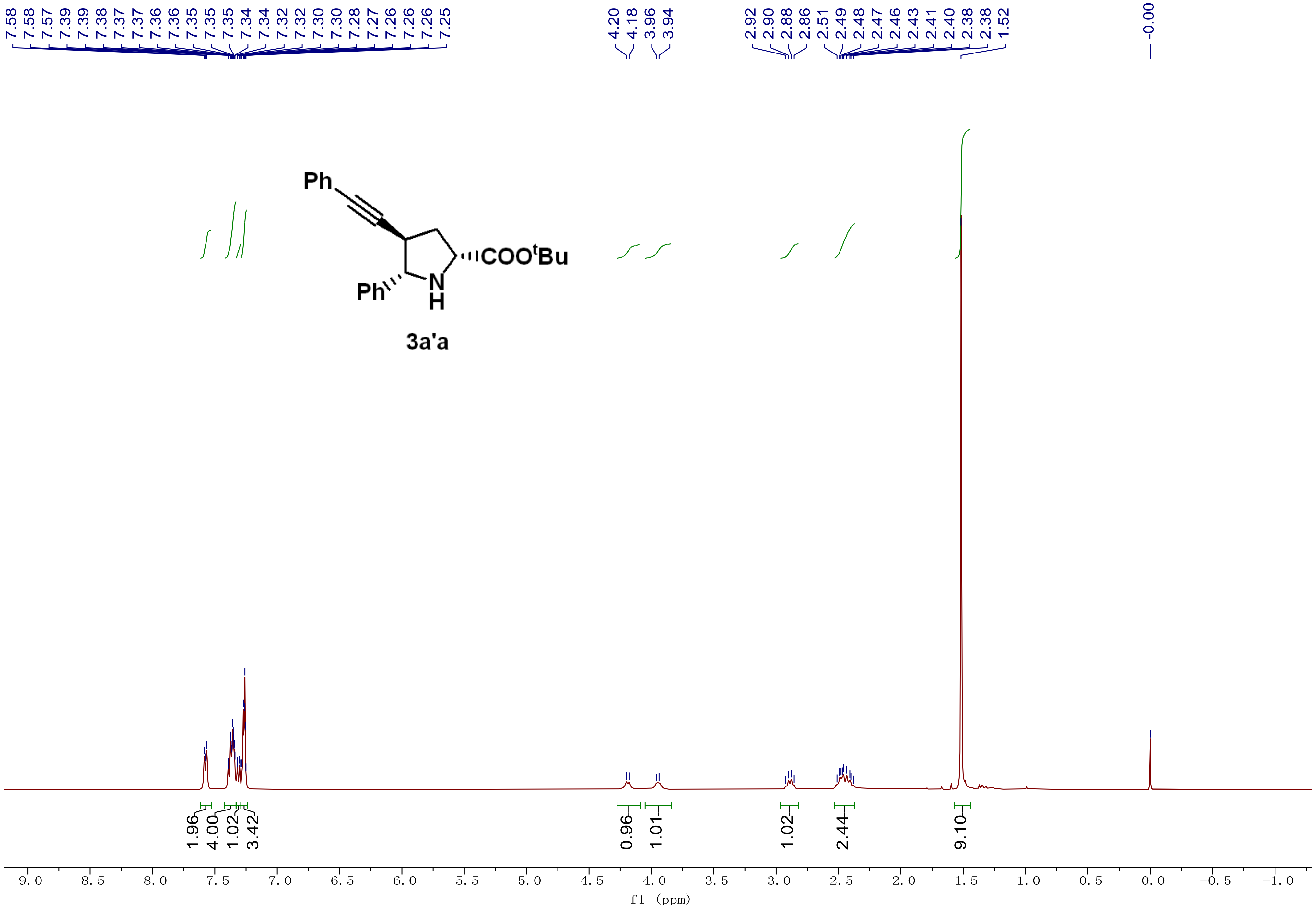
7.58 7.58 7.57 7.39 7.39 7.38 7.37 7.37 7.36 7.36 7.35 7.35 7.35 7.34 7.34 7.32 7.32 7.30 7.30 7.28 7.27 7.26 7.26 7.26 7.25
4.20 4.18 3.96 3.94
2.92 2.90 2.88 2.86 2.51 2.49 2.48 2.47 2.46 2.43 2.41 2.40 2.38 2.38 1.52
-0.00
Ph
COOtBu
Ph
N
H
3a'a
1.96 4.00 1.02 3.42
0.96 1.01
1.02 2.44
9.10
9.0 8.5 8.0 7.5 7.0 6.5 6.0 5.5 5.0 4.5 4.0 3.5 3.0 2.5 2.0 1.5 1.0 0.5 0.0 -0.5 -1.0
f1 (ppm)

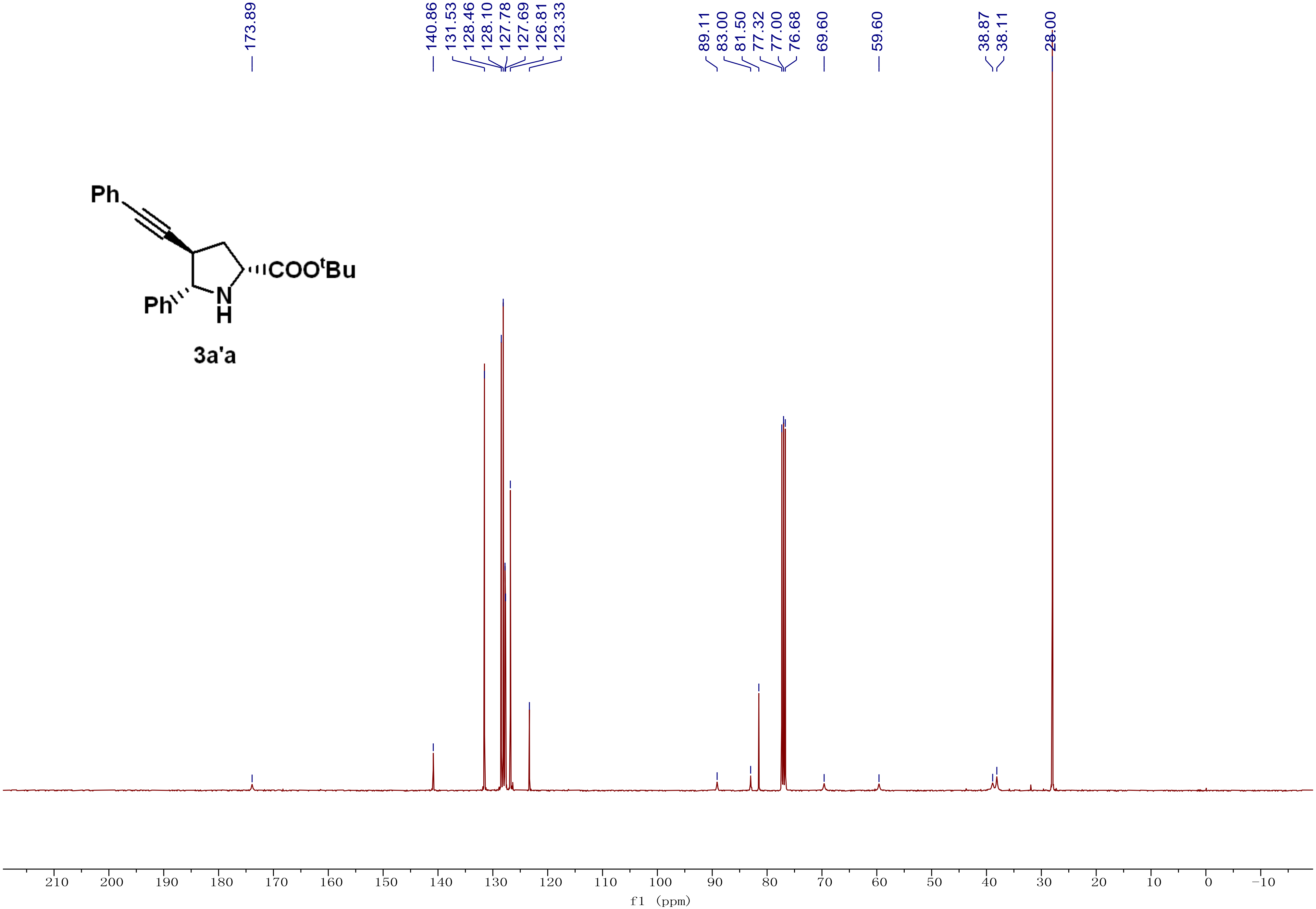
Ph
COO^{t}Bu
Ph
N
H
173.89
140.86
131.53
128.46
128.10
127.78
127.69
126.81
123.33
89.11
83.00
81.50
77.32
77.00
76.68
69.60
59.60
38.87
38.11
28.00
210 200 190 180 170 160 150 140 130 120 110 100 90 80 70 60 50 40 30 20 10 0 -10
f1 (ppm)

3a'a

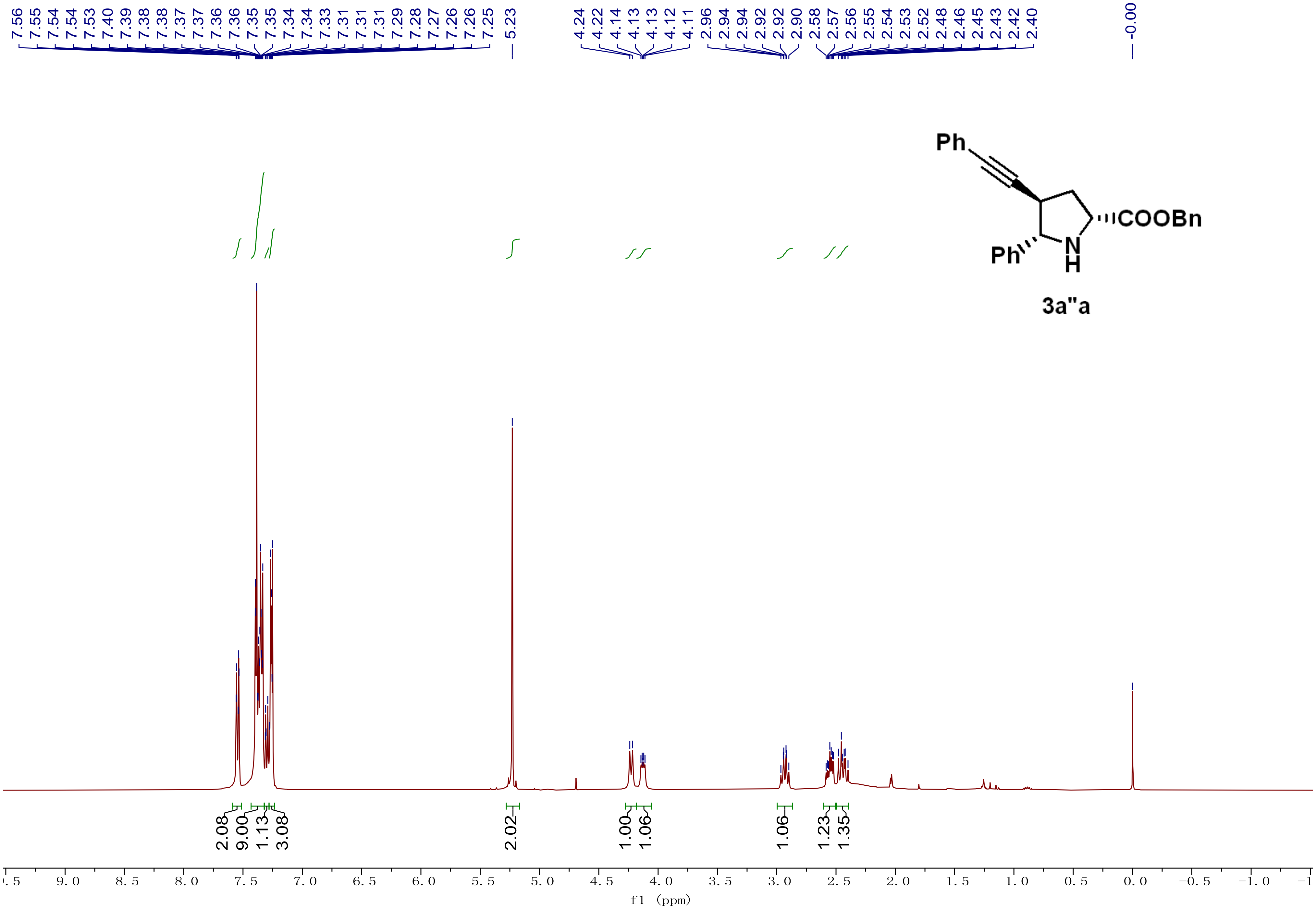
7.56 7.55 7.54 7.54 7.53 7.40 7.39 7.38 7.38 7.37 7.37 7.36 7.36 7.35 7.35 7.34 7.34 7.33 7.31 7.31 7.31 7.29 7.28 7.27 7.26 7.26 7.25
5.23
4.24 4.22 4.14 4.13 4.13 4.12 4.11 2.96 2.94 2.94 2.92 2.92 2.90 2.58 2.57 2.56 2.55 2.54 2.53 2.52 2.48 2.46 2.45 2.43 2.42 2.40
-0.00
Ph
COOBn
Ph
N
H
3a"a
2.08 9.00 1.13 3.08
2.02
1.00 1.06
1.06
1.23 1.35
f1 (ppm)

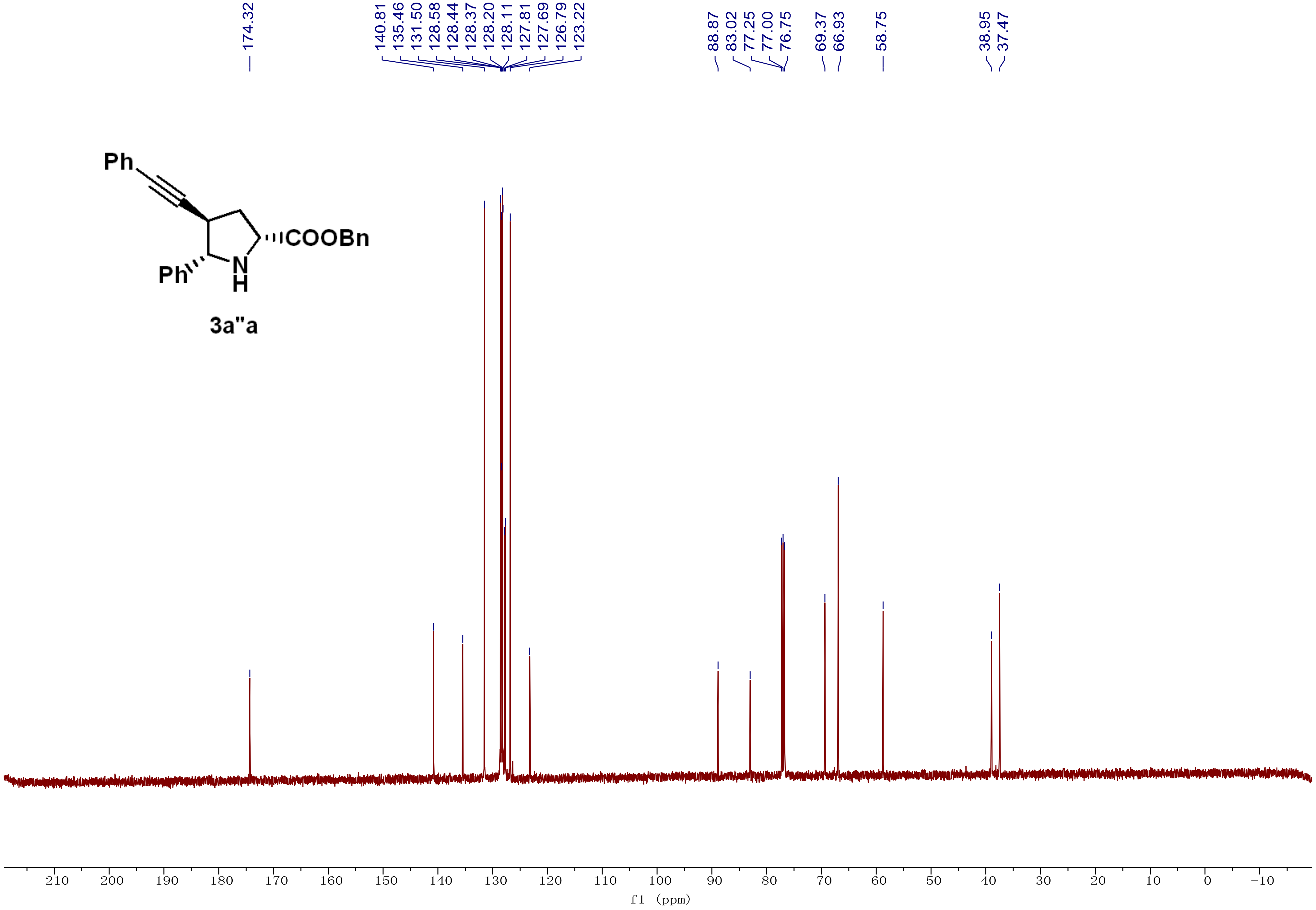
174.32
140.81
135.46
131.50
128.58
128.44
128.37
128.20
128.11
127.81
127.69
126.79
123.22
88.87
83.02
77.25
77.00
76.75
69.37
66.93
58.75
38.95
37.47
Ph
COOBn
Ph
N
H
210 200 190 180 170 160 150 140 130 120 110 100 90 80 70 60 50 40 30 20 10 0 -10
f1 (ppm)

3a"a

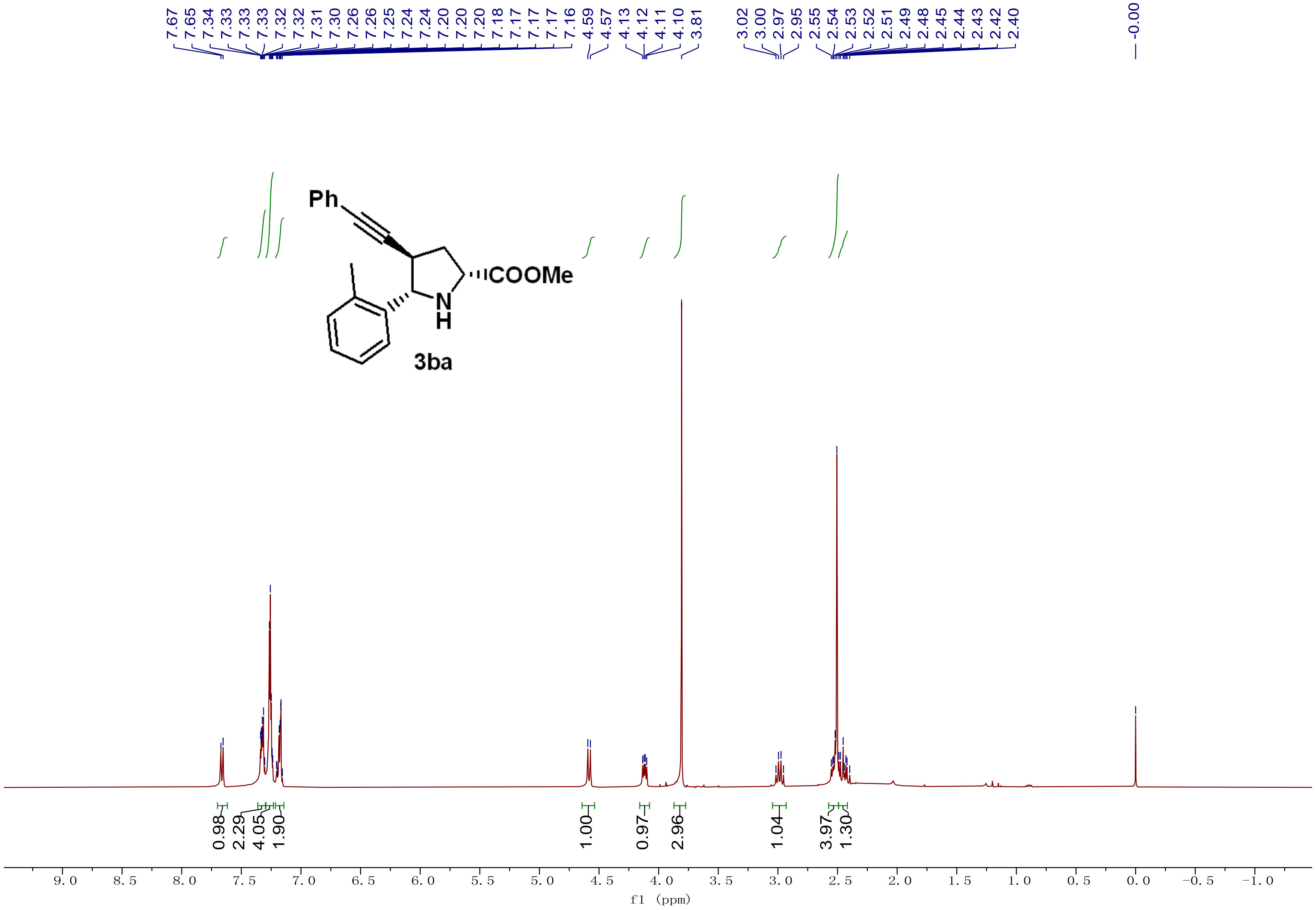
7.67 7.65 7.34 7.33 7.33 7.33 7.32 7.32 7.31 7.30 7.26 7.26 7.25 7.24 7.24 7.20 7.20 7.20 7.18 7.17 7.17 7.17 7.16 4.59 4.57 4.13 4.12 4.11 4.10 3.81
3.02 3.00 2.97 2.95 2.55 2.54 2.53 2.52 2.51 2.49 2.48 2.45 2.44 2.43 2.42 2.40
-0.00
Ph
COOMe
N
H
3ba
0.98 2.29 4.05 1.90 1.00 0.97 2.96 1.04 3.97 1.30
9.0 8.5 8.0 7.5 7.0 6.5 6.0 5.5 5.0 4.5 4.0 3.5 3.0 2.5 2.0 1.5 1.0 0.5 0.0 -0.5 -1.0
f1 (ppm)

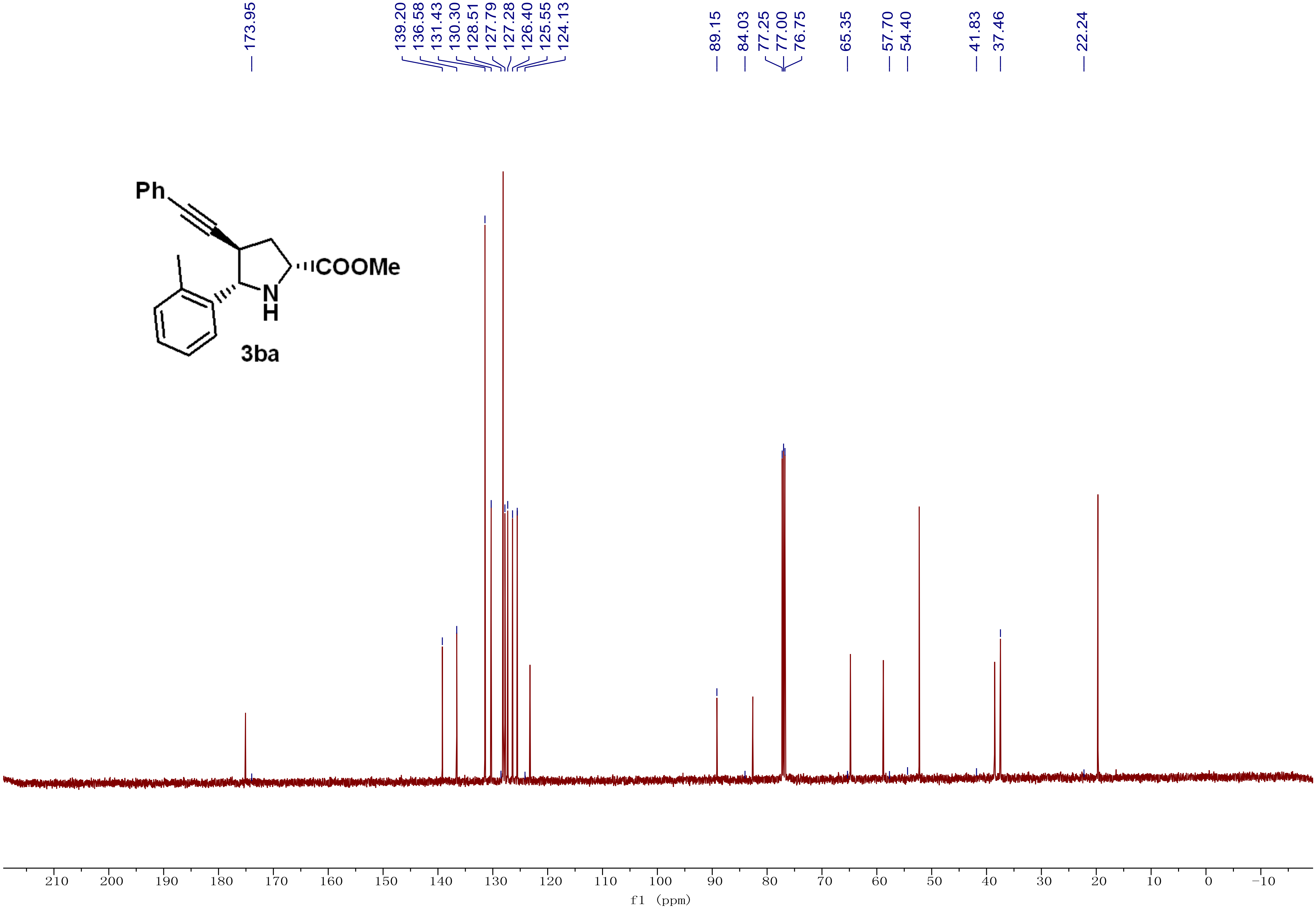
Ph
COOMe
N
H
3ba
173.95
139.20
136.58
131.43
130.30
128.51
127.79
127.28
126.40
125.55
124.13
89.15
84.03
77.25
77.00
76.75
65.35
57.70
54.40
41.83
37.46
22.24
210 200 190 180 170 160 150 140 130 120 110 100 90 80 70 60 50 40 30 20 10 0 -10
f1 (ppm)

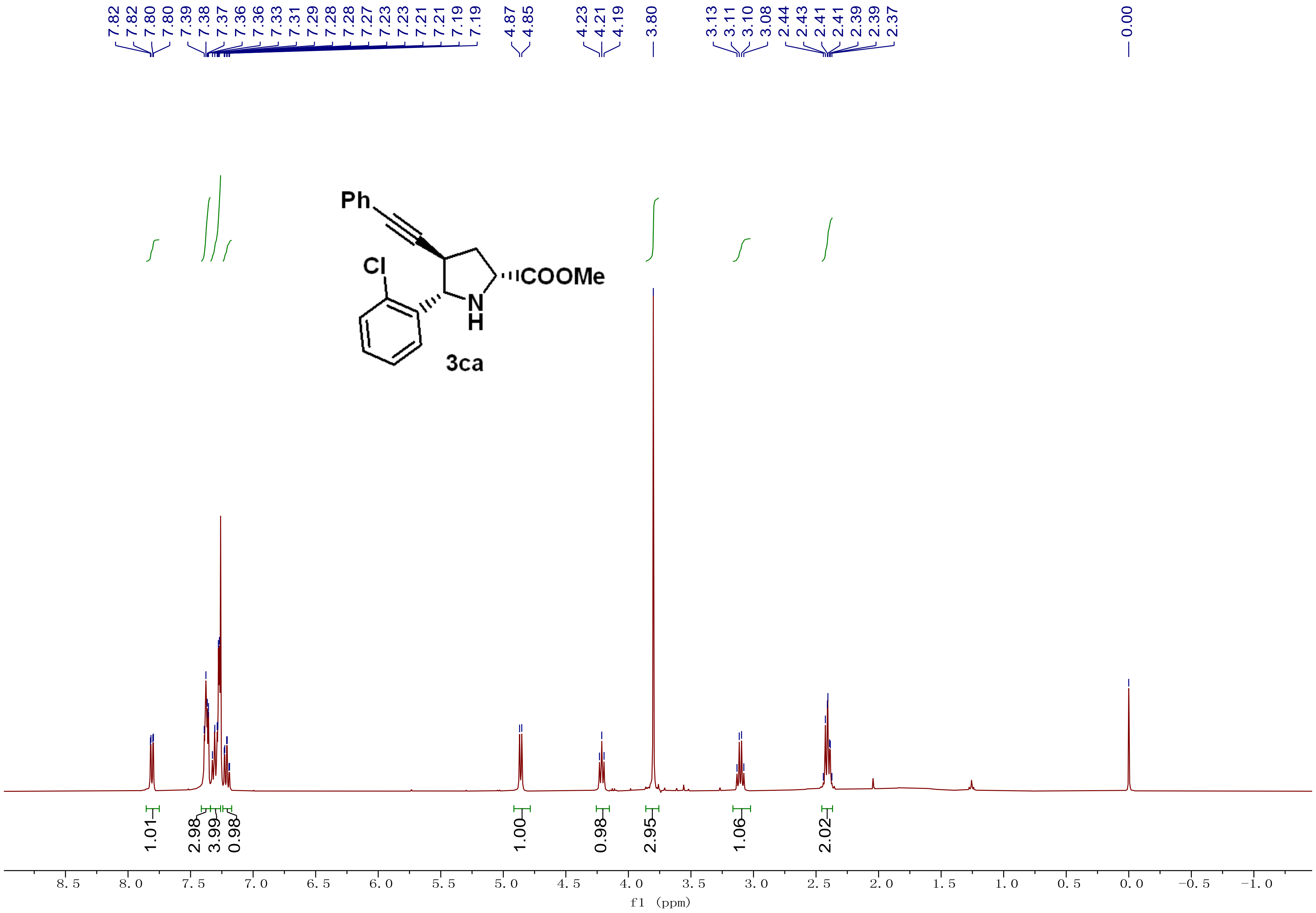
7.82 7.82 7.80 7.80 7.39 7.38 7.37 7.36 7.36 7.33 7.31 7.29 7.28 7.28 7.27 7.23 7.23 7.21 7.21 7.19 7.19
4.87 4.85
4.23 4.21 4.19
3.80
3.13 3.11 3.10 3.08
2.44 2.43 2.41 2.41 2.39 2.39 2.37
0.00
Ph
Cl
COOMe
N
H
3ca
1.01
2.98
3.99
0.98
1.00
0.98
2.95
1.06
2.02
8.5 8.0 7.5 7.0 6.5 6.0 5.5 5.0 4.5 4.0 3.5 3.0 2.5 2.0 1.5 1.0 0.5 0.0 −0.5 −1.0
f1 (ppm)

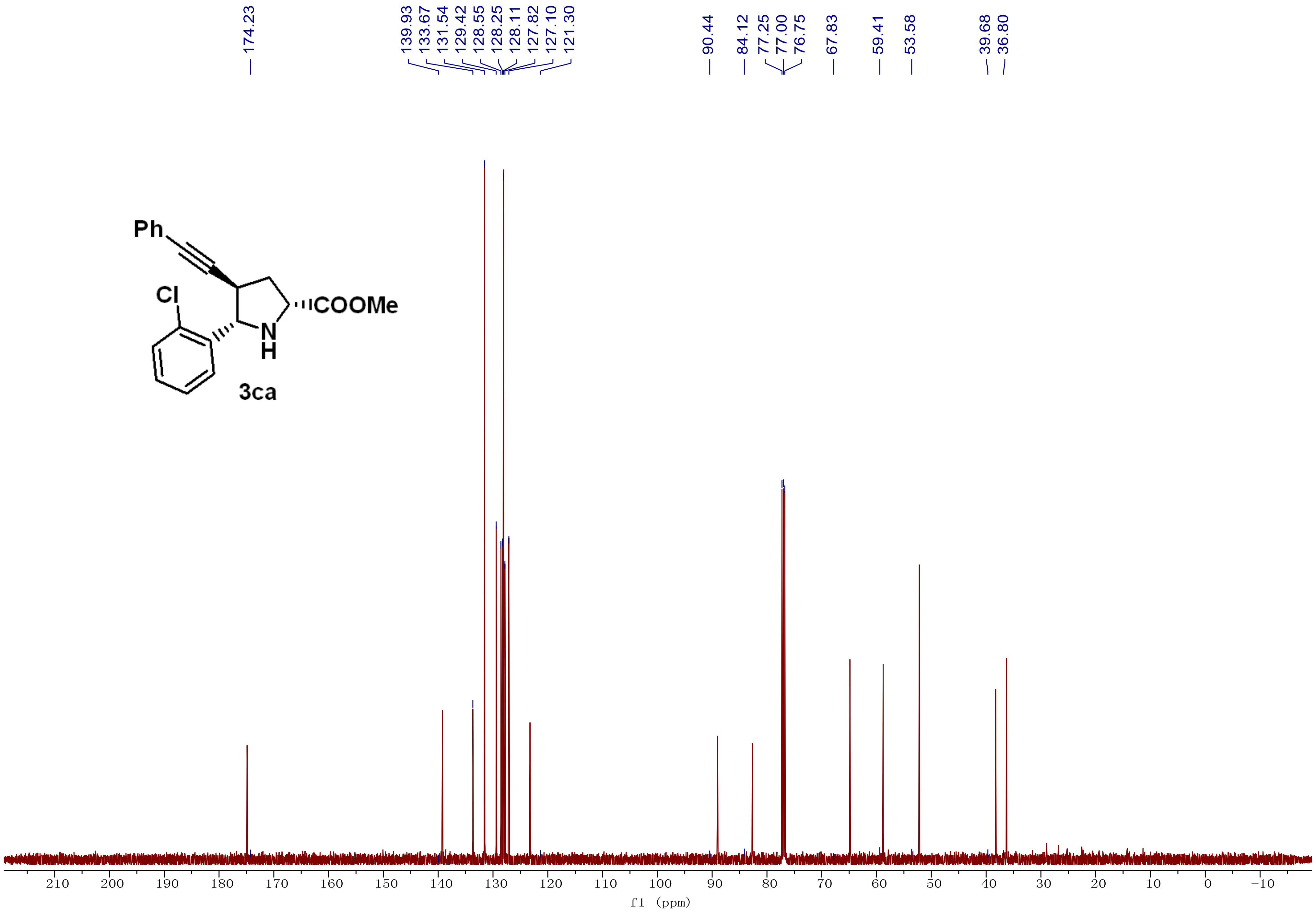
174.23
139.93
133.67
131.54
129.42
128.55
128.25
128.11
127.82
127.10
121.30
90.44
84.12
77.25
77.00
76.75
67.83
59.41
53.58
39.68
36.80
Ph
Cl
COOMe
N
H
3ca
210 200 190 180 170 160 150 140 130 120 110 100 90 80 70 60 50 40 30 20 10 0 -10
f1 (ppm)

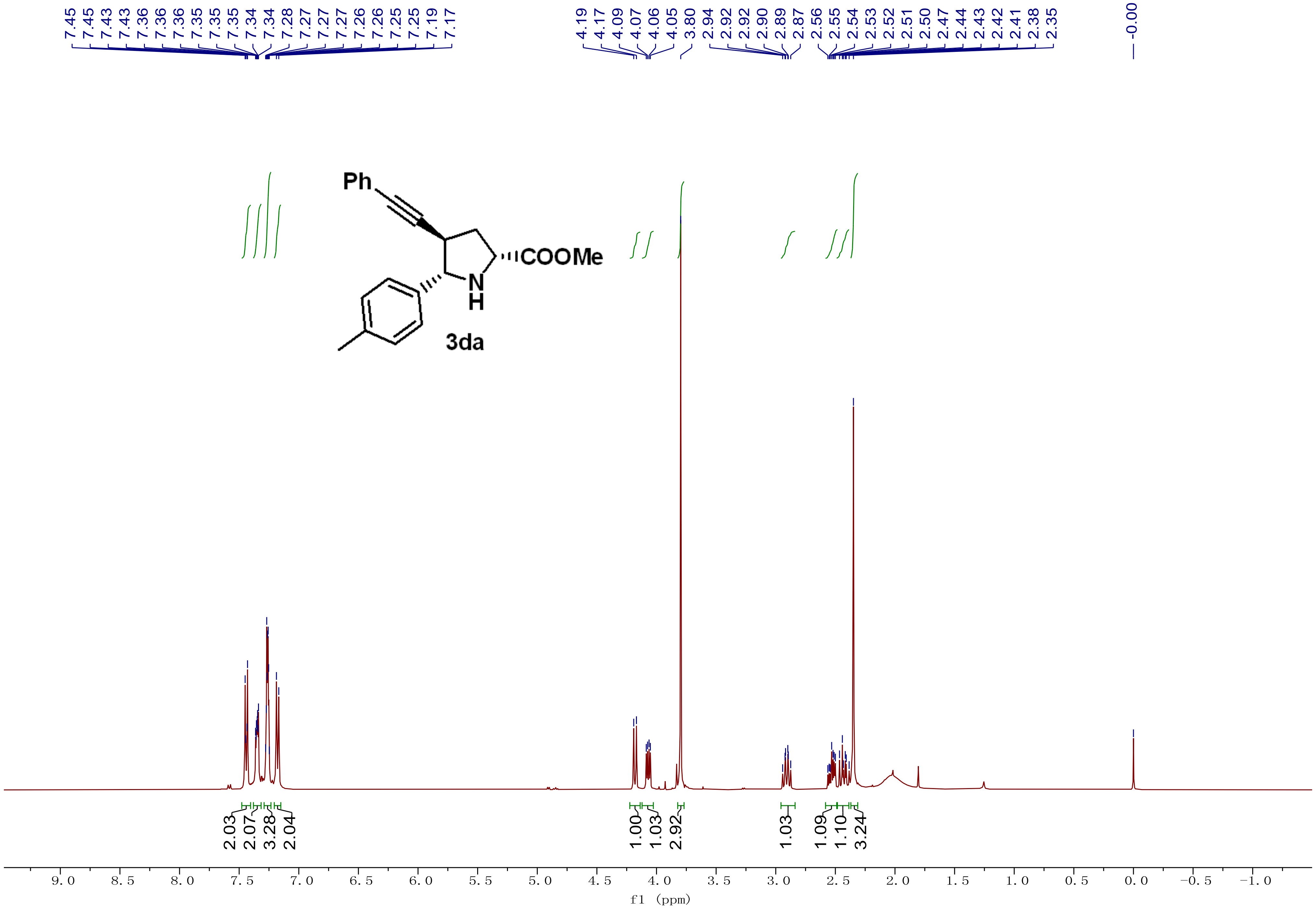
7.45 7.45 7.43 7.43 7.36 7.36 7.36 7.35 7.35 7.35 7.34 7.34 7.28 7.27 7.27 7.27 7.26 7.26 7.25 7.25 7.19 7.17
4.19 4.17 4.09 4.07 4.06 4.05 3.80 2.94 2.92 2.92 2.90 2.89 2.87 2.56 2.55 2.54 2.53 2.52 2.51 2.50 2.47 2.44 2.43 2.42 2.41 2.38 2.35
-0.00
Ph
COOMe
N
H
3da
2.03 2.07 3.28 2.04
1.00 1.03 2.92
1.03
1.09 1.10 3.24
9.0 8.5 8.0 7.5 7.0 6.5 6.0 5.5 5.0 4.5 4.0 3.5 3.0 2.5 2.0 1.5 1.0 0.5 0.0 -0.5 -1.0
f1 (ppm)

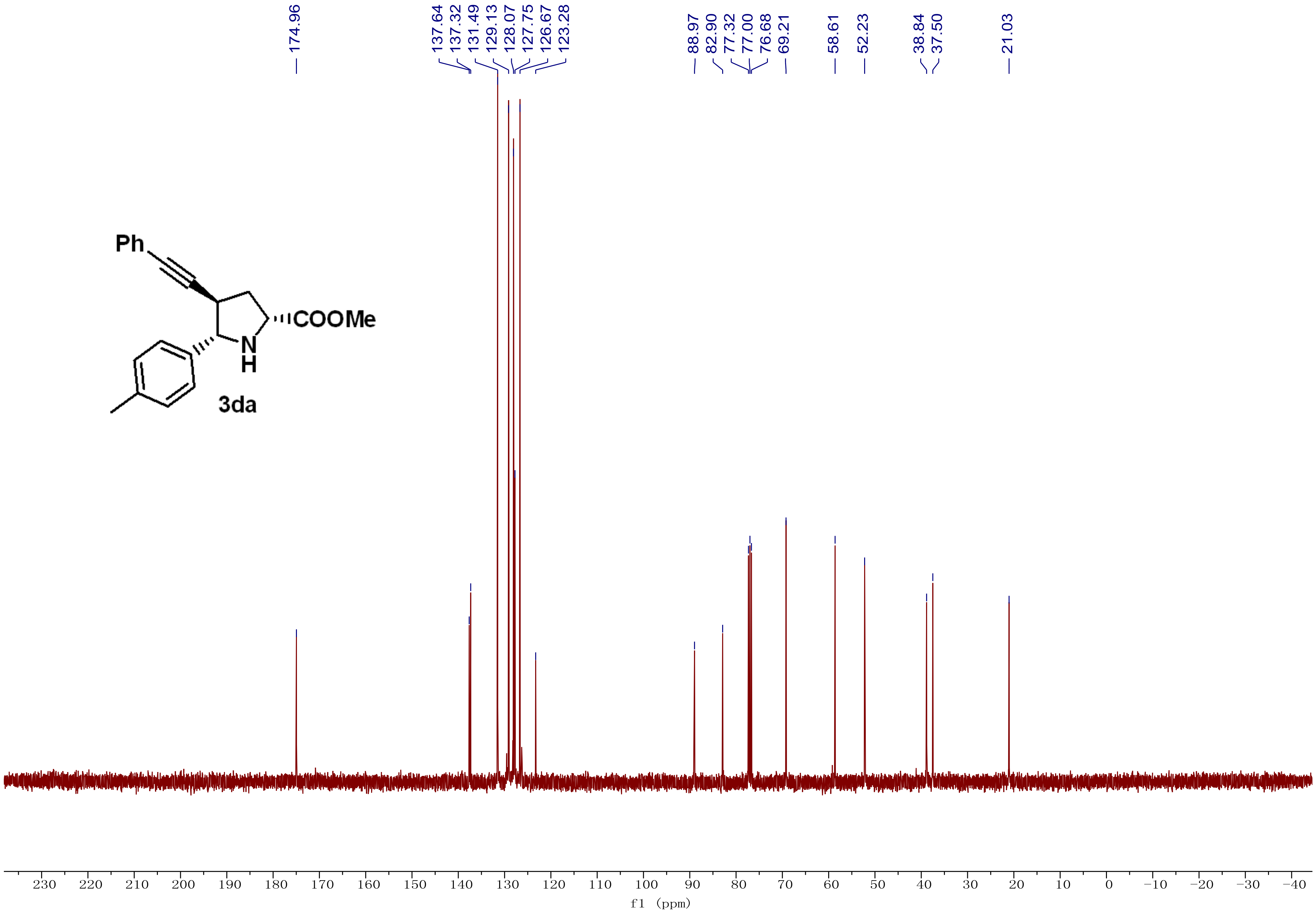
174.96
137.64
137.32
131.49
129.13
128.07
127.75
126.67
123.28
88.97
82.90
77.32
77.00
76.68
69.21
58.61
52.23
38.84
37.50
21.03
Ph
COOMe
N
H
3da
230 220 210 200 190 180 170 160 150 140 130 120 110 100 90 80 70 60 50 40 30 20 10 0 −10 −20 −30 −40
f1 (ppm)

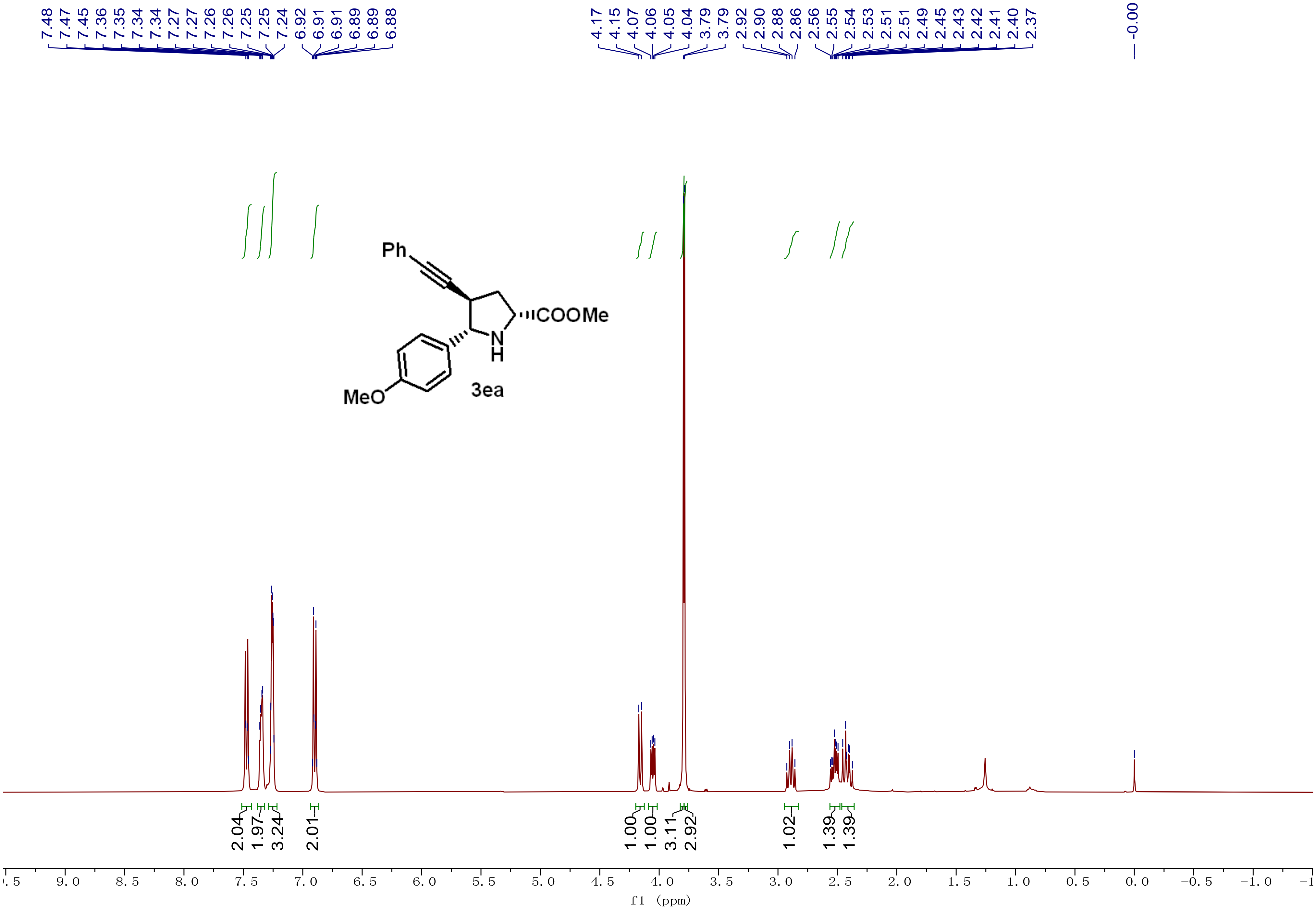
7.48 7.47 7.45 7.36 7.35 7.34 7.34 7.27 7.27 7.26 7.26 7.25 7.25 7.24 6.92 6.91 6.91 6.89 6.89 6.88
4.17 4.15 4.07 4.06 4.05 4.04 3.79 3.79 2.92 2.90 2.88 2.86 2.56 2.55 2.54 2.53 2.51 2.51 2.49 2.45 2.43 2.42 2.41 2.40 2.37
-0.00
Ph
COOMe
N
H
MeO
3ea
2.04 1.97 3.24 2.01 1.00 1.00 3.11 2.92 1.02 1.39 1.39
9.0 8.5 8.0 7.5 7.0 6.5 6.0 5.5 5.0 4.5 4.0 3.5 3.0 2.5 2.0 1.5 1.0 0.5 0.0 −0.5 −1.0
f1 (ppm)

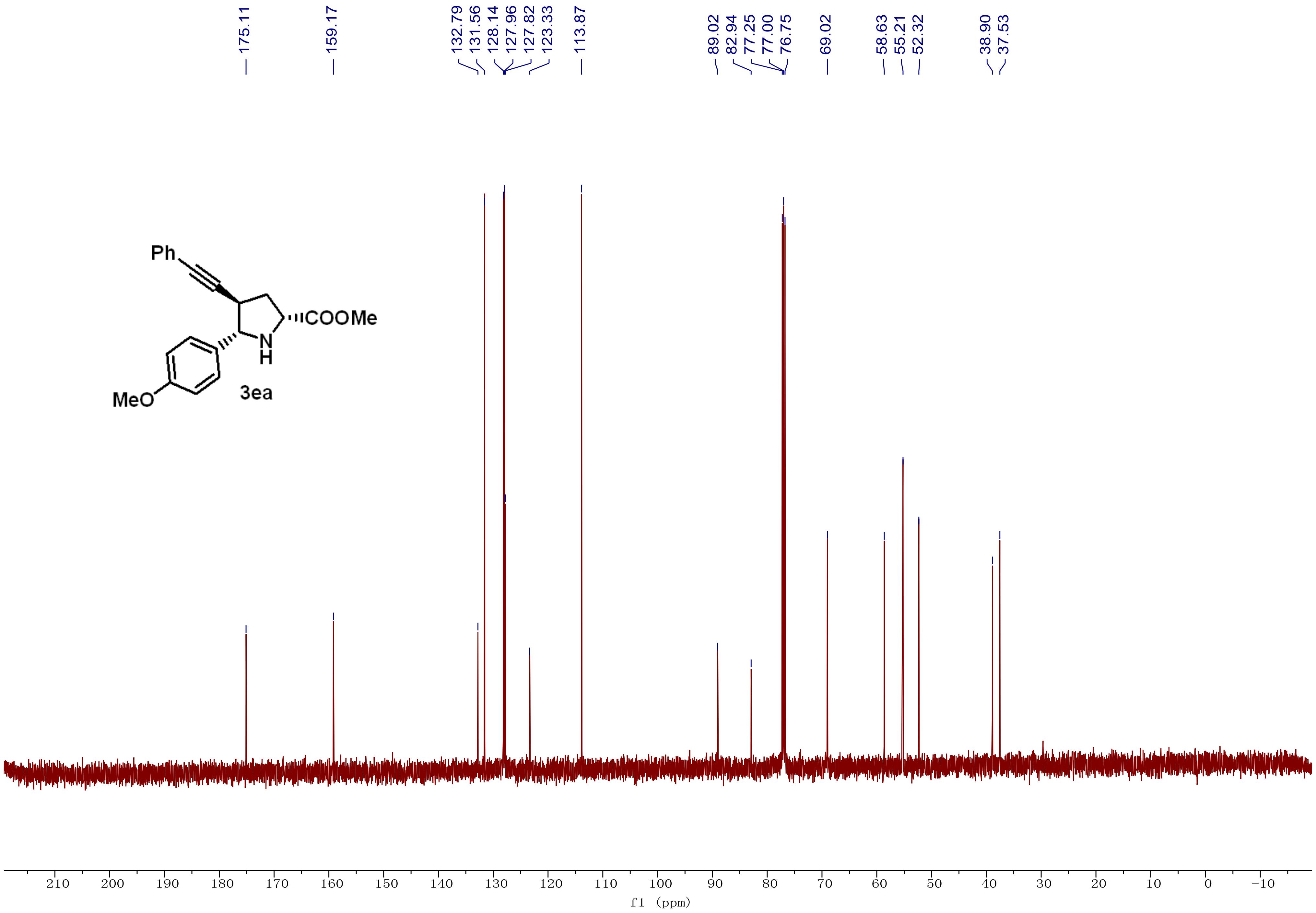
175.11
159.17
132.79
131.56
128.14
127.96
127.82
123.33
113.87
89.02
82.94
77.25
77.00
76.75
69.02
58.63
55.21
52.32
38.90
37.53
Ph
COOMe
N
H
MeO
3ea
210 200 190 180 170 160 150 140 130 120 110 100 90 80 70 60 50 40 30 20 10 0 −10
f1 (ppm)

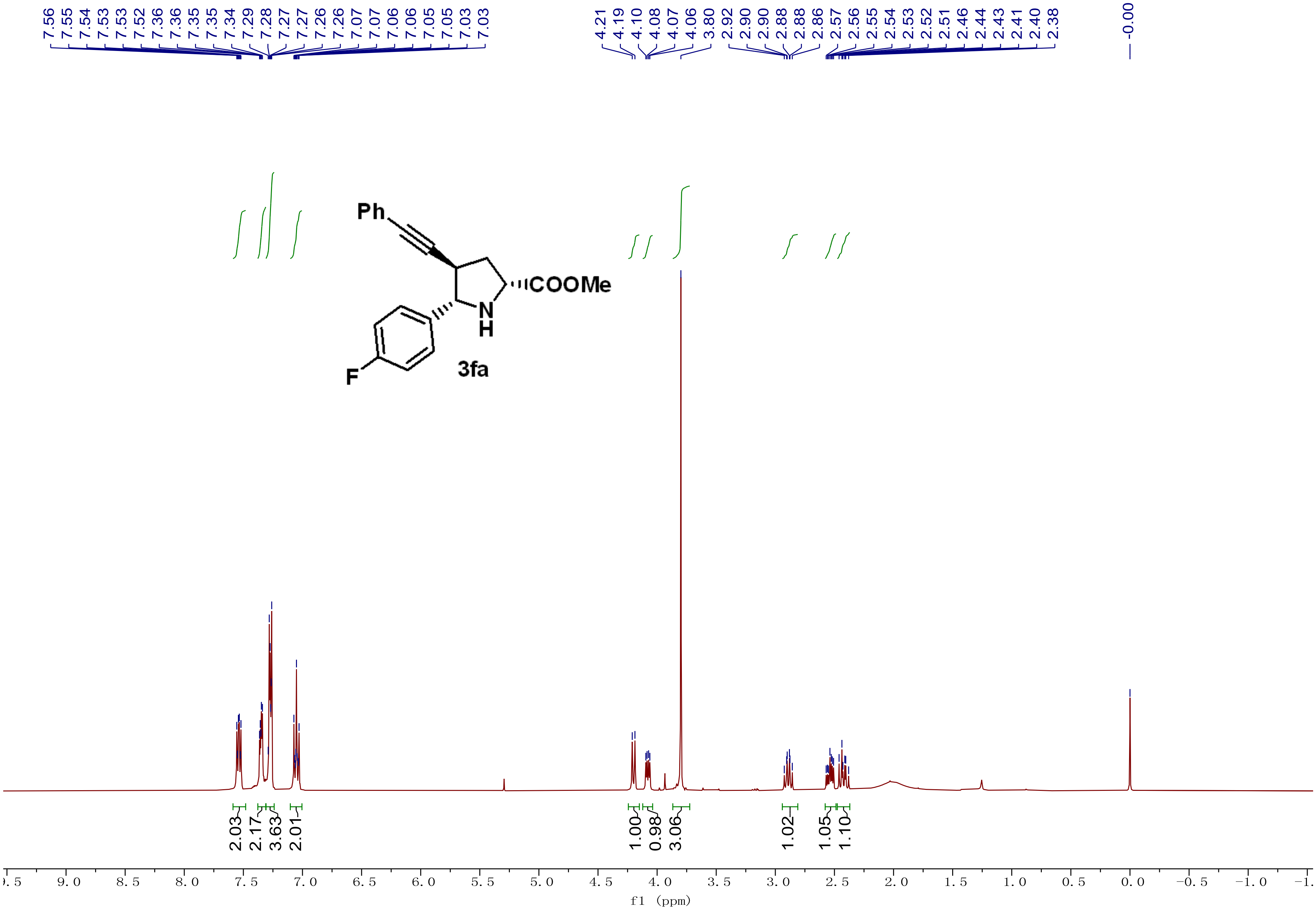
7.56
7.55
7.54
7.53
7.53
7.52
7.36
7.36
7.35
7.35
7.34
7.29
7.28
7.27
7.27
7.26
7.26
7.07
7.07
7.06
7.06
7.05
7.05
7.03
7.03
4.21
4.19
4.10
4.08
4.07
4.06
3.80
2.92
2.90
2.90
2.88
2.88
2.86
2.57
2.56
2.55
2.54
2.53
2.52
2.51
2.46
2.44
2.43
2.41
2.40
2.38
-0.00
Ph
COOMe
N
H
F
3fa
2.03
2.17
3.63
2.01
1.00
0.98
3.06
1.02
1.05
1.10
9.0
8.5
8.0
7.5
7.0
6.5
6.0
5.5
5.0
4.5
4.0
3.5
3.0
2.5
2.0
1.5
1.0
0.5
0.0
-0.5
-1.0
f1 (ppm)

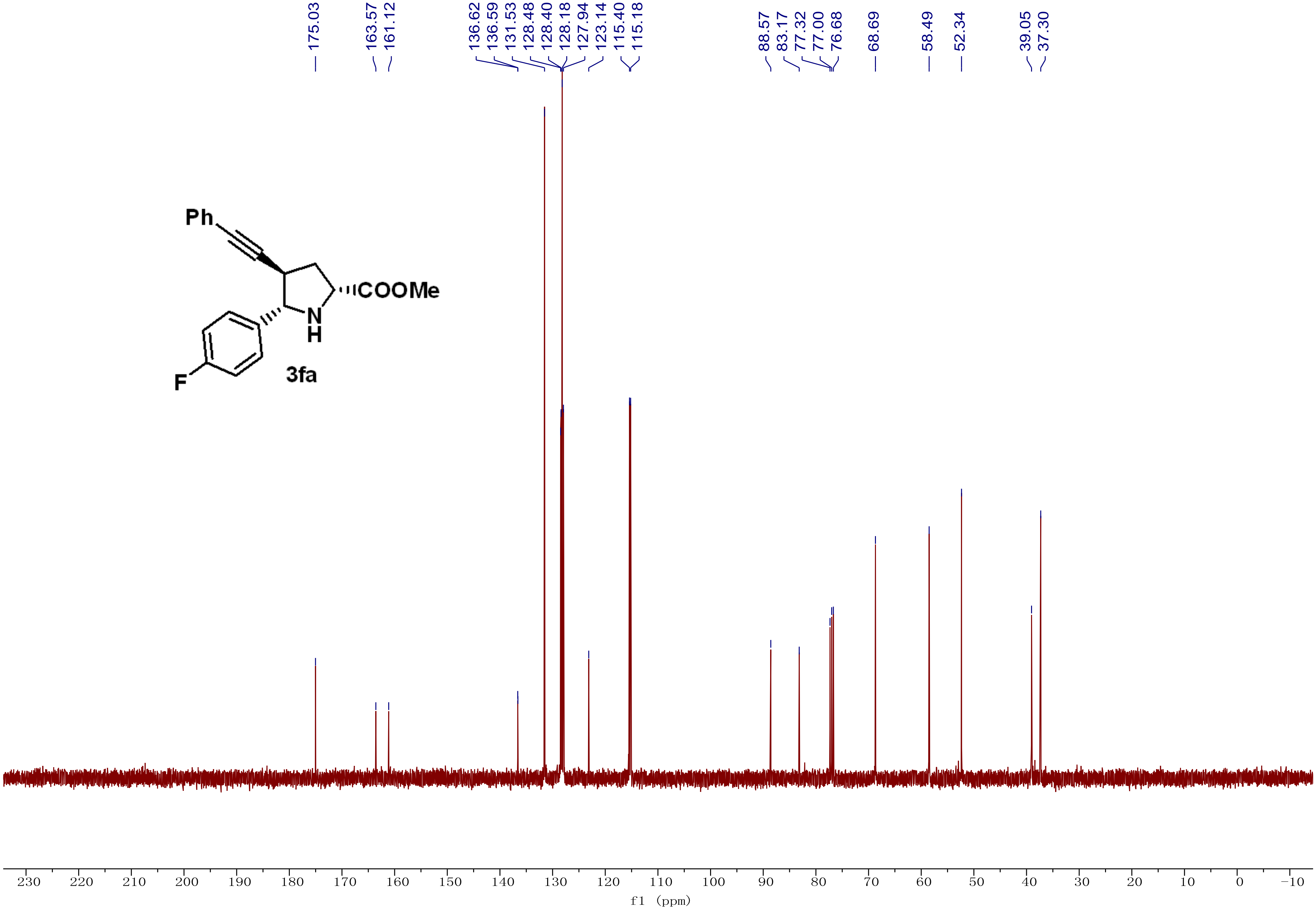

3fa
175.03
163.57
161.12
136.62
136.59
131.53
128.48
128.40
128.18
127.94
123.14
115.40
115.18
88.57
83.17
77.32
77.00
76.68
68.69
58.49
52.34
39.05
37.30
230 220 210 200 190 180 170 160 150 140 130 120 110 100 90 80 70 60 50 40 30 20 10 0 −10
f1 (ppm)

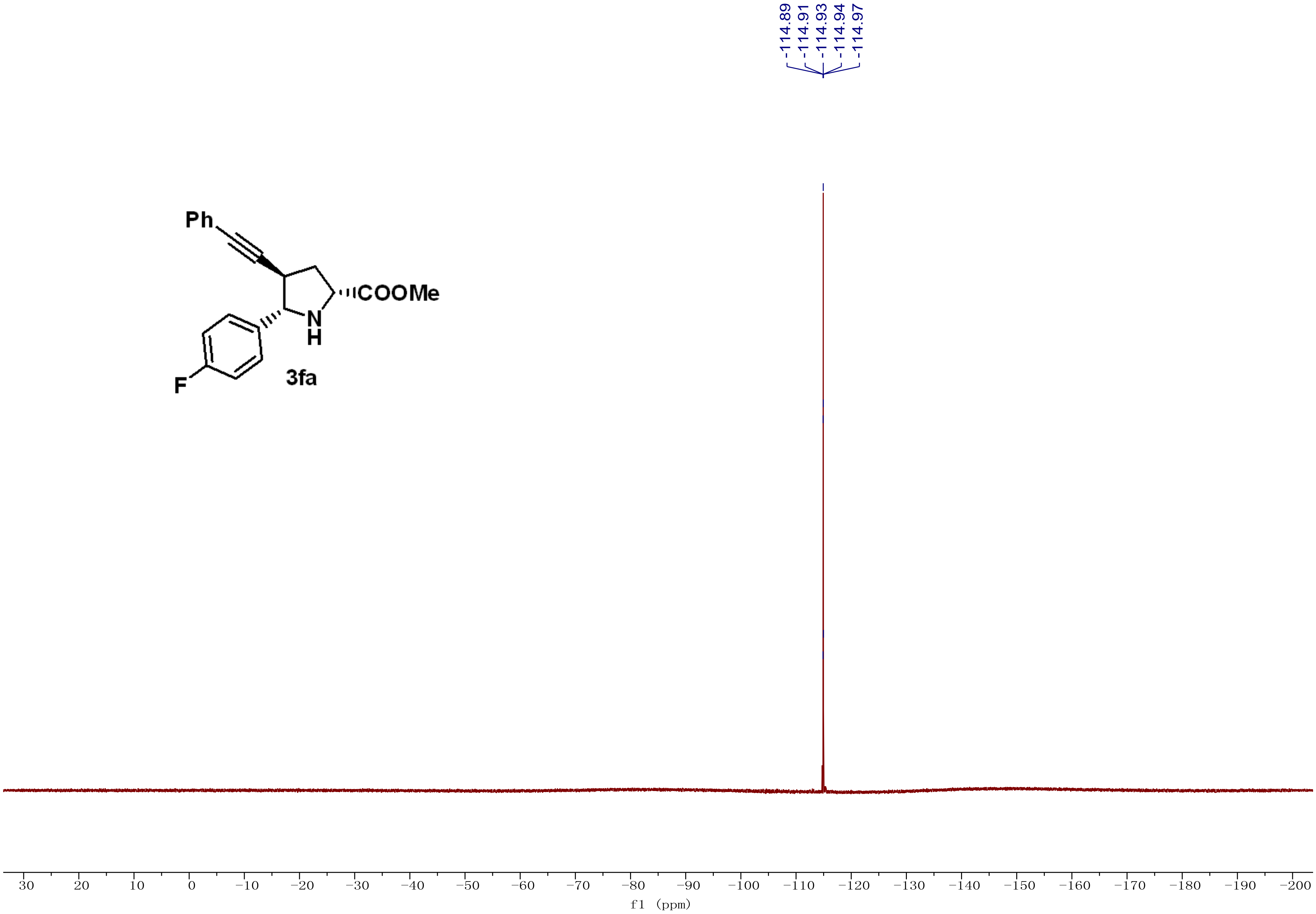

Ph
COOMe
N
H
F
-114.89
-114.91
-114.93
-114.94
-114.97
30 20 10 0 −10 −20 −30 −40 −50 −60 −70 −80 −90 −100 −110 −120 −130 −140 −150 −160 −170 −180 −190 −200
f1 (ppm)

3fa

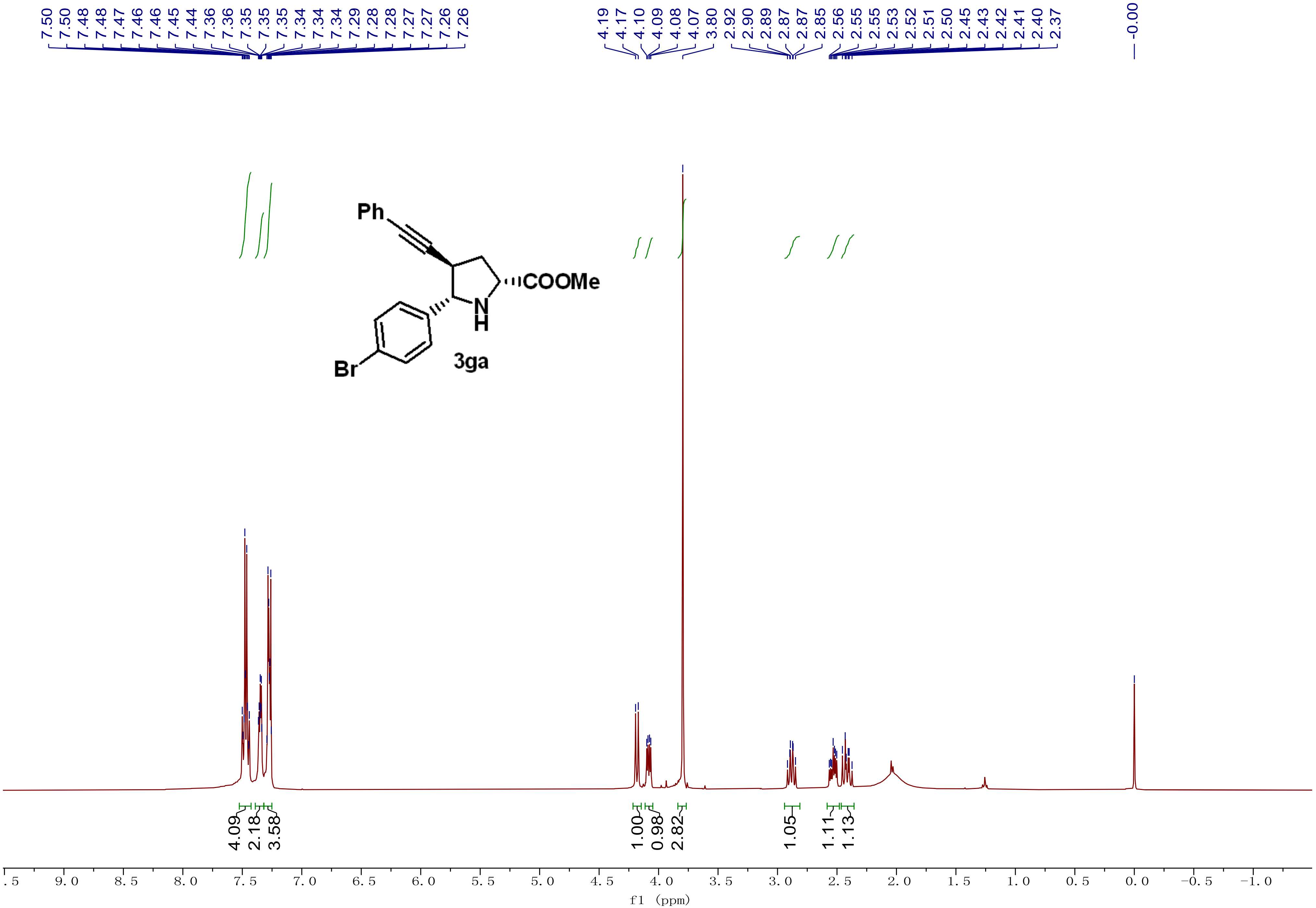
7.50 7.50 7.48 7.48 7.47 7.46 7.46 7.45 7.44 7.36 7.36 7.35 7.35 7.35 7.34 7.34 7.34 7.29 7.28 7.28 7.27 7.27 7.26 7.26
4.19 4.17 4.10 4.09 4.08 4.07 3.80 2.92 2.90 2.89 2.87 2.87 2.85 2.56 2.55 2.55 2.53 2.52 2.51 2.50 2.45 2.43 2.42 2.41 2.40 2.37
-0.00
Ph
COOMe
N
H
Br
3ga
4.09 2.18 3.58
1.00 0.98 2.82
1.05
1.11 1.13
.5 9.0 8.5 8.0 7.5 7.0 6.5 6.0 5.5 5.0 4.5 4.0 3.5 3.0 2.5 2.0 1.5 1.0 0.5 0.0 -0.5 -1.0
f1 (ppm)

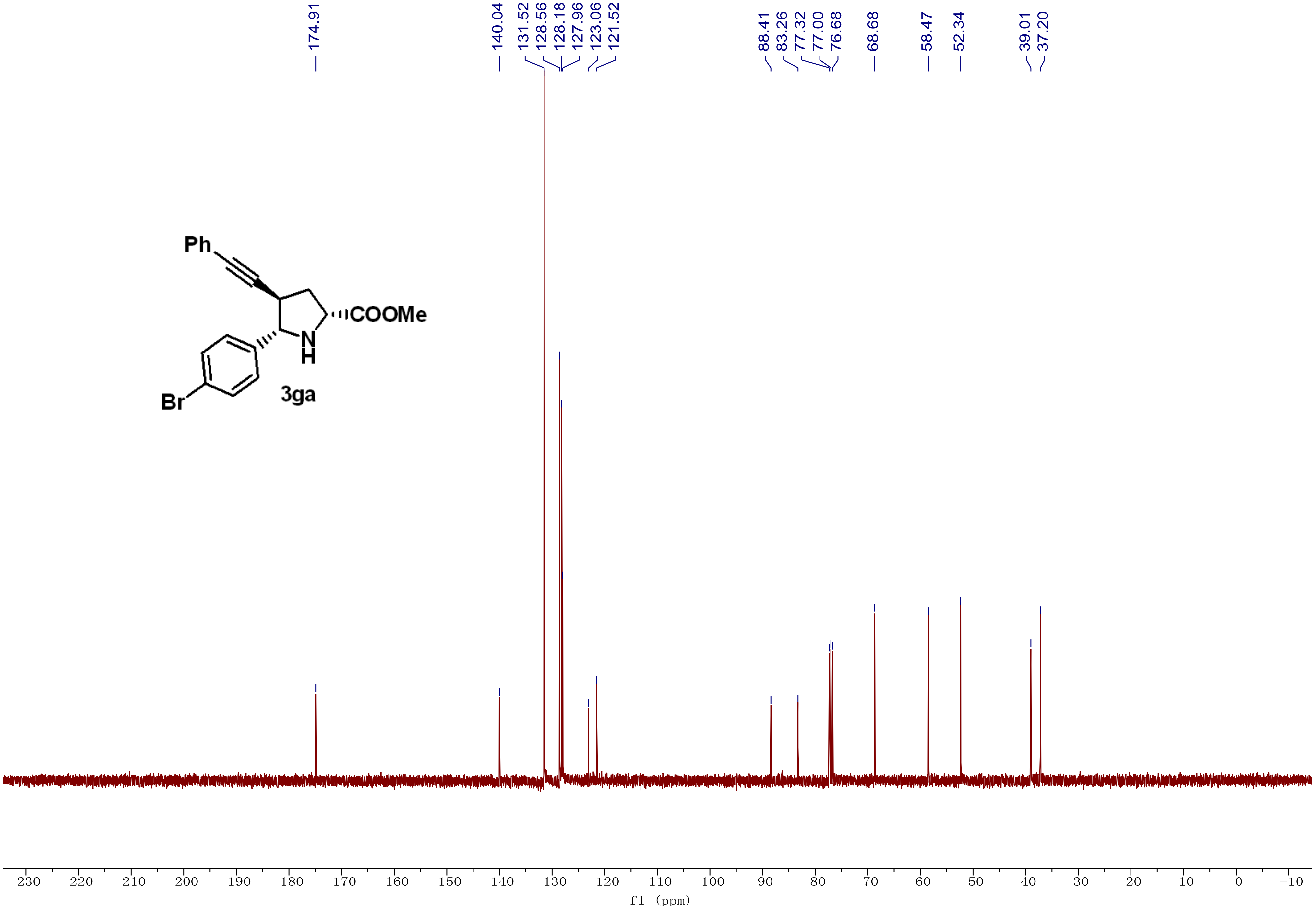

174.91
140.04
131.52
128.56
128.18
127.96
123.06
121.52
88.41
83.26
77.32
77.00
76.68
68.68
58.47
52.34
39.01
37.20
Ph
COOMe
N
H
Br
3ga
230 220 210 200 190 180 170 160 150 140 130 120 110 100 90 80 70 60 50 40 30 20 10 0 -10
f1 (ppm)

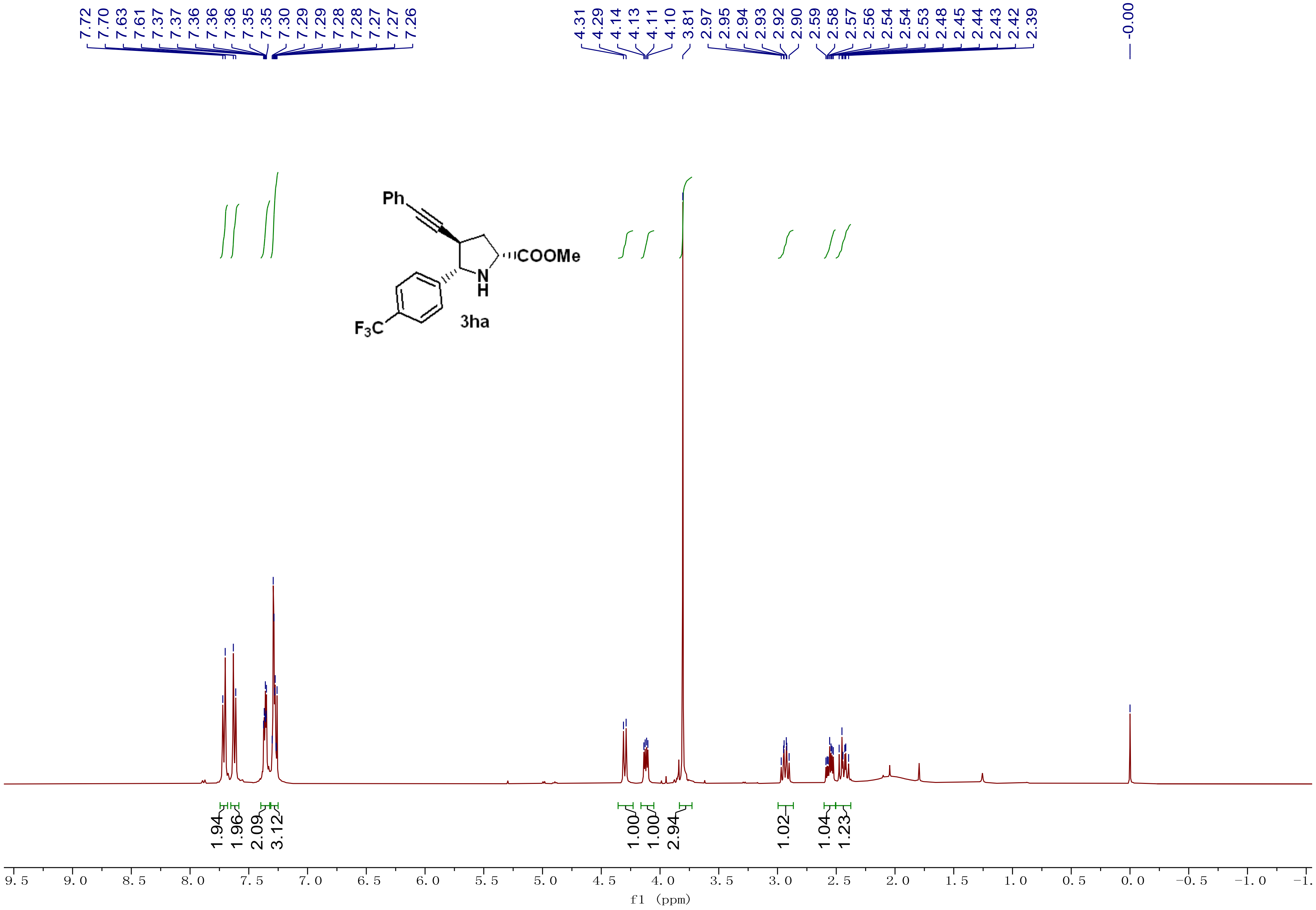
7.72 7.70 7.63 7.61 7.37 7.37 7.36 7.36 7.36 7.35 7.35 7.30 7.29 7.29 7.28 7.28 7.27 7.27 7.26
4.31 4.29 4.14 4.13 4.11 4.10 3.81 2.97 2.95 2.94 2.93 2.92 2.90 2.59 2.58 2.57 2.56 2.54 2.54 2.53 2.48 2.45 2.44 2.43 2.42 2.39
-0.00
Ph
COOMe
N
H
F3C
3ha
1.94 1.96 2.09 3.12 1.00 1.00 2.94 1.02 1.04 1.23
9.5 9.0 8.5 8.0 7.5 7.0 6.5 6.0 5.5 5.0 4.5 4.0 3.5 3.0 2.5 2.0 1.5 1.0 0.5 0.0 -0.5 -1.0
f1 (ppm)

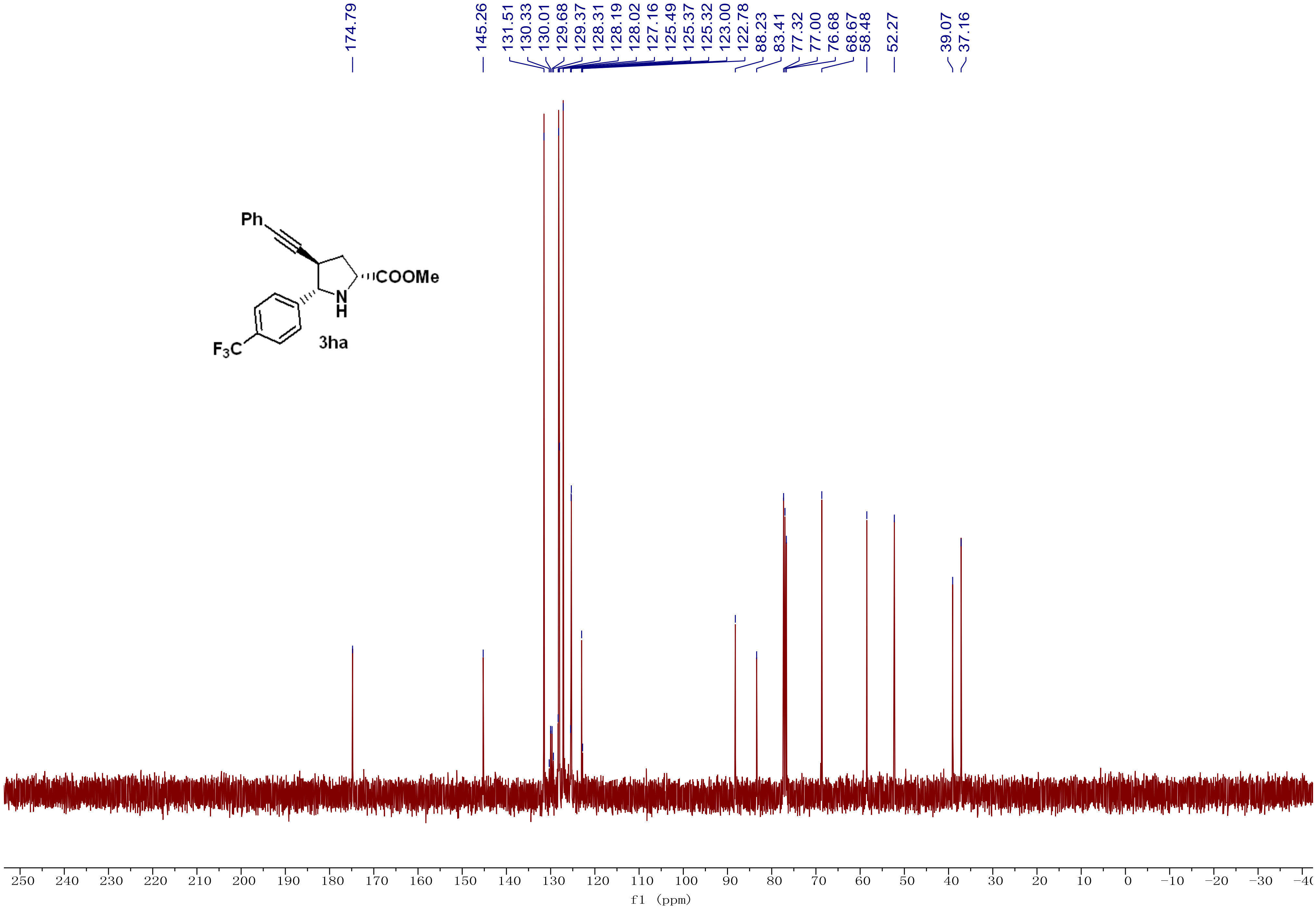
174.79
145.26
131.51
130.33
130.01
129.68
129.37
128.31
128.19
128.02
127.16
125.49
125.37
125.32
123.00
122.78
88.23
83.41
77.32
77.00
76.68
68.67
58.48
52.27
39.07
37.16
Ph
COOMe
N
H
F3C
3ha
250 240 230 220 210 200 190 180 170 160 150 140 130 120 110 100 90 80 70 60 50 40 30 20 10 0 −10 −20 −30
f1 (ppm)

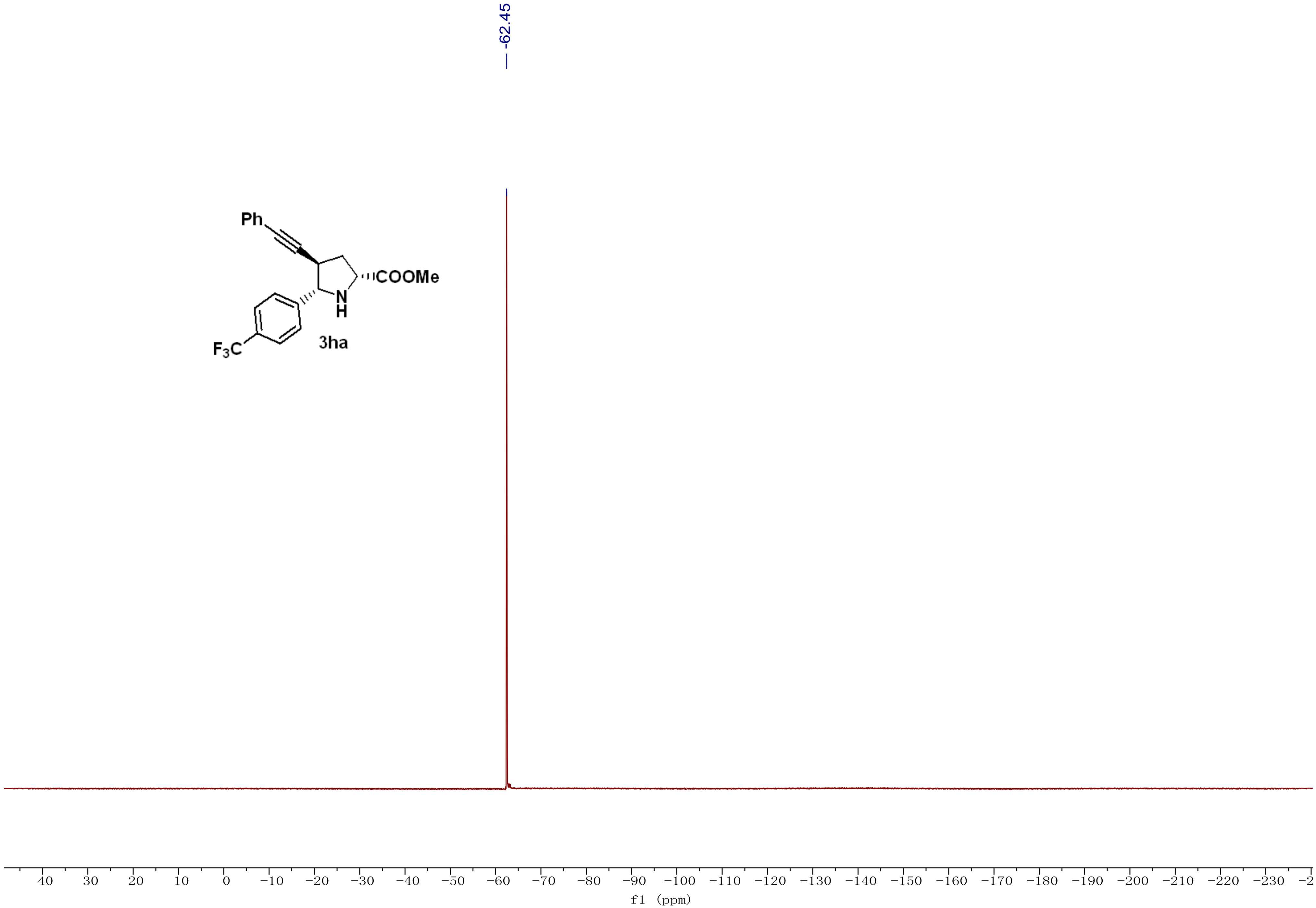
-62.45
Ph
COOMe
N
H
F3C
3ha
40 30 20 10 0 −10 −20 −30 −40 −50 −60 −70 −80 −90 −100 −110 −120 −130 −140 −150 −160 −170 −180 −190 −200 −210 −220 −230 −2
f1 (ppm)

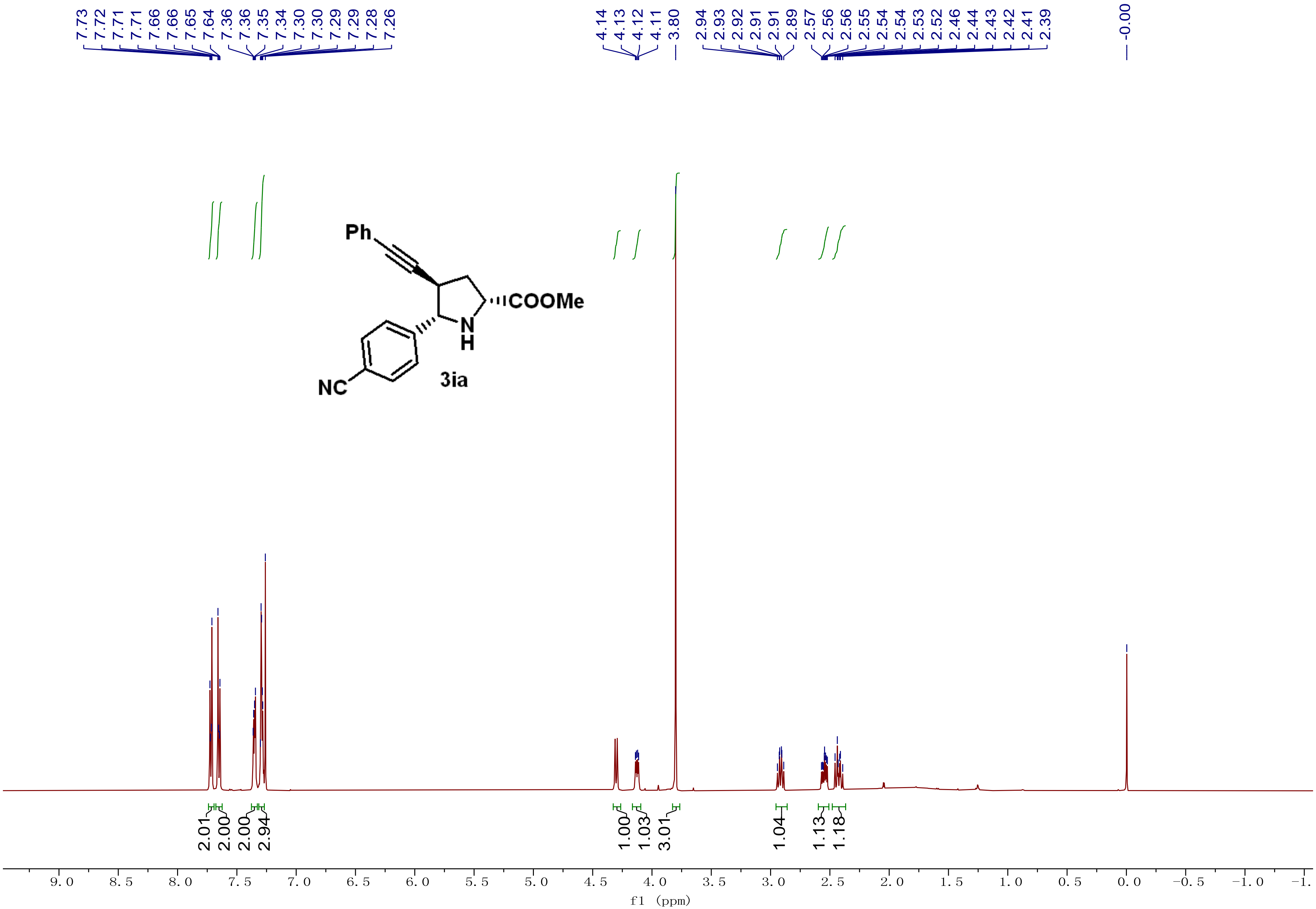
7.73 7.72 7.71 7.71 7.66 7.66 7.65 7.64 7.36 7.36 7.35 7.34 7.30 7.30 7.29 7.29 7.28 7.26
4.14 4.13 4.12 4.11 3.80 2.94 2.93 2.92 2.91 2.91 2.89 2.57 2.56 2.56 2.55 2.54 2.54 2.53 2.52 2.46 2.44 2.43 2.42 2.41 2.39
-0.00
Ph
COOMe
N
H
NC
3ia
2.01 2.00 2.00 2.94
1.00 1.03 3.01
1.04 1.13 1.18
9.0 8.5 8.0 7.5 7.0 6.5 6.0 5.5 5.0 4.5 4.0 3.5 3.0 2.5 2.0 1.5 1.0 0.5 0.0 −0.5 −1.0 −1.
f1 (ppm)

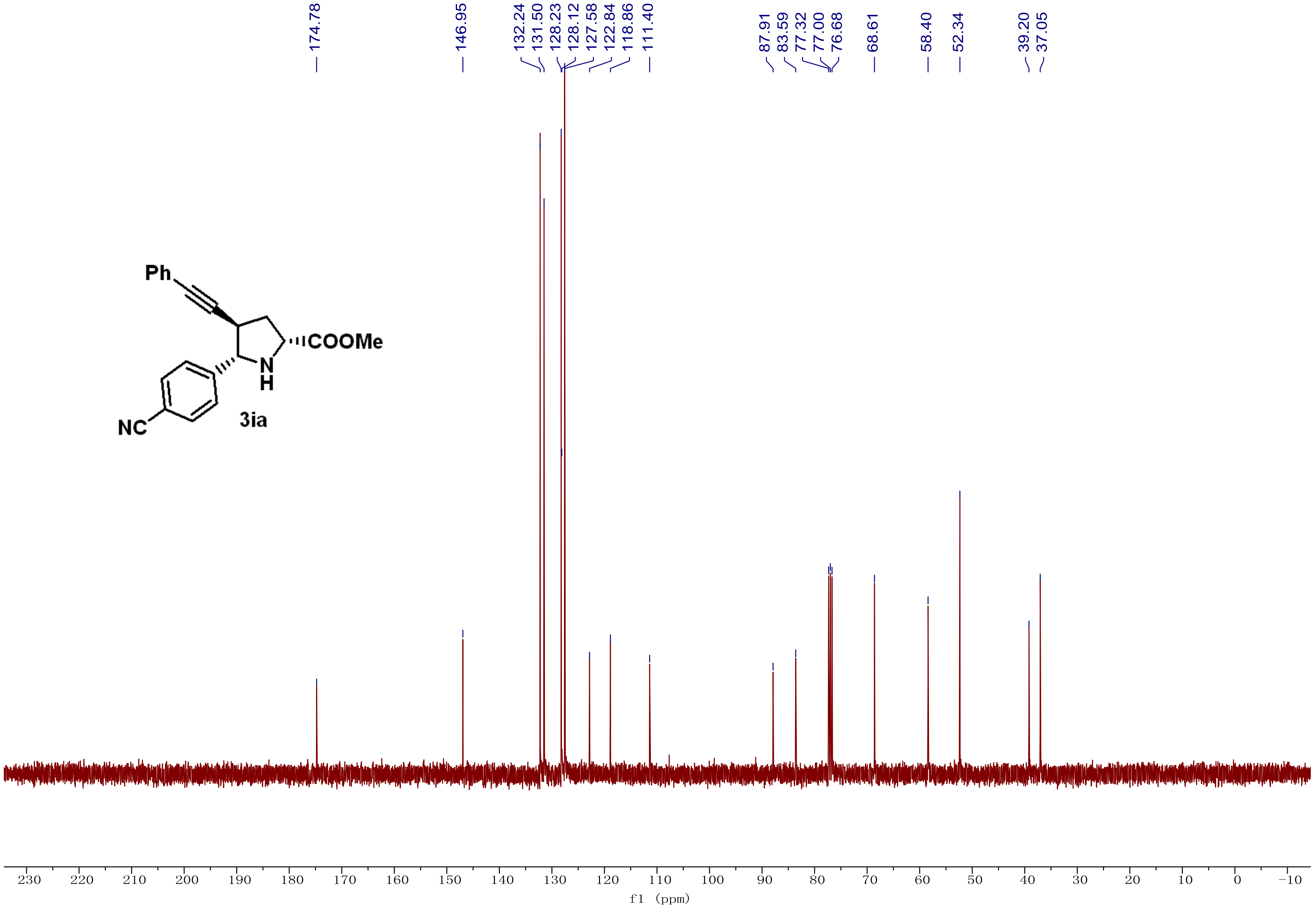

3ia
174.78
146.95
132.24
131.50
128.23
128.12
127.58
122.84
118.86
111.40
87.91
83.59
77.32
77.00
76.68
68.61
58.40
52.34
39.20
37.05
230
220
210
200
190
180
170
160
150
140
130
120
110
100
90
80
70
60
50
40
30
20
10
0
−10
f1 (ppm)

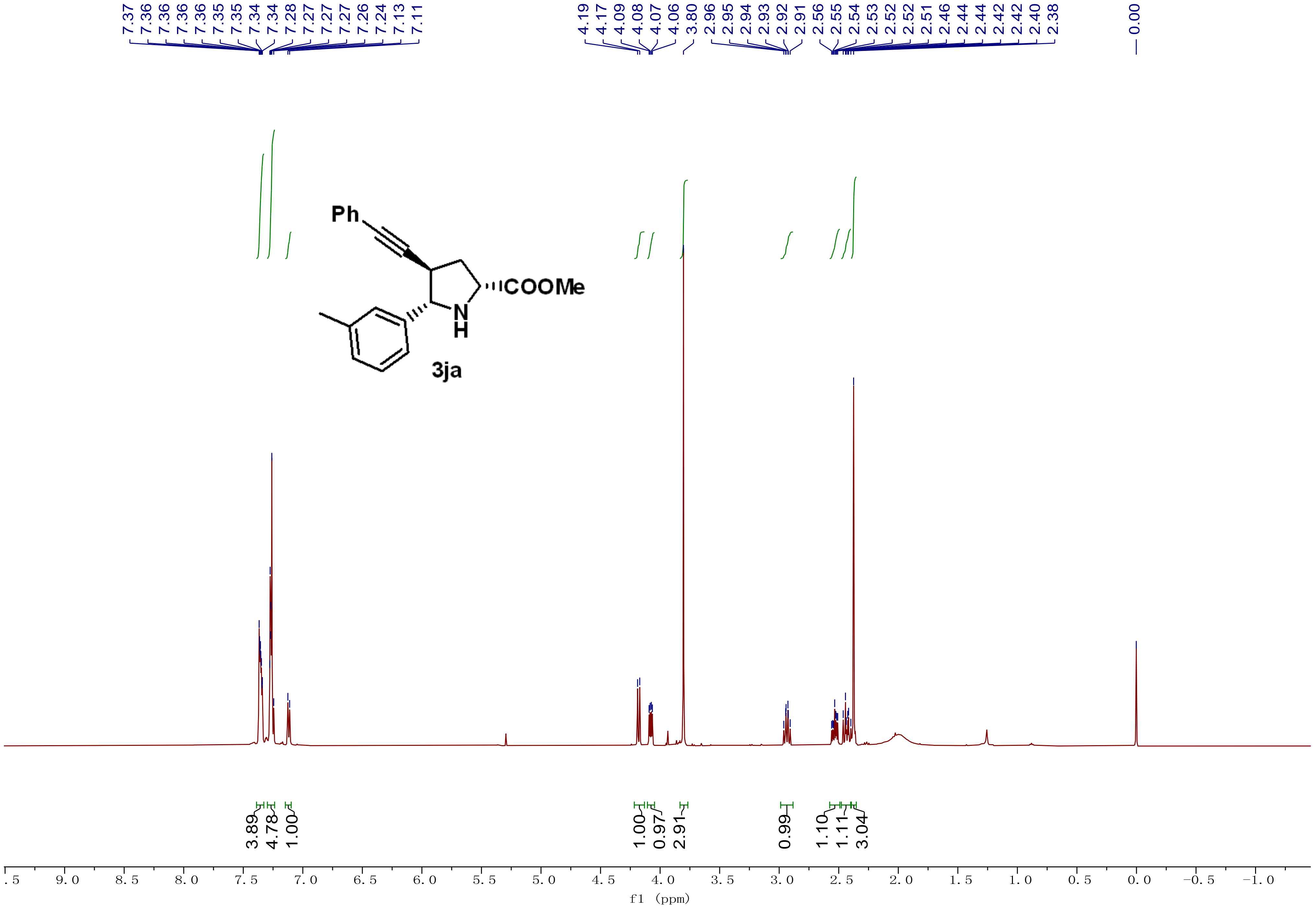
7.37
7.36
7.36
7.36
7.36
7.35
7.35
7.34
7.34
7.28
7.27
7.27
7.27
7.26
7.24
7.13
7.11
4.19
4.17
4.09
4.08
4.07
4.06
3.80
2.96
2.95
2.94
2.93
2.92
2.91
2.56
2.55
2.54
2.53
2.52
2.52
2.51
2.46
2.44
2.44
2.42
2.42
2.40
2.38
0.00
Ph
COOMe
N
H
3ja
3.89
4.78
1.00
1.00
0.97
2.91
0.99
1.10
1.11
3.04
f1 (ppm)

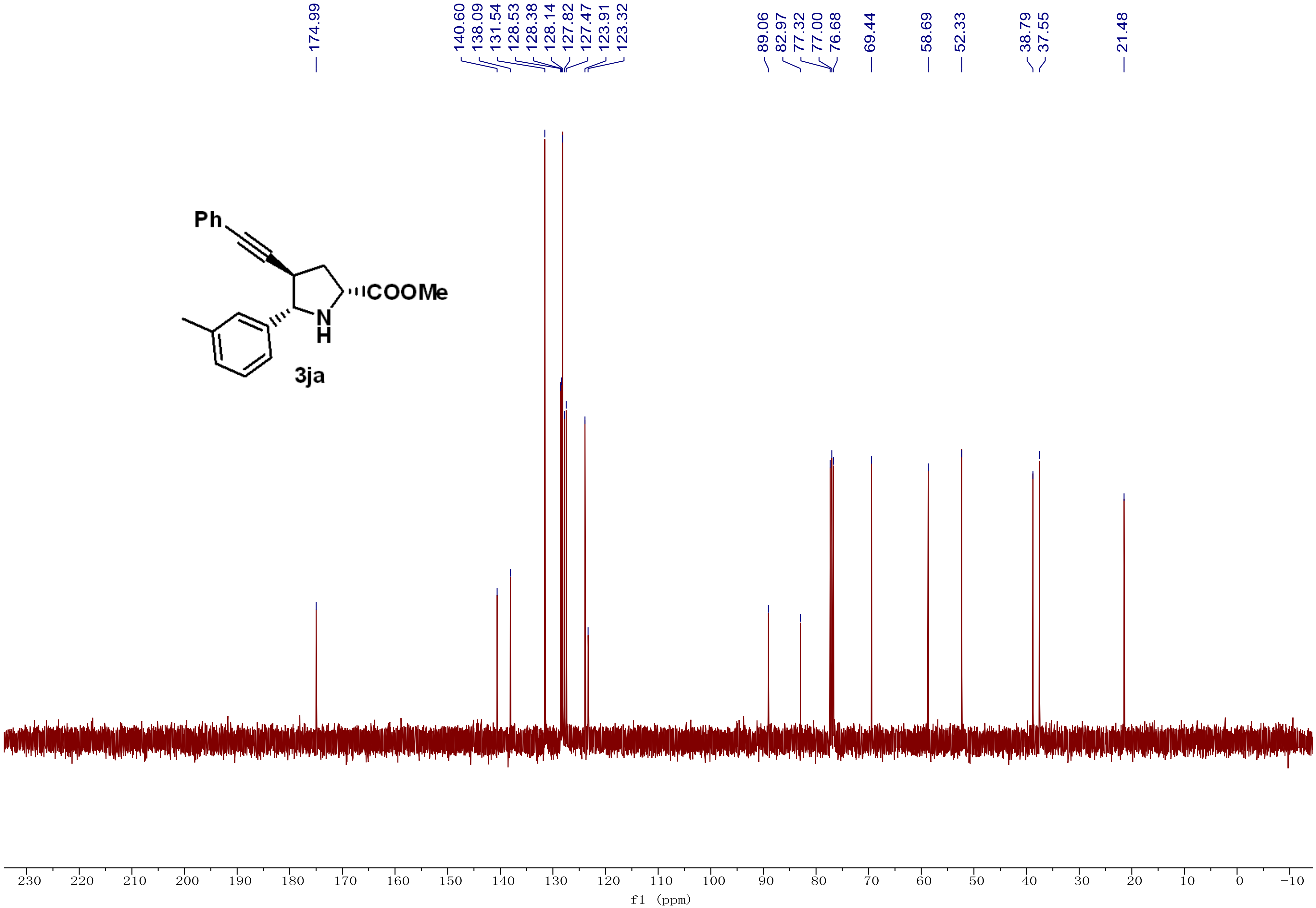
174.99
140.60
138.09
131.54
128.53
128.38
128.14
127.82
127.47
123.91
123.32
89.06
82.97
77.32
77.00
76.68
69.44
58.69
52.33
38.79
37.55
21.48
Ph
COOMe
N
H
3ja
230 220 210 200 190 180 170 160 150 140 130 120 110 100 90 80 70 60 50 40 30 20 10 0 −10
f1 (ppm)

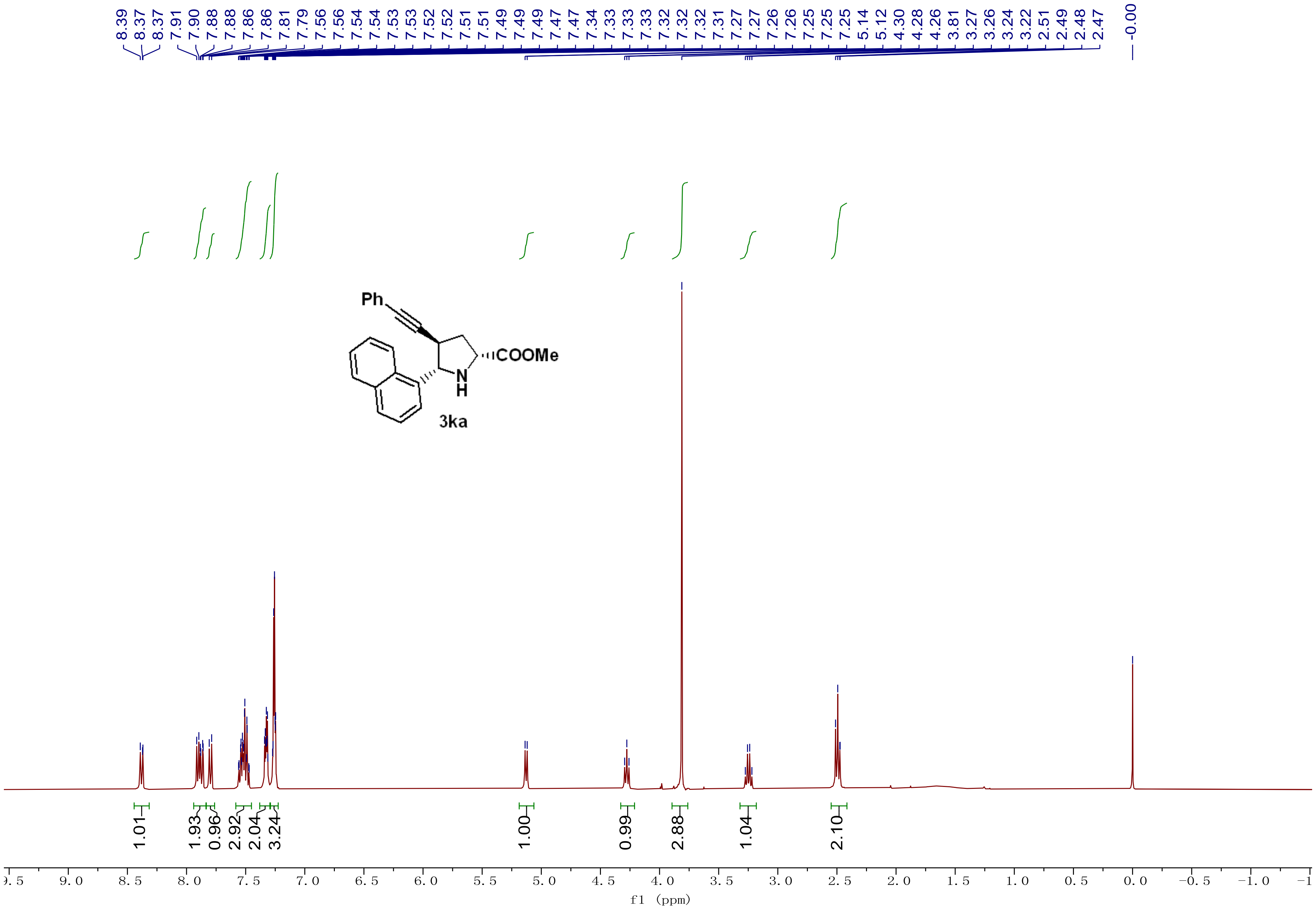

8.39 8.37 8.37 7.91 7.90 7.88 7.88 7.86 7.86 7.81 7.79 7.56 7.56 7.54 7.54 7.53 7.53 7.52 7.52 7.51 7.51 7.49 7.49 7.47 7.47 7.34 7.33 7.33 7.33 7.32 7.32 7.32 7.31 7.27 7.27 7.26 7.26 7.25 7.25 7.25 5.14 5.12 4.30 4.28 4.26 3.81 3.27 3.26 3.24 3.22 2.51 2.49 2.48 2.47
-0.00
Ph
COOMe
N
H
3ka
1.01 1.93 0.96 2.92 2.04 3.24 1.00 0.99 2.88 1.04 2.10
9.0 8.5 8.0 7.5 7.0 6.5 6.0 5.5 5.0 4.5 4.0 3.5 3.0 2.5 2.0 1.5 1.0 0.5 0.0 −0.5 −1.0
f1 (ppm)

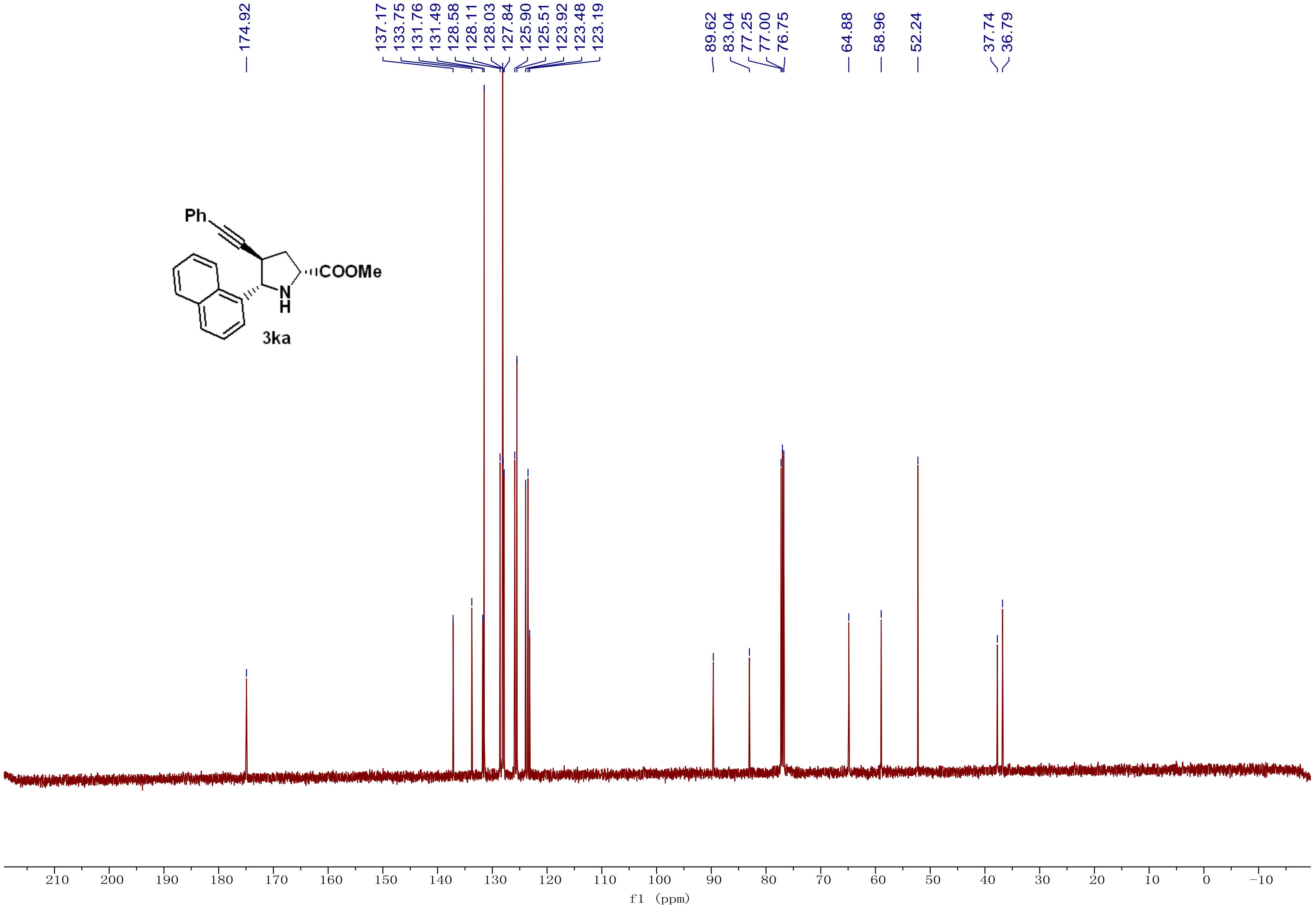

174.92
137.17
133.75
131.76
131.49
128.58
128.11
128.03
127.84
125.90
125.51
123.92
123.48
123.19
89.62
83.04
77.25
77.00
76.75
64.88
58.96
52.24
37.74
36.79
Ph
COOMe
N
H
3ka
210
200
190
180
170
160
150
140
130
120
110
100
90
80
70
60
50
40
30
20
10
0
-10
f1 (ppm)

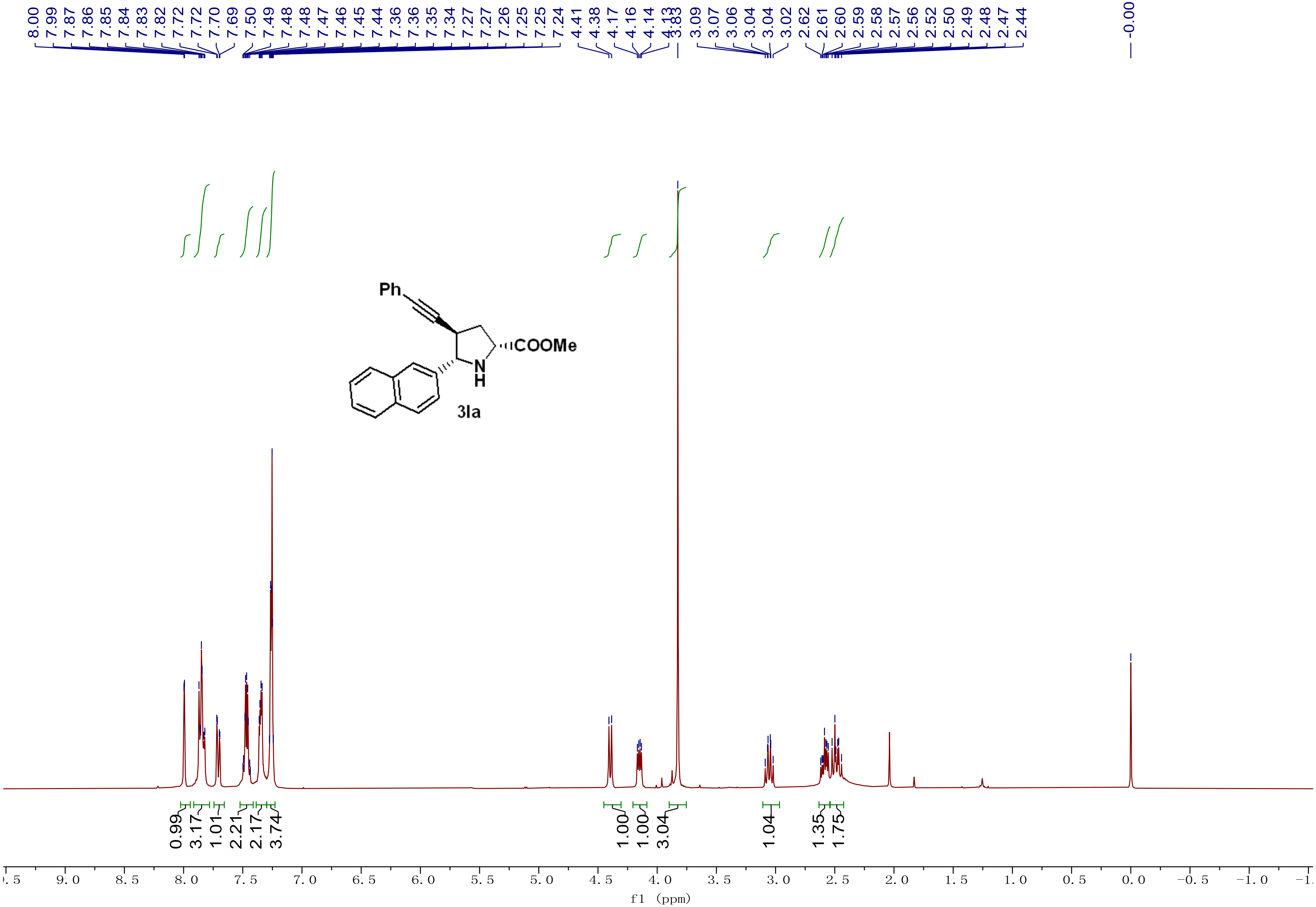
8.00 7.99 7.87 7.86 7.85 7.84 7.83 7.82 7.72 7.72 7.70 7.69 7.50 7.49 7.48 7.48 7.47 7.46 7.45 7.44 7.36 7.36 7.35 7.34 7.27 7.27 7.26 7.25 7.25 7.24
4.41 4.38 4.17 4.16 4.14 4.13 3.83 3.09 3.07 3.06 3.04 3.04 3.02 2.62 2.61 2.60 2.59 2.58 2.57 2.56 2.52 2.50 2.49 2.48 2.47 2.44
-0.00
Ph
COOMe
N
H
3la
0.99
3.17
1.01
2.21
2.17
3.74
1.00
1.00
3.04
1.04
1.35
1.75
9.0 8.5 8.0 7.5 7.0 6.5 6.0 5.5 5.0 4.5 4.0 3.5 3.0 2.5 2.0 1.5 1.0 0.5 0.0 −0.5 −1.0
f1 (ppm)

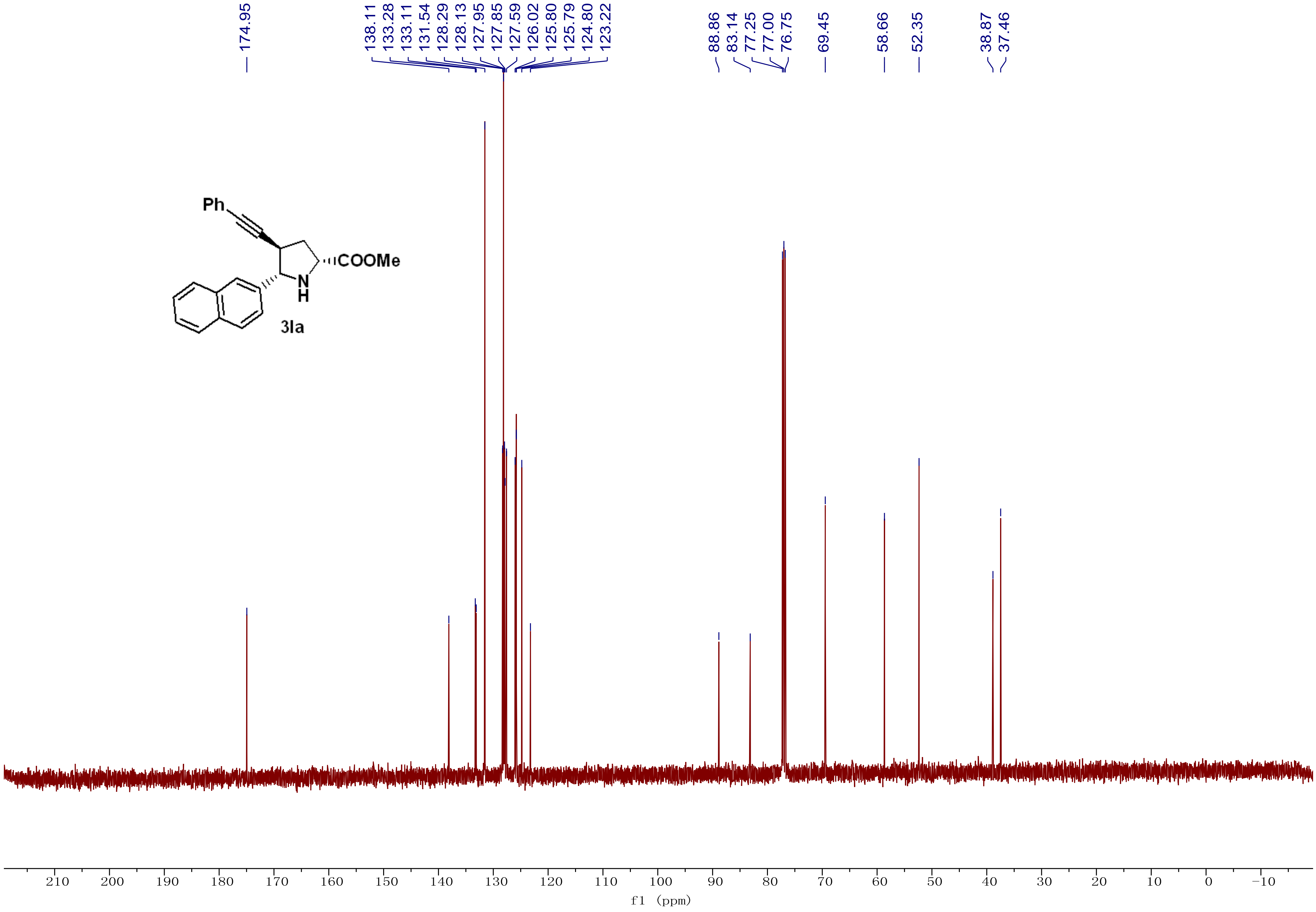
174.95
138.11
133.28
133.11
131.54
128.29
128.13
127.95
127.85
127.59
126.02
125.80
125.79
124.80
123.22
88.86
83.14
77.25
77.00
76.75
69.45
58.66
52.35
38.87
37.46
Ph
COOMe
N
H
3la
210 200 190 180 170 160 150 140 130 120 110 100 90 80 70 60 50 40 30 20 10 0 −10
f1 (ppm)

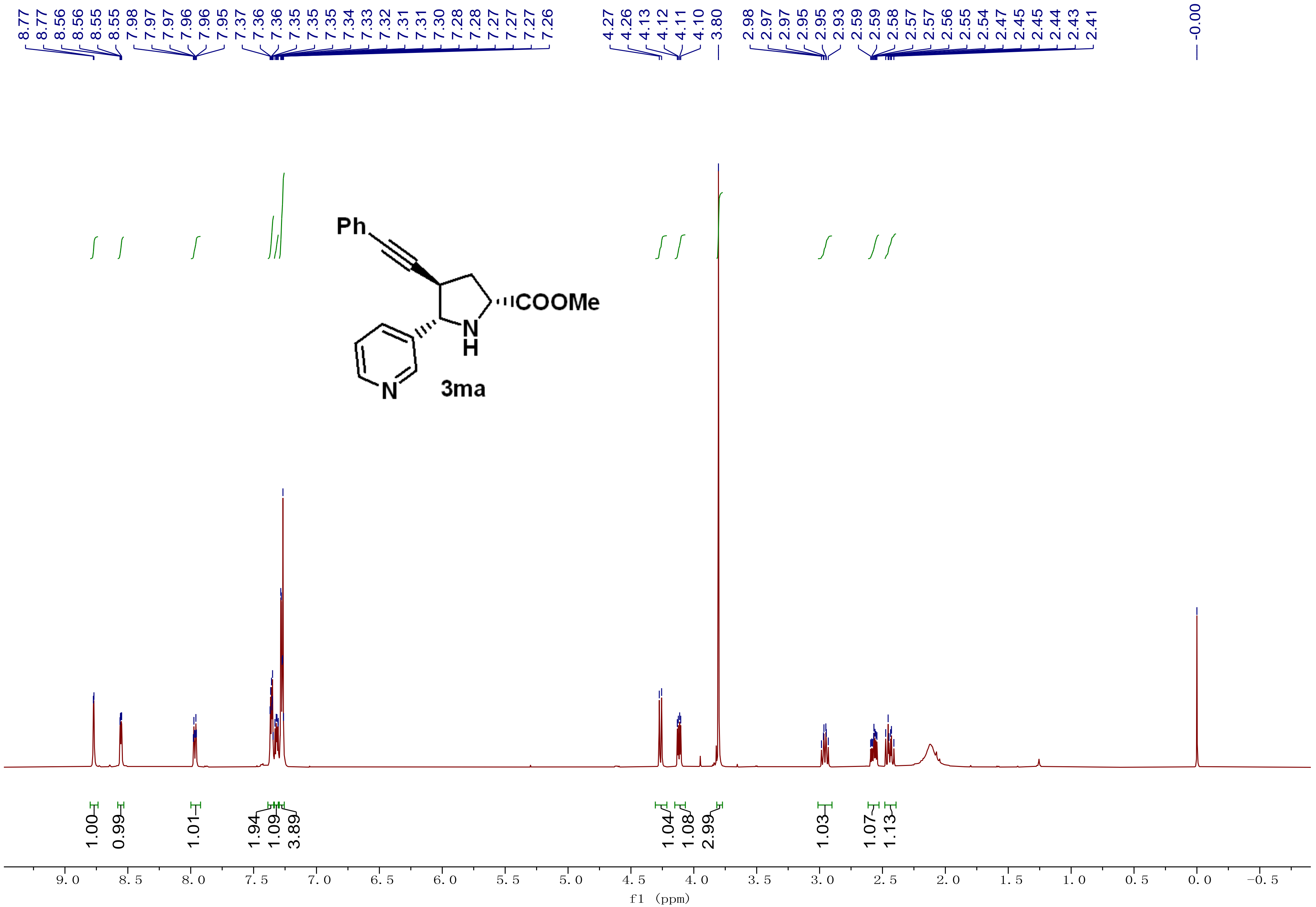
8.77 8.77 8.56 8.56 8.55 8.55 7.98 7.97 7.97 7.96 7.96 7.95 7.37 7.36 7.36 7.35 7.35 7.35 7.34 7.33 7.32 7.31 7.31 7.30 7.28 7.28 7.27 7.27 7.27 7.26
4.27 4.26 4.13 4.12 4.11 4.10 3.80
2.98 2.97 2.97 2.95 2.95 2.93 2.59 2.59 2.58 2.57 2.57 2.56 2.55 2.54 2.47 2.45 2.45 2.44 2.43 2.41
-0.00
Ph
COOMe
N
H
N
3ma
1.00 0.99 1.01 1.94 1.09 3.89 1.04 1.08 2.99 1.03 1.07 1.13
9.0 8.5 8.0 7.5 7.0 6.5 6.0 5.5 5.0 4.5 4.0 3.5 3.0 2.5 2.0 1.5 1.0 0.5 0.0 -0.5
f1 (ppm)

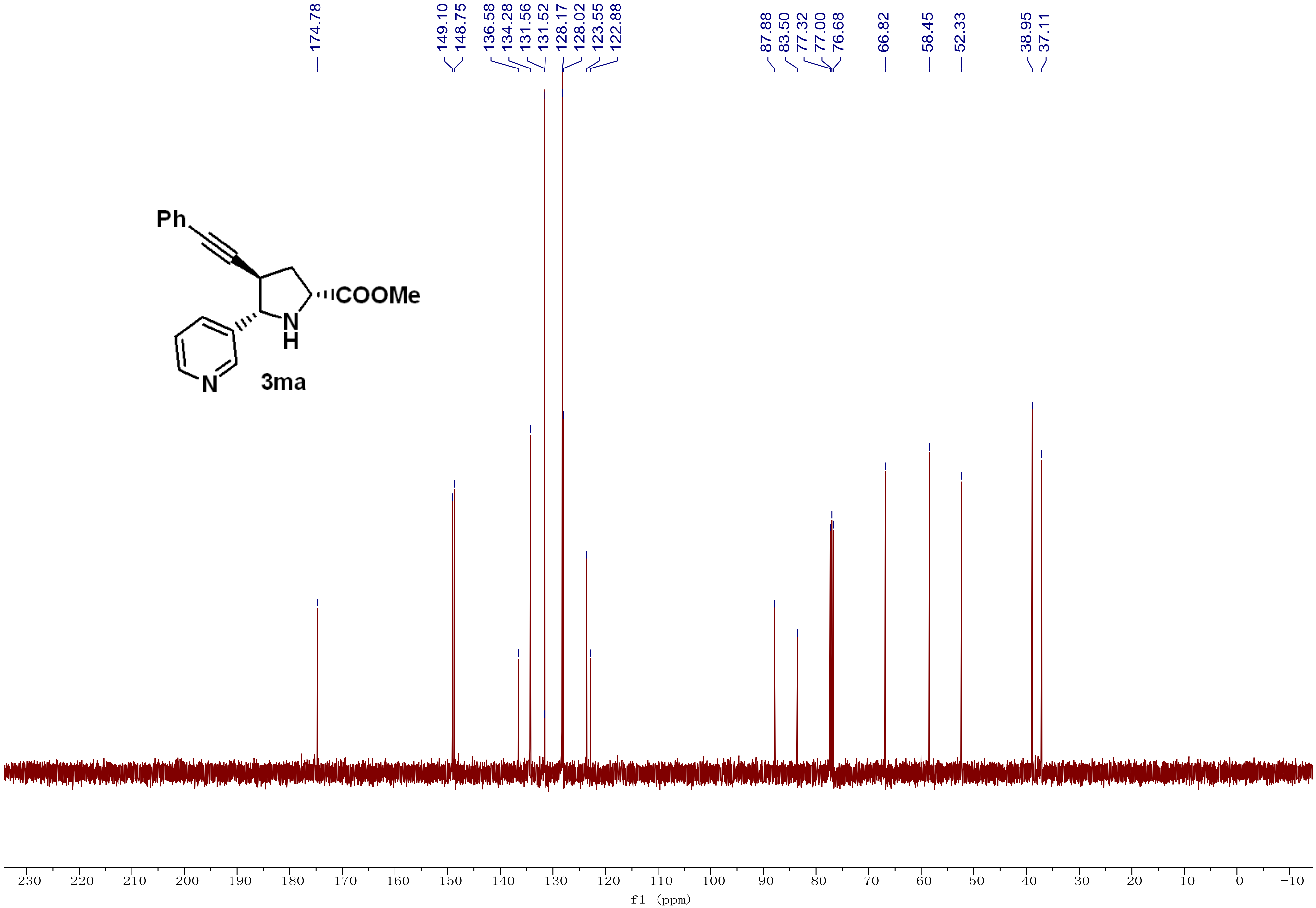
174.78
149.10
148.75
136.58
134.28
131.56
131.52
128.17
128.02
123.55
122.88
87.88
83.50
77.32
77.00
76.68
66.82
58.45
52.33
38.95
37.11
Ph
COOMe
N
H
N
3ma
230 220 210 200 190 180 170 160 150 140 130 120 110 100 90 80 70 60 50 40 30 20 10 0 −10
f1 (ppm)

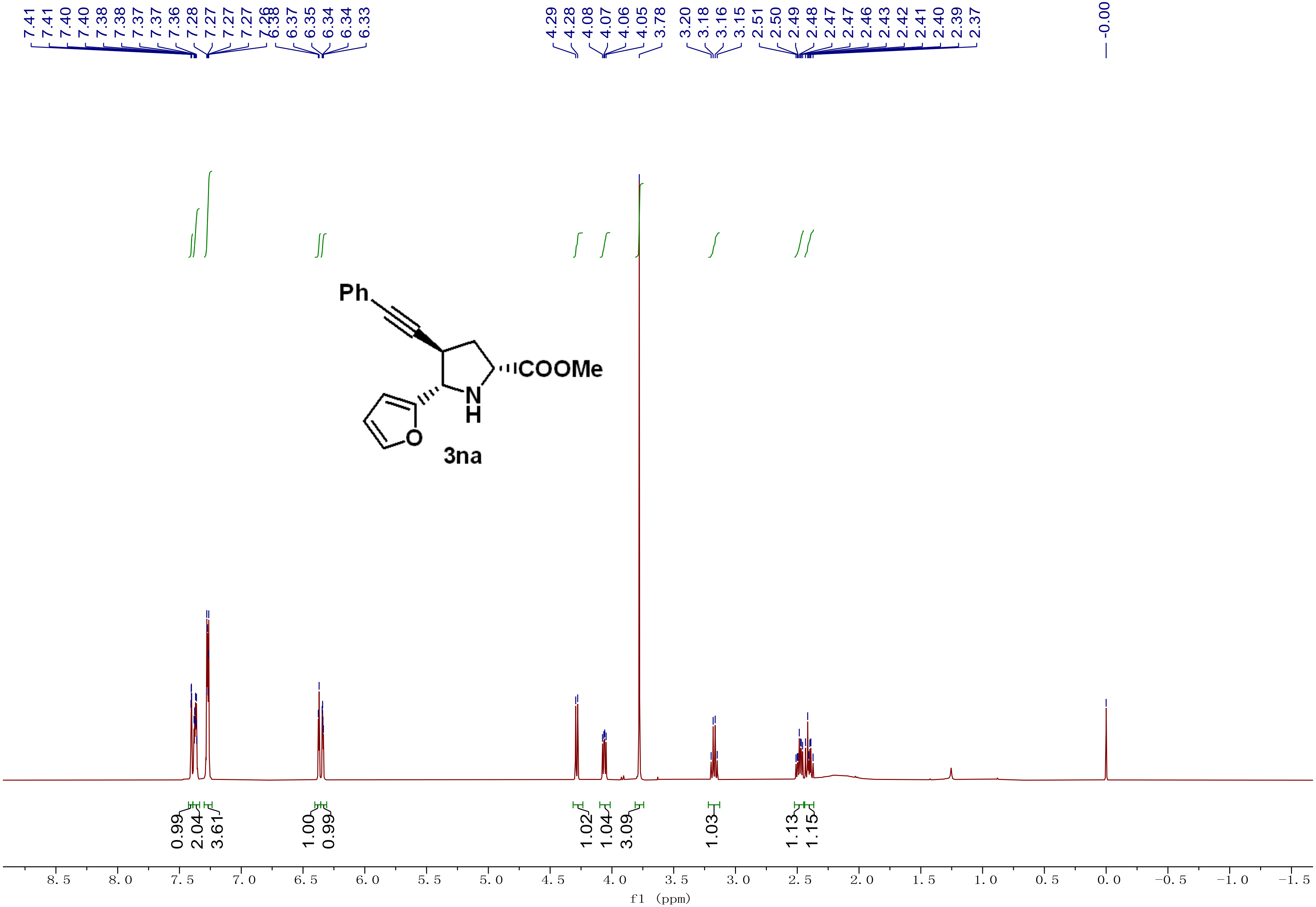
7.41
7.41
7.40
7.40
7.38
7.38
7.37
7.37
7.36
7.28
7.27
7.27
7.27
7.26
6.38
6.37
6.35
6.34
6.34
6.33
4.29
4.28
4.08
4.07
4.06
4.05
3.78
3.20
3.18
3.16
3.15
2.51
2.50
2.49
2.48
2.47
2.47
2.46
2.43
2.42
2.41
2.40
2.39
2.37
-0.00
Ph
COOMe
N
H
O
3na
0.99
2.04
3.61
1.00
0.99
1.02
1.04
3.09
1.03
1.13
1.15
8.5
8.0
7.5
7.0
6.5
6.0
5.5
5.0
4.5
4.0
3.5
3.0
2.5
2.0
1.5
1.0
0.5
0.0
−0.5
−1.0
−1.5
f1 (ppm)

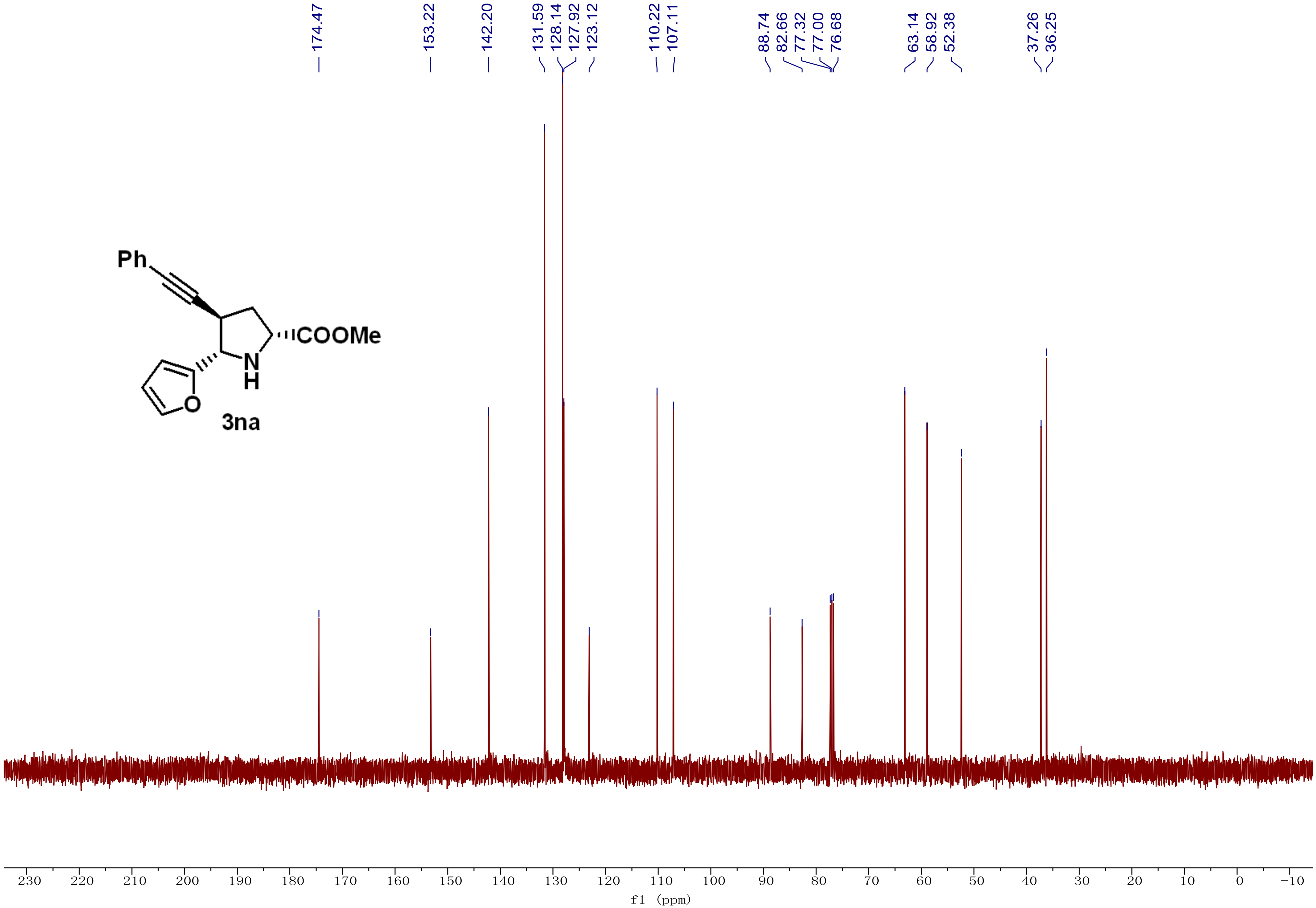
174.47
153.22
142.20
131.59
128.14
127.92
123.12
110.22
107.11
88.74
82.66
77.32
77.00
76.68
63.14
58.92
52.38
37.26
36.25
Ph
COOMe
N
H
O
3na
230 220 210 200 190 180 170 160 150 140 130 120 110 100 90 80 70 60 50 40 30 20 10 0 −10
f1 (ppm)

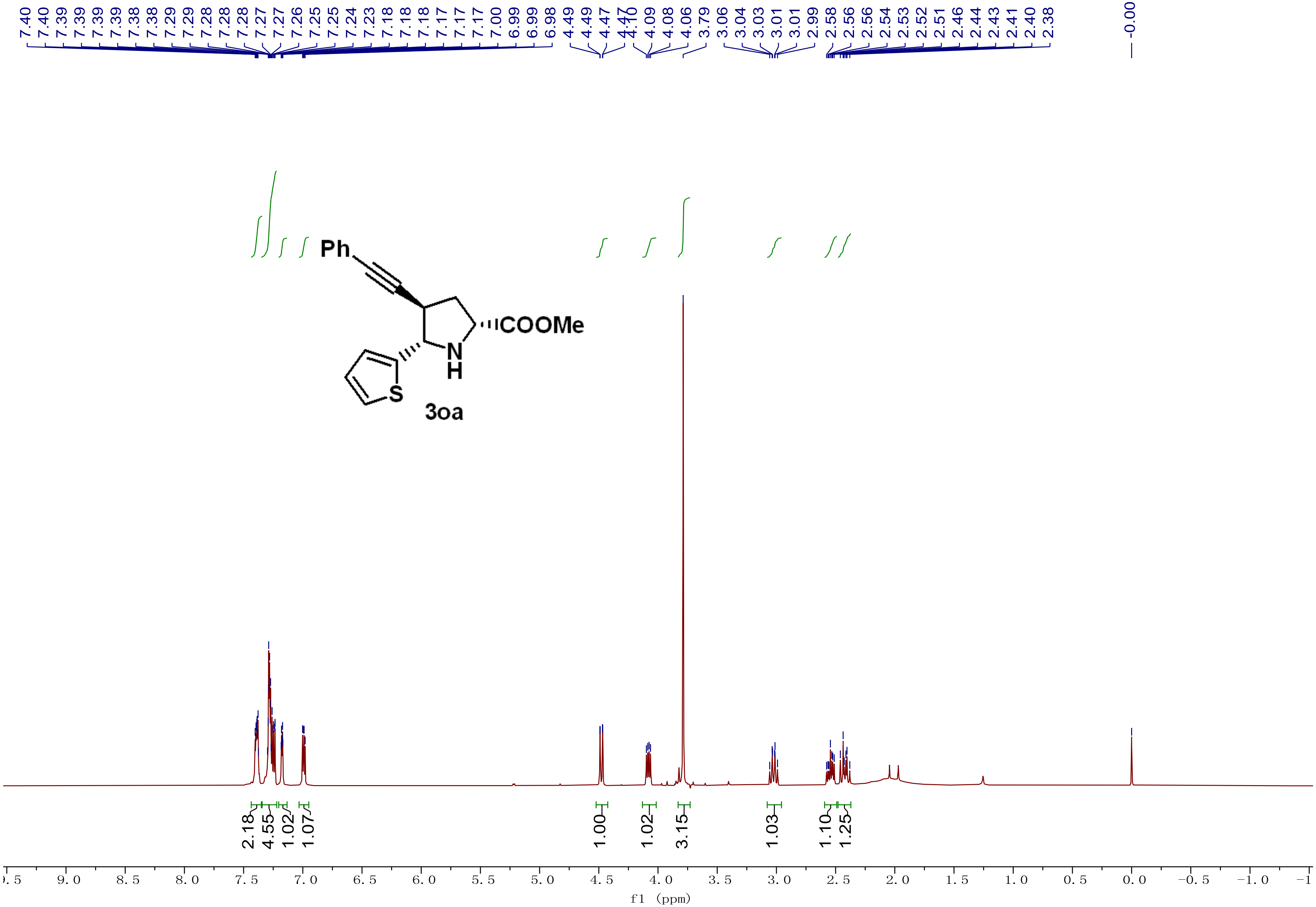
7.40 7.40 7.39 7.39 7.39 7.39 7.38 7.38 7.29 7.29 7.28 7.28 7.28 7.27 7.27 7.26 7.25 7.25 7.24 7.23 7.18 7.18 7.18 7.17 7.17 7.17 7.00 6.99 6.99 6.98 4.49 4.49 4.47 4.47 4.10 4.09 4.08 4.06 3.79 3.06 3.04 3.03 3.01 3.01 2.99 2.58 2.56 2.56 2.54 2.53 2.52 2.51 2.46 2.44 2.43 2.41 2.40 2.38
-0.00
Ph
COOMe
N
H
S
3oa
2.18 4.55 1.02 1.07 1.00 1.02 3.15 1.03 1.10 1.25
9.0 8.5 8.0 7.5 7.0 6.5 6.0 5.5 5.0 4.5 4.0 3.5 3.0 2.5 2.0 1.5 1.0 0.5 0.0 −0.5 −1.0
f1 (ppm)

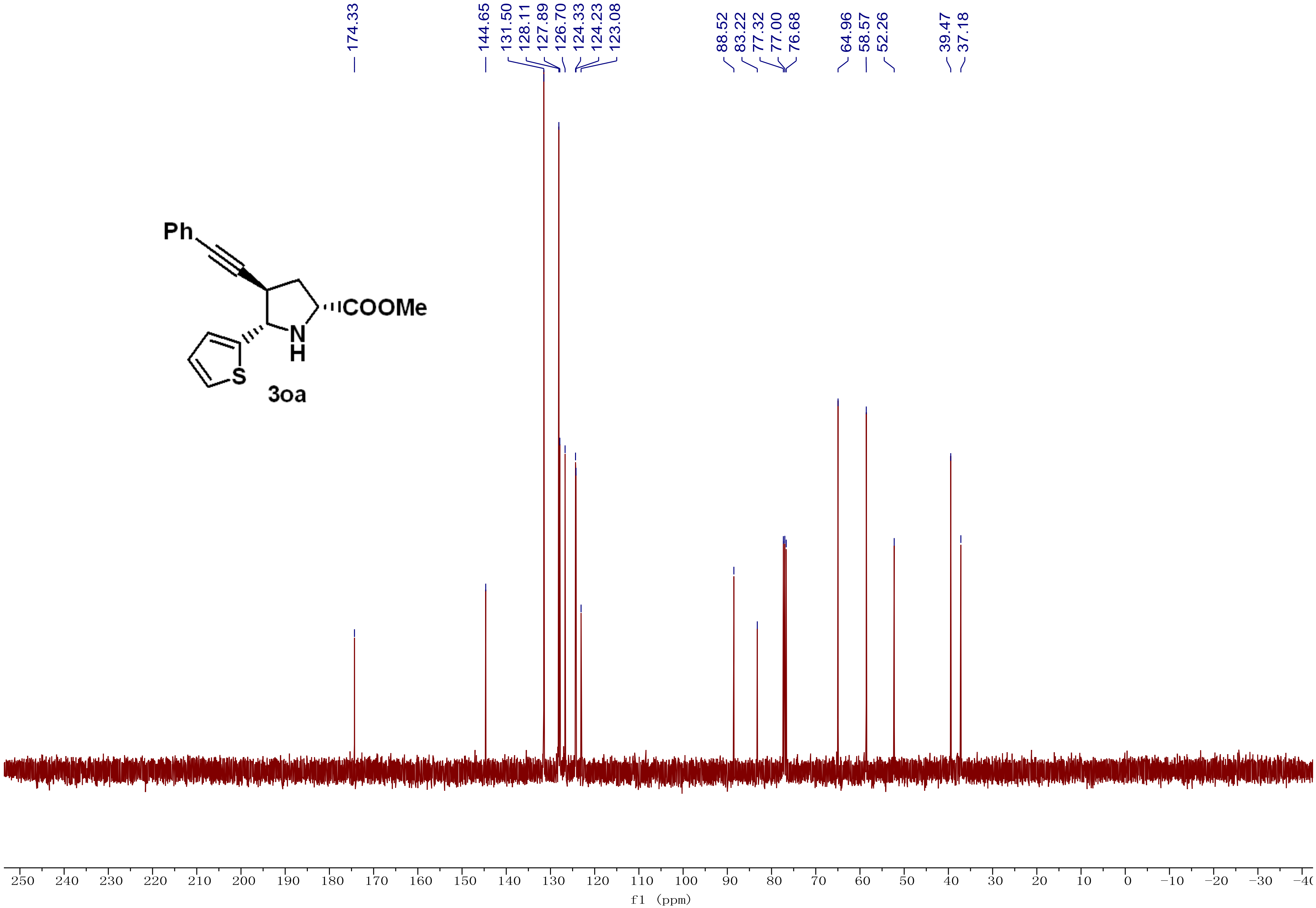
174.33
144.65
131.50
128.11
127.89
126.70
124.33
124.23
123.08
88.52
83.22
77.32
77.00
76.68
64.96
58.57
52.26
39.47
37.18
Ph
COOMe
N
H
S
3oa
250 240 230 220 210 200 190 180 170 160 150 140 130 120 110 100 90 80 70 60 50 40 30 20 10 0 −10 −20 −30 −40
f1 (ppm)

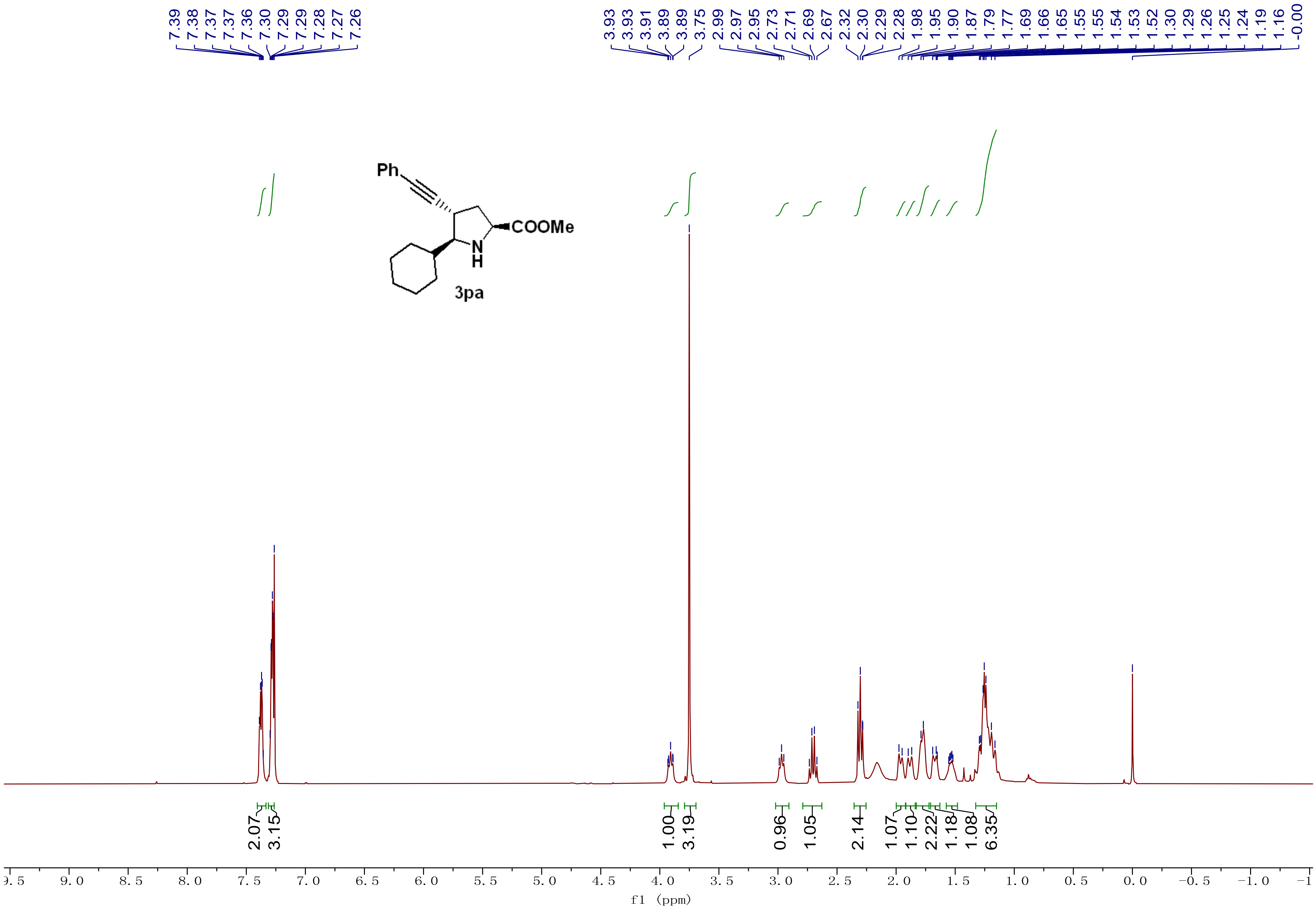

7.39 7.38 7.37 7.37 7.36 7.30 7.29 7.29 7.28 7.27 7.26
3.93 3.93 3.91 3.89 3.89 3.75 2.99 2.97 2.95 2.73 2.71 2.69 2.67 2.32 2.30 2.29 2.28 1.98 1.95 1.90 1.87 1.79 1.77 1.69 1.66 1.65 1.55 1.55 1.54 1.53 1.52 1.30 1.29 1.26 1.25 1.24 1.19 1.16 -0.00
Ph
COOMe
N
H
3pa
2.07 3.15 1.00 3.19 0.96 1.05 2.14 1.07 1.10 2.22 1.18 1.08 6.35
9.5 9.0 8.5 8.0 7.5 7.0 6.5 6.0 5.5 5.0 4.5 4.0 3.5 3.0 2.5 2.0 1.5 1.0 0.5 0.0 −0.5 −1.0 −1
f1 (ppm)

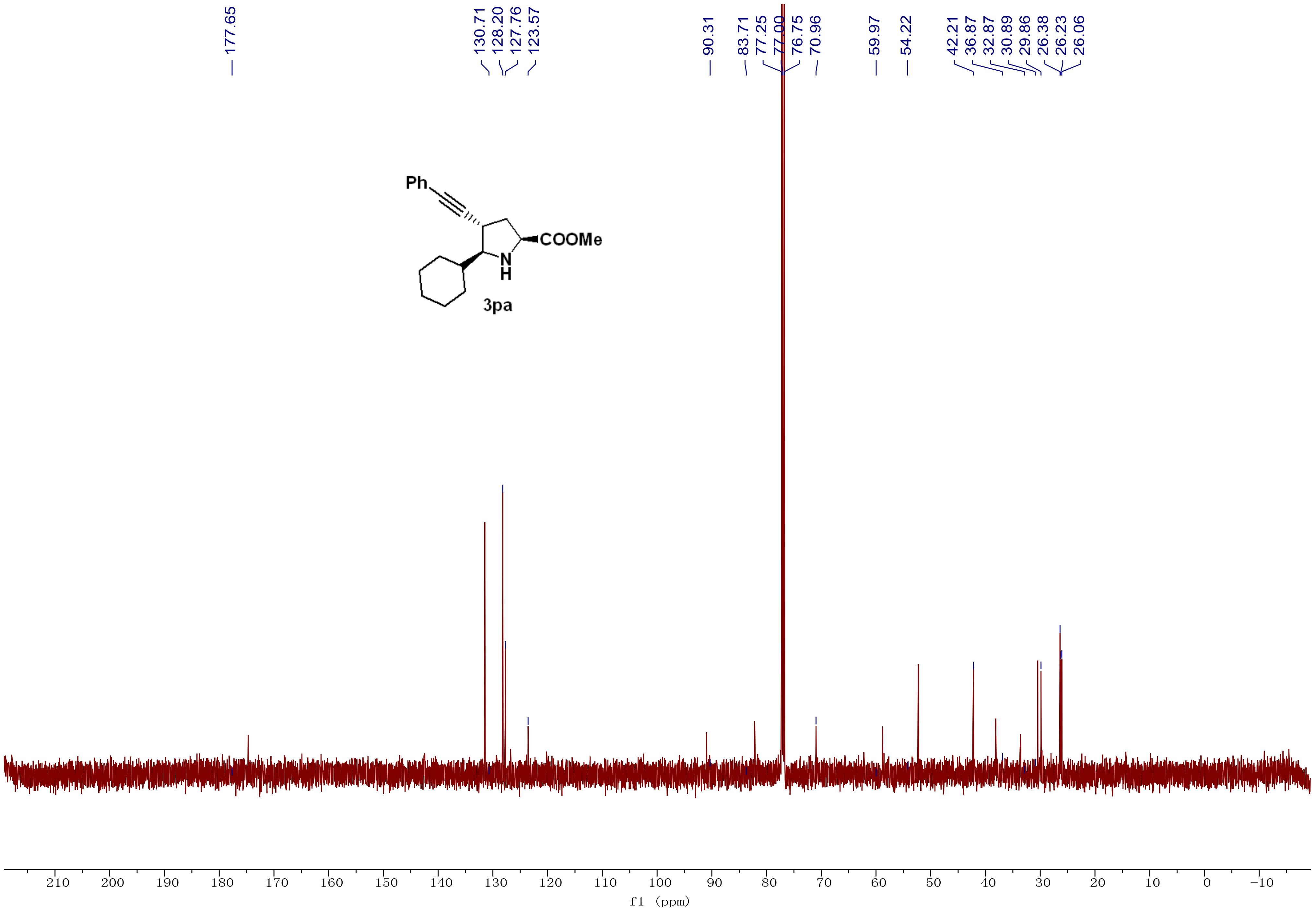
177.65
130.71
128.20
127.76
123.57
90.31
83.71
77.25
77.00
76.75
70.96
59.97
54.22
42.21
36.87
32.87
30.89
29.86
26.38
26.23
26.06
Ph
COOMe
N
H
3pa
210
200
190
180
170
160
150
140
130
120
110
100
90
80
70
60
50
40
30
20
10
0
-10
f1 (ppm)

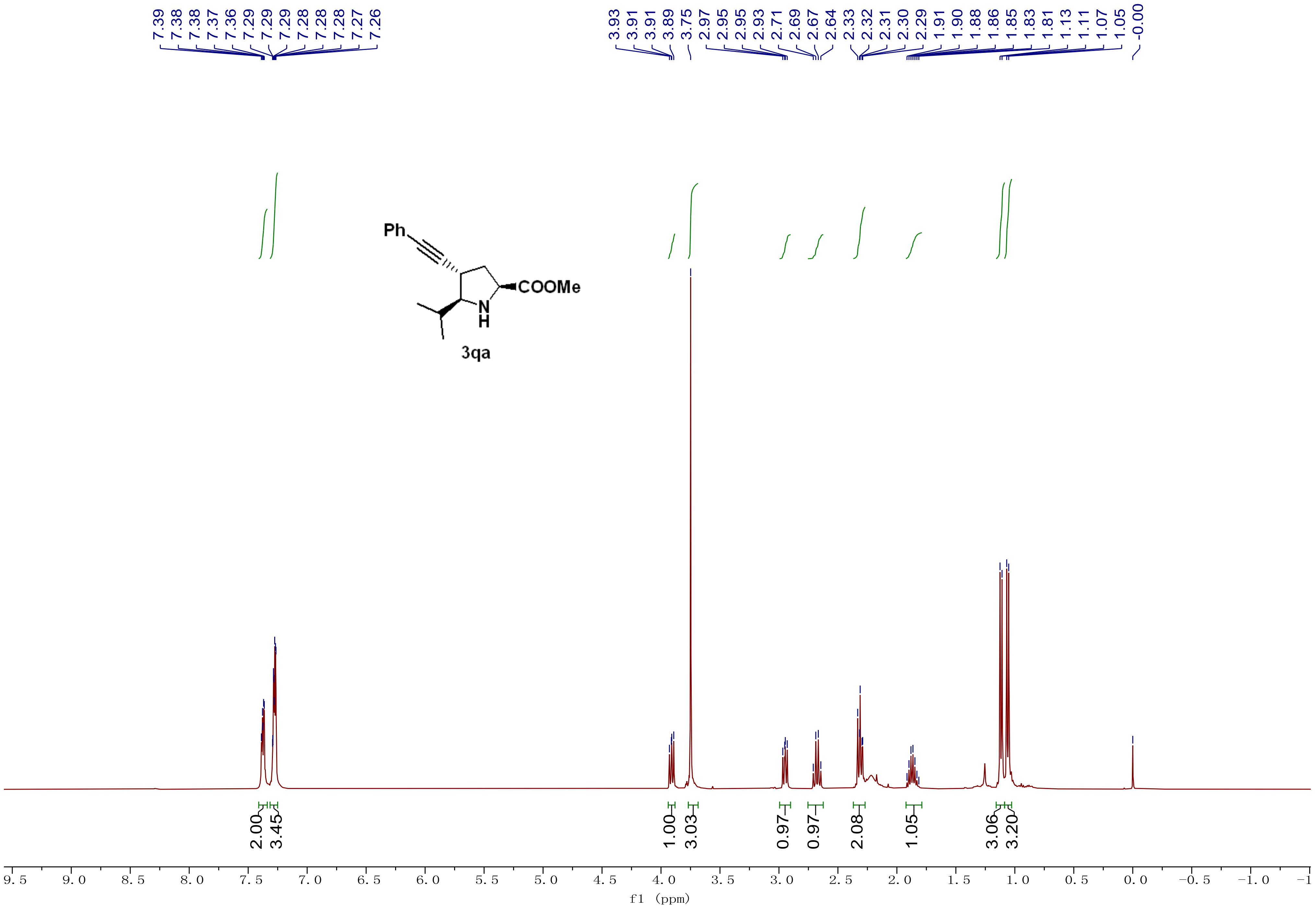
7.39
7.38
7.38
7.37
7.36
7.29
7.29
7.29
7.28
7.28
7.28
7.27
7.26
3.93
3.91
3.91
3.89
3.75
2.97
2.95
2.95
2.93
2.71
2.69
2.67
2.64
2.33
2.32
2.31
2.30
2.29
1.91
1.90
1.88
1.86
1.85
1.83
1.81
1.13
1.11
1.07
1.05
-0.00
Ph
COOMe
N
H
3qa
2.00
3.45
1.00
3.03
0.97
0.97
2.08
1.05
3.06
3.20
f1 (ppm)

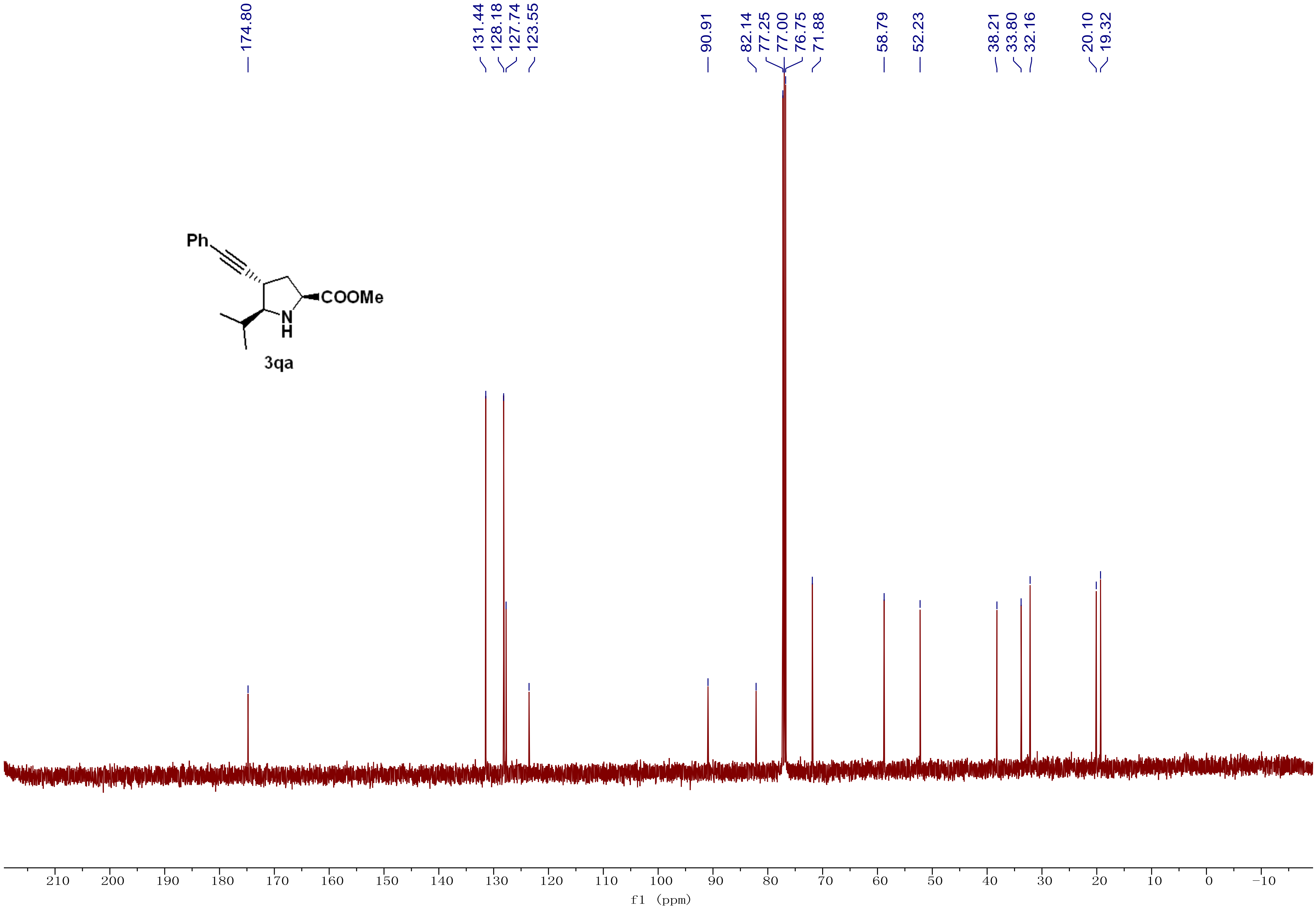
174.80
131.44
128.18
127.74
123.55
90.91
82.14
77.25
77.00
76.75
71.88
58.79
52.23
38.21
33.80
32.16
20.10
19.32
Ph
COOMe
N
H
3qa
210 200 190 180 170 160 150 140 130 120 110 100 90 80 70 60 50 40 30 20 10 0 -10
f1 (ppm)

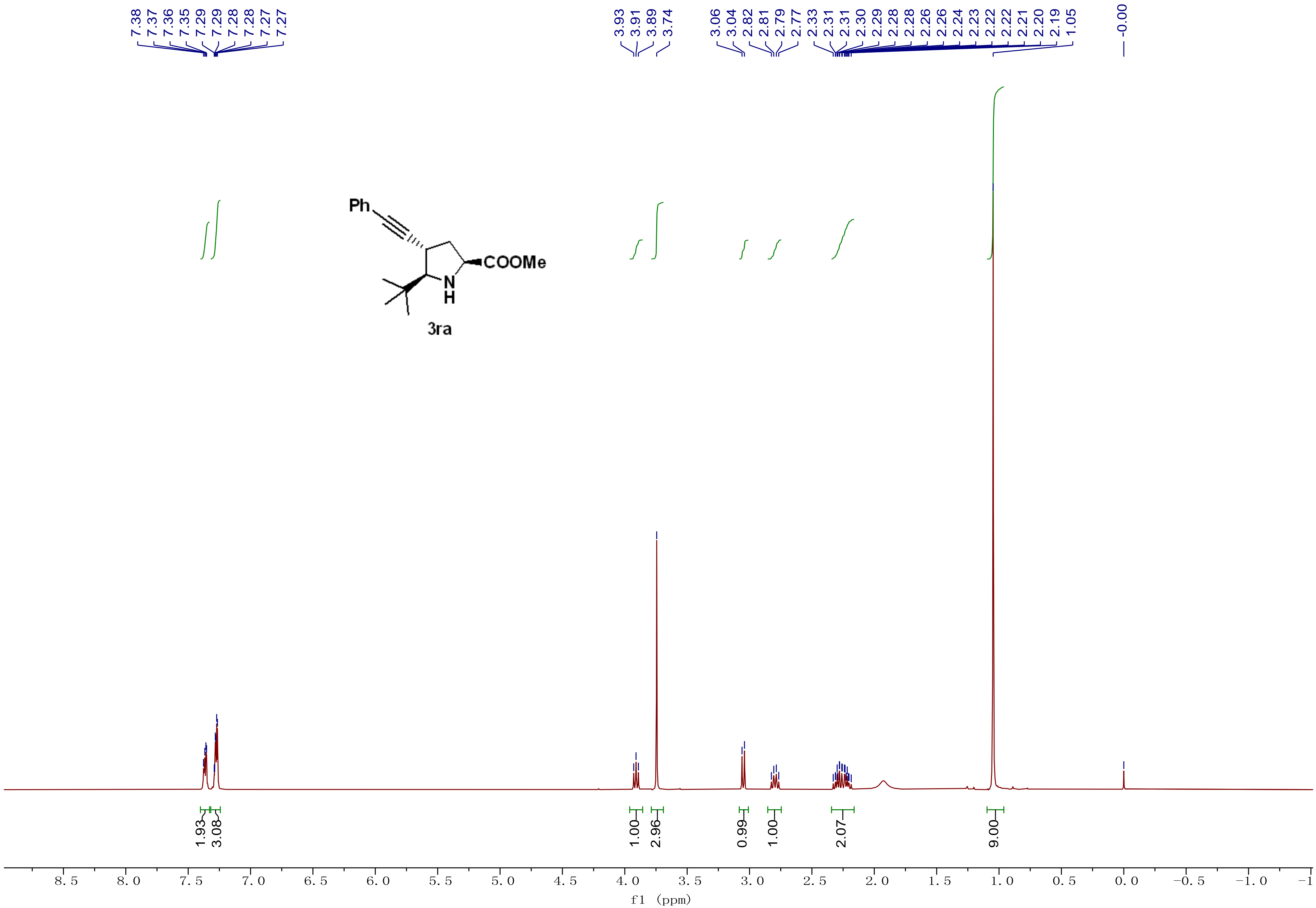
7.38 7.37 7.36 7.35 7.29 7.29 7.28 7.28 7.27 7.27
3.93 3.91 3.89 3.74
3.06 3.04 2.82 2.81 2.79 2.77 2.33 2.31 2.31 2.30 2.29 2.28 2.28 2.26 2.26 2.24 2.23 2.22 2.22 2.21 2.20 2.19 1.05
-0.00
Ph
COOMe
N
H
3ra
1.93 3.08 1.00 2.96 0.99 1.00 2.07 9.00
8.5 8.0 7.5 7.0 6.5 6.0 5.5 5.0 4.5 4.0 3.5 3.0 2.5 2.0 1.5 1.0 0.5 0.0 -0.5 -1.0 -1
f1 (ppm)

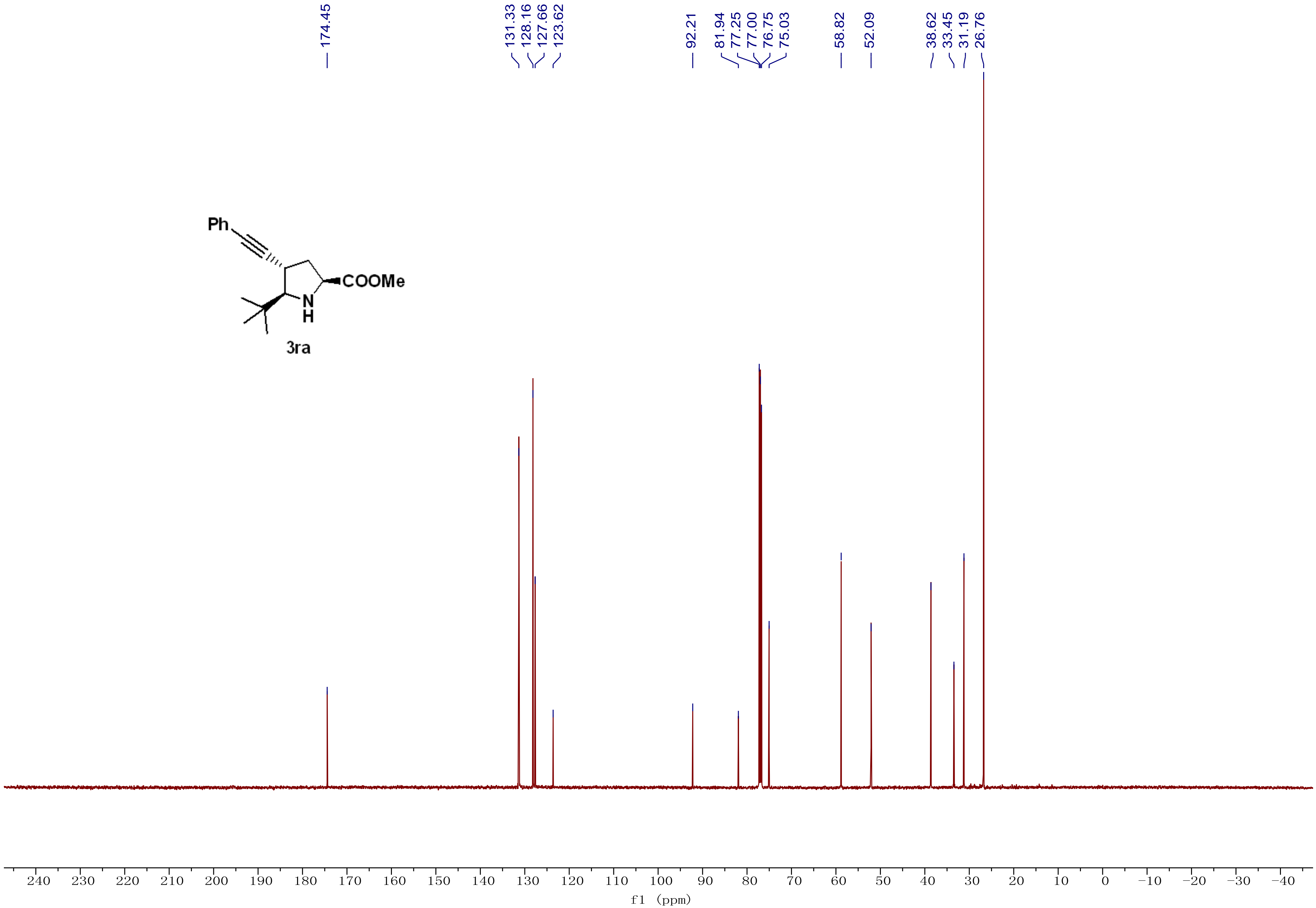

174.45
131.33
128.16
127.66
123.62
92.21
81.94
77.25
77.00
76.75
75.03
58.82
52.09
38.62
33.45
31.19
26.76
Ph
COOMe
N
H
3ra
240 230 220 210 200 190 180 170 160 150 140 130 120 110 100 90 80 70 60 50 40 30 20 10 0 −10 −20 −30 −40
f1 (ppm)

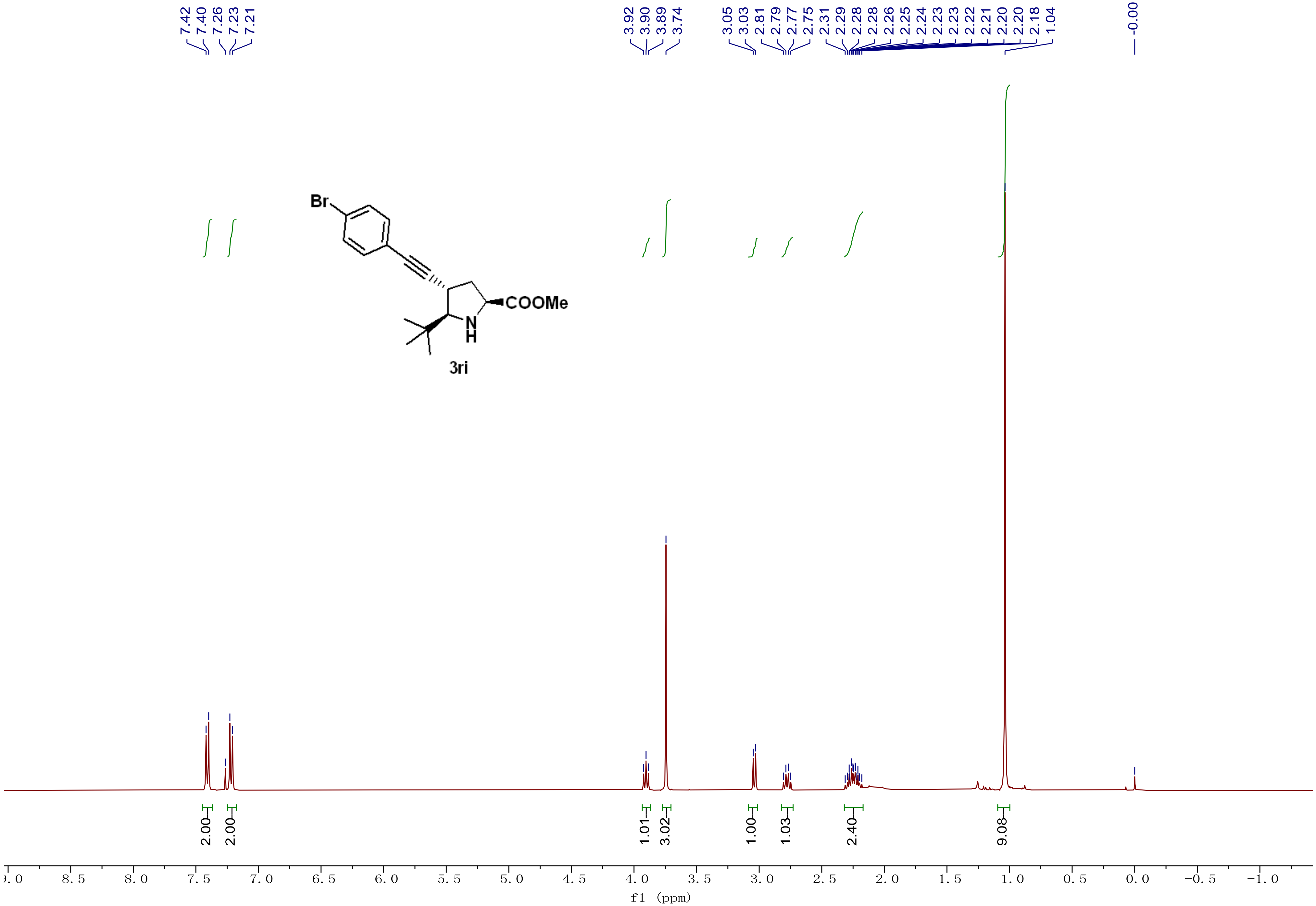
7.42
7.40
7.26
7.23
7.21
3.92
3.90
3.89
3.74
3.05
3.03
2.81
2.79
2.77
2.75
2.31
2.29
2.28
2.28
2.26
2.25
2.24
2.23
2.23
2.22
2.21
2.20
2.20
2.18
1.04
-0.00
Br
COOMe
N
H
3ri
2.00
2.00
1.01
3.02
1.00
1.03
2.40
9.08
f1 (ppm)

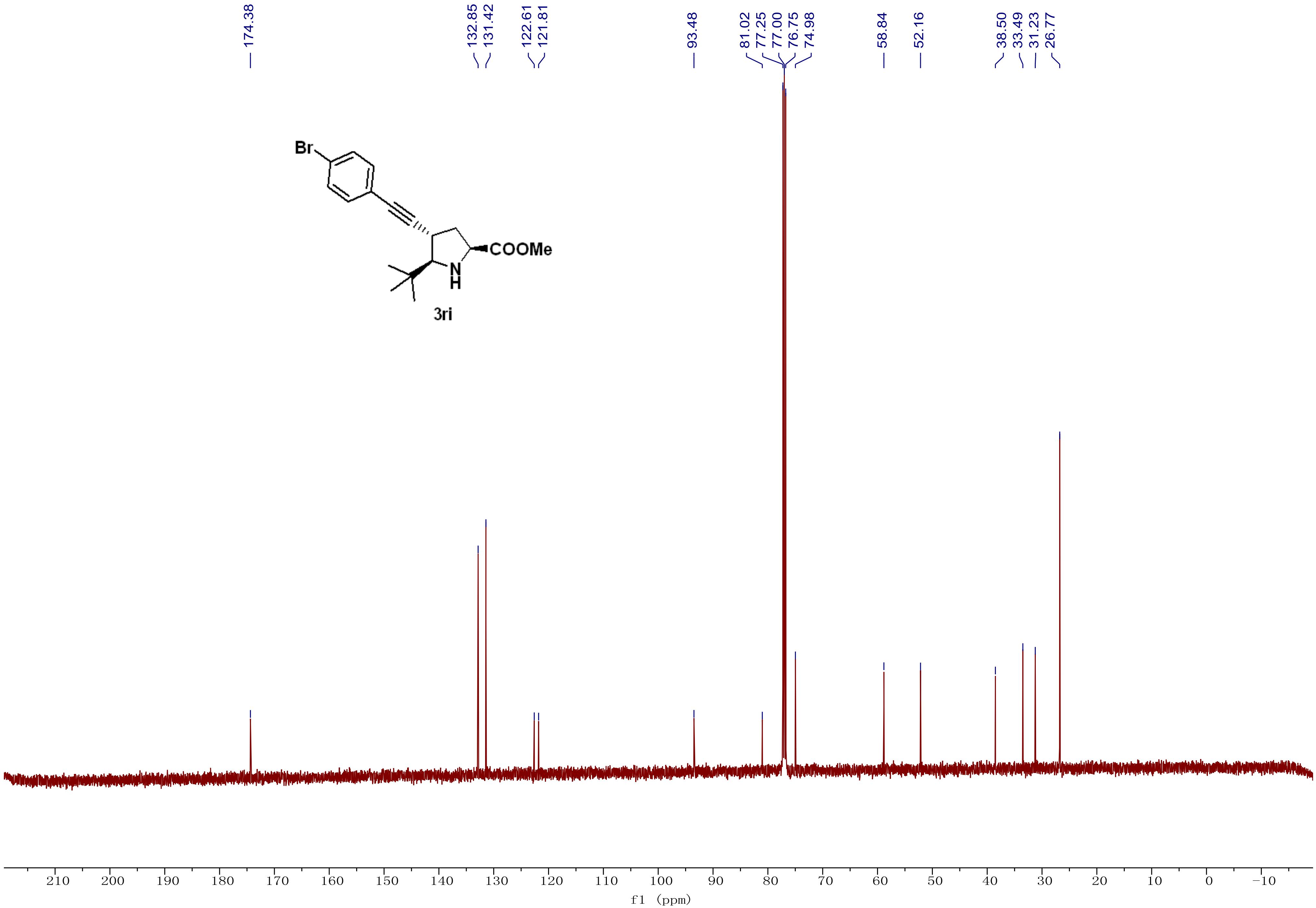
174.38
132.85
131.42
122.61
121.81
93.48
81.02
77.25
77.00
76.75
74.98
58.84
52.16
38.50
33.49
31.23
26.77
Br
COOMe
N
H
3ri
210 200 190 180 170 160 150 140 130 120 110 100 90 80 70 60 50 40 30 20 10 0 −10
f1 (ppm)

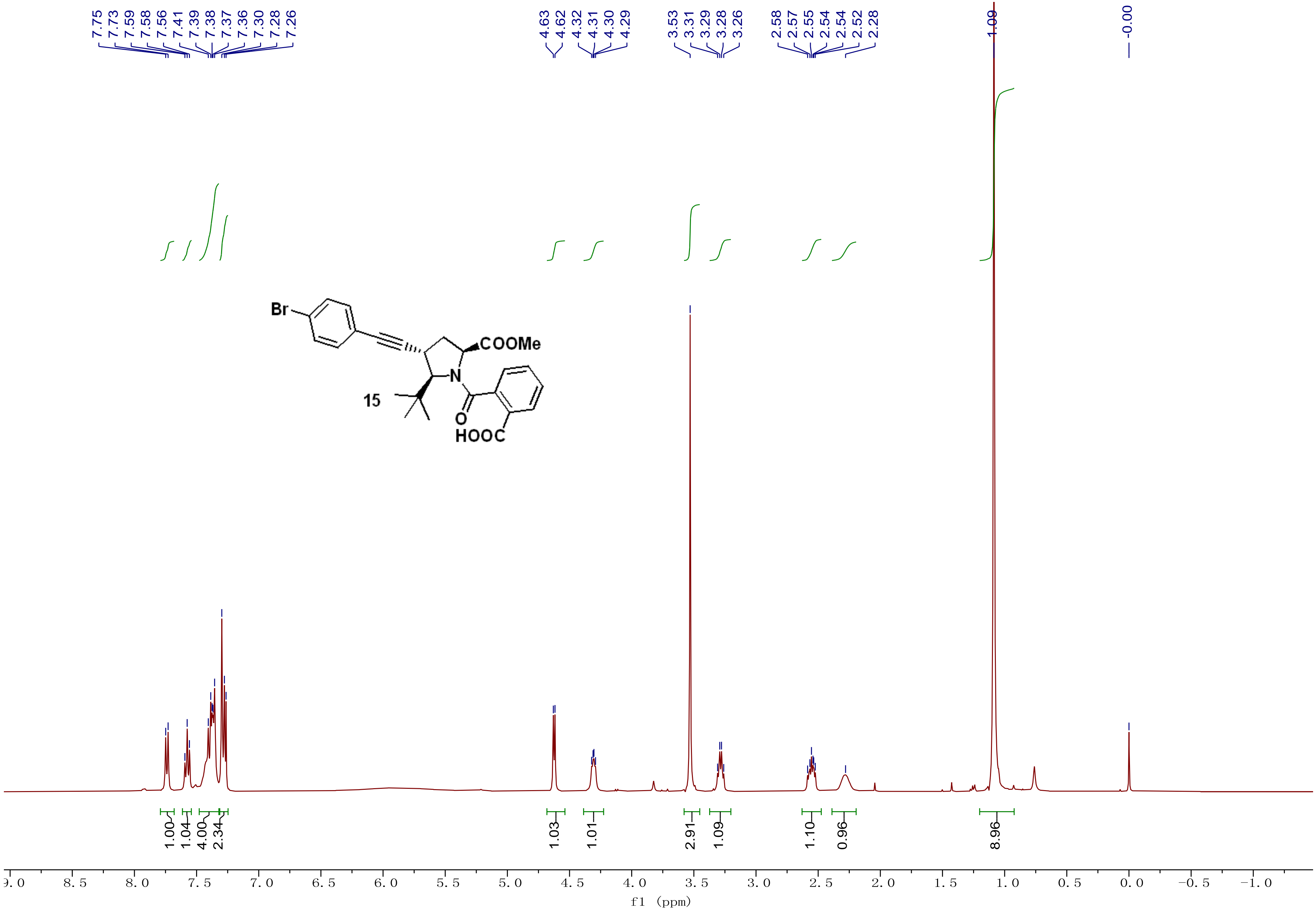
7.75
7.73
7.59
7.58
7.56
7.41
7.39
7.38
7.37
7.36
7.30
7.28
7.26
4.63
4.62
4.32
4.31
4.30
4.29
3.53
3.31
3.29
3.28
3.26
2.58
2.57
2.55
2.54
2.54
2.52
2.28
1.09
-0.00
Br
COOMe
N
O
HOOC
15
1.00
1.04
4.00
2.34
1.03
1.01
2.91
1.09
1.10
0.96
8.96
9.0
8.5
8.0
7.5
7.0
6.5
6.0
5.5
5.0
4.5
4.0
3.5
3.0
2.5
2.0
1.5
1.0
0.5
0.0
−0.5
−1.0
f1 (ppm)

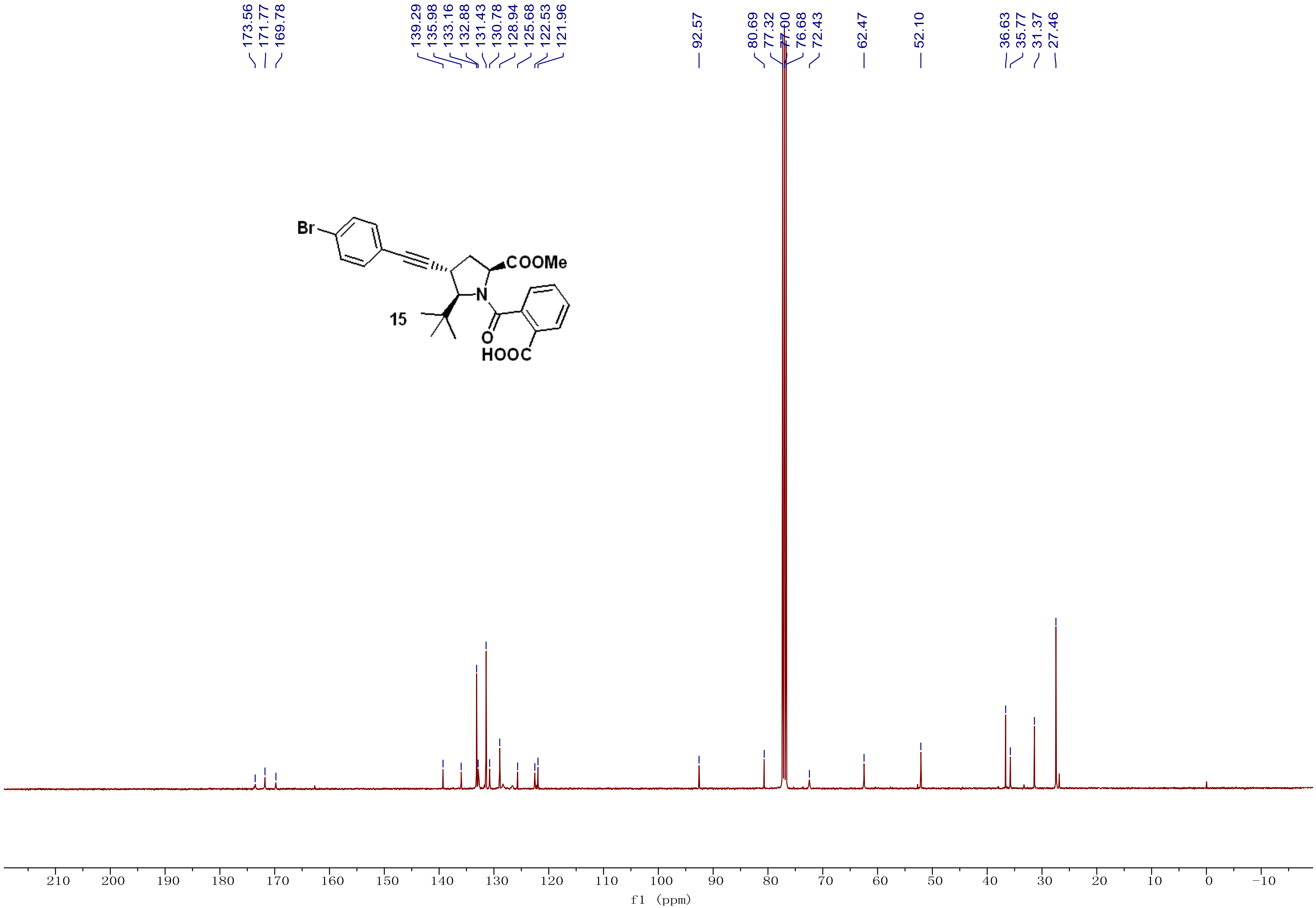

173.56
171.77
169.78
139.29
135.98
133.16
132.88
131.43
130.78
128.94
125.68
122.53
121.96
92.57
80.69
77.32
77.00
76.68
72.43
62.47
52.10
36.63
35.77
31.37
27.46
Br
COOMe
N
O
HOOC
15
210
200
190
180
170
160
150
140
130
120
110
100
90
80
70
60
50
40
30
20
10
0
−10
f1 (ppm)

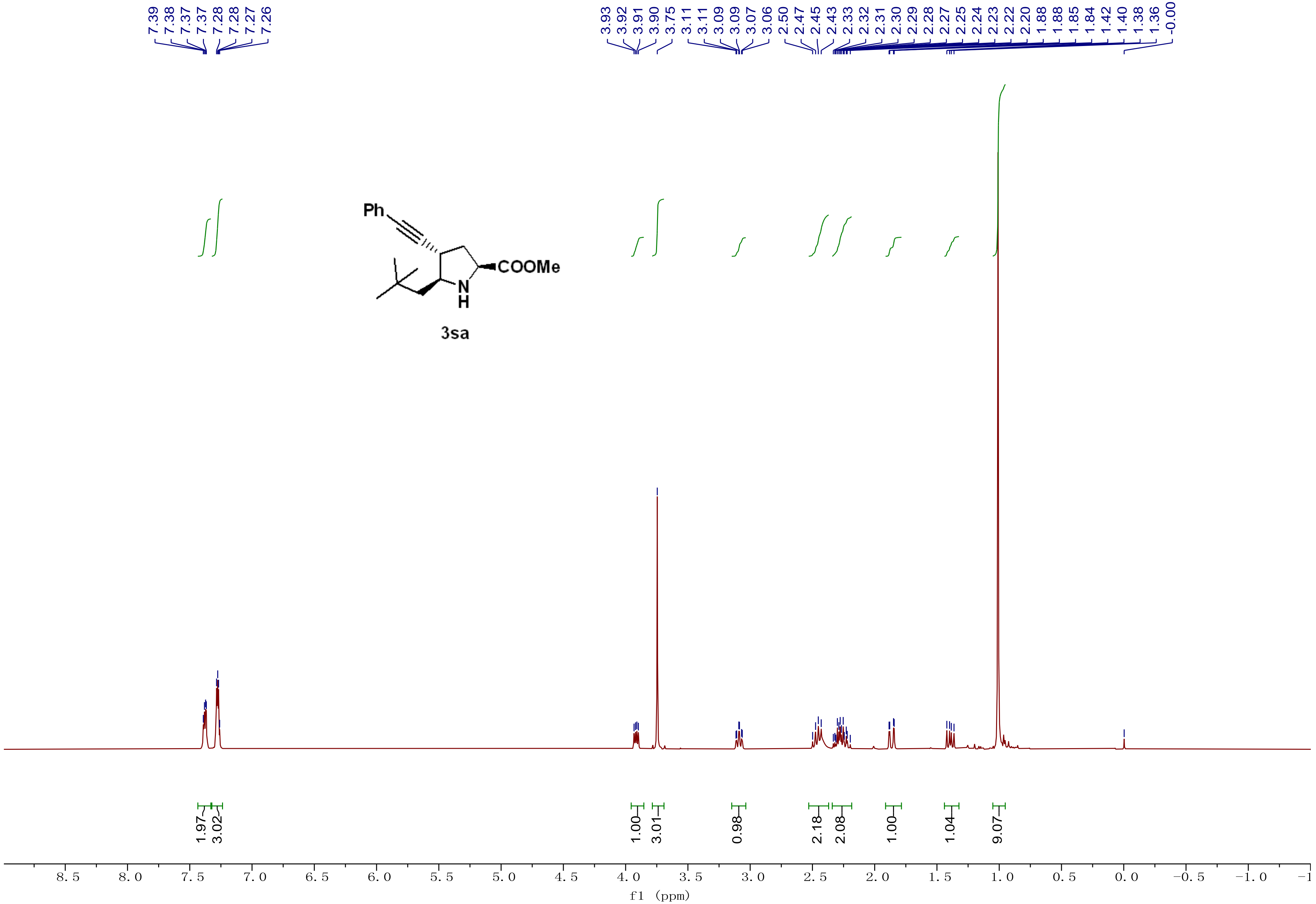
7.39 7.38 7.37 7.37 7.28 7.28 7.27 7.26
3.93 3.92 3.91 3.90 3.75 3.11 3.11 3.09 3.09 3.07 3.06 2.50 2.47 2.45 2.43 2.33 2.32 2.31 2.30 2.29 2.28 2.27 2.25 2.24 2.23 2.22 2.20 1.88 1.88 1.85 1.84 1.42 1.40 1.38 1.36 -0.00
Ph
COOMe
N
H
3sa
1.97 3.02 1.00 3.01 0.98 2.18 2.08 1.00 1.04 9.07
8.5 8.0 7.5 7.0 6.5 6.0 5.5 5.0 4.5 4.0 3.5 3.0 2.5 2.0 1.5 1.0 0.5 0.0 -0.5 -1.0 -1
f1 (ppm)

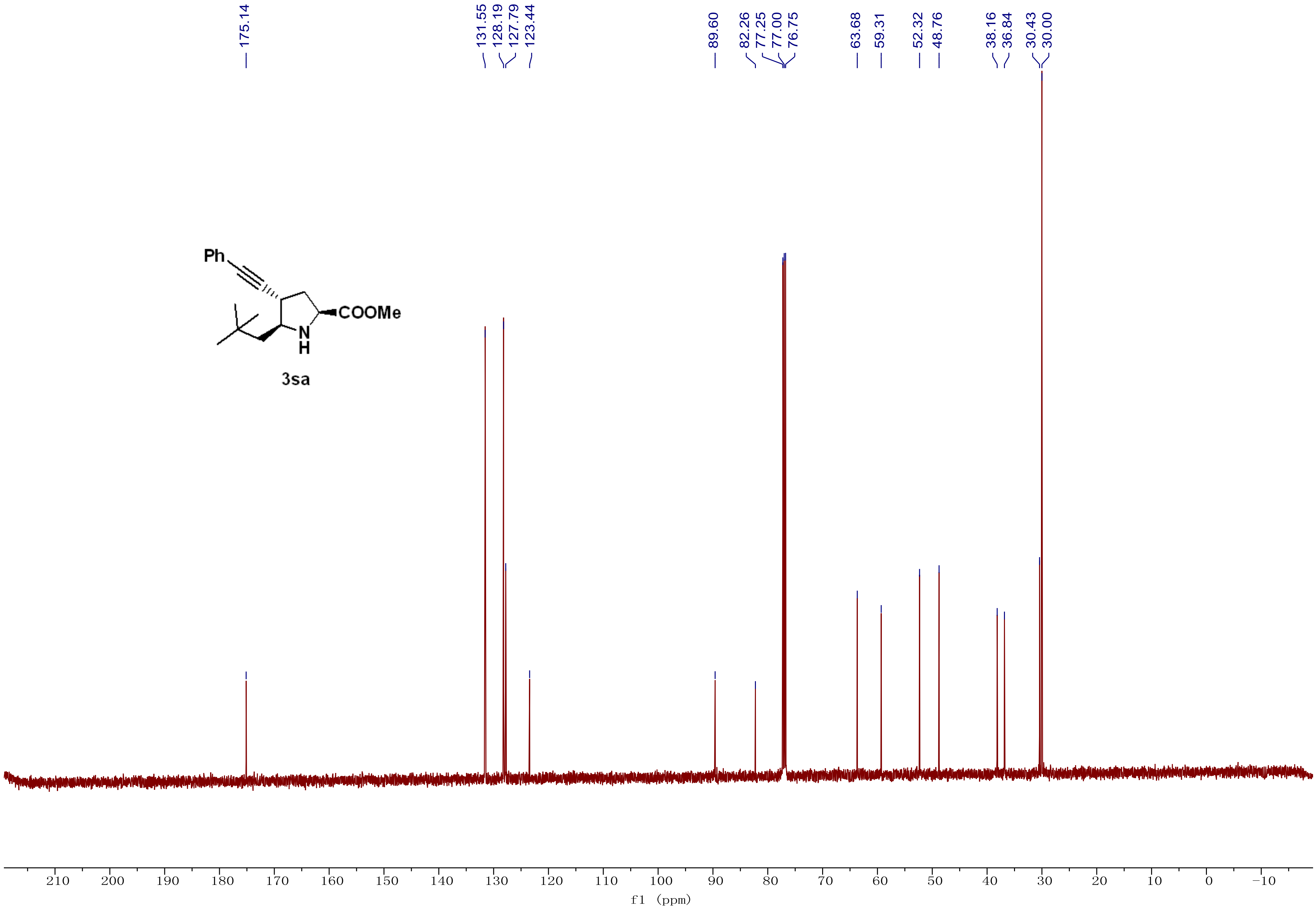
175.14
131.55
128.19
127.79
123.44
89.60
82.26
77.25
77.00
76.75
63.68
59.31
52.32
48.76
38.16
36.84
30.43
30.00
Ph
COOMe
N
H
3sa
210 200 190 180 170 160 150 140 130 120 110 100 90 80 70 60 50 40 30 20 10 0 −10
f1 (ppm)

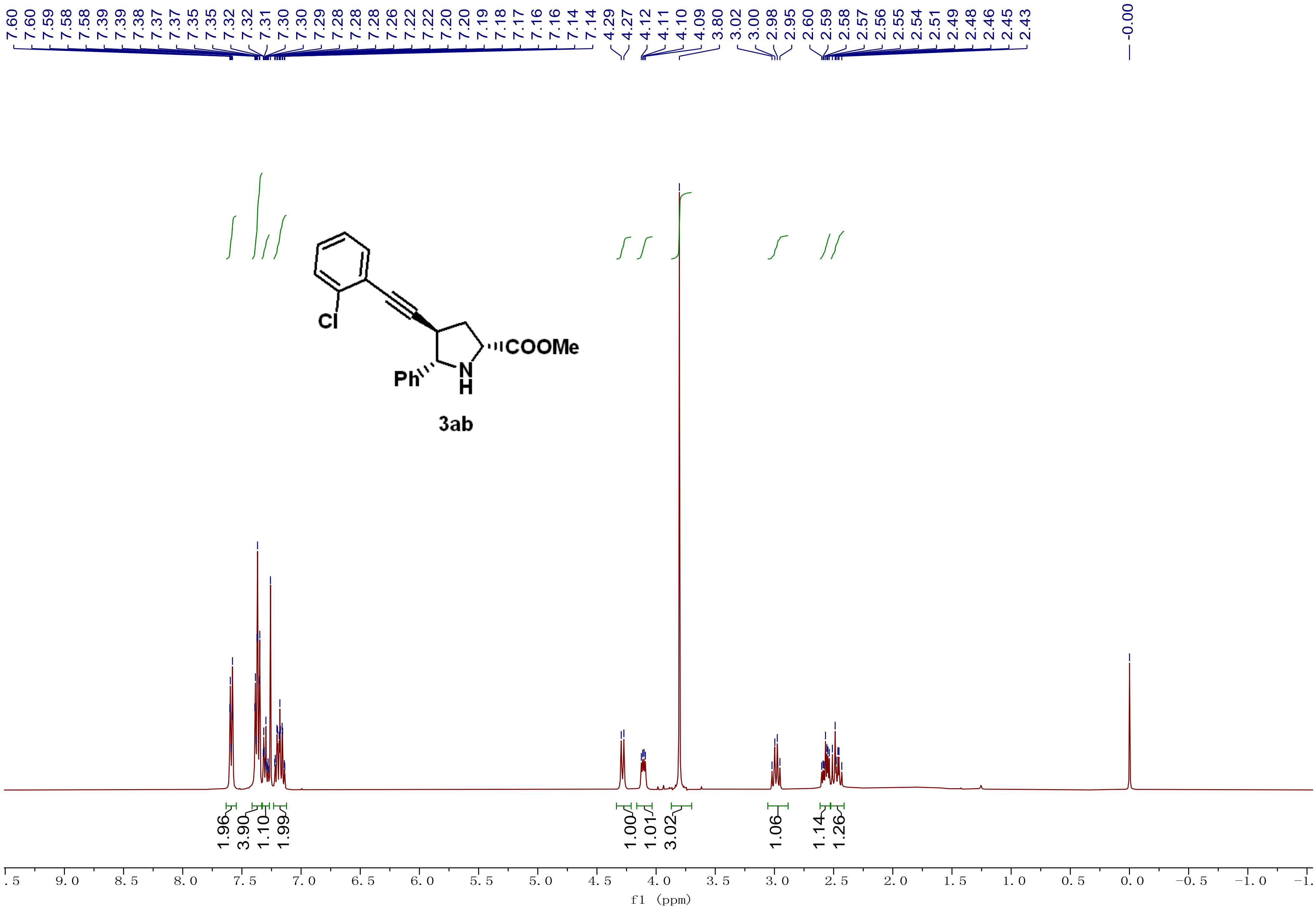
7.60 7.60 7.59 7.58 7.58 7.39 7.39 7.38 7.37 7.37 7.35 7.35 7.32 7.32 7.31 7.30 7.30 7.29 7.28 7.28 7.28 7.26 7.22 7.22 7.20 7.20 7.19 7.18 7.17 7.16 7.16 7.14 7.14 4.29 4.27 4.12 4.11 4.10 4.09 3.80 3.02 3.00 2.98 2.95 2.60 2.59 2.58 2.57 2.56 2.55 2.54 2.51 2.49 2.48 2.46 2.45 2.43
-0.00
Cl
COOMe
Ph
N
H
3ab
1.96 3.90 1.10 1.99 1.00 1.01 3.02 1.06 1.14 1.26
f1 (ppm)

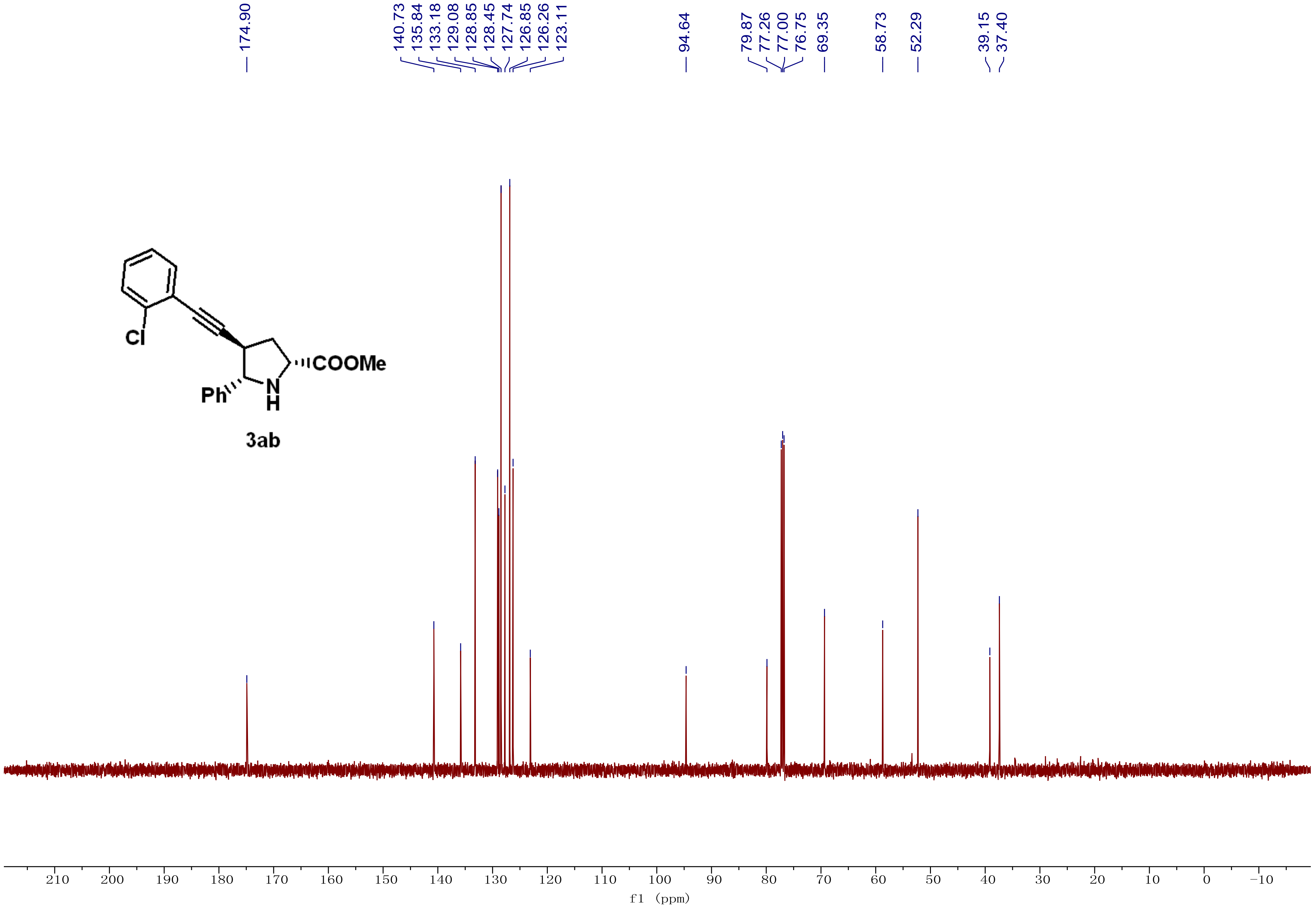
174.90
140.73
135.84
133.18
129.08
128.85
128.45
127.74
126.85
126.26
123.11
94.64
79.87
77.26
77.00
76.75
69.35
58.73
52.29
39.15
37.40
COOMe
Cl
Ph
N
H
3ab
210
200
190
180
170
160
150
140
130
120
110
100
90
80
70
60
50
40
30
20
10
0
−10
f1 (ppm)

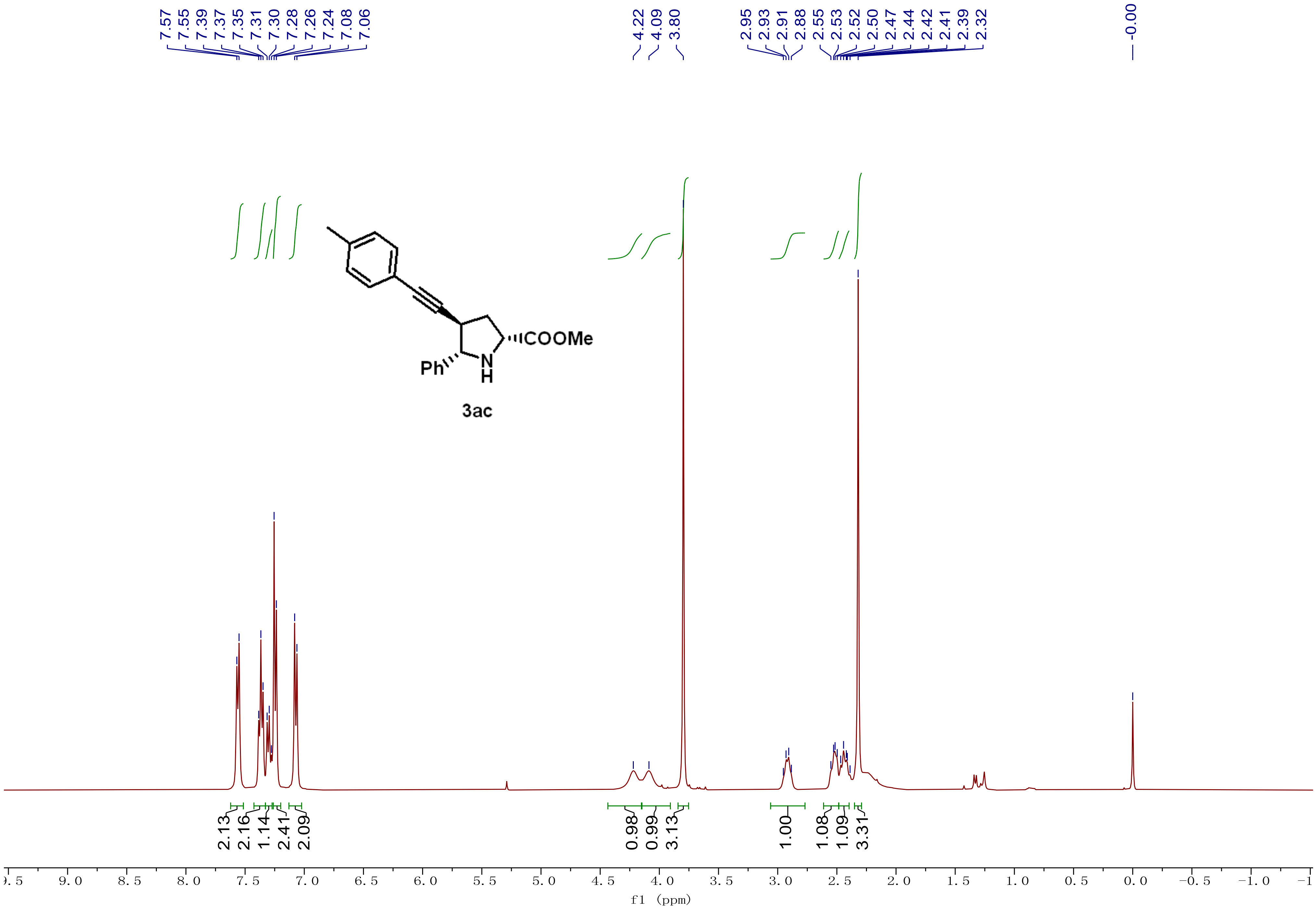
7.57
7.55
7.39
7.37
7.35
7.31
7.30
7.28
7.26
7.24
7.08
7.06
4.22
4.09
3.80
2.95
2.93
2.91
2.88
2.55
2.53
2.52
2.50
2.47
2.44
2.42
2.41
2.39
2.32
-0.00
COOMe
Ph
N
H
3ac
2.13
2.16
1.14
2.41
2.09
0.98
0.99
3.13
1.00
1.08
1.09
3.31
9.5
9.0
8.5
8.0
7.5
7.0
6.5
6.0
5.5
5.0
4.5
4.0
3.5
3.0
2.5
2.0
1.5
1.0
0.5
0.0
-0.5
-1.0
-1
f1 (ppm)

3ac

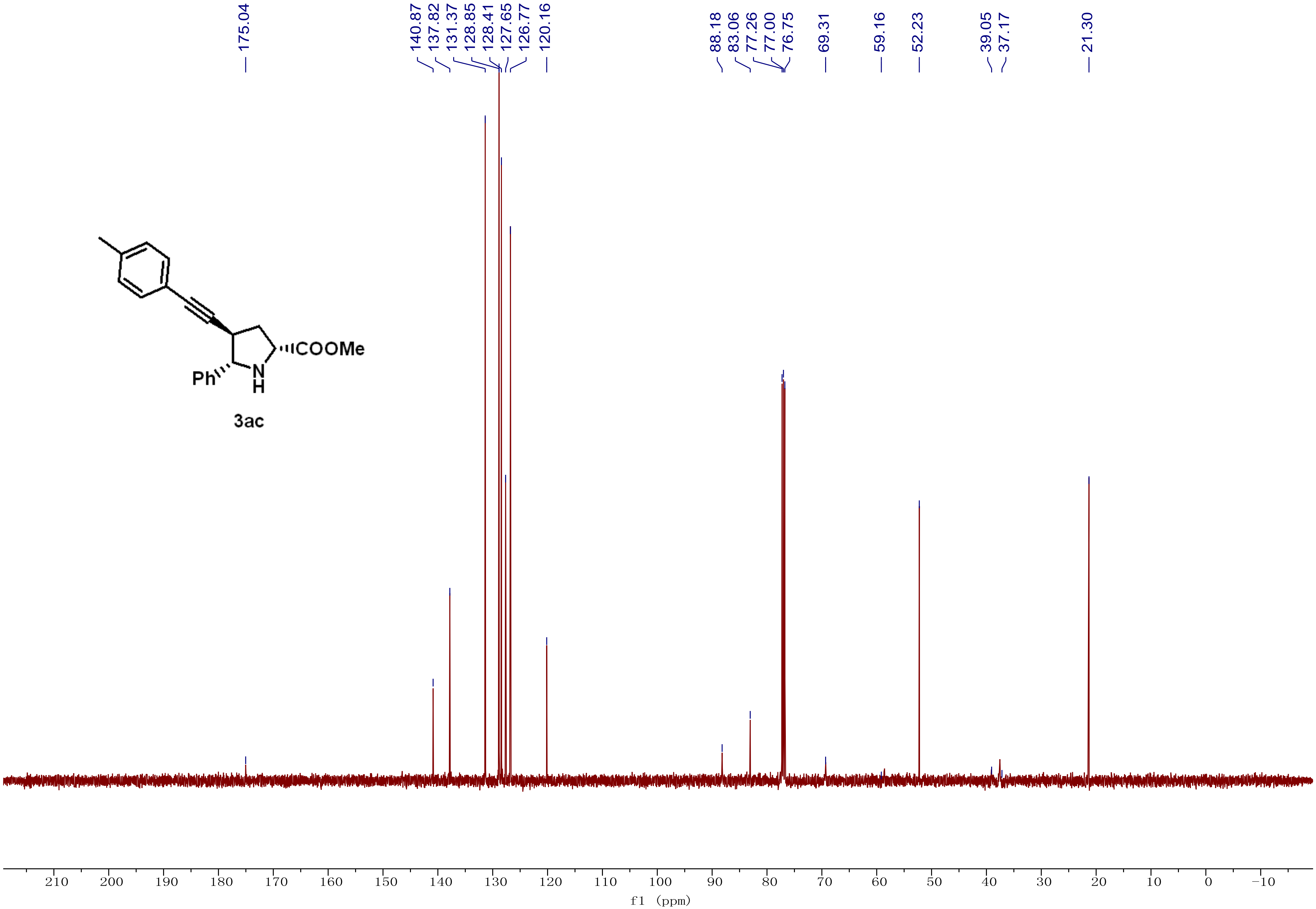
175.04
140.87
137.82
131.37
128.85
128.41
127.65
126.77
120.16
88.18
83.06
77.26
77.00
76.75
69.31
59.16
52.23
39.05
37.17
21.30
210
200
190
180
170
160
150
140
130
120
110
100
90
80
70
60
50
40
30
20
10
0
-10
f1 (ppm)

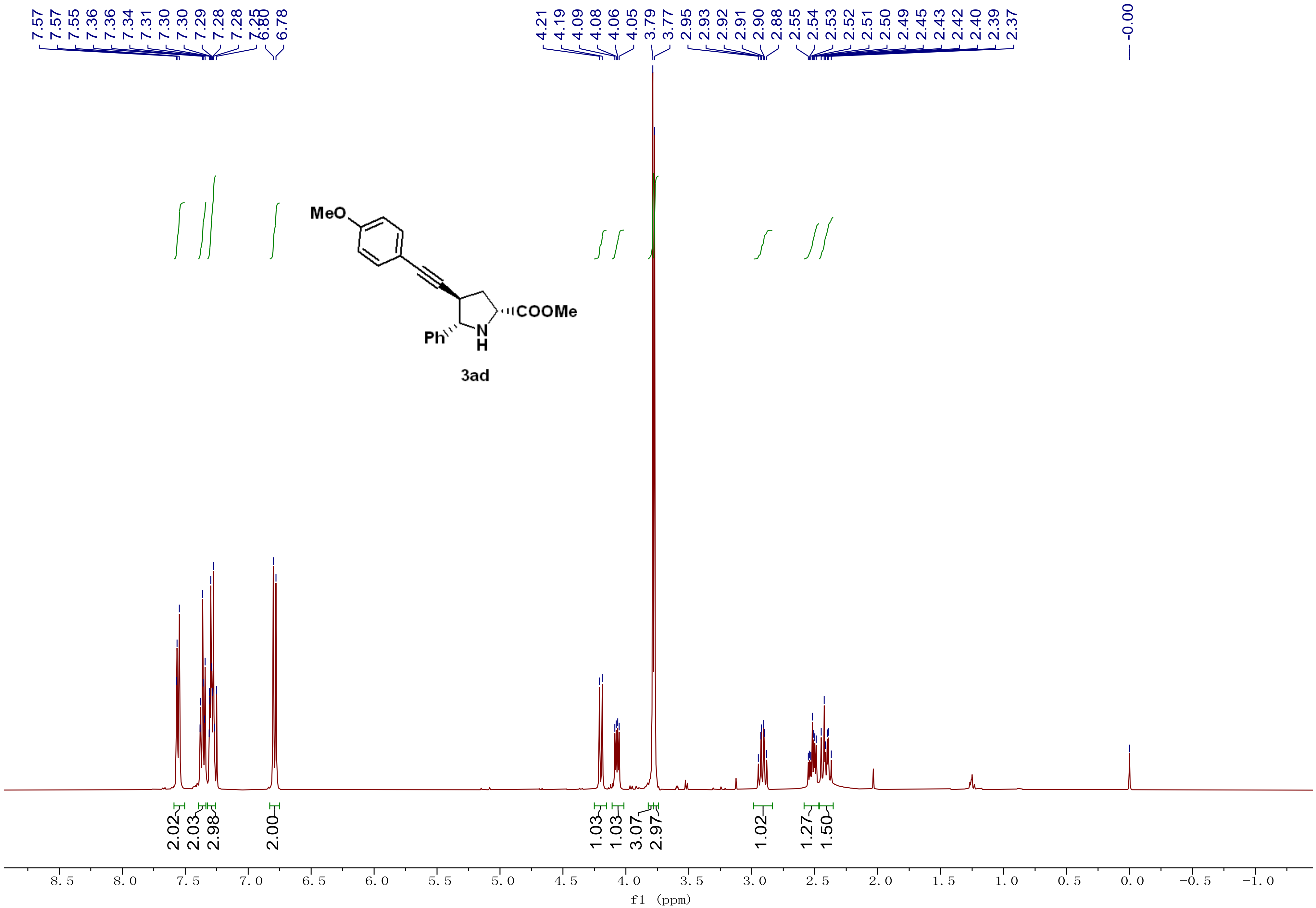
7.57 7.57 7.55 7.36 7.36 7.34 7.31 7.30 7.30 7.29 7.28 7.28 7.25 6.80 6.78
4.21 4.19 4.09 4.08 4.06 4.05 3.79 3.77 2.95 2.93 2.92 2.91 2.90 2.88 2.55 2.54 2.53 2.52 2.51 2.50 2.49 2.45 2.43 2.42 2.40 2.39 2.37
-0.00
MeO
COOMe
Ph
N
H
3ad
2.02 2.03 2.98 2.00 1.03 1.03 3.07 2.97 1.02 1.27 1.50
8.5 8.0 7.5 7.0 6.5 6.0 5.5 5.0 4.5 4.0 3.5 3.0 2.5 2.0 1.5 1.0 0.5 0.0 -0.5 -1.0
f1 (ppm)

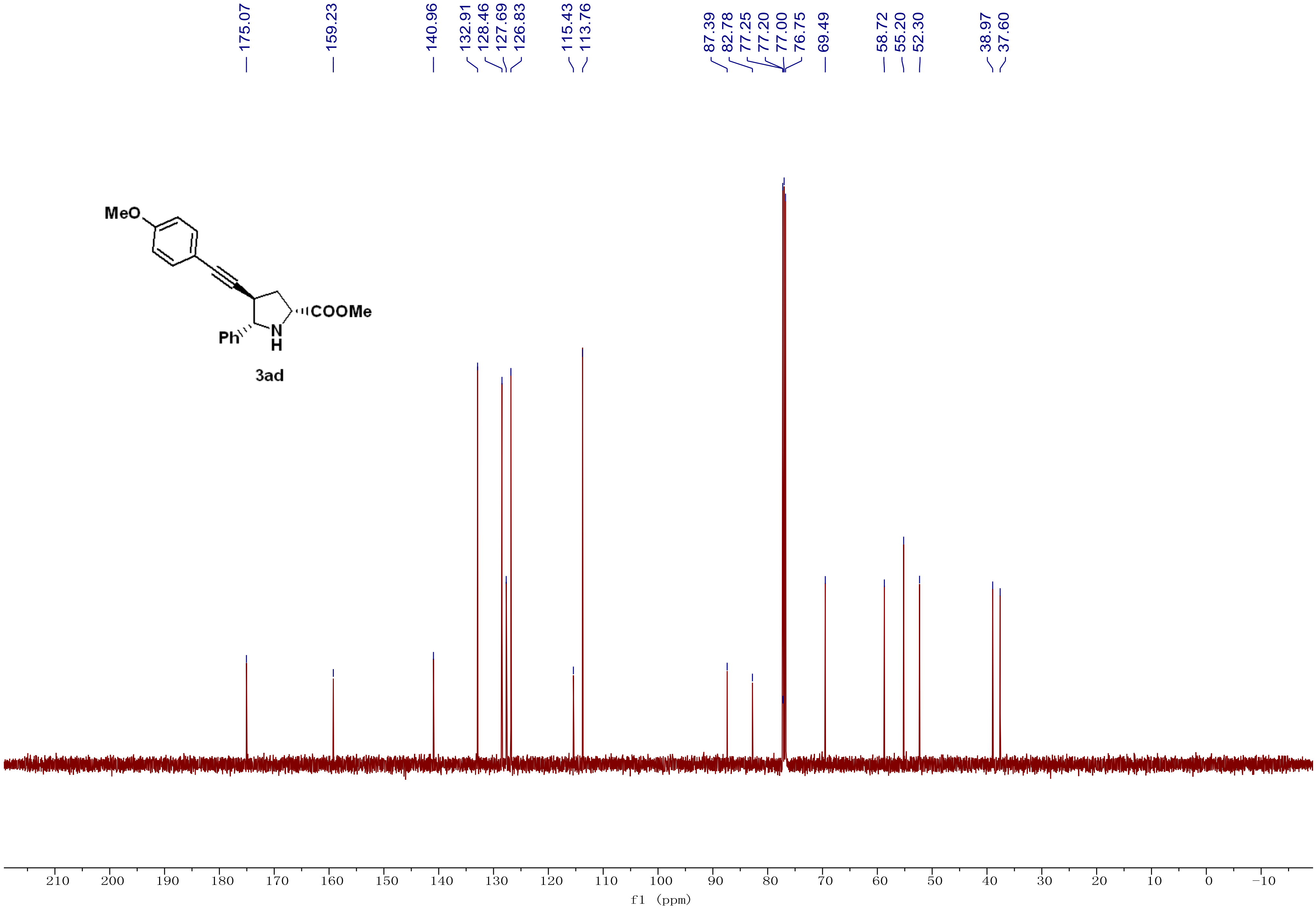
MeO
COOMe
Ph
N
H
3ad
175.07
159.23
140.96
132.91
128.46
127.69
126.83
115.43
113.76
87.39
82.78
77.25
77.20
77.00
76.75
69.49
58.72
55.20
52.30
38.97
37.60
210 200 190 180 170 160 150 140 130 120 110 100 90 80 70 60 50 40 30 20 10 0 -10
f1 (ppm)

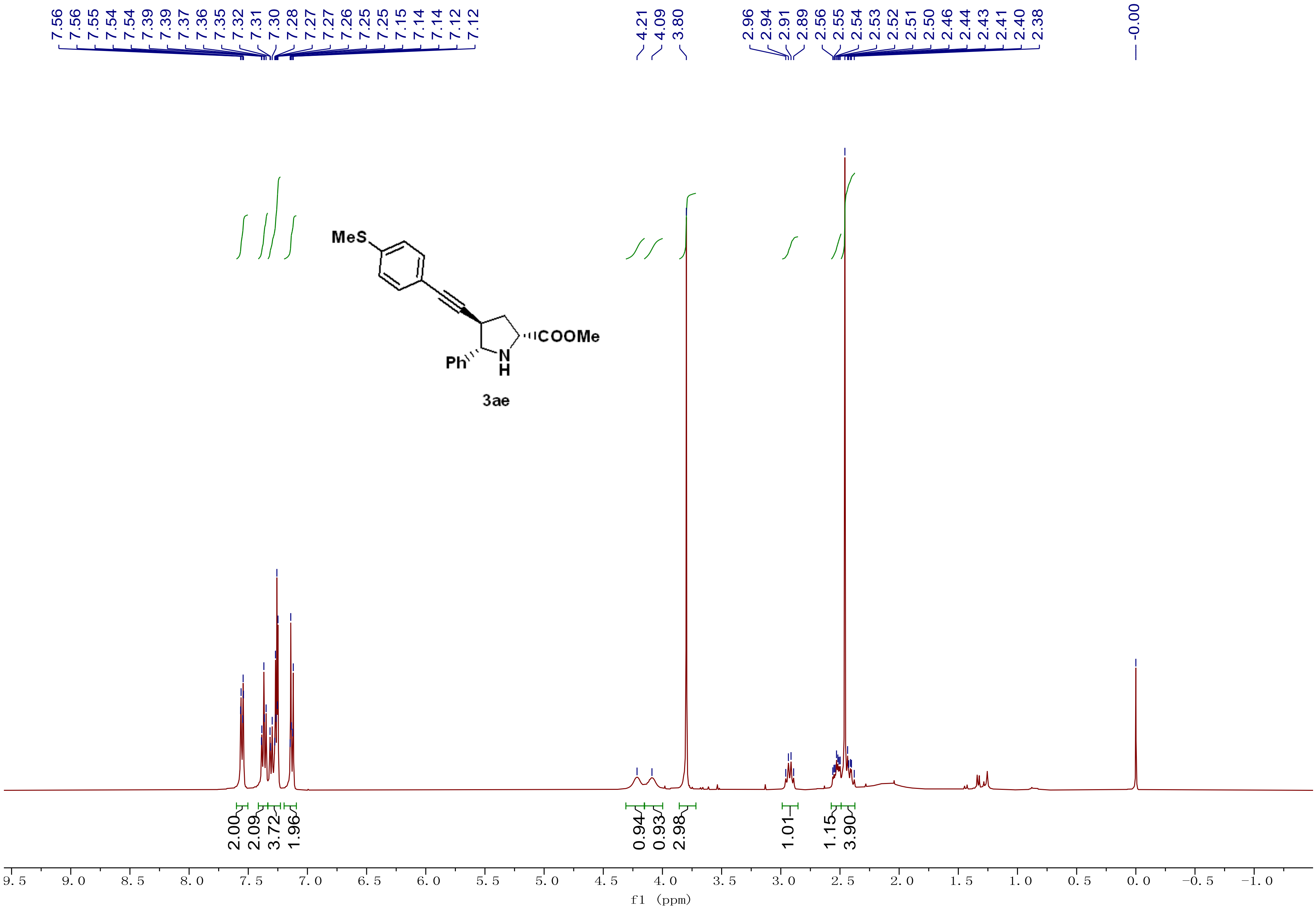
7.56 7.56 7.55 7.54 7.54 7.39 7.39 7.37 7.36 7.35 7.32 7.31 7.30 7.28 7.27 7.27 7.26 7.25 7.25 7.15 7.14 7.14 7.12 7.12
4.21 4.09 3.80
2.96 2.94 2.91 2.89 2.56 2.55 2.54 2.53 2.52 2.51 2.50 2.46 2.44 2.43 2.41 2.40 2.38
-0.00
MeS
COOMe
Ph
N
H
3ae
2.00 2.09 3.72 1.96
0.94 0.93 2.98
1.01
1.15 3.90
9.5 9.0 8.5 8.0 7.5 7.0 6.5 6.0 5.5 5.0 4.5 4.0 3.5 3.0 2.5 2.0 1.5 1.0 0.5 0.0 -0.5 -1.0
f1 (ppm)

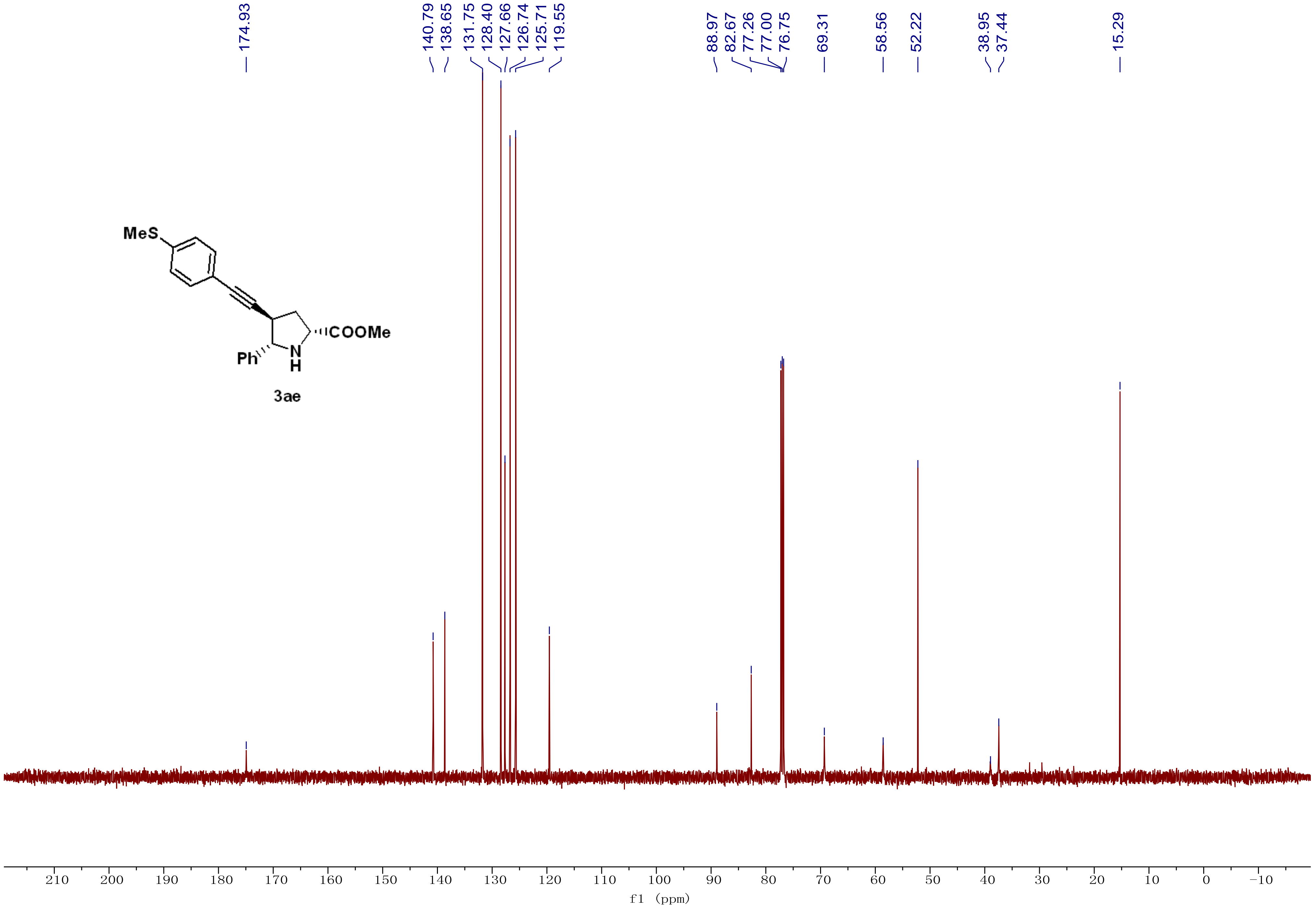
MeS
COOMe
Ph
N
H
3ae
174.93
140.79
138.65
131.75
128.40
127.66
126.74
125.71
119.55
88.97
82.67
77.26
77.00
76.75
69.31
58.56
52.22
38.95
37.44
15.29
210
200
190
180
170
160
150
140
130
120
110
100
90
80
70
60
50
40
30
20
10
0
−10
f1 (ppm)

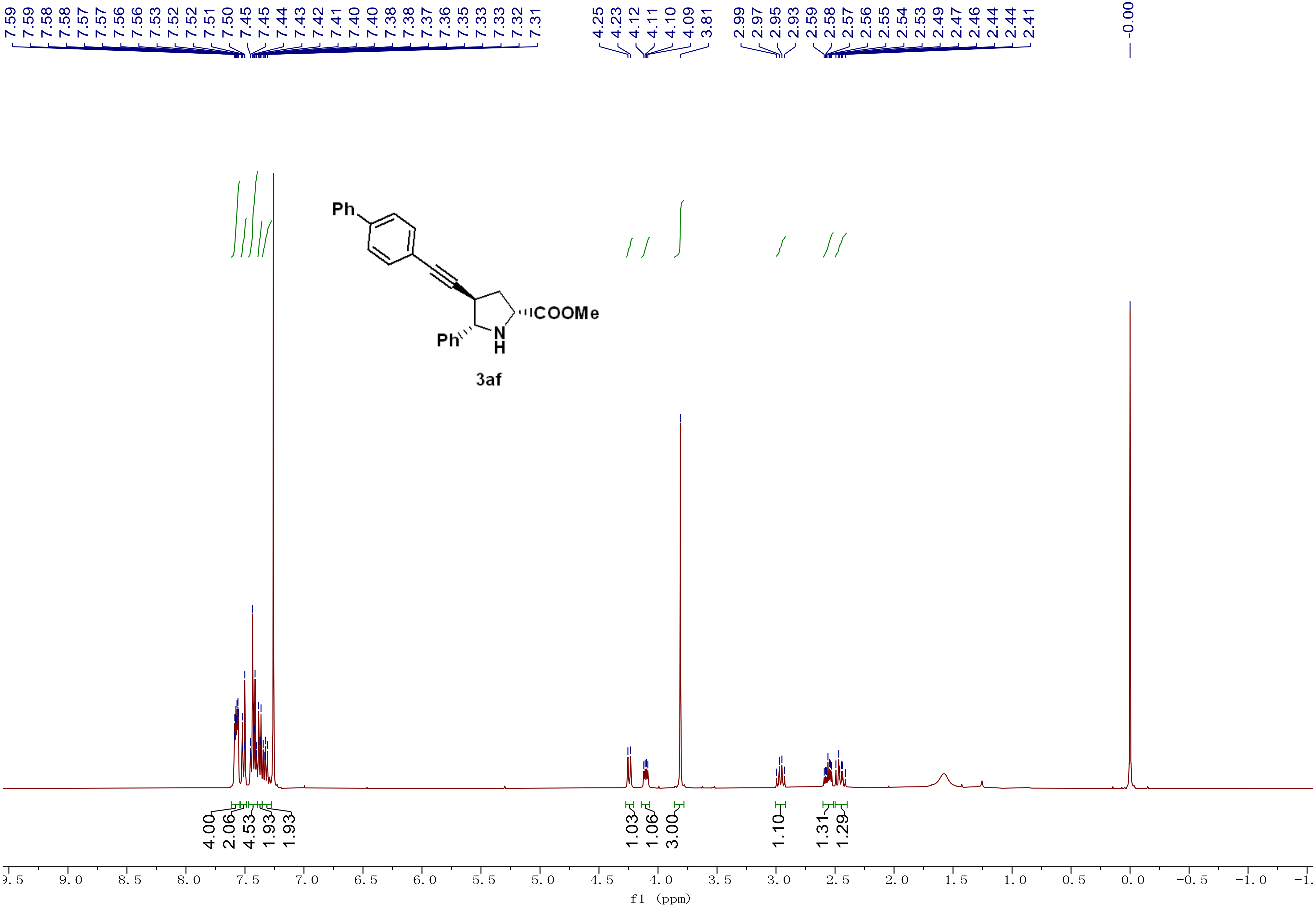
7.59 7.59 7.58 7.58 7.57 7.57 7.56 7.56 7.53 7.52 7.52 7.51 7.50 7.45 7.45 7.44 7.43 7.42 7.41 7.40 7.40 7.38 7.38 7.37 7.36 7.35 7.33 7.33 7.32 7.31
4.25 4.23 4.12 4.11 4.10 4.09 3.81
2.99 2.97 2.95 2.93 2.59 2.58 2.57 2.56 2.55 2.54 2.53 2.49 2.47 2.46 2.44 2.44 2.41
-0.00
Ph
COOMe
Ph
N
H
3af
4.00 2.06 4.53 1.93 1.93
1.03 1.06 3.00
1.10
1.31 1.29
9.5 9.0 8.5 8.0 7.5 7.0 6.5 6.0 5.5 5.0 4.5 4.0 3.5 3.0 2.5 2.0 1.5 1.0 0.5 0.0 -0.5 -1.0 -1.
f1 (ppm)

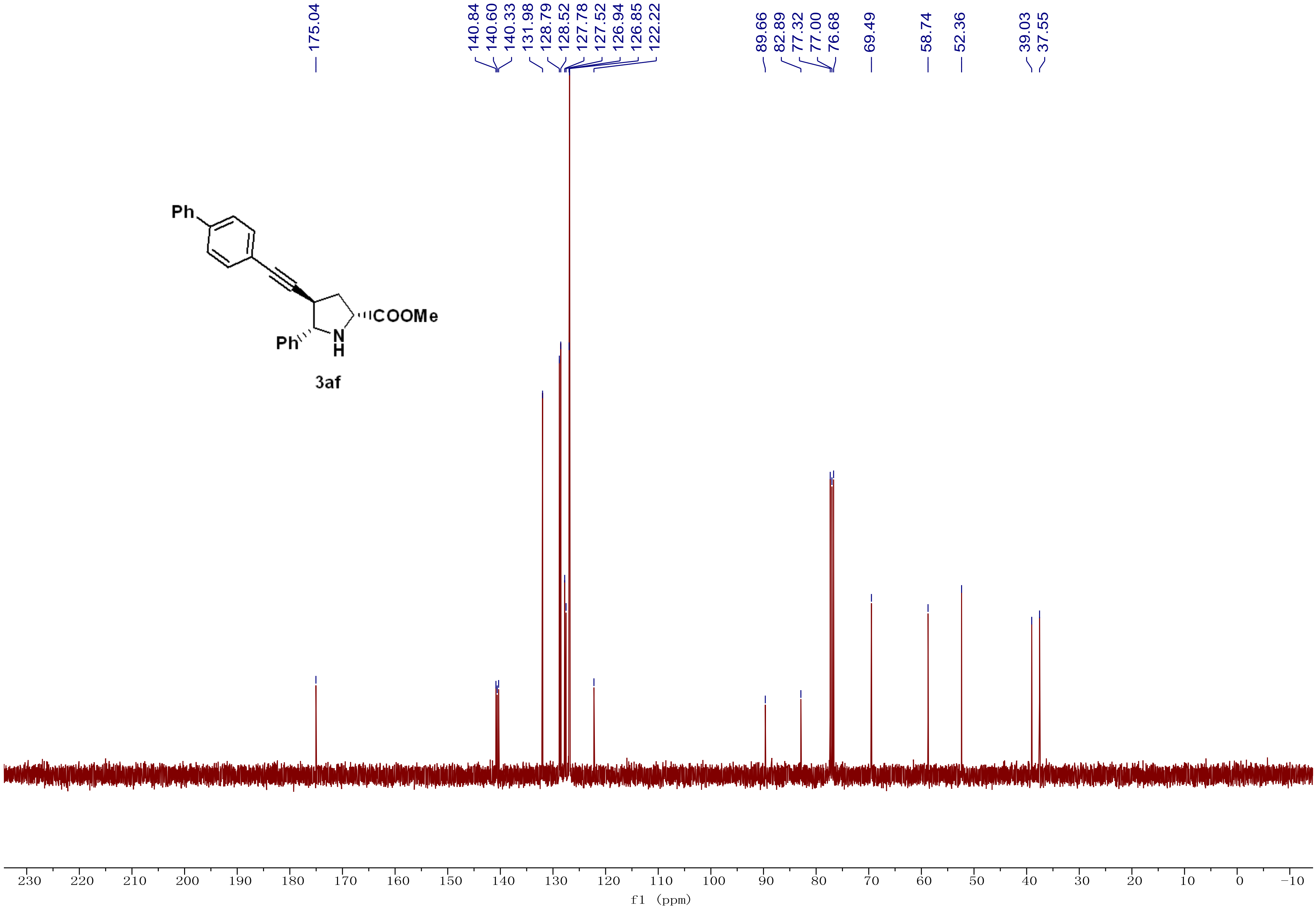
175.04
140.84
140.60
140.33
131.98
128.79
128.52
127.78
127.52
126.94
126.85
122.22
89.66
82.89
77.32
77.00
76.68
69.49
58.74
52.36
39.03
37.55
Ph
Ph
COOMe
N
H
3af
230 220 210 200 190 180 170 160 150 140 130 120 110 100 90 80 70 60 50 40 30 20 10 0 −10
f1 (ppm)

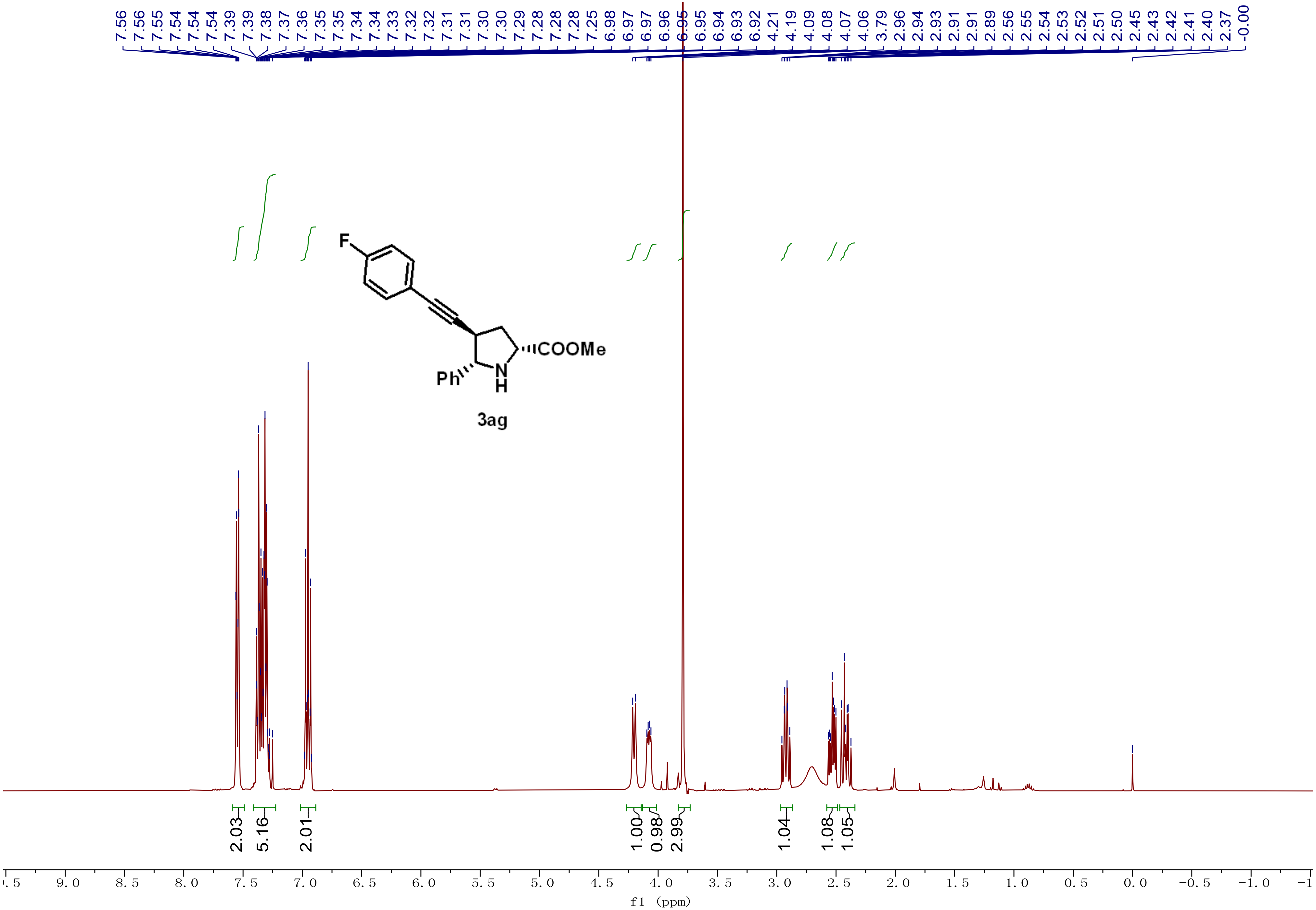
7.56 7.56 7.55 7.54 7.54 7.54 7.39 7.39 7.38 7.37 7.36 7.35 7.35 7.34 7.34 7.33 7.32 7.32 7.31 7.31 7.30 7.30 7.29 7.28 7.28 7.28 7.25 6.98 6.97 6.97 6.96 6.95 6.95 6.94 6.93 6.92 4.21 4.19 4.09 4.08 4.07 4.06 3.79 2.96 2.94 2.93 2.91 2.91 2.89 2.56 2.55 2.54 2.53 2.52 2.51 2.50 2.45 2.43 2.42 2.41 2.40 2.37 -0.00
F
COOMe
Ph
N
H
3ag
2.03
5.16
2.01
1.00
0.98
2.99
1.04
1.08
1.05
f1 (ppm)

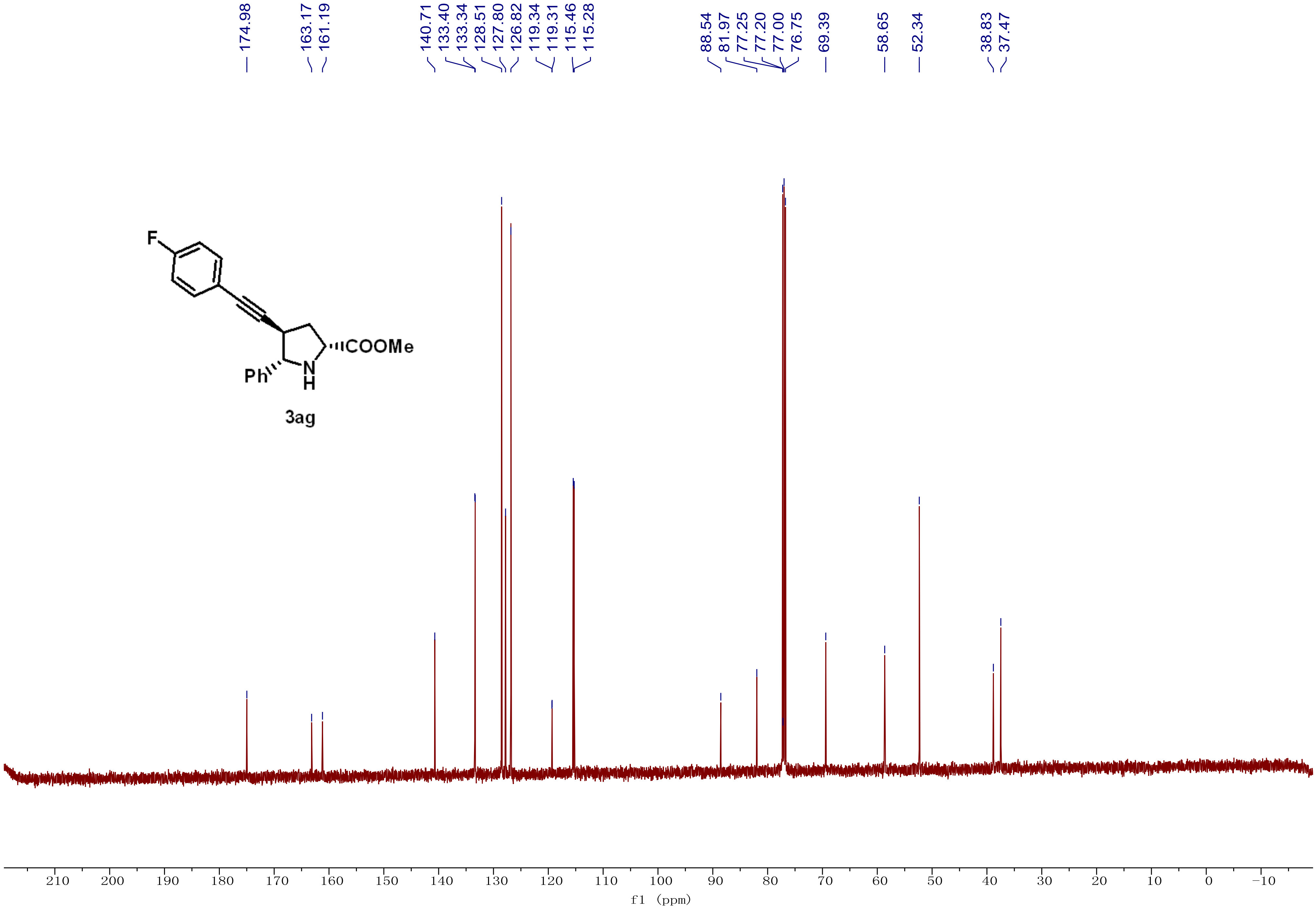

174.98
163.17
161.19
140.71
133.40
133.34
128.51
127.80
126.82
119.34
119.31
115.46
115.28
88.54
81.97
77.25
77.20
77.00
76.75
69.39
58.65
52.34
38.83
37.47
F
COOMe
Ph
N
H
3ag
210 200 190 180 170 160 150 140 130 120 110 100 90 80 70 60 50 40 30 20 10 0 -10
f1 (ppm)

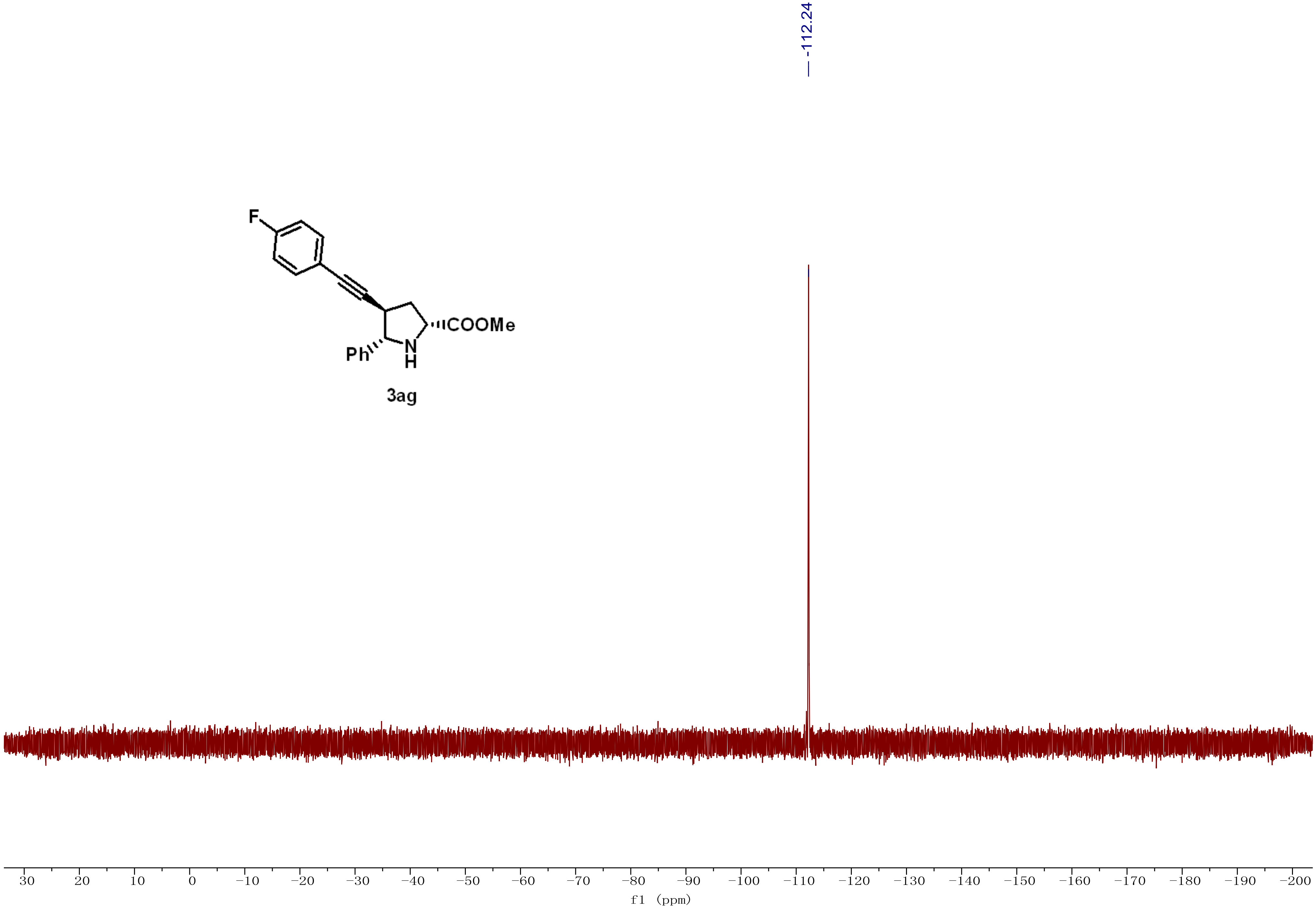
-112.24
F
COOMe
Ph
N
H
3ag
30 20 10 0 −10 −20 −30 −40 −50 −60 −70 −80 −90 −100 −110 −120 −130 −140 −150 −160 −170 −180 −190 −200
f1 (ppm)

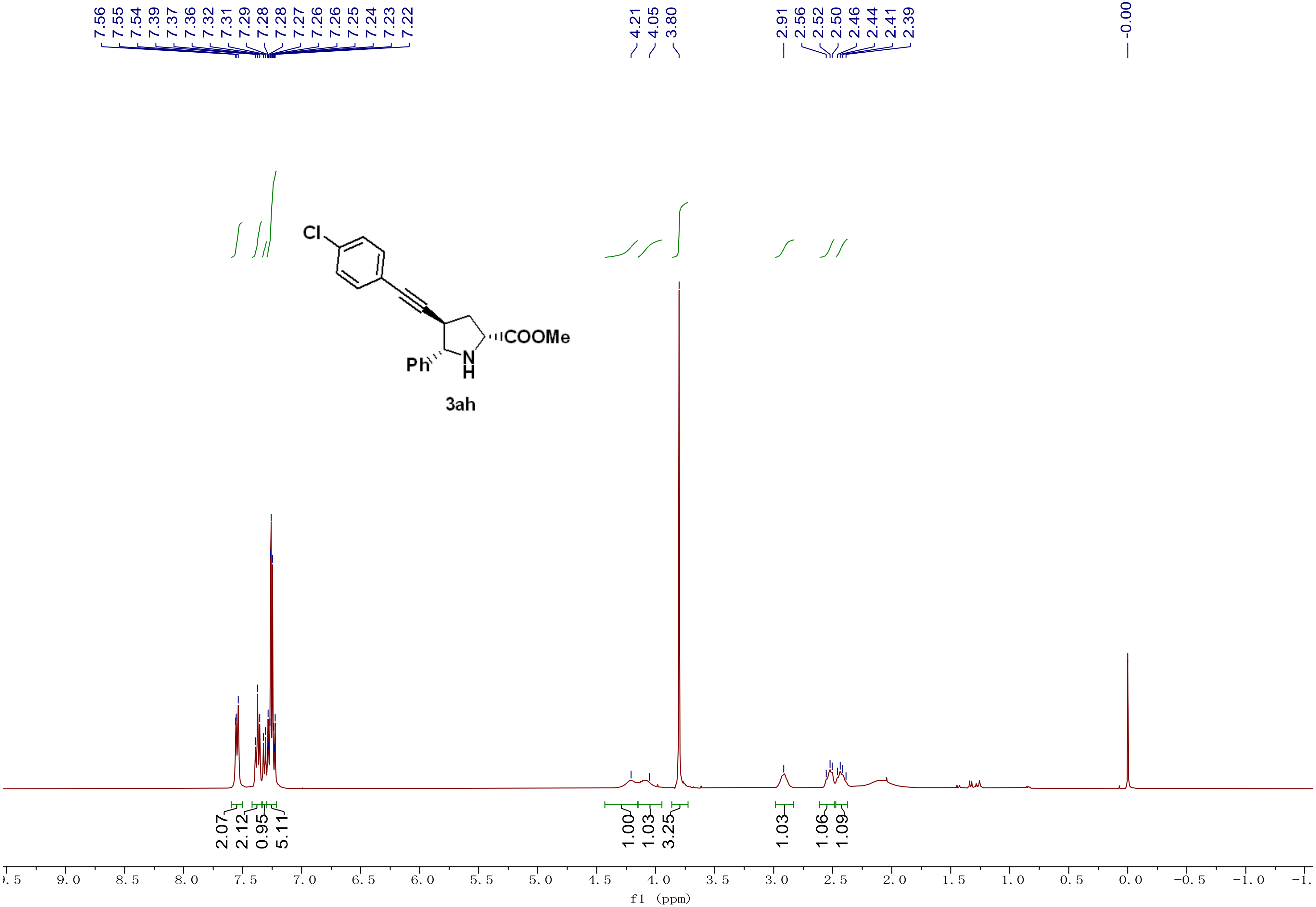

7.56
7.55
7.54
7.39
7.37
7.36
7.32
7.31
7.29
7.28
7.28
7.27
7.26
7.26
7.25
7.24
7.23
7.22
4.21
4.05
3.80
2.91
2.56
2.52
2.50
2.46
2.44
2.41
2.39
-0.00
Cl
COOMe
Ph
N
H
3ah
2.07
2.12
0.95
5.11
1.00
1.03
3.25
1.03
1.06
1.09
f1 (ppm)

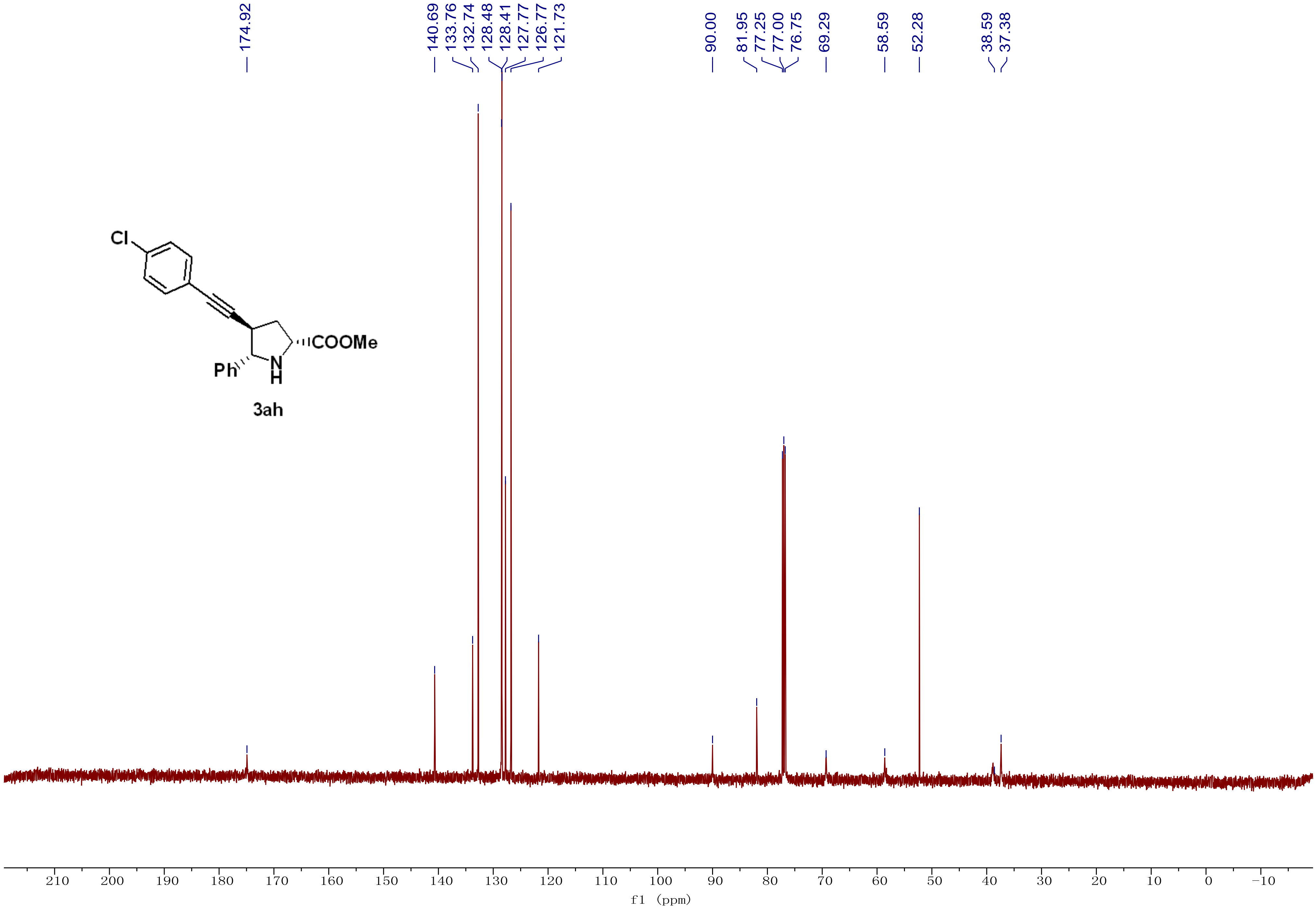
174.92
140.69
133.76
132.74
128.48
128.41
127.77
126.77
121.73
90.00
81.95
77.25
77.00
76.75
69.29
58.59
52.28
38.59
37.38
Cl
COOMe
Ph
N
H
3ah
210 200 190 180 170 160 150 140 130 120 110 100 90 80 70 60 50 40 30 20 10 0 −10
f1 (ppm)

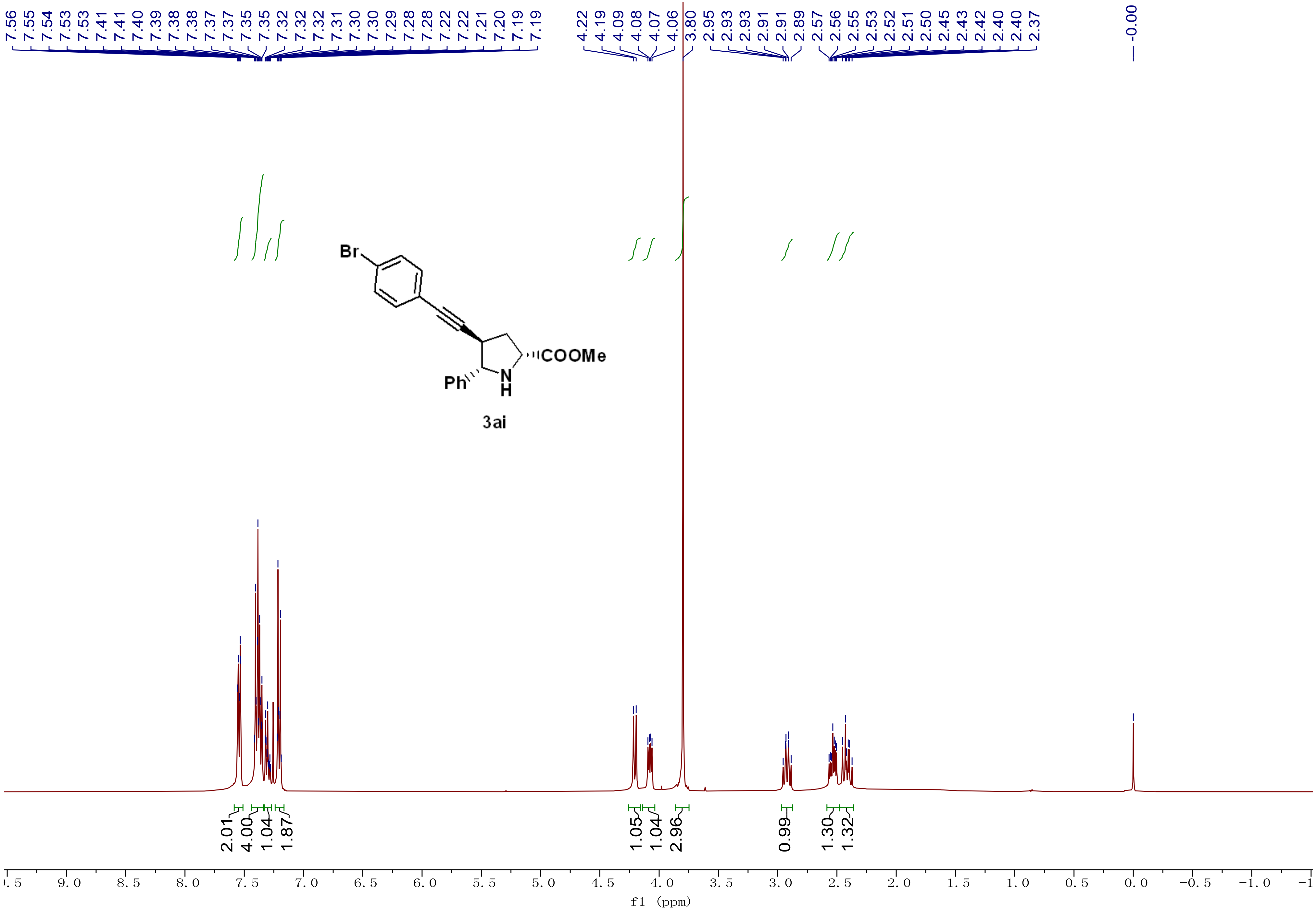
7.56 7.55 7.54 7.53 7.53 7.41 7.41 7.40 7.39 7.38 7.38 7.37 7.37 7.35 7.35 7.32 7.32 7.32 7.31 7.30 7.30 7.29 7.28 7.28 7.22 7.22 7.21 7.20 7.19 7.19
4.22 4.19 4.09 4.08 4.07 4.06 3.80 2.95 2.93 2.93 2.91 2.91 2.89 2.57 2.56 2.55 2.53 2.52 2.51 2.50 2.45 2.43 2.42 2.40 2.40 2.37
-0.00
Br
Ph
N
H
COOMe
3ai
2.01 4.00 1.04 1.87
1.05 1.04 2.96
0.99
1.30 1.32
f1 (ppm)

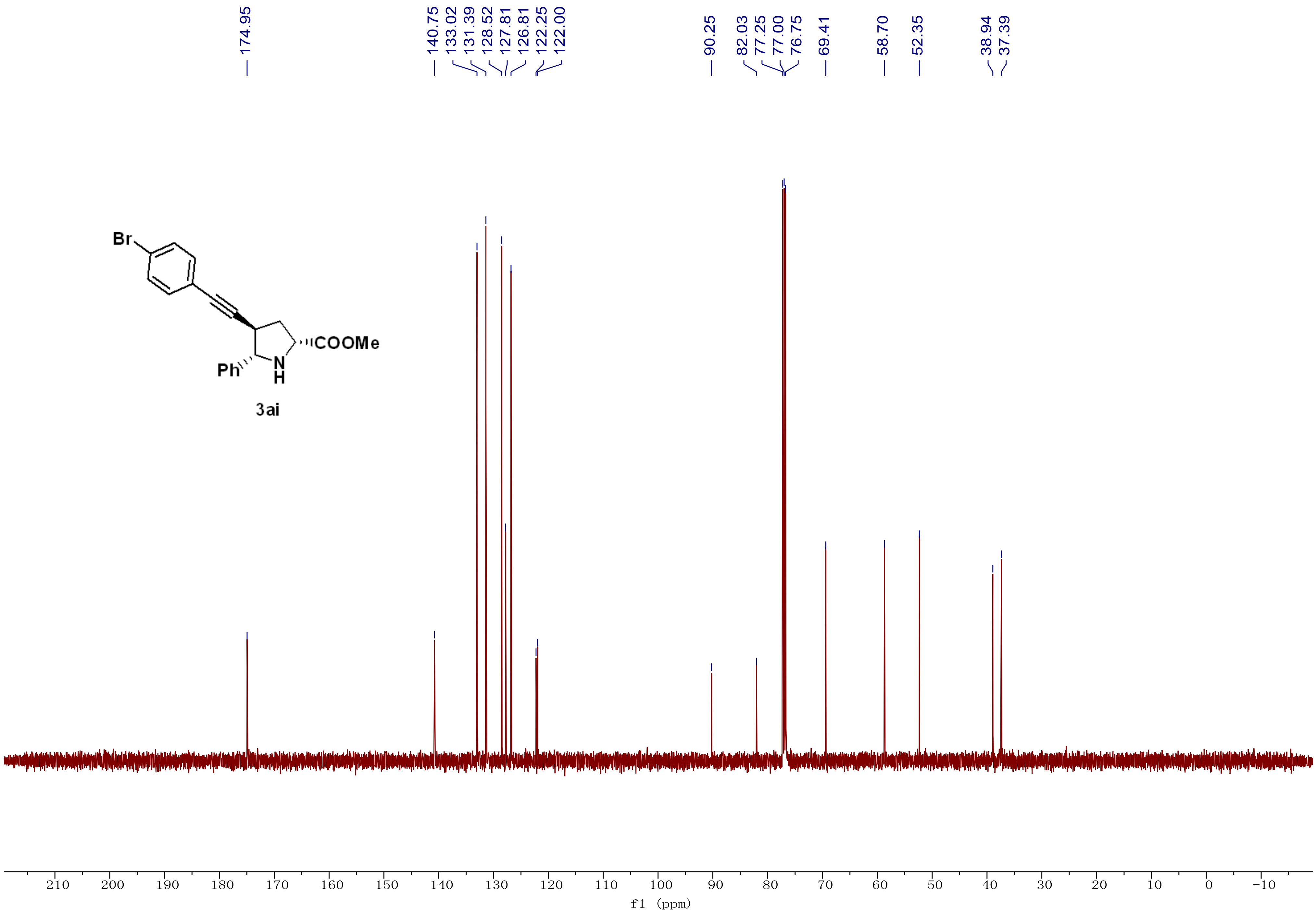

174.95
140.75
133.02
131.39
128.52
127.81
126.81
122.25
122.00
90.25
82.03
77.25
77.00
76.75
69.41
58.70
52.35
38.94
37.39
Br
COOMe
Ph
N
H
3ai
210
200
190
180
170
160
150
140
130
120
110
100
90
80
70
60
50
40
30
20
10
0
-10
f1 (ppm)

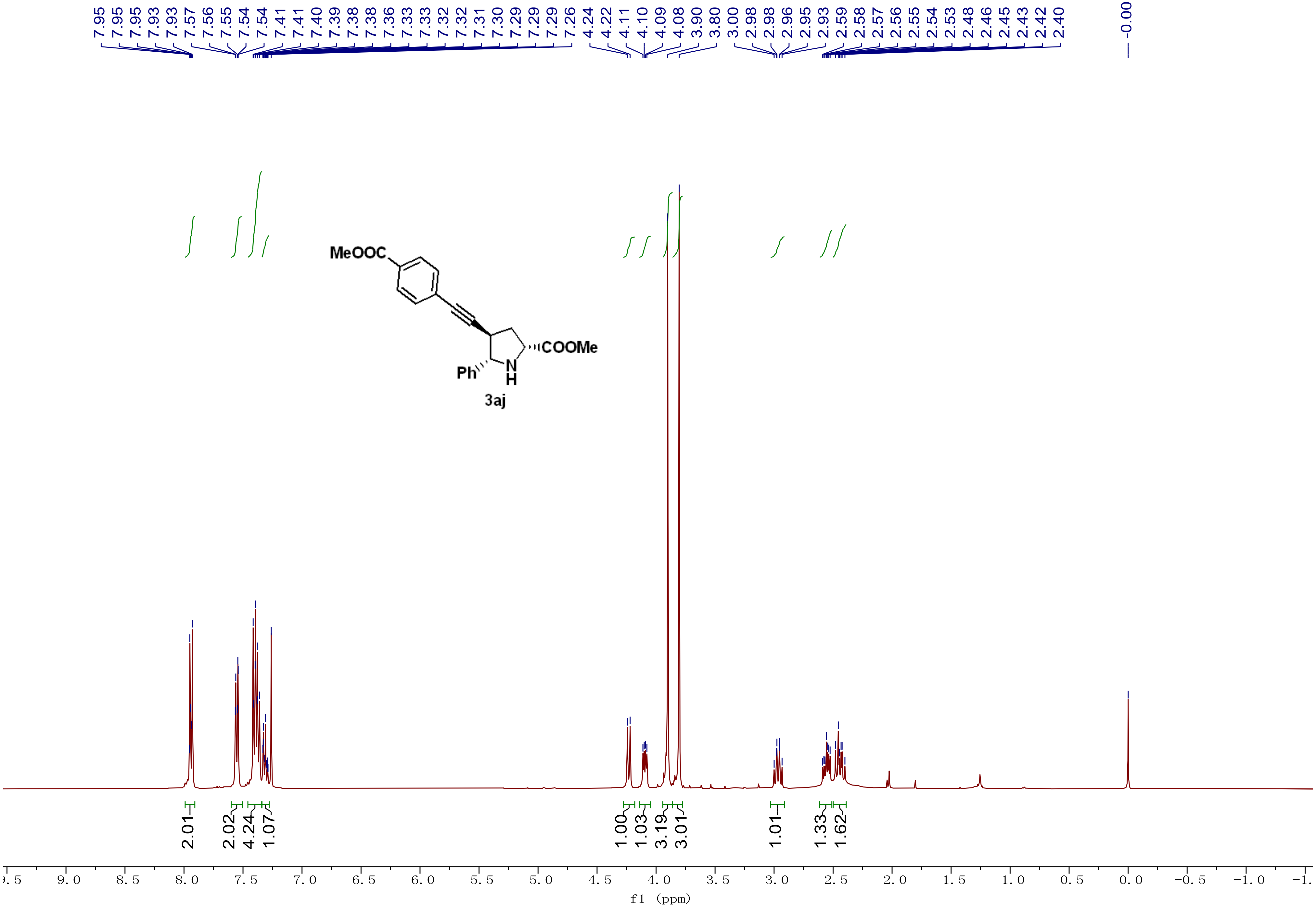
7.95 7.95 7.95 7.93 7.93 7.57 7.56 7.55 7.54 7.54 7.41 7.41 7.40 7.39 7.38 7.38 7.36 7.33 7.33 7.32 7.32 7.31 7.30 7.29 7.29 7.29 7.26 4.24 4.22 4.11 4.10 4.09 4.08 3.90 3.80 3.00 2.98 2.98 2.96 2.95 2.93 2.59 2.58 2.57 2.56 2.55 2.54 2.53 2.48 2.46 2.45 2.43 2.42 2.40
-0.00
MeOOC
COOMe
Ph
N
H
3aj
2.01 2.02 4.24 1.07 1.00 1.03 3.19 3.01 1.01 1.33 1.62
f1 (ppm)

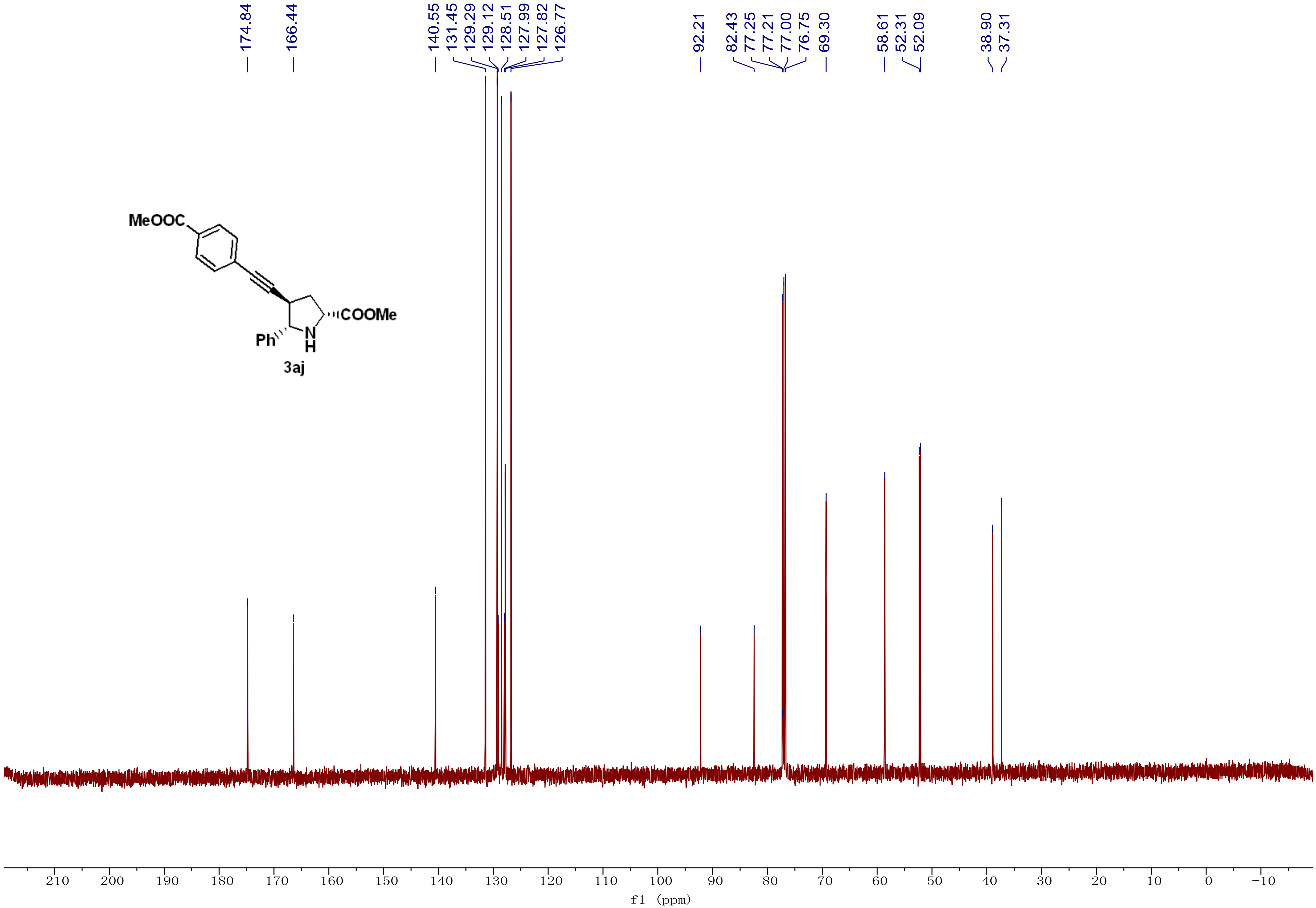
174.84
166.44
140.55
131.45
129.29
129.12
128.51
127.99
127.82
126.77
92.21
82.43
77.25
77.21
77.00
76.75
69.30
58.61
52.31
52.09
38.90
37.31
MeOOC
COOMe
Ph
N
H
3aj
210 200 190 180 170 160 150 140 130 120 110 100 90 80 70 60 50 40 30 20 10 0 −10
f1 (ppm)

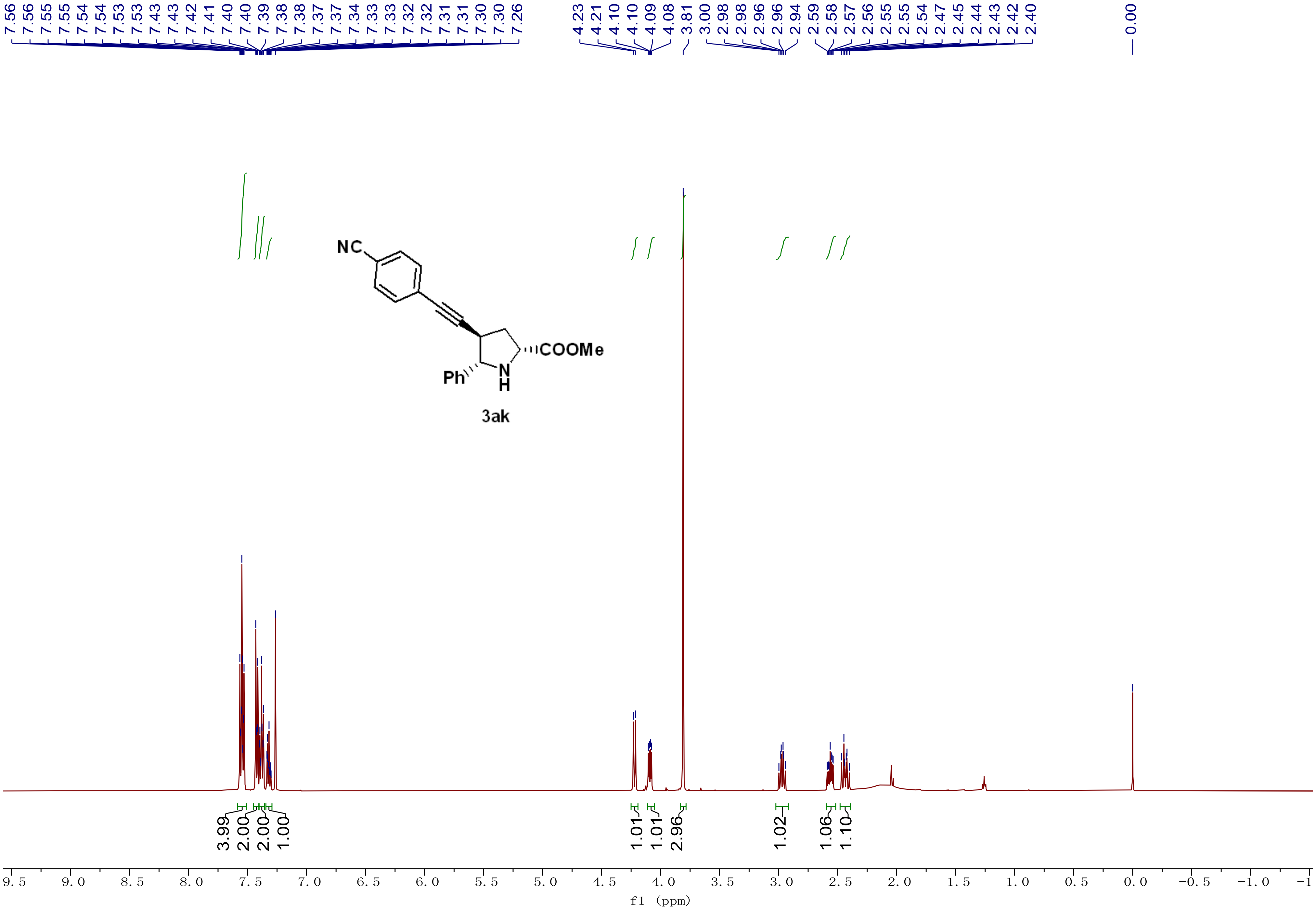
7.56 7.56 7.55 7.55 7.54 7.54 7.53 7.53 7.43 7.43 7.42 7.41 7.40 7.40 7.39 7.38 7.38 7.37 7.37 7.34 7.33 7.33 7.32 7.32 7.31 7.31 7.30 7.30 7.26
4.23 4.21 4.10 4.10 4.09 4.08 3.81 3.00 2.98 2.98 2.96 2.96 2.94 2.59 2.58 2.57 2.56 2.55 2.55 2.54 2.47 2.45 2.44 2.43 2.42 2.40
0.00
NC
COOMe
Ph
N
H
3ak
3.99 2.00 2.00 1.00
1.01 1.01 2.96
1.02
1.06 1.10
9.5 9.0 8.5 8.0 7.5 7.0 6.5 6.0 5.5 5.0 4.5 4.0 3.5 3.0 2.5 2.0 1.5 1.0 0.5 0.0 −0.5 −1.0 −1
f1 (ppm)

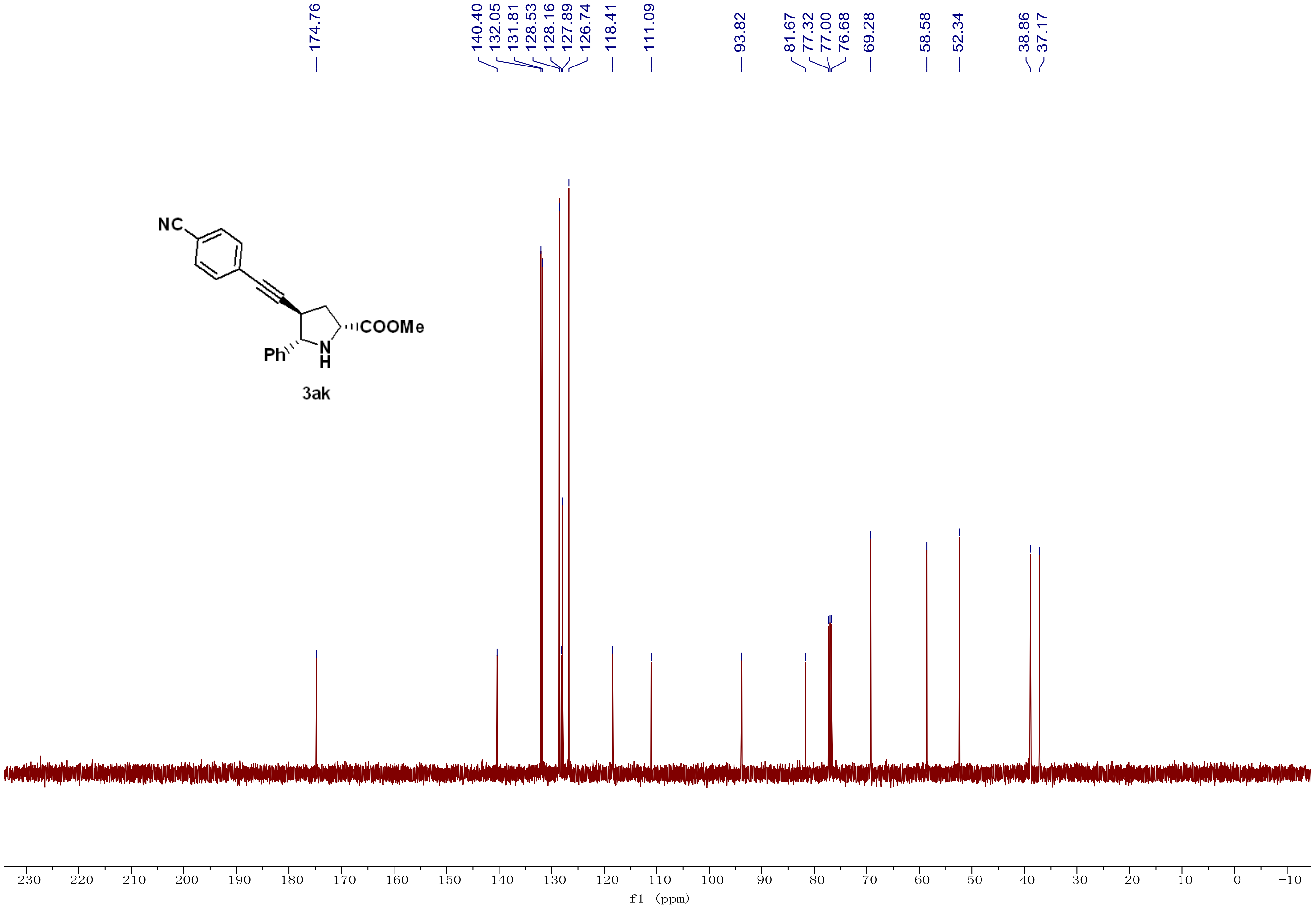
174.76
140.40
132.05
131.81
128.53
128.16
127.89
126.74
118.41
111.09
93.82
81.67
77.32
77.00
76.68
69.28
58.58
52.34
38.86
37.17
NC
COOMe
Ph
N
H
3ak
230 220 210 200 190 180 170 160 150 140 130 120 110 100 90 80 70 60 50 40 30 20 10 0 −10
f1 (ppm)

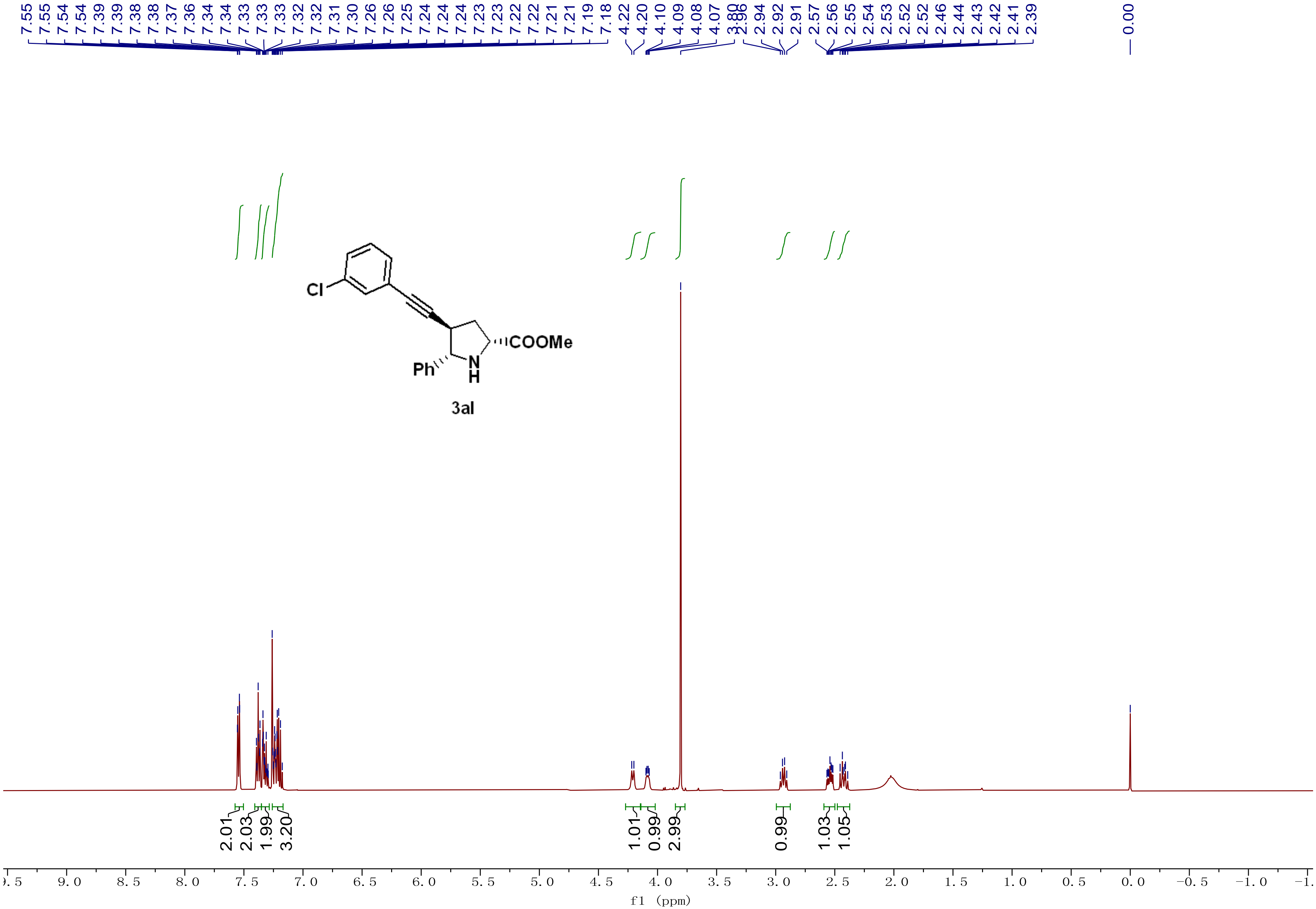
7.55 7.55 7.54 7.54 7.39 7.39 7.38 7.38 7.37 7.36 7.34 7.34 7.33 7.33 7.33 7.32 7.32 7.31 7.30 7.26 7.26 7.25 7.24 7.24 7.24 7.23 7.23 7.22 7.22 7.21 7.21 7.19 7.18 4.22 4.20 4.10 4.09 4.08 4.07 3.80 2.96 2.94 2.92 2.91 2.57 2.56 2.55 2.54 2.53 2.52 2.52 2.46 2.44 2.43 2.42 2.41 2.39
0.00
Cl
COOMe
Ph
N
H
3al
2.01 2.03 1.99 3.20 1.01 0.99 2.99 0.99 1.03 1.05
f1 (ppm)

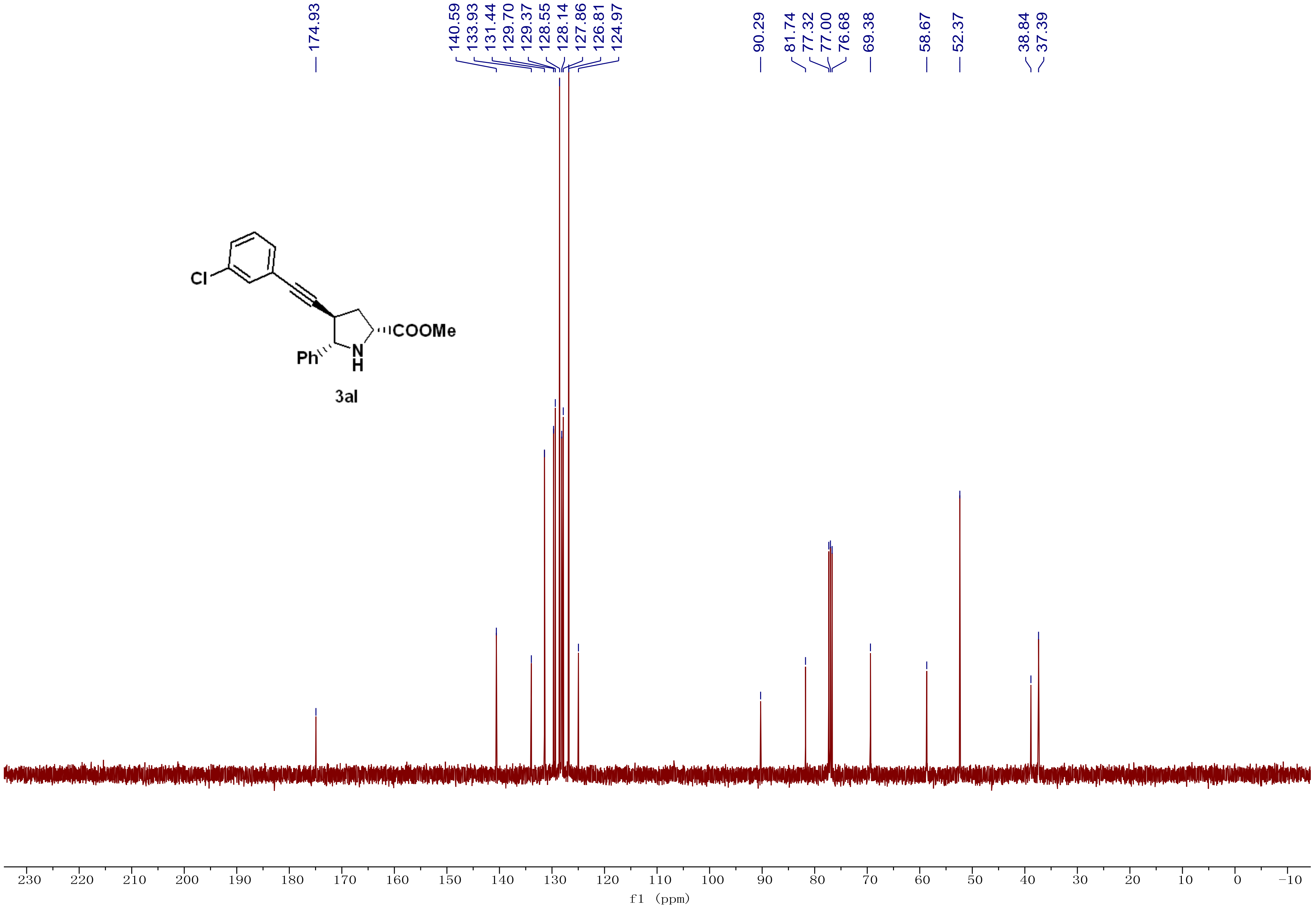
174.93
140.59
133.93
131.44
129.70
129.37
128.55
128.14
127.86
126.81
124.97
90.29
81.74
77.32
77.00
76.68
69.38
58.67
52.37
38.84
37.39
Cl
COOMe
Ph
N
H
3al
230 220 210 200 190 180 170 160 150 140 130 120 110 100 90 80 70 60 50 40 30 20 10 0 −10
f1 (ppm)

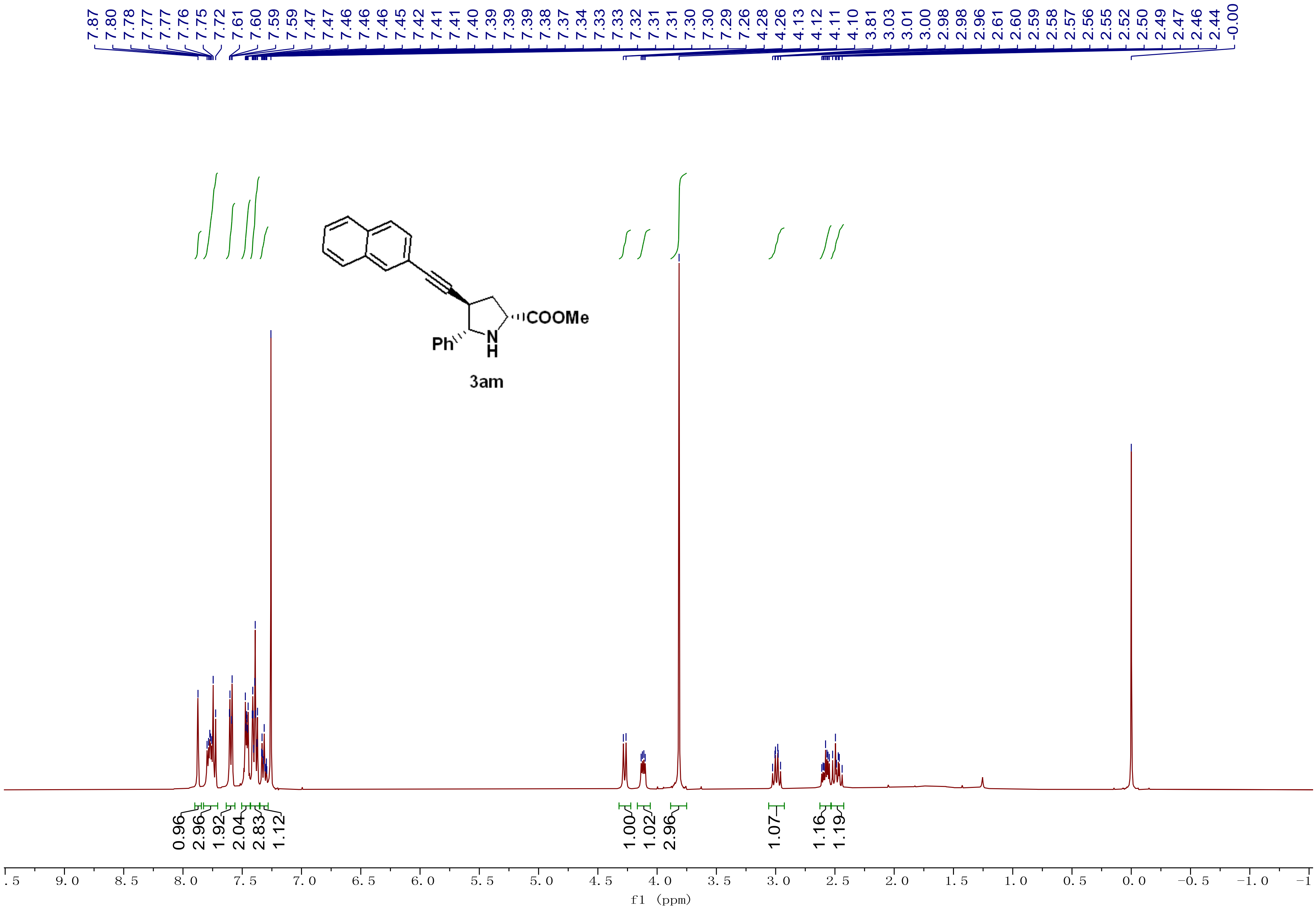
7.87 7.80 7.78 7.77 7.77 7.76 7.75 7.72 7.61 7.60 7.59 7.59 7.47 7.47 7.46 7.46 7.46 7.45 7.42 7.41 7.41 7.40 7.39 7.39 7.39 7.38 7.37 7.34 7.33 7.33 7.32 7.31 7.31 7.30 7.30 7.29 7.26 4.28 4.26 4.13 4.12 4.11 4.10 3.81 3.03 3.01 3.00 2.98 2.98 2.96 2.61 2.60 2.59 2.58 2.57 2.56 2.55 2.52 2.50 2.49 2.47 2.46 2.44 -0.00
COOMe
Ph
N
H
3am
0.96 2.96 1.92 2.04 2.83 1.12 1.00 1.02 2.96 1.07 1.16 1.19
9.0 8.5 8.0 7.5 7.0 6.5 6.0 5.5 5.0 4.5 4.0 3.5 3.0 2.5 2.0 1.5 1.0 0.5 0.0 −0.5 −1.0
f1 (ppm)

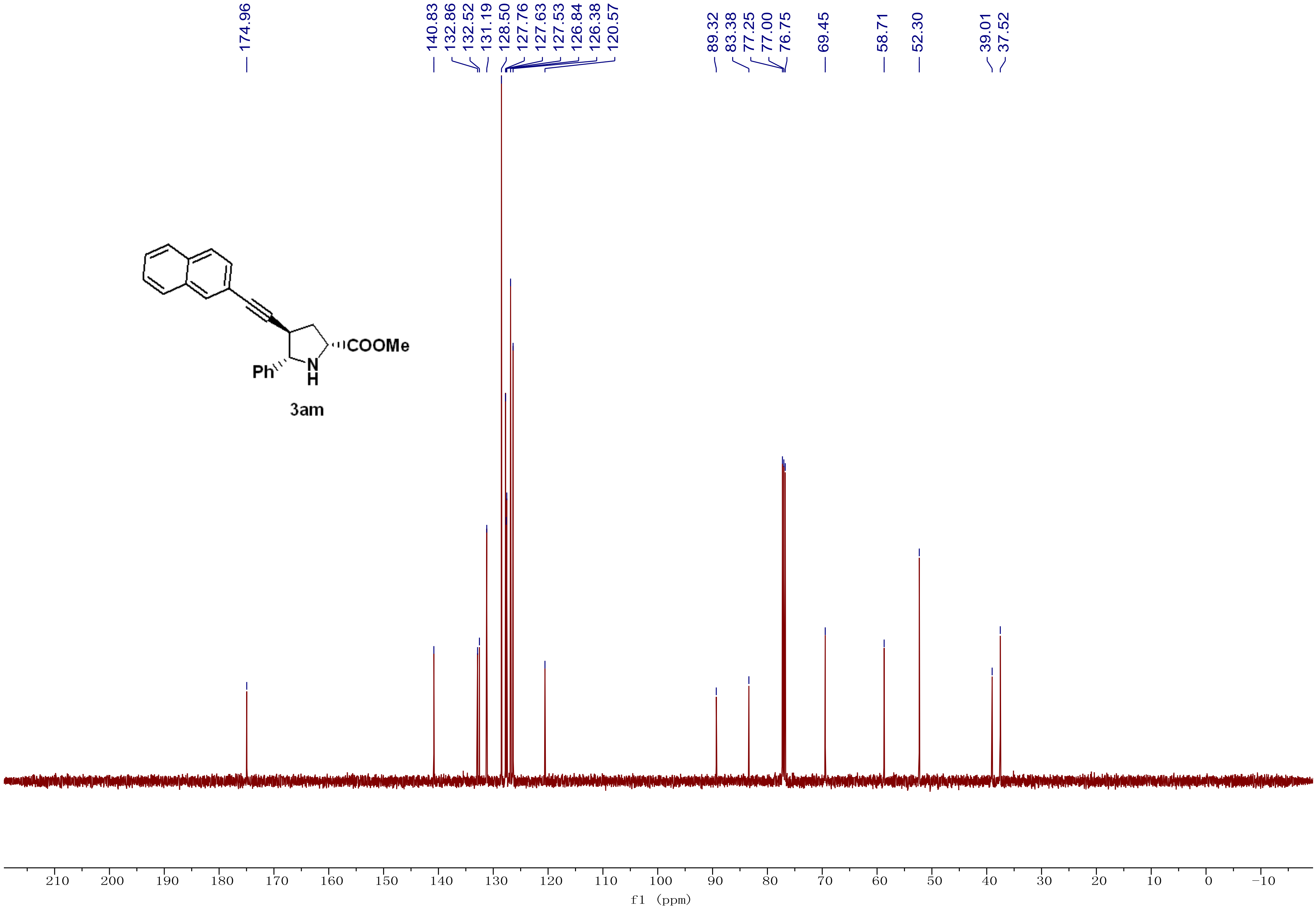

174.96
140.83
132.86
132.52
131.19
128.50
127.76
127.63
127.53
126.84
126.38
120.57
89.32
83.38
77.25
77.00
76.75
69.45
58.71
52.30
39.01
37.52
COOMe
Ph
N
H
210 200 190 180 170 160 150 140 130 120 110 100 90 80 70 60 50 40 30 20 10 0 −10
f1 (ppm)

3am

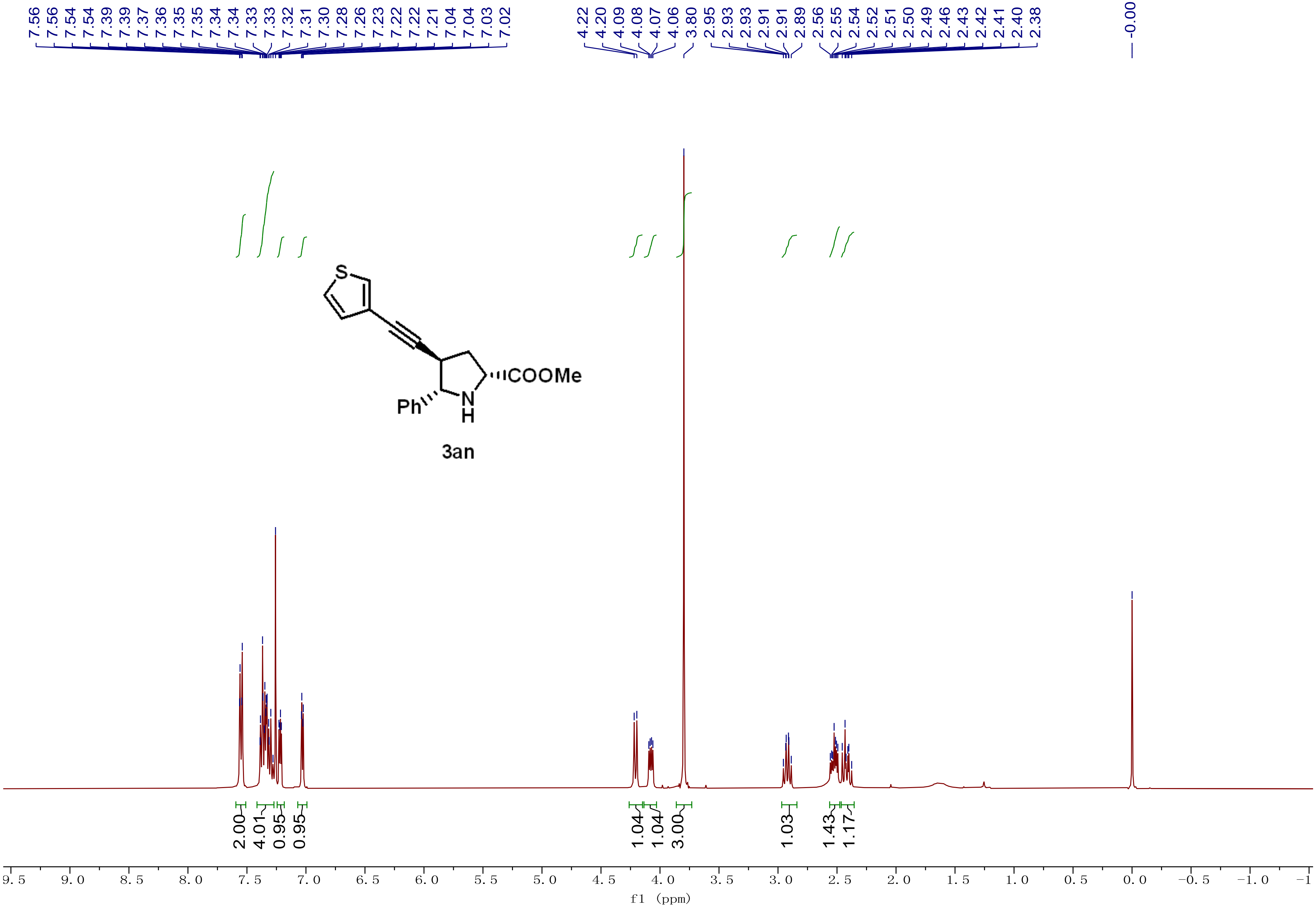
7.56
7.56
7.54
7.54
7.39
7.39
7.37
7.36
7.35
7.35
7.34
7.34
7.33
7.33
7.32
7.31
7.30
7.28
7.26
7.23
7.22
7.22
7.21
7.04
7.04
7.03
7.02
4.22
4.20
4.09
4.08
4.07
4.06
3.80
2.95
2.93
2.93
2.91
2.91
2.89
2.56
2.55
2.54
2.52
2.51
2.50
2.49
2.46
2.43
2.42
2.41
2.40
2.38
-0.00
S
COOMe
Ph
N
H
3an
2.00
4.01
0.95
0.95
1.04
1.04
3.00
1.03
1.43
1.17
9.5
9.0
8.5
8.0
7.5
7.0
6.5
6.0
5.5
5.0
4.5
4.0
3.5
3.0
2.5
2.0
1.5
1.0
0.5
0.0
−0.5
−1.0
f1 (ppm)

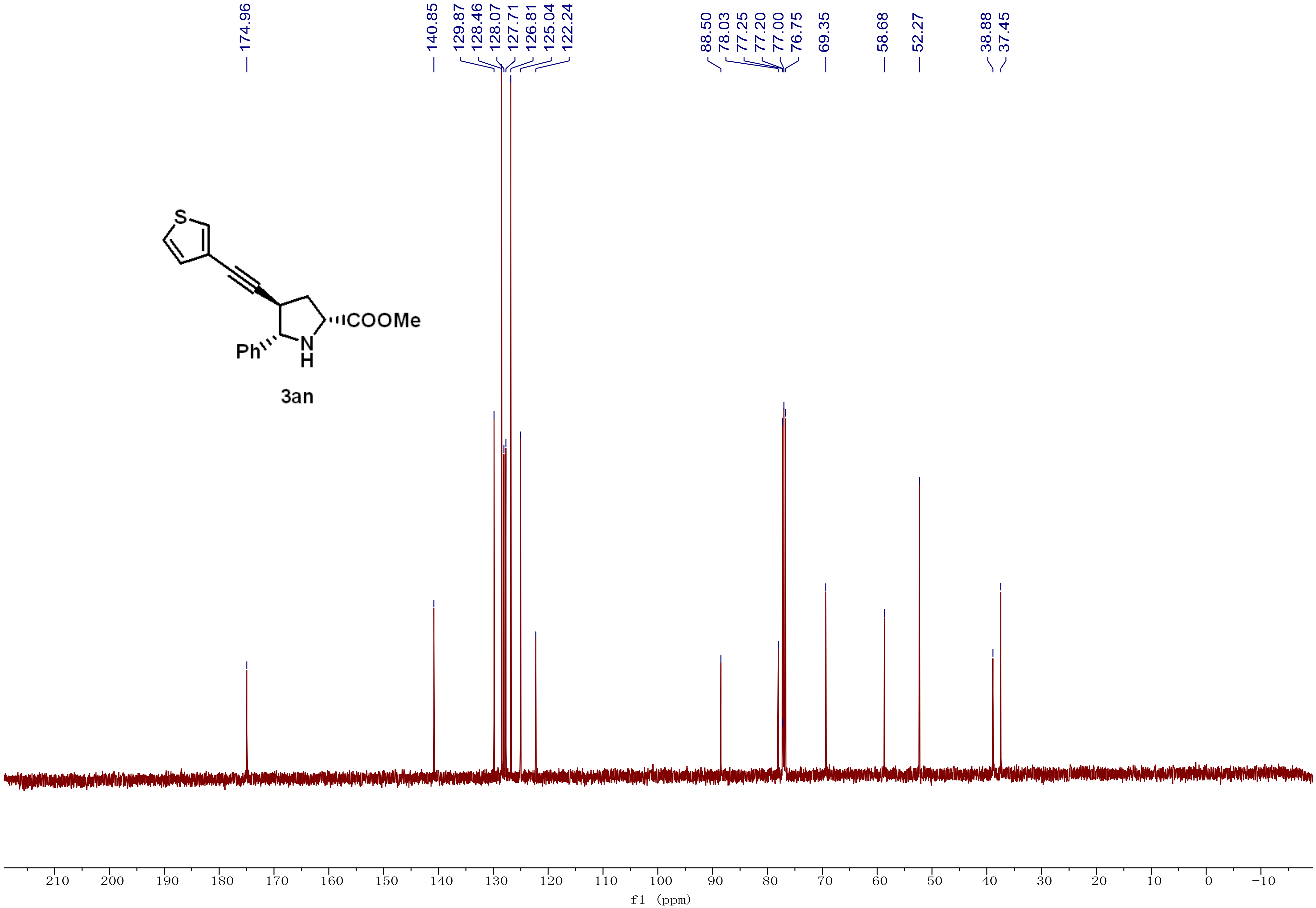
174.96
140.85
129.87
128.46
128.07
127.71
126.81
125.04
122.24
88.50
78.03
77.25
77.20
77.00
76.75
69.35
58.68
52.27
38.88
37.45
S
COOMe
Ph
N
H
3an
210 200 190 180 170 160 150 140 130 120 110 100 90 80 70 60 50 40 30 20 10 0 −10
f1 (ppm)

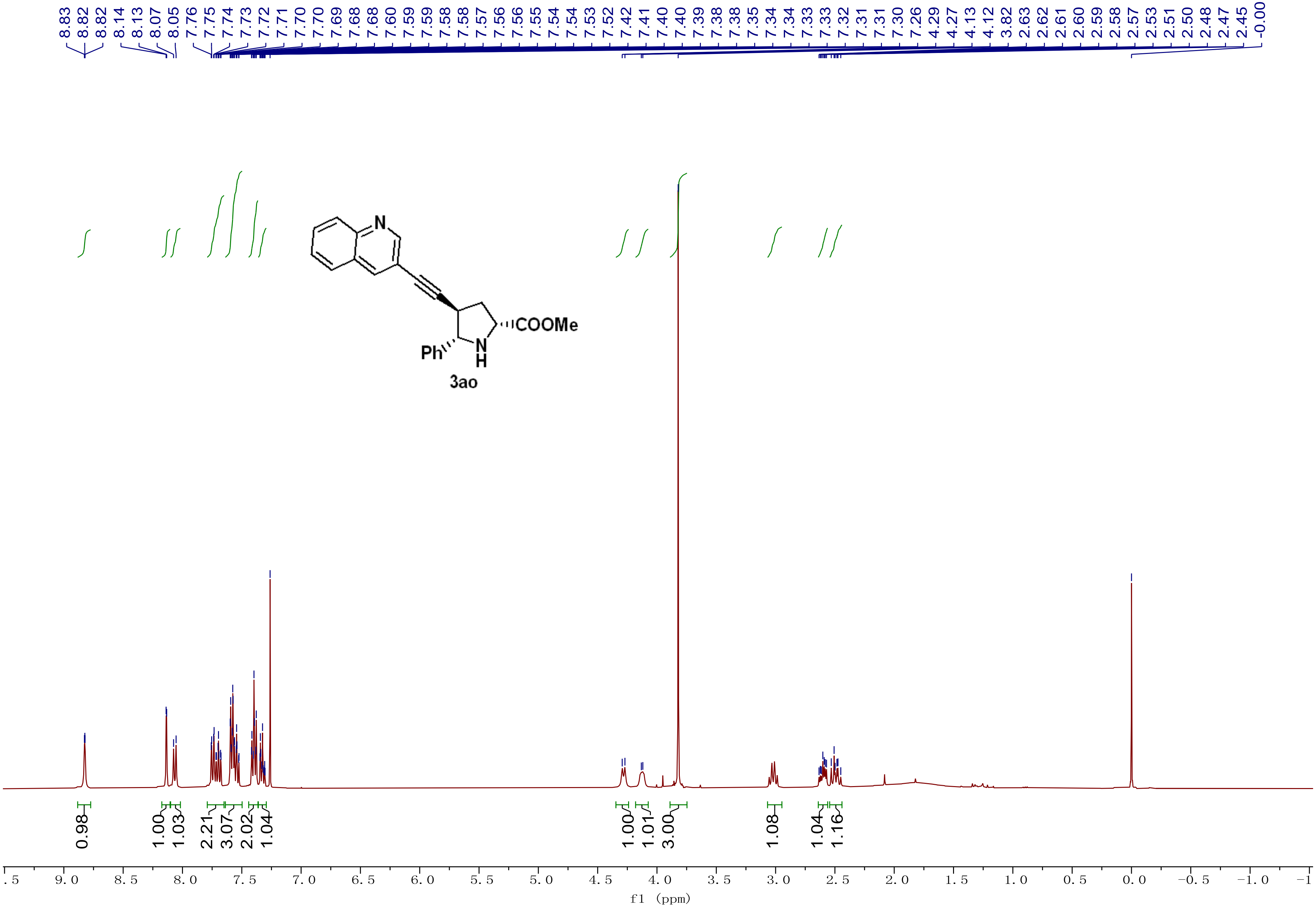
8.83 8.82 8.82 8.14 8.13 8.07 8.05 7.76 7.75 7.74 7.73 7.72 7.71 7.70 7.70 7.69 7.68 7.68 7.60 7.59 7.59 7.58 7.58 7.57 7.56 7.56 7.55 7.54 7.54 7.53 7.52 7.42 7.41 7.40 7.40 7.39 7.38 7.38 7.35 7.34 7.34 7.33 7.33 7.32 7.31 7.31 7.30 7.26 4.29 4.27 4.13 4.12 3.82 2.63 2.62 2.61 2.60 2.59 2.58 2.57 2.53 2.51 2.50 2.48 2.47 2.45 -0.00
N
COOMe
Ph
N
H
3ao
0.98
1.00
1.03
2.21
3.07
2.02
1.04
1.00
1.01
3.00
1.08
1.04
1.16
.5 9.0 8.5 8.0 7.5 7.0 6.5 6.0 5.5 5.0 4.5 4.0 3.5 3.0 2.5 2.0 1.5 1.0 0.5 0.0 −0.5 −1.0 −1
f1 (ppm)

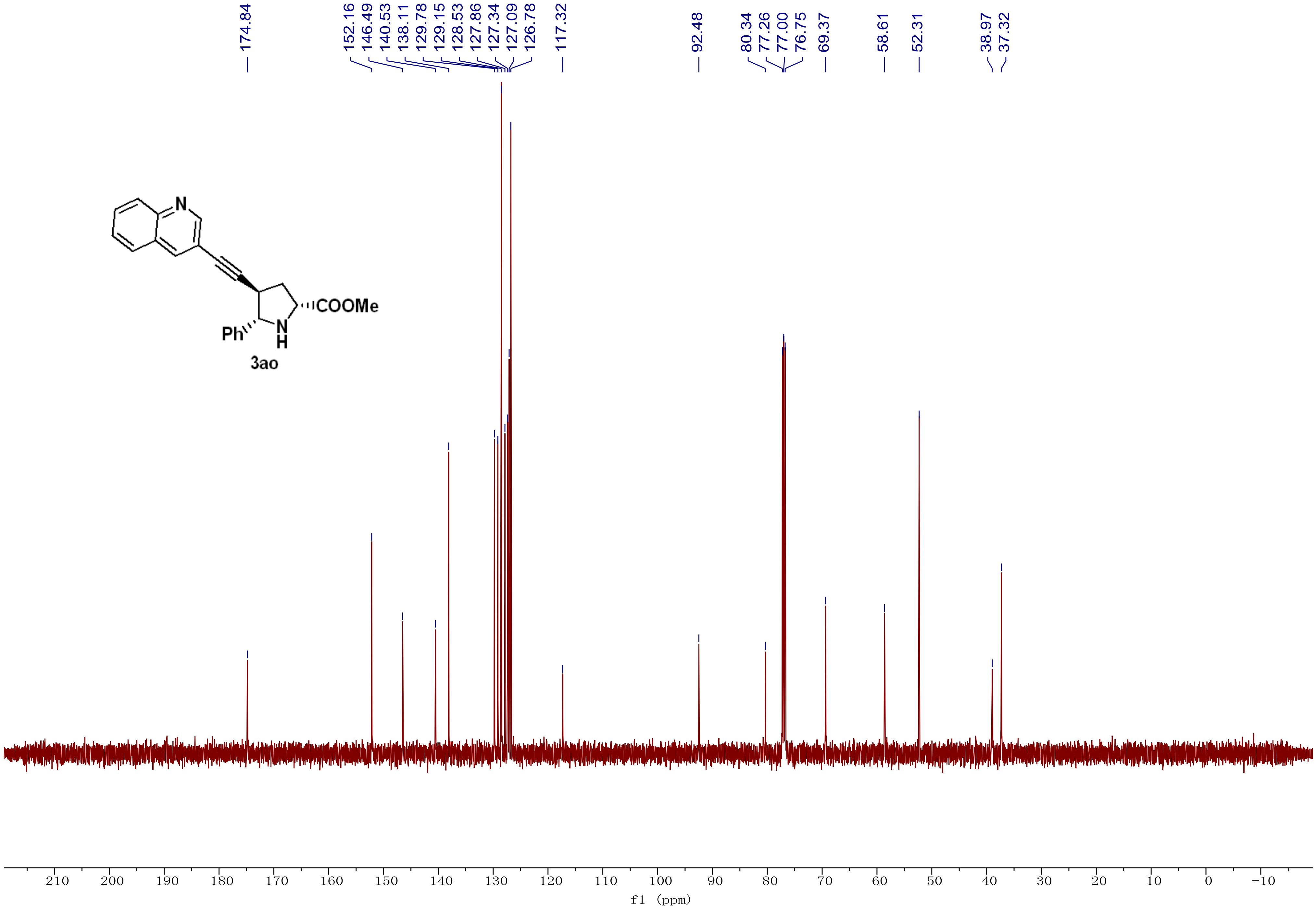
174.84
152.16
146.49
140.53
138.11
129.78
129.15
128.53
127.86
127.34
127.09
126.78
117.32
92.48
80.34
77.26
77.00
76.75
69.37
58.61
52.31
38.97
37.32
N
COOMe
Ph
N
H
3ao
210 200 190 180 170 160 150 140 130 120 110 100 90 80 70 60 50 40 30 20 10 0 −10
f1 (ppm)

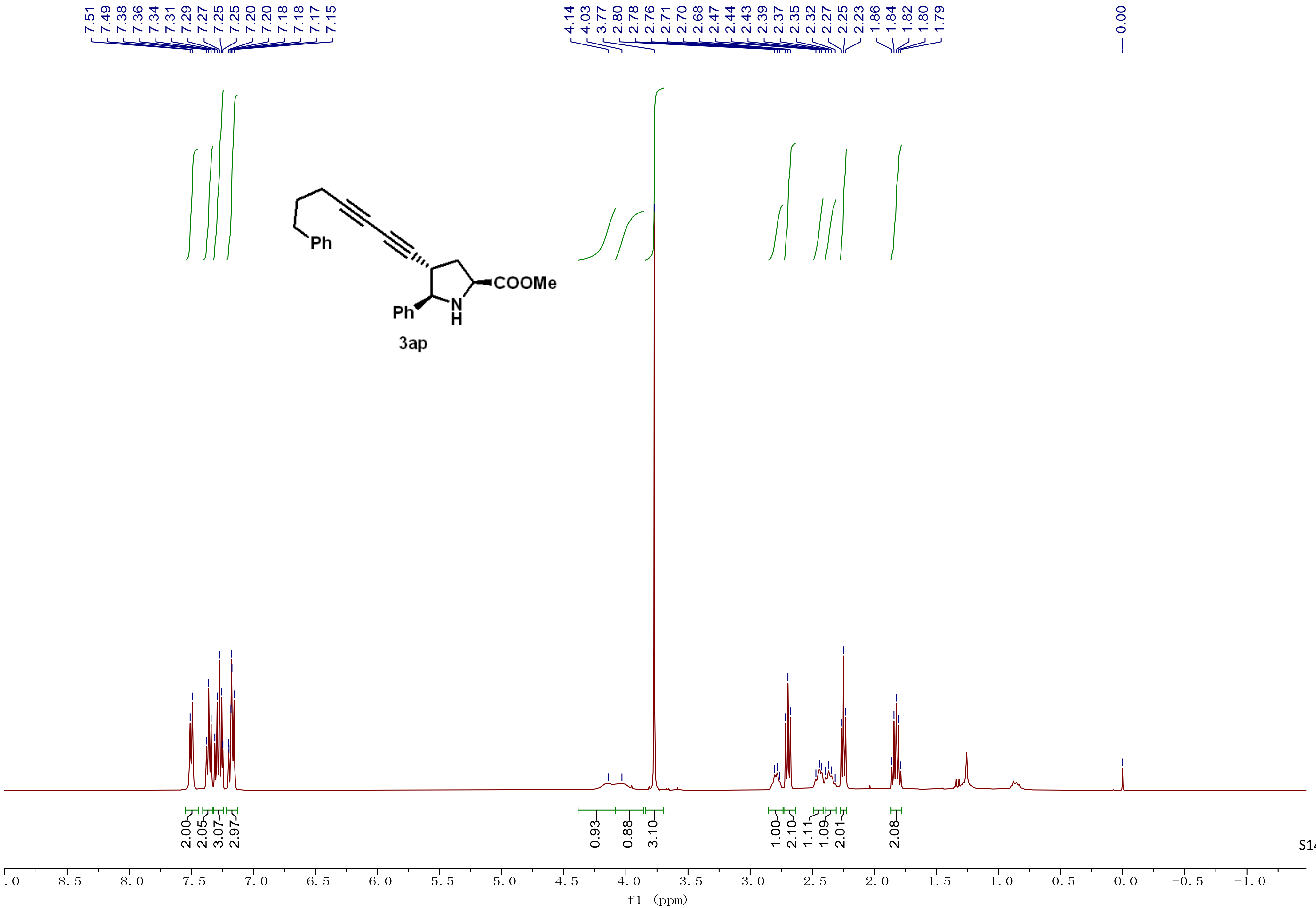
7.51 7.49 7.38 7.36 7.34 7.31 7.29 7.27 7.25 7.20 7.20 7.18 7.18 7.17 7.15
4.14 4.03 3.77 2.80 2.78 2.76 2.71 2.70 2.68 2.47 2.44 2.43 2.39 2.37 2.35 2.32 2.27 2.25 2.23 1.86 1.84 1.82 1.80 1.79
0.00
Ph
Ph
N
H
COOMe
3ap
2.00 2.05 3.07 2.97
0.93 0.88 3.10
1.00 2.10 1.11 1.09 2.01
2.08
f1 (ppm)

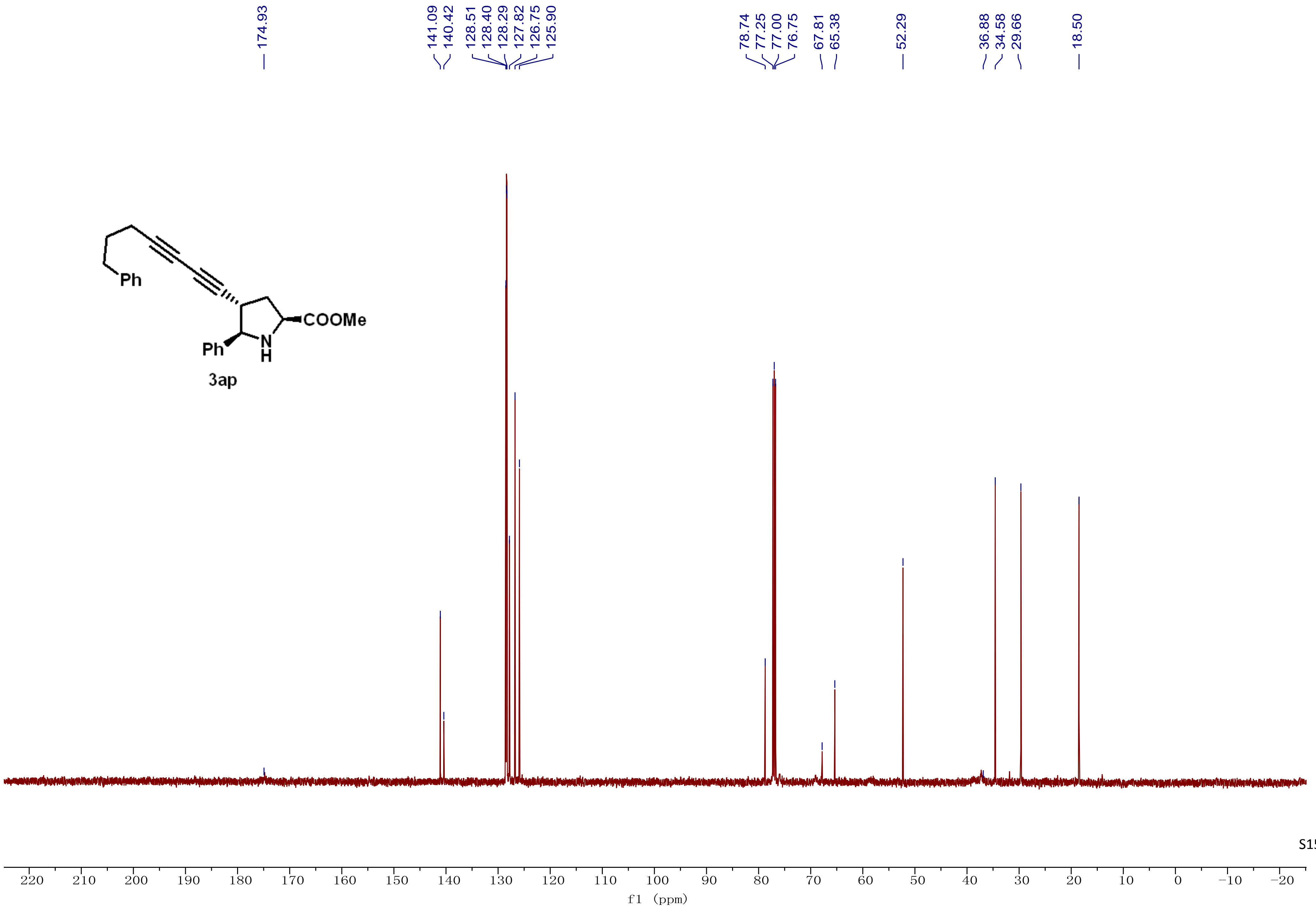
174.93
141.09
140.42
128.51
128.40
128.29
127.82
126.75
125.90
78.74
77.25
77.00
76.75
67.81
65.38
52.29
36.88
34.58
29.66
18.50
Ph
COOMe
Ph
N
H
3ap
220 210 200 190 180 170 160 150 140 130 120 110 100 90 80 70 60 50 40 30 20 10 0 -10 -20
f1 (ppm)

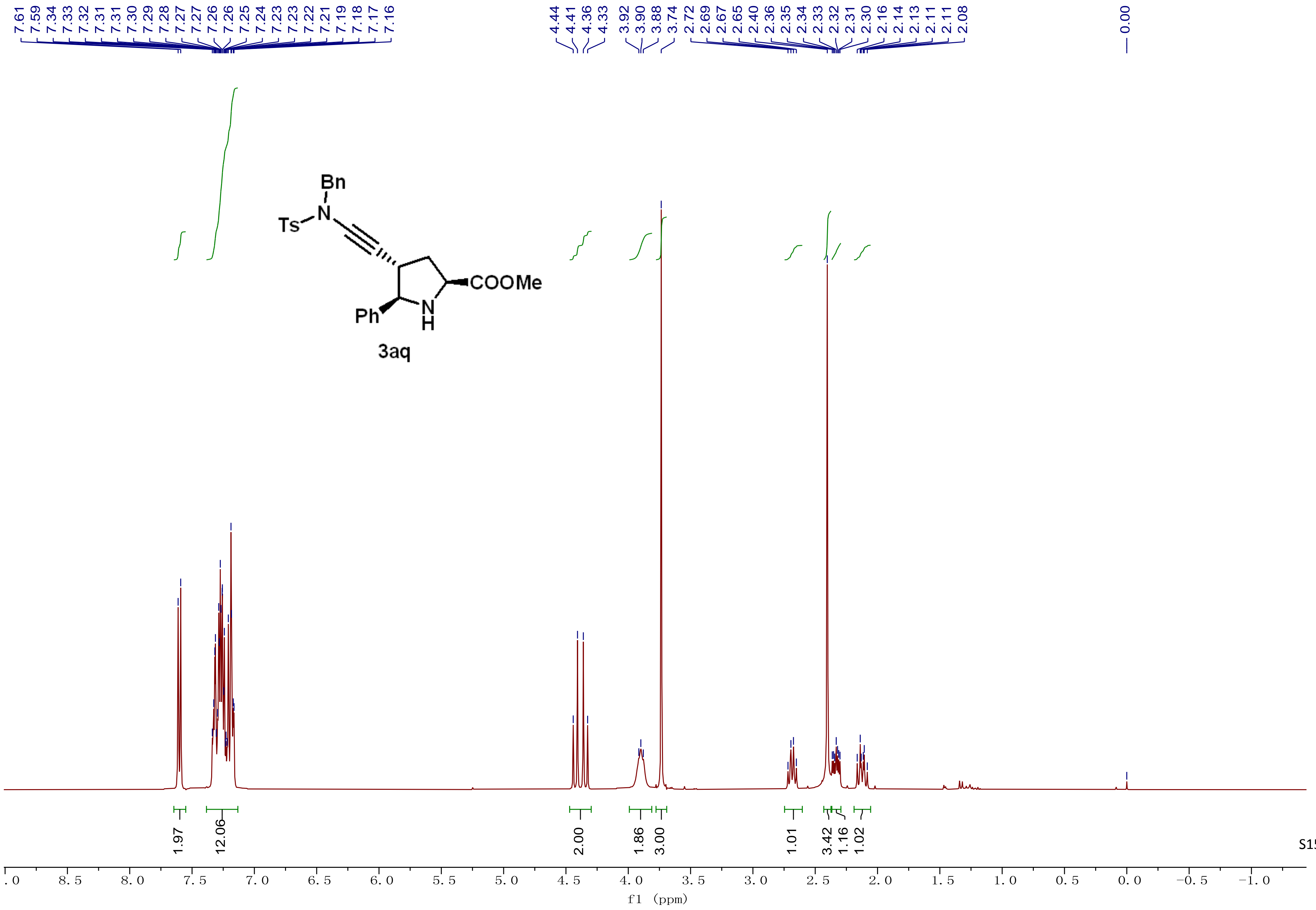
7.61 7.59 7.34 7.33 7.32 7.31 7.31 7.30 7.29 7.28 7.27 7.27 7.26 7.26 7.25 7.24 7.23 7.23 7.22 7.21 7.19 7.18 7.17 7.16
4.44 4.41 4.36 4.33 3.92 3.90 3.88 3.74 2.72 2.69 2.67 2.65 2.40 2.36 2.35 2.34 2.33 2.32 2.31 2.30 2.16 2.14 2.13 2.11 2.11 2.08
0.00
Bn
Ts
N
COOMe
Ph
N
H
3aq
1.97
12.06
2.00
1.86
3.00
1.01
3.42
1.16
1.02
.0 8.5 8.0 7.5 7.0 6.5 6.0 5.5 5.0 4.5 4.0 3.5 3.0 2.5 2.0 1.5 1.0 0.5 0.0 −0.5 −1.0
f1 (ppm)

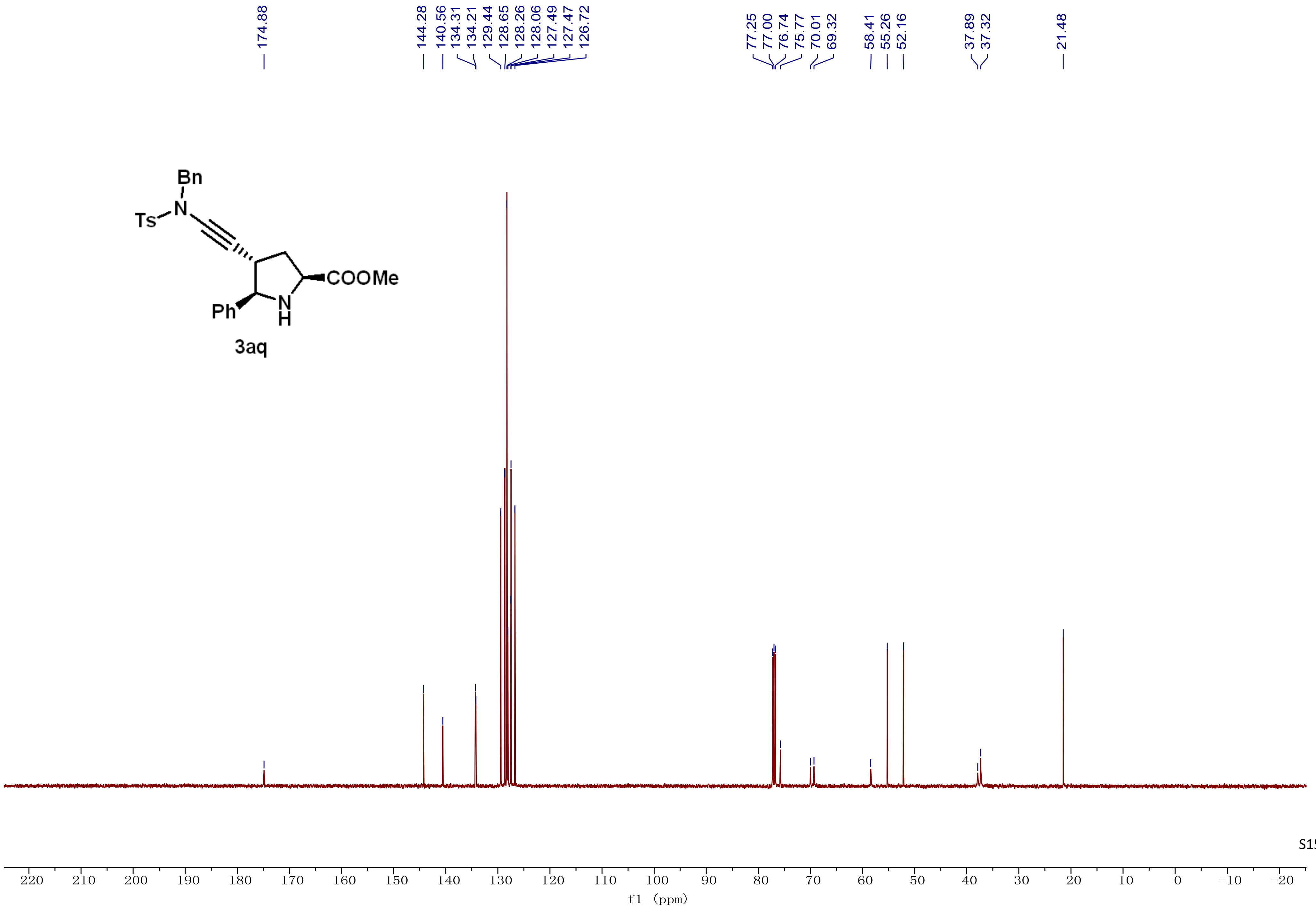

174.88
144.28
140.56
134.31
134.21
129.44
128.65
128.26
128.06
127.49
127.47
126.72
77.25
77.00
76.74
75.77
70.01
69.32
58.41
55.26
52.16
37.89
37.32
21.48
Bn
Ts
N
COOMe
Ph
N
H
3aq
220 210 200 190 180 170 160 150 140 130 120 110 100 90 80 70 60 50 40 30 20 10 0 −10 −20
f1 (ppm)

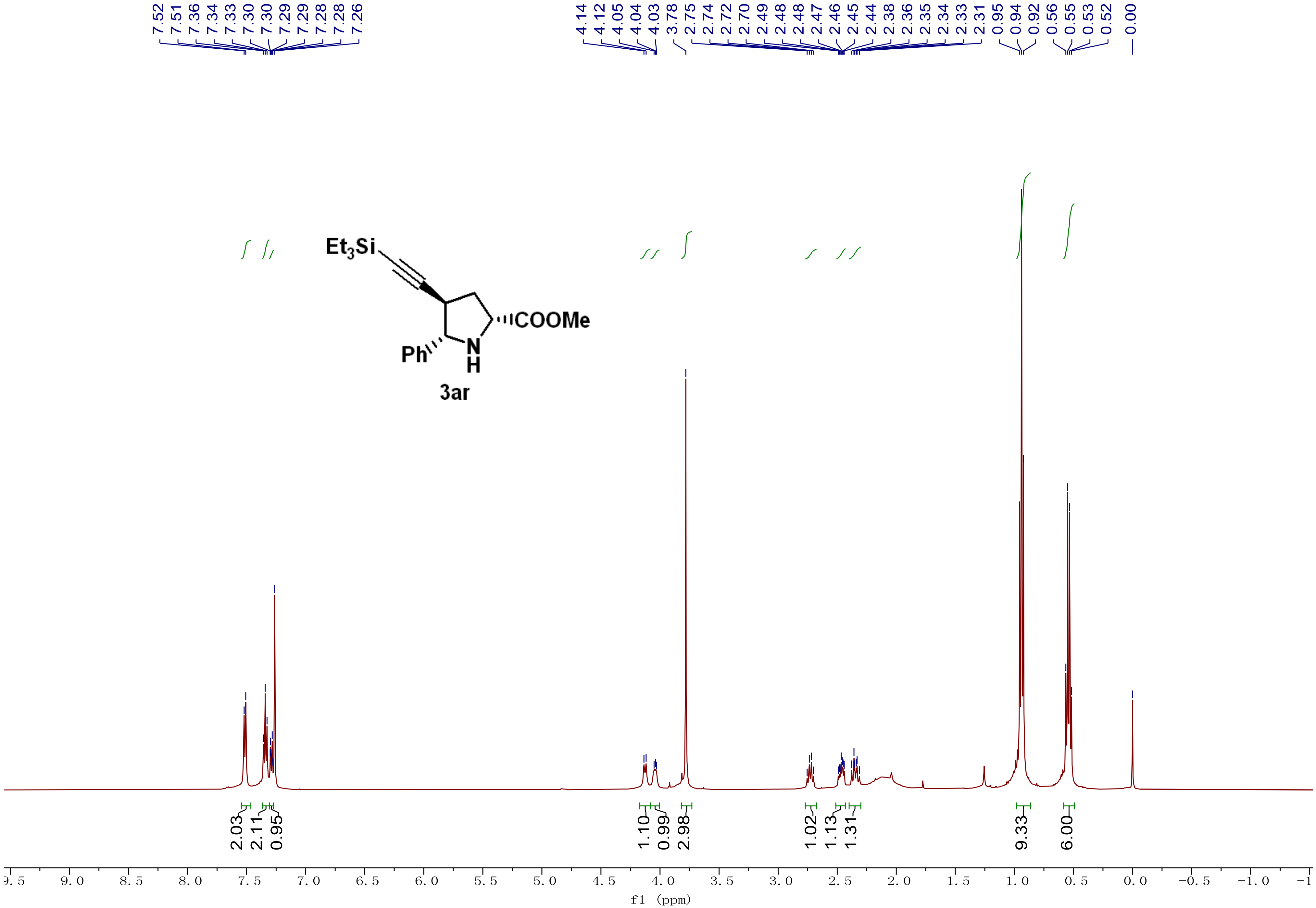

7.52
7.51
7.36
7.34
7.33
7.30
7.30
7.29
7.29
7.28
7.28
7.26
4.14
4.12
4.05
4.04
4.03
3.78
2.75
2.74
2.72
2.70
2.49
2.48
2.48
2.47
2.46
2.45
2.44
2.38
2.36
2.35
2.34
2.33
2.31
0.95
0.94
0.92
0.56
0.55
0.53
0.52
0.00
Et3Si
COOMe
Ph
N
H
3ar
2.03
2.11
0.95
1.10
0.99
2.98
1.02
1.13
1.31
9.33
6.00
f1 (ppm)

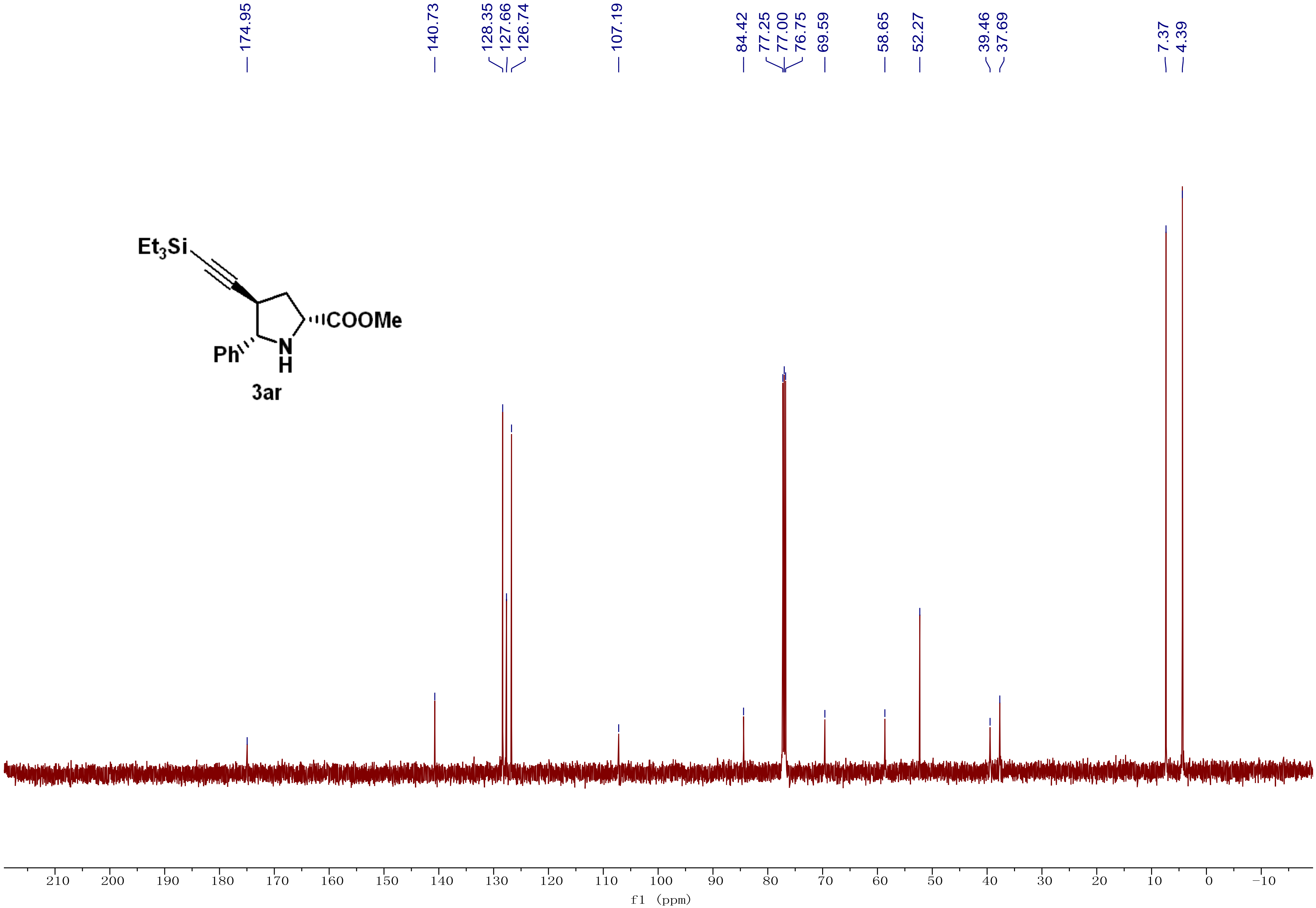
174.95
140.73
128.35
127.66
126.74
107.19
84.42
77.25
77.00
76.75
69.59
58.65
52.27
39.46
37.69
7.37
4.39
Et3Si
COOMe
Ph
N
H
3ar
210 200 190 180 170 160 150 140 130 120 110 100 90 80 70 60 50 40 30 20 10 0 −10
f1 (ppm)

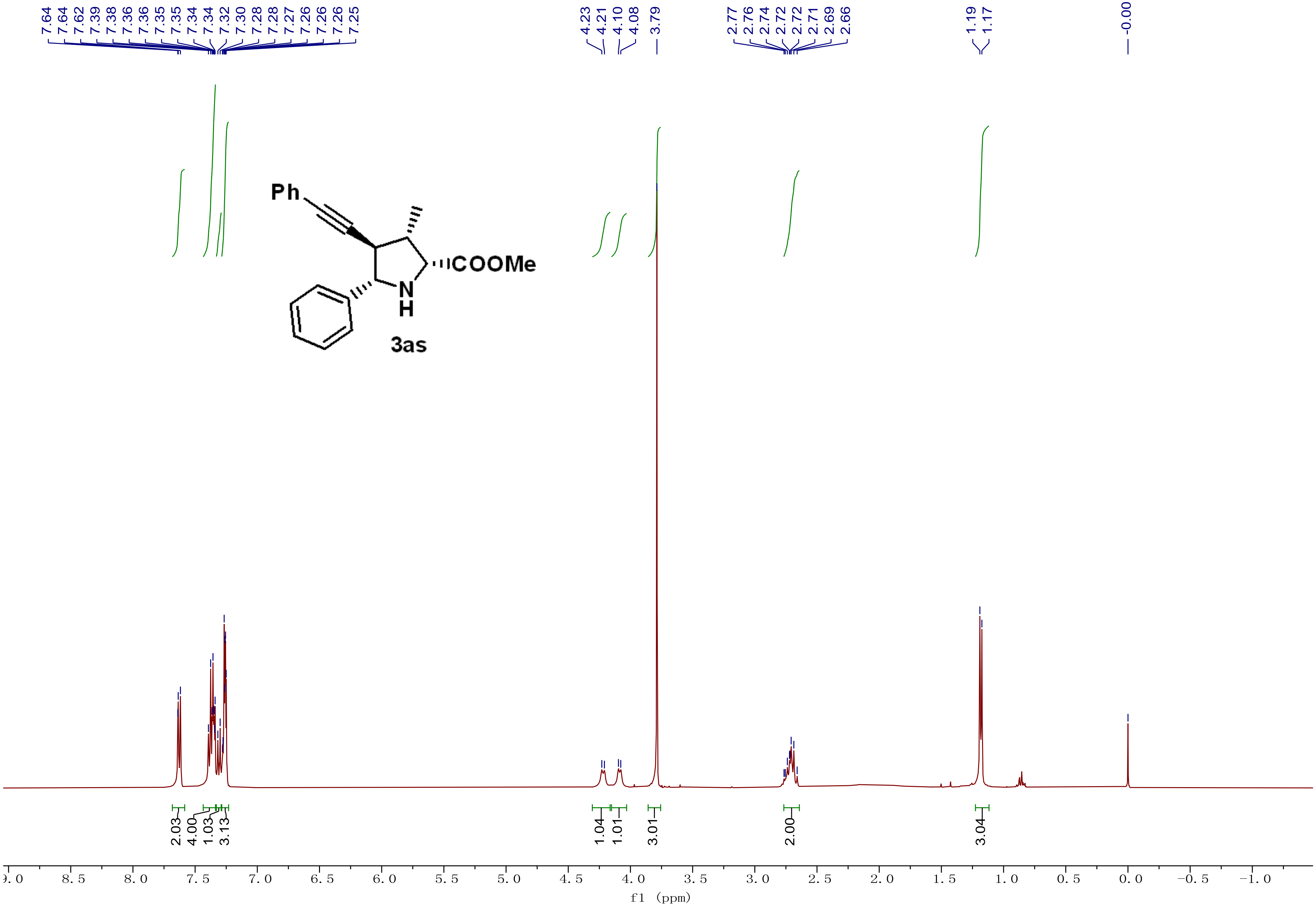
7.64 7.64 7.62 7.39 7.38 7.36 7.36 7.35 7.35 7.34 7.34 7.32 7.30 7.28 7.28 7.27 7.26 7.26 7.26 7.25
4.23 4.21 4.10 4.08 3.79
2.77 2.76 2.74 2.72 2.72 2.71 2.69 2.66
1.19 1.17
-0.00
Ph
COOMe
N
H
3as
2.03 4.00 1.03 3.13
1.04 1.01 3.01
2.00
3.04
9.0 8.5 8.0 7.5 7.0 6.5 6.0 5.5 5.0 4.5 4.0 3.5 3.0 2.5 2.0 1.5 1.0 0.5 0.0 -0.5 -1.0
f1 (ppm)

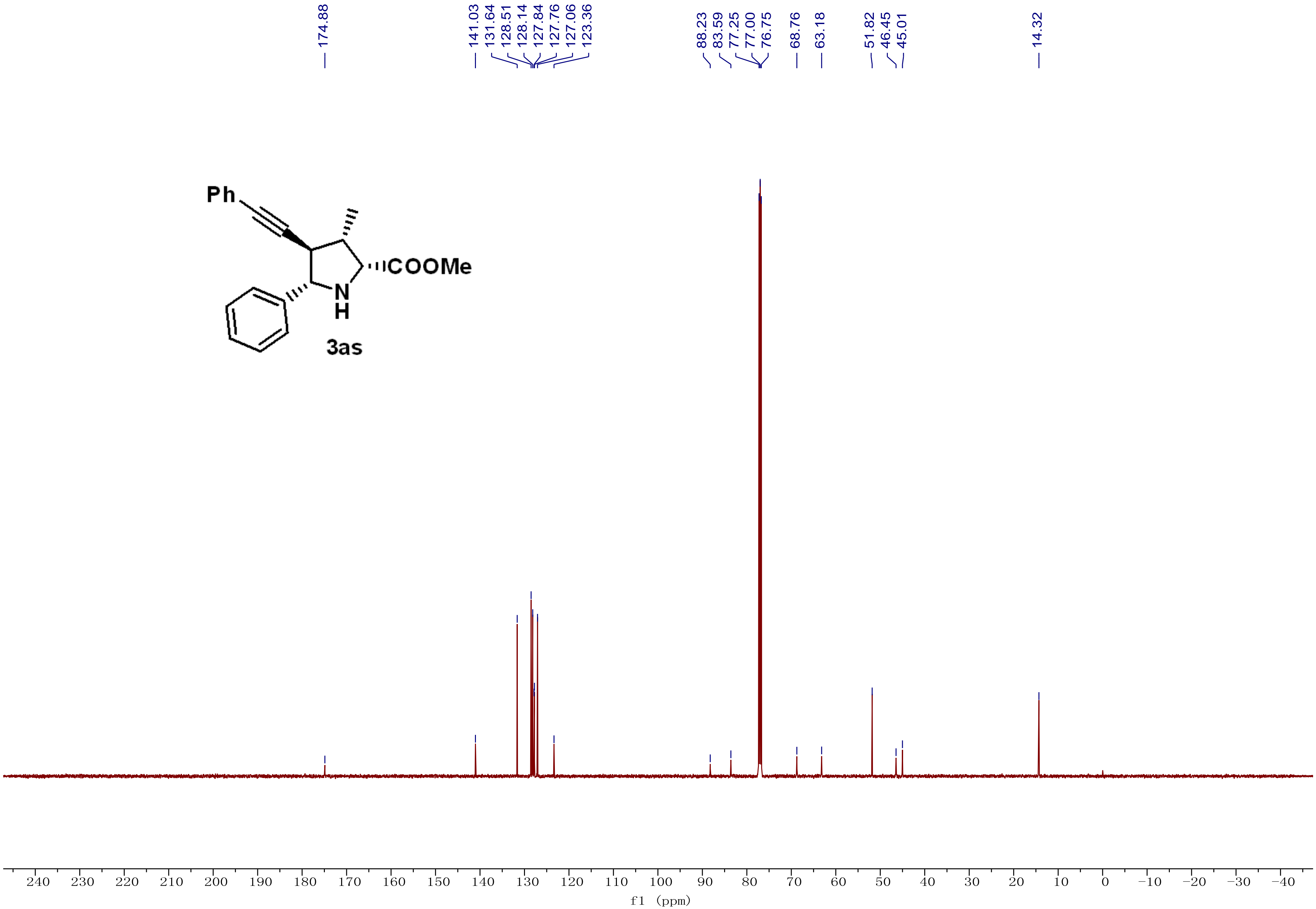

174.88
141.03
131.64
128.51
128.14
127.84
127.76
127.06
123.36
88.23
83.59
77.25
77.00
76.75
68.76
63.18
51.82
46.45
45.01
14.32
Ph
COOMe
N
H
3as
240 230 220 210 200 190 180 170 160 150 140 130 120 110 100 90 80 70 60 50 40 30 20 10 0 −10 −20 −30 −40
f1 (ppm)

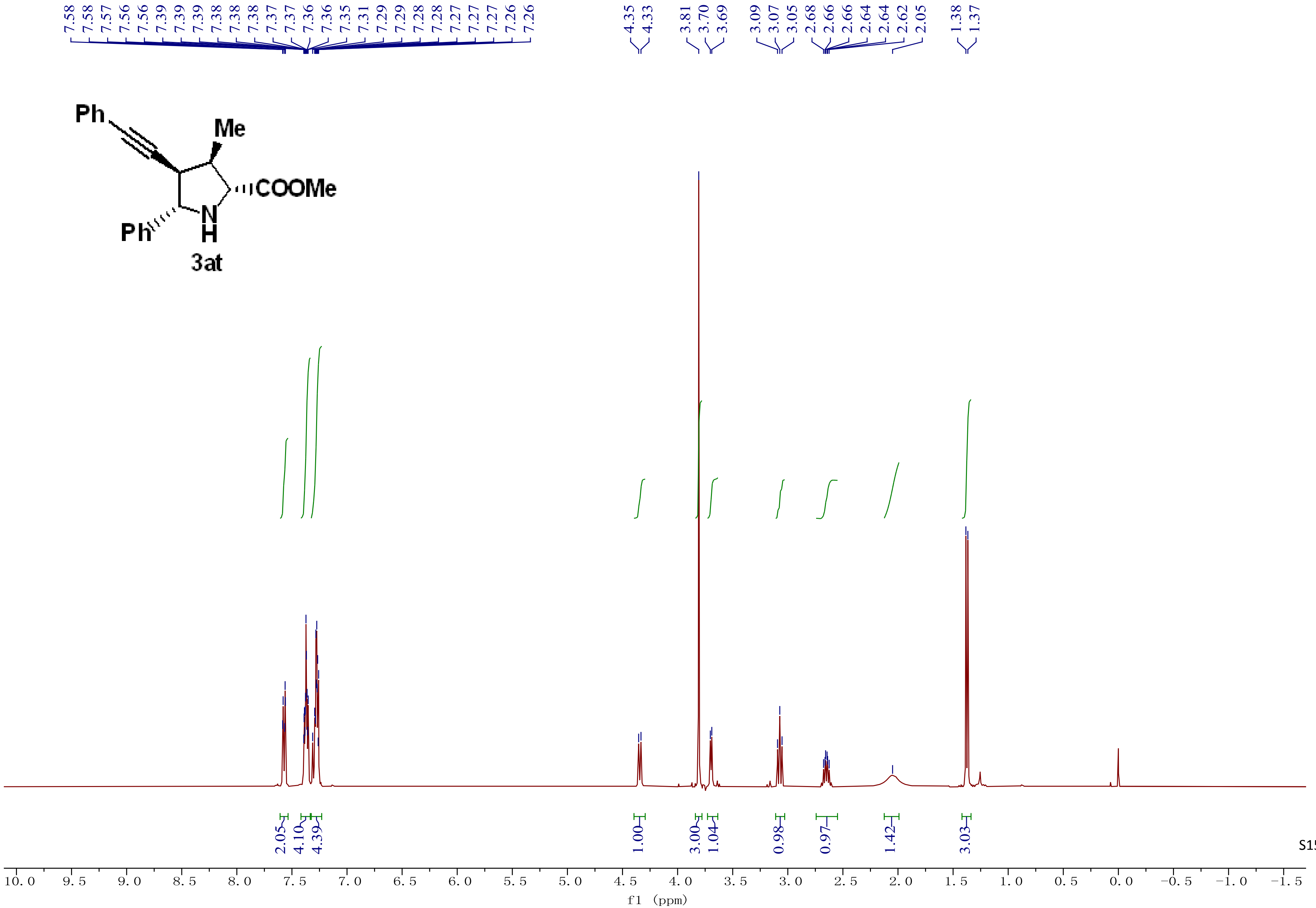

7.58
7.58
7.57
7.56
7.56
7.39
7.39
7.39
7.38
7.38
7.38
7.37
7.37
7.36
7.36
7.35
7.31
7.29
7.29
7.28
7.28
7.27
7.27
7.27
7.26
7.26
4.35
4.33
3.81
3.70
3.69
3.09
3.07
3.05
2.68
2.66
2.66
2.64
2.64
2.62
2.05
1.38
1.37
Ph
Me
COOMe
Ph
N
H
3at
2.05
4.10
4.39
1.00
3.00
1.04
0.98
0.97
1.42
3.03
f1 (ppm)

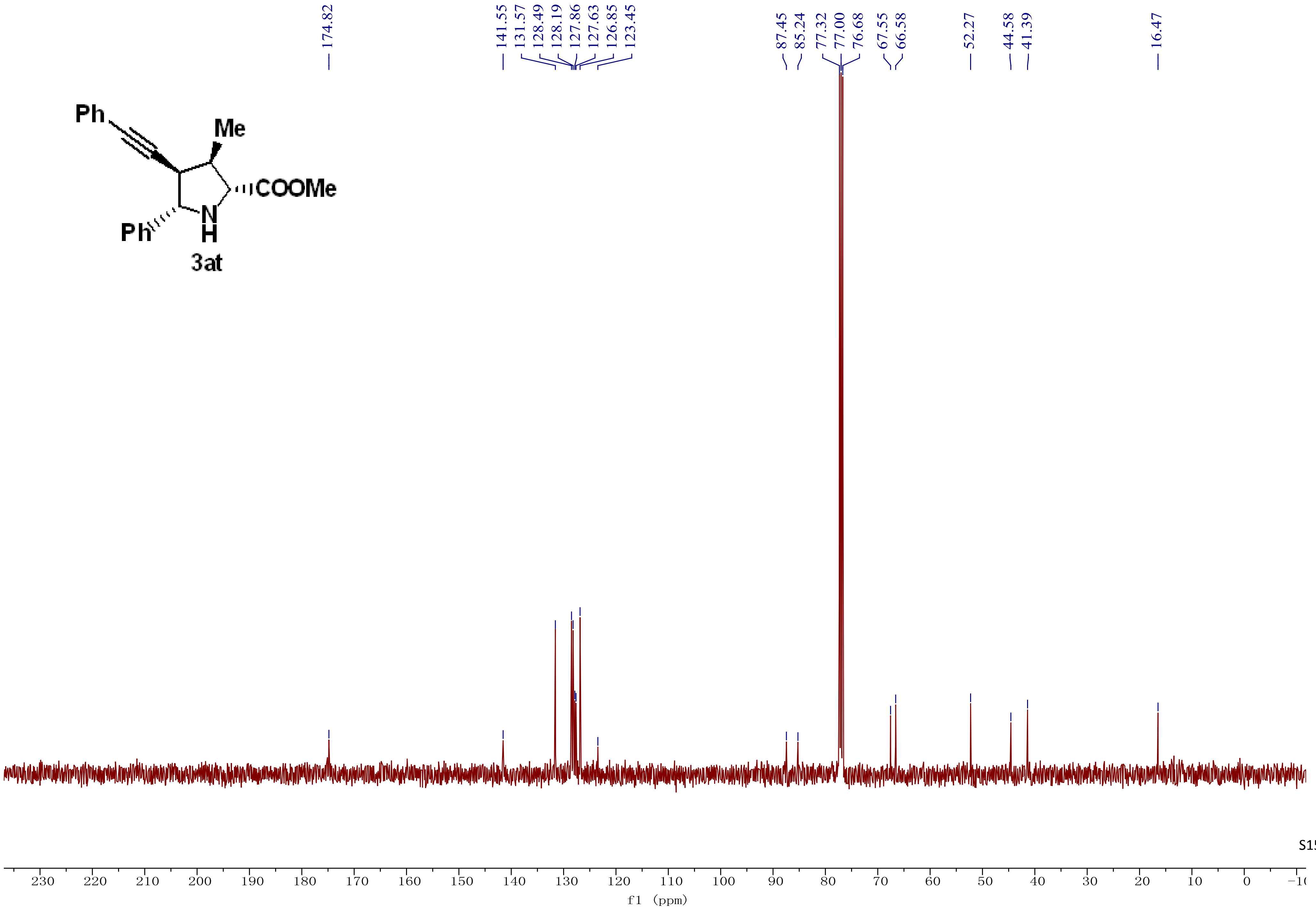
Ph
Me
COOMe
Ph
N
H
3at
174.82
141.55
131.57
128.49
128.19
127.86
127.63
126.85
123.45
87.45
85.24
77.32
77.00
76.68
67.55
66.58
52.27
44.58
41.39
16.47
230 220 210 200 190 180 170 160 150 140 130 120 110 100 90 80 70 60 50 40 30 20 10 0 -10
f1 (ppm)

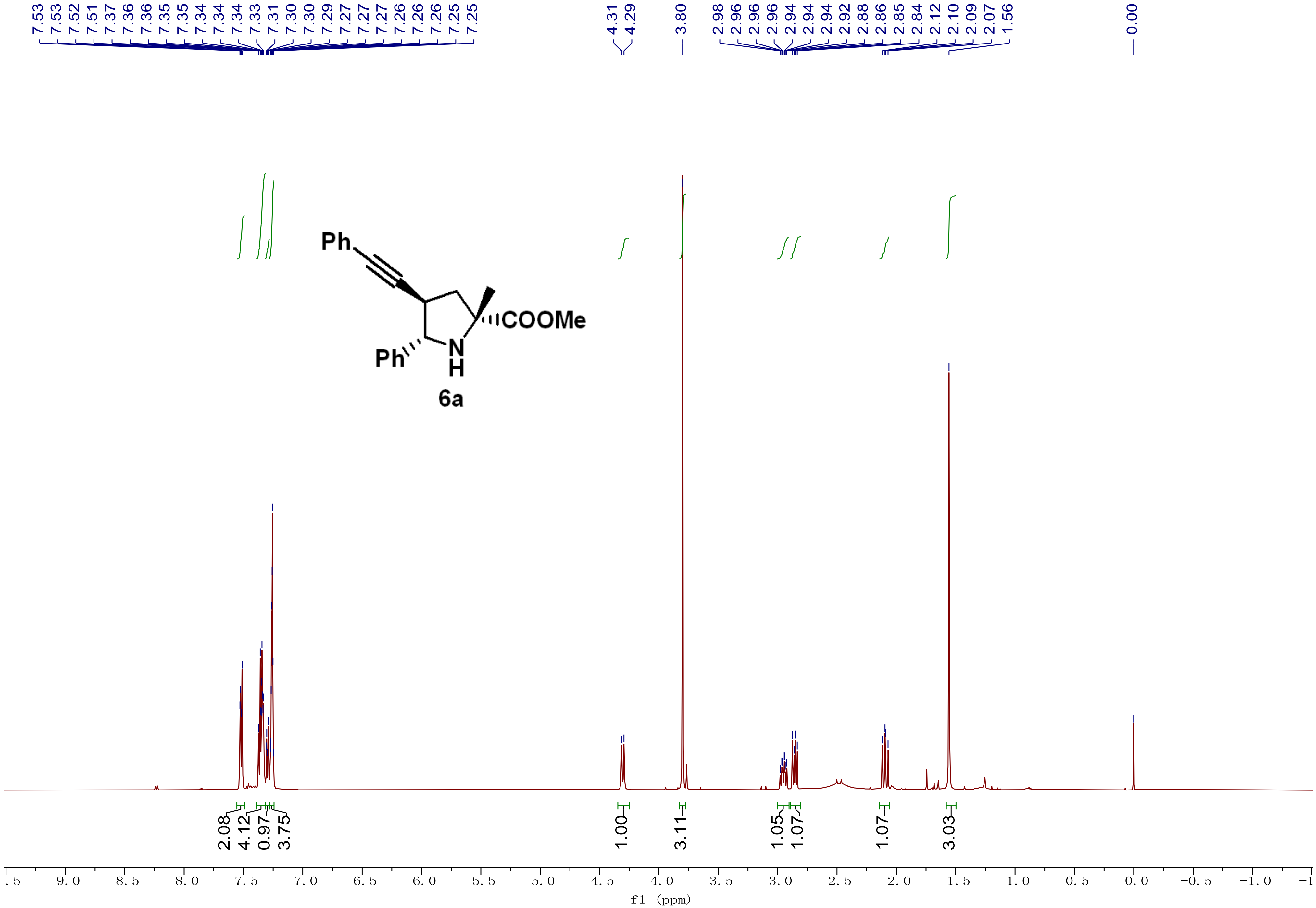
7.53 7.53 7.52 7.51 7.37 7.36 7.36 7.35 7.35 7.34 7.34 7.34 7.33 7.31 7.30 7.30 7.29 7.27 7.27 7.27 7.26 7.26 7.26 7.25 7.25
4.31 4.29
3.80
2.98 2.96 2.96 2.96 2.94 2.94 2.94 2.92 2.88 2.86 2.85 2.84 2.12 2.10 2.09 2.07 1.56
0.00
Ph
COOMe
Ph
N
H
6a
2.08 4.12 0.97 3.75
1.00
3.11
1.05 1.07
1.07
3.03
9.0 8.5 8.0 7.5 7.0 6.5 6.0 5.5 5.0 4.5 4.0 3.5 3.0 2.5 2.0 1.5 1.0 0.5 0.0 −0.5 −1.0
f1 (ppm)

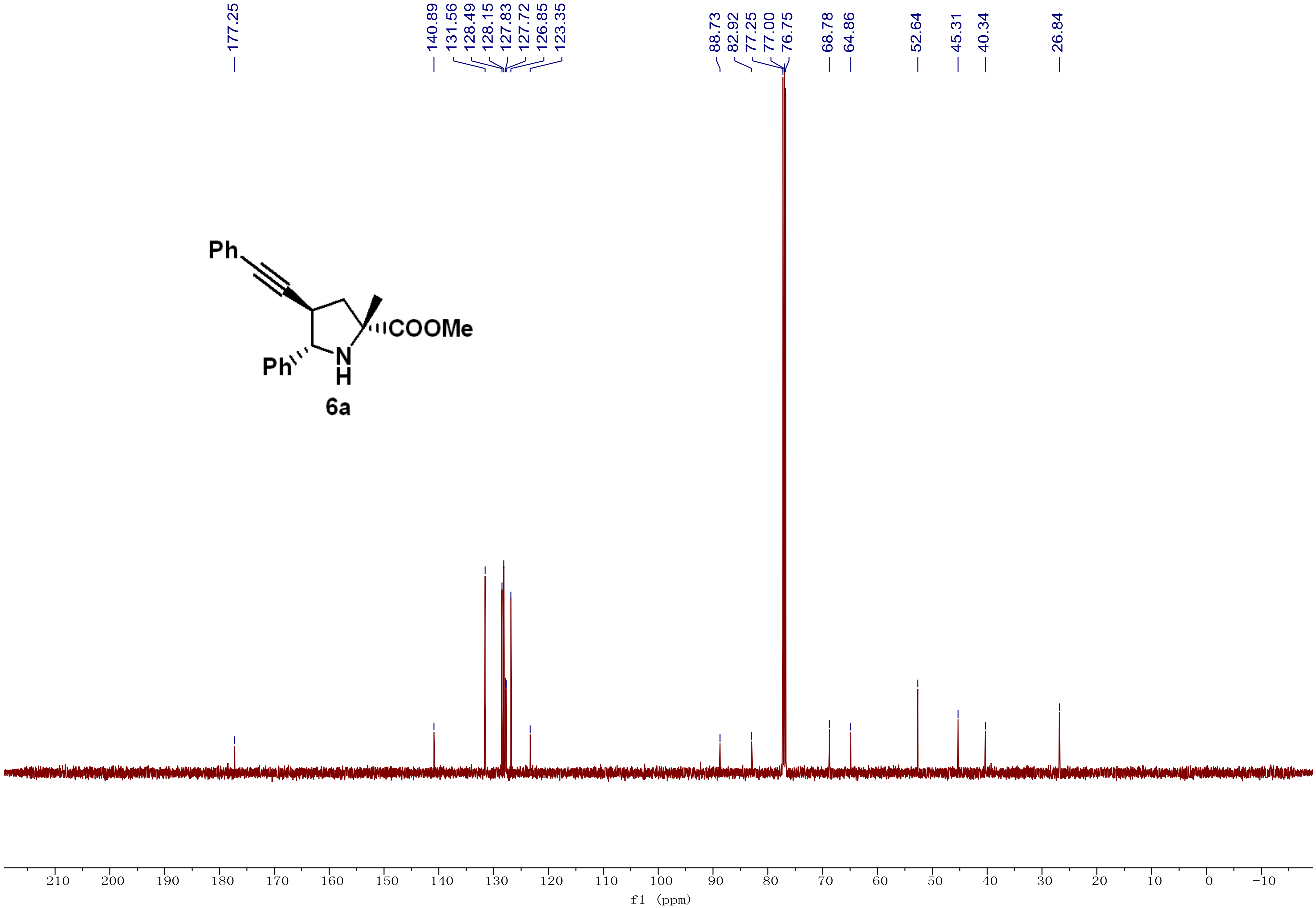
177.25
140.89
131.56
128.49
128.15
127.83
127.72
126.85
123.35
88.73
82.92
77.25
77.00
76.75
68.78
64.86
52.64
45.31
40.34
26.84
Ph
COOMe
Ph
N
H
6a
210 200 190 180 170 160 150 140 130 120 110 100 90 80 70 60 50 40 30 20 10 0 −10
f1 (ppm)

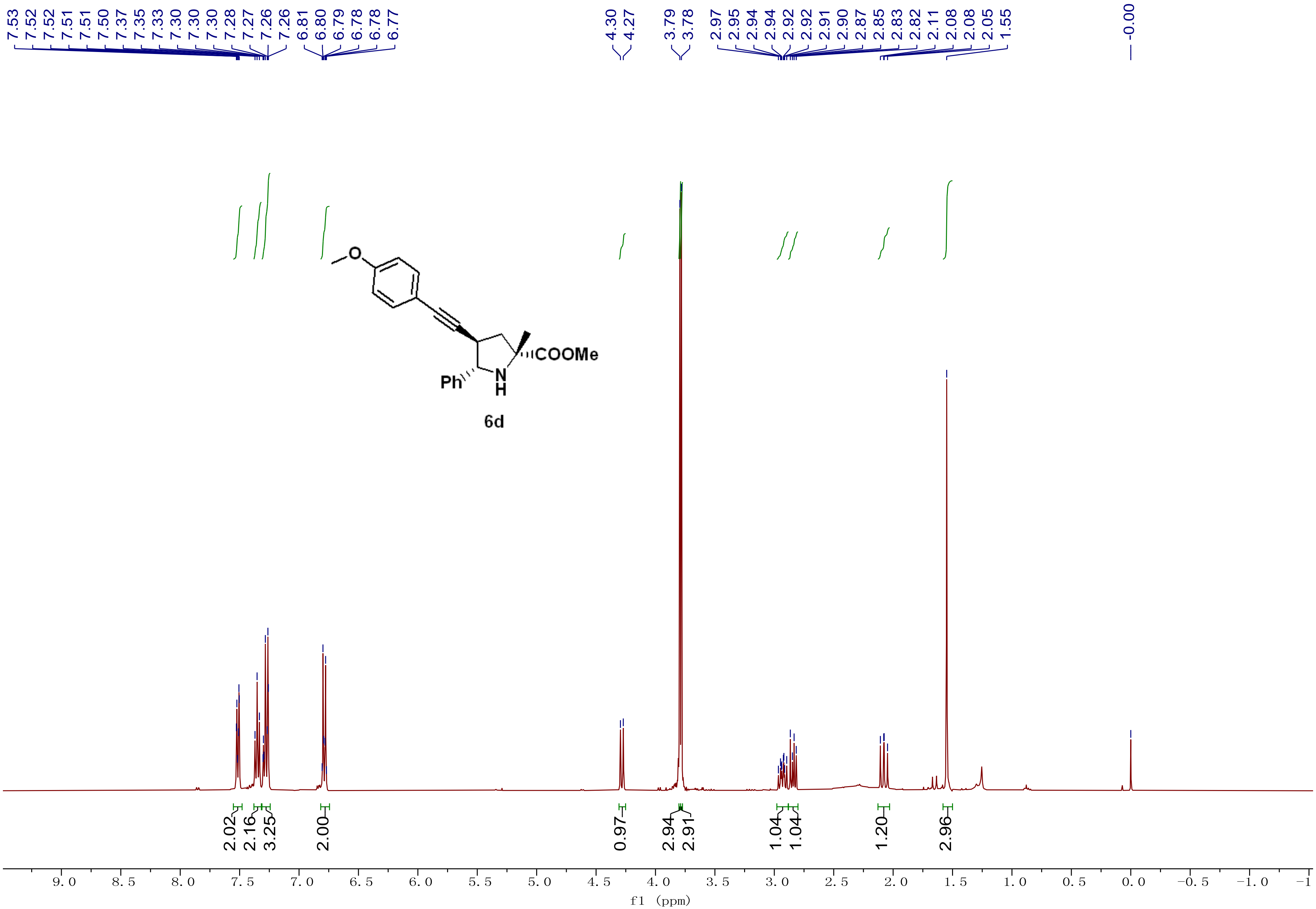
7.53 7.52 7.52 7.51 7.51 7.50 7.37 7.35 7.33 7.30 7.30 7.30 7.28 7.27 7.26 7.26 6.81 6.80 6.79 6.78 6.78 6.77
4.30 4.27
3.79 3.78
2.97 2.95 2.94 2.94 2.92 2.92 2.91 2.90 2.87 2.85 2.83 2.82 2.11 2.08 2.08 2.05 1.55
-0.00
O
COOMe
Ph
N
H
6d
2.02 2.16 3.25 2.00 0.97 2.94 2.91 1.04 1.04 1.20 2.96
9.0 8.5 8.0 7.5 7.0 6.5 6.0 5.5 5.0 4.5 4.0 3.5 3.0 2.5 2.0 1.5 1.0 0.5 0.0 −0.5 −1.0 −1
f1 (ppm)

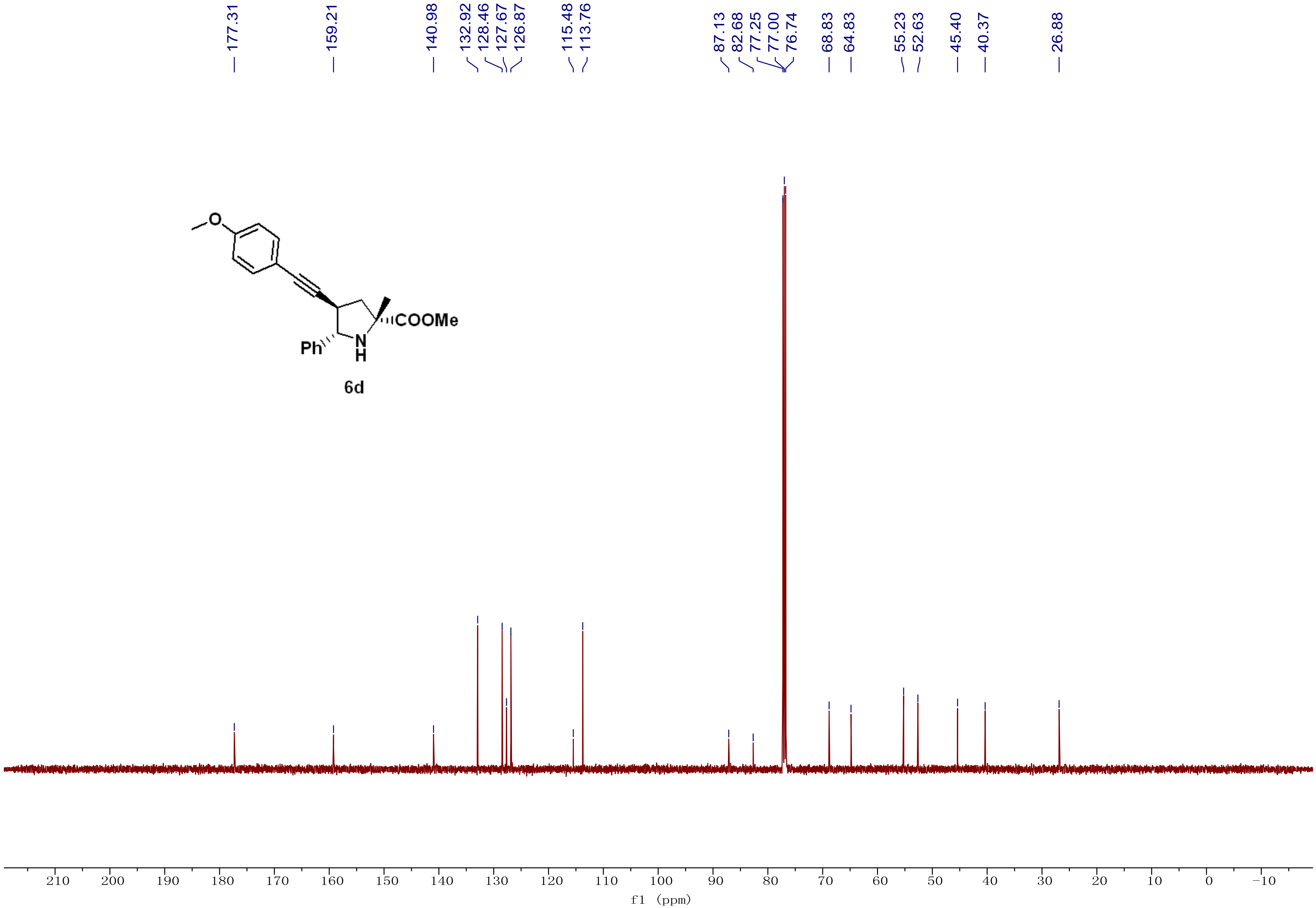
177.31
159.21
140.98
132.92
128.46
127.67
126.87
115.48
113.76
87.13
82.68
77.25
77.00
76.74
68.83
64.83
55.23
52.63
45.40
40.37
26.88
COOMe
Ph
N
H
6d
210 200 190 180 170 160 150 140 130 120 110 100 90 80 70 60 50 40 30 20 10 0 −10
f1 (ppm)

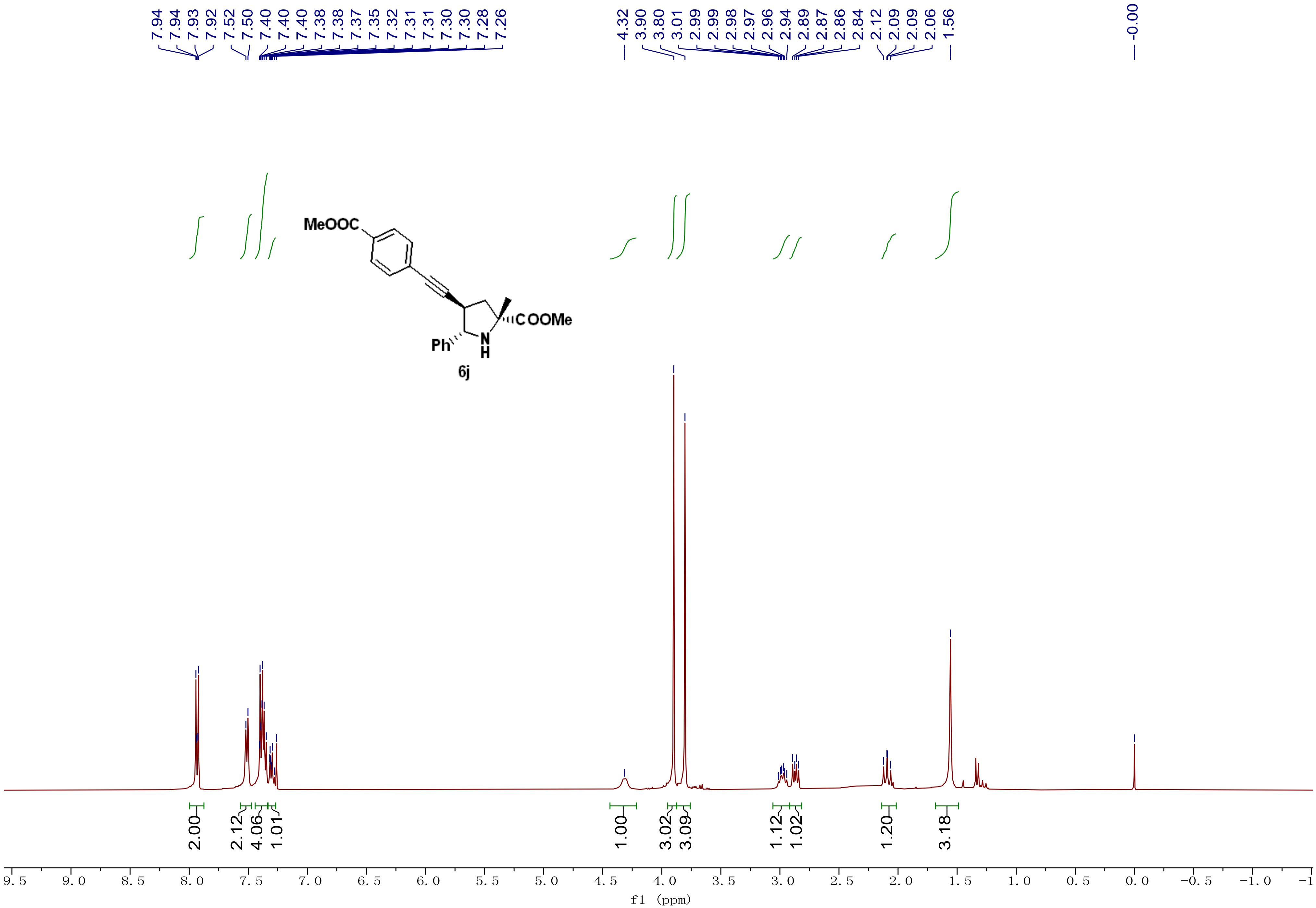
7.94 7.94 7.93 7.92 7.52 7.50 7.40 7.40 7.40 7.38 7.38 7.37 7.35 7.32 7.31 7.31 7.30 7.30 7.28 7.26
4.32 3.90 3.80 3.01 2.99 2.99 2.98 2.97 2.96 2.94 2.89 2.87 2.86 2.84 2.12 2.09 2.09 2.06 1.56
-0.00
MeOOC
COOMe
Ph
N
H
6j
2.00 2.12 4.06 1.01 1.00 3.02 3.09 1.12 1.02 1.20 3.18
9.5 9.0 8.5 8.0 7.5 7.0 6.5 6.0 5.5 5.0 4.5 4.0 3.5 3.0 2.5 2.0 1.5 1.0 0.5 0.0 -0.5 -1.0 -1
f1 (ppm)

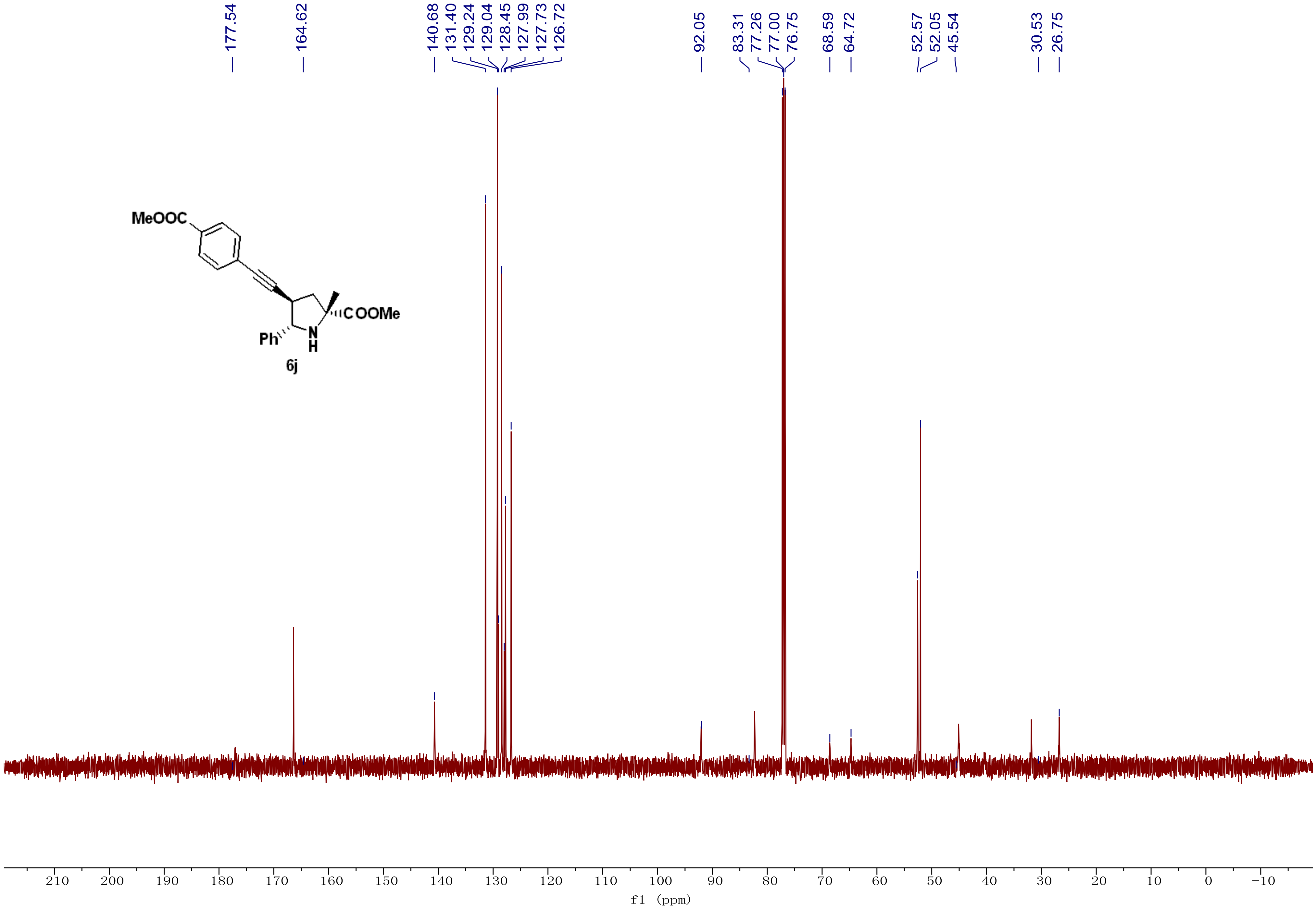
177.54
164.62
140.68
131.40
129.24
129.04
128.45
127.99
127.73
126.72
92.05
83.31
77.26
77.00
76.75
68.59
64.72
52.57
52.05
45.54
30.53
26.75
MeOOC
COOMe
Ph
N
H
6j
210 200 190 180 170 160 150 140 130 120 110 100 90 80 70 60 50 40 30 20 10 0 −10
f1 (ppm)

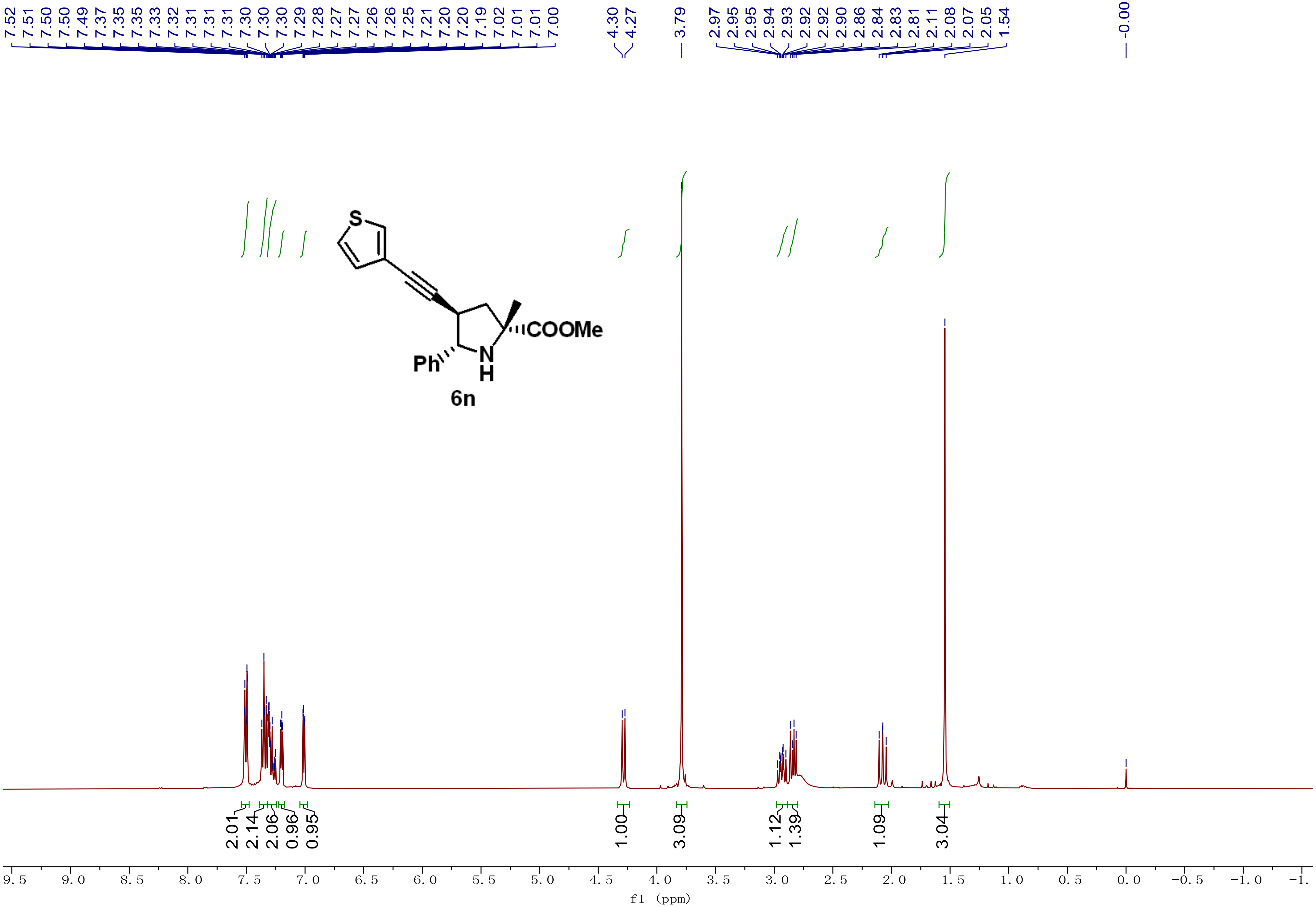
7.52 7.51 7.50 7.50 7.49 7.37 7.35 7.35 7.33 7.32 7.31 7.31 7.31 7.30 7.30 7.30 7.29 7.28 7.27 7.27 7.26 7.26 7.25 7.21 7.20 7.20 7.19 7.02 7.01 7.01 7.00
4.30 4.27
3.79
2.97 2.95 2.95 2.94 2.93 2.92 2.92 2.90 2.86 2.84 2.83 2.81 2.11 2.08 2.07 2.05 1.54
-0.00
S
COOMe
Ph
N
H
6n
2.01 2.14 2.06 0.96 0.95
1.00
3.09
1.12 1.39
1.09
3.04
9.5 9.0 8.5 8.0 7.5 7.0 6.5 6.0 5.5 5.0 4.5 4.0 3.5 3.0 2.5 2.0 1.5 1.0 0.5 0.0 −0.5 −1.0 −1.
f1 (ppm)

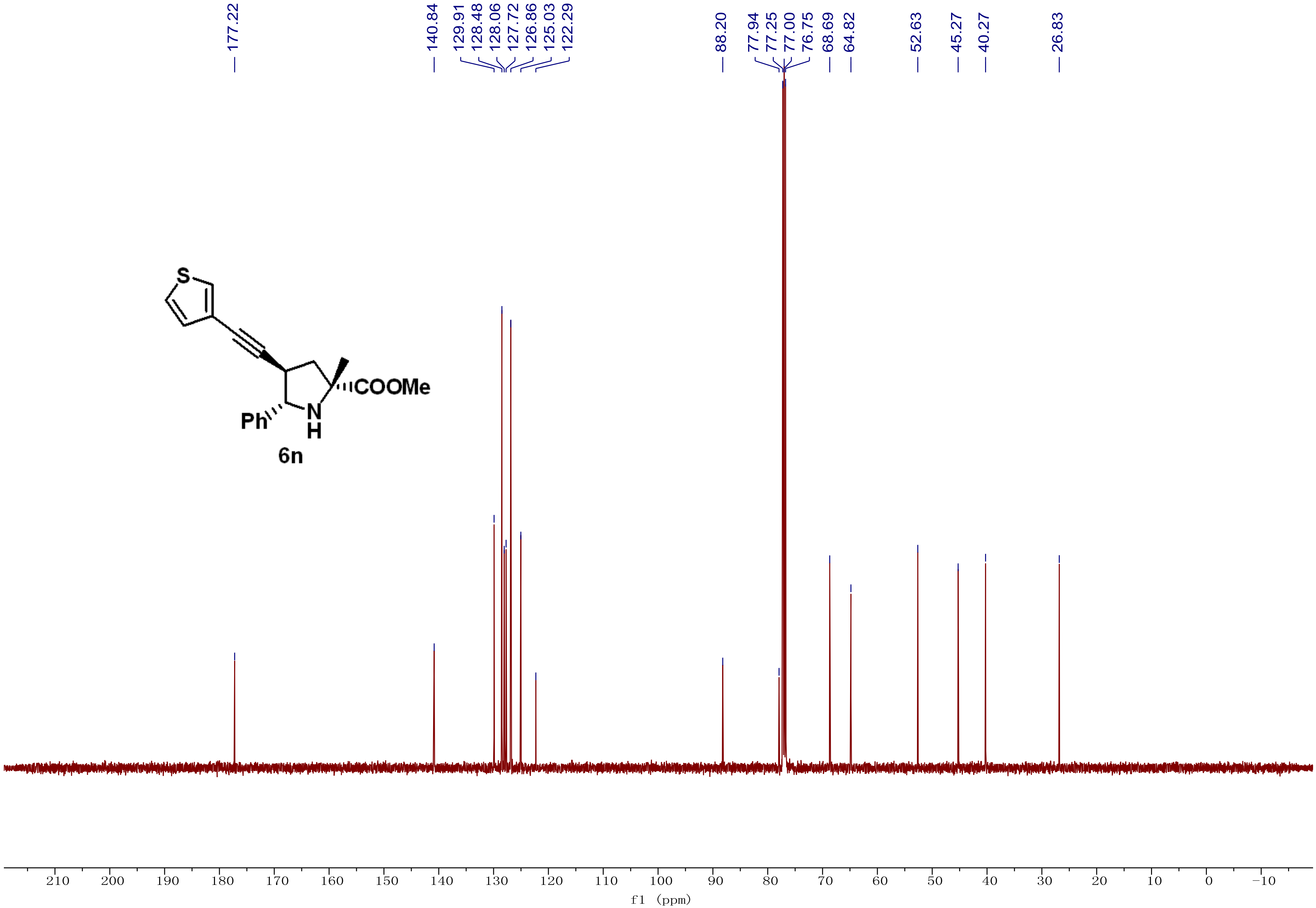
177.22
140.84
129.91
128.48
128.06
127.72
126.86
125.03
122.29
88.20
77.94
77.25
77.00
76.75
68.69
64.82
52.63
45.27
40.27
26.83
S
COOMe
Ph
N
H
6n
210 200 190 180 170 160 150 140 130 120 110 100 90 80 70 60 50 40 30 20 10 0 −10
f1 (ppm)

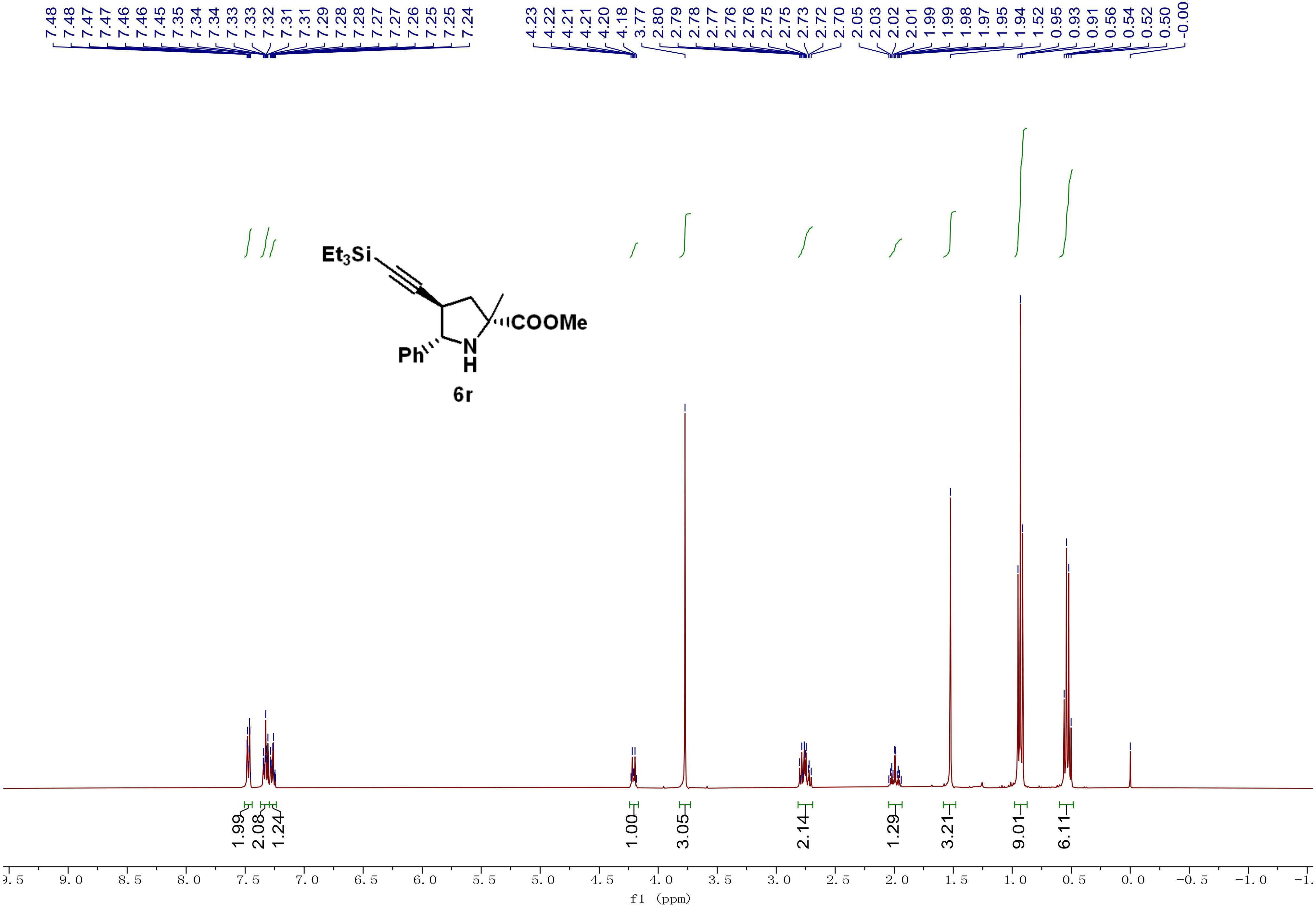
7.48 7.48 7.47 7.47 7.46 7.46 7.45 7.35 7.34 7.34 7.33 7.33 7.32 7.31 7.31 7.29 7.28 7.28 7.27 7.27 7.26 7.25 7.25 7.24
4.23 4.22 4.21 4.21 4.20 4.18 3.77 2.80 2.79 2.78 2.77 2.76 2.76 2.75 2.75 2.73 2.72 2.70 2.05 2.03 2.02 2.01 1.99 1.99 1.98 1.97 1.95 1.94 1.52 0.95 0.93 0.91 0.56 0.54 0.52 0.50 -0.00
Et3Si
COOMe
Ph
N
H
6r
1.99 2.08 1.24 1.00 3.05 2.14 1.29 3.21 9.01 6.11
9.5 9.0 8.5 8.0 7.5 7.0 6.5 6.0 5.5 5.0 4.5 4.0 3.5 3.0 2.5 2.0 1.5 1.0 0.5 0.0 −0.5 −1.0 −1.
f1 (ppm)

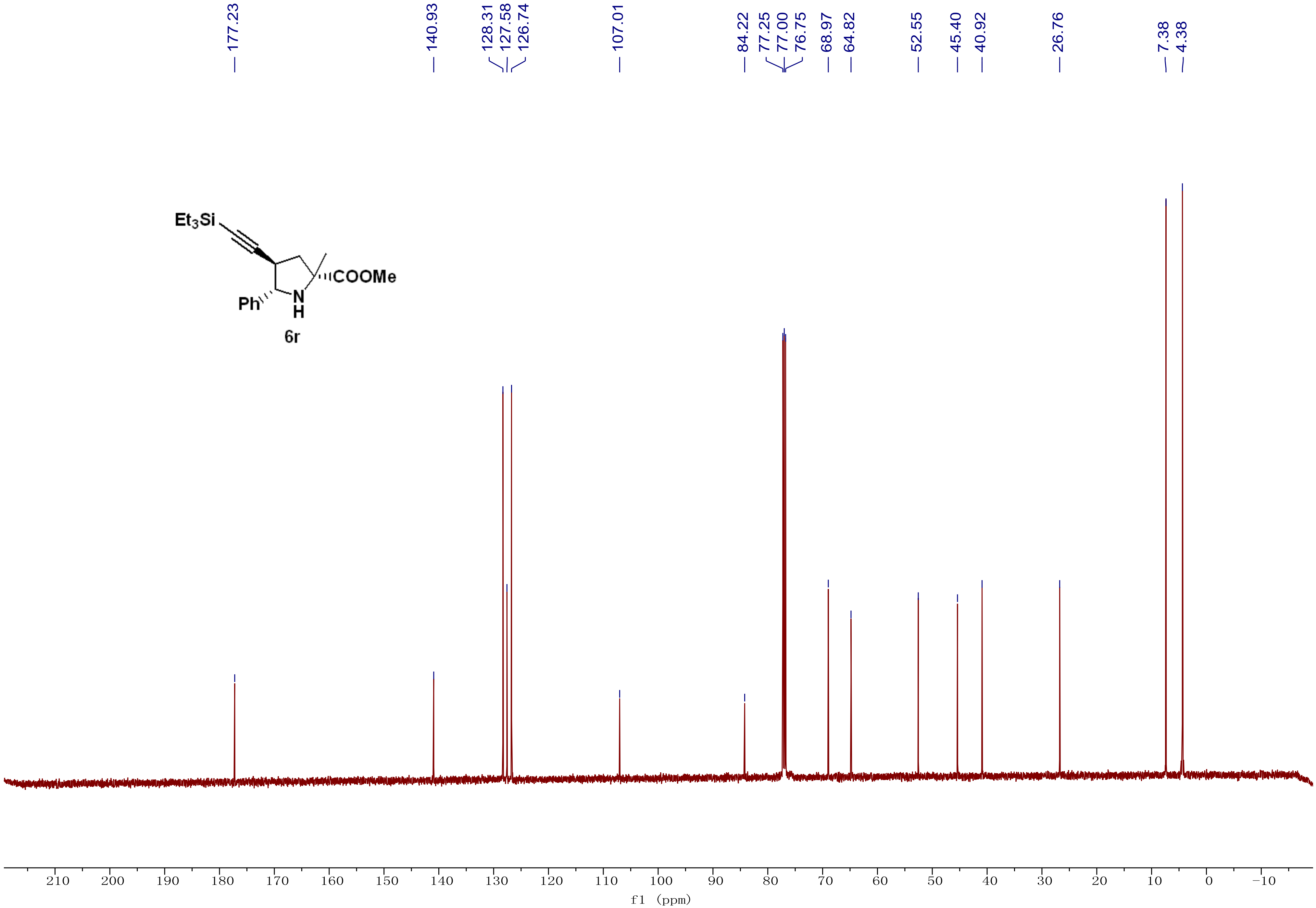
177.23
140.93
128.31
127.58
126.74
107.01
84.22
77.25
77.00
76.75
68.97
64.82
52.55
45.40
40.92
26.76
7.38
4.38
Et3Si
COOMe
Ph
N
H
6r
210 200 190 180 170 160 150 140 130 120 110 100 90 80 70 60 50 40 30 20 10 0 −10
f1 (ppm)

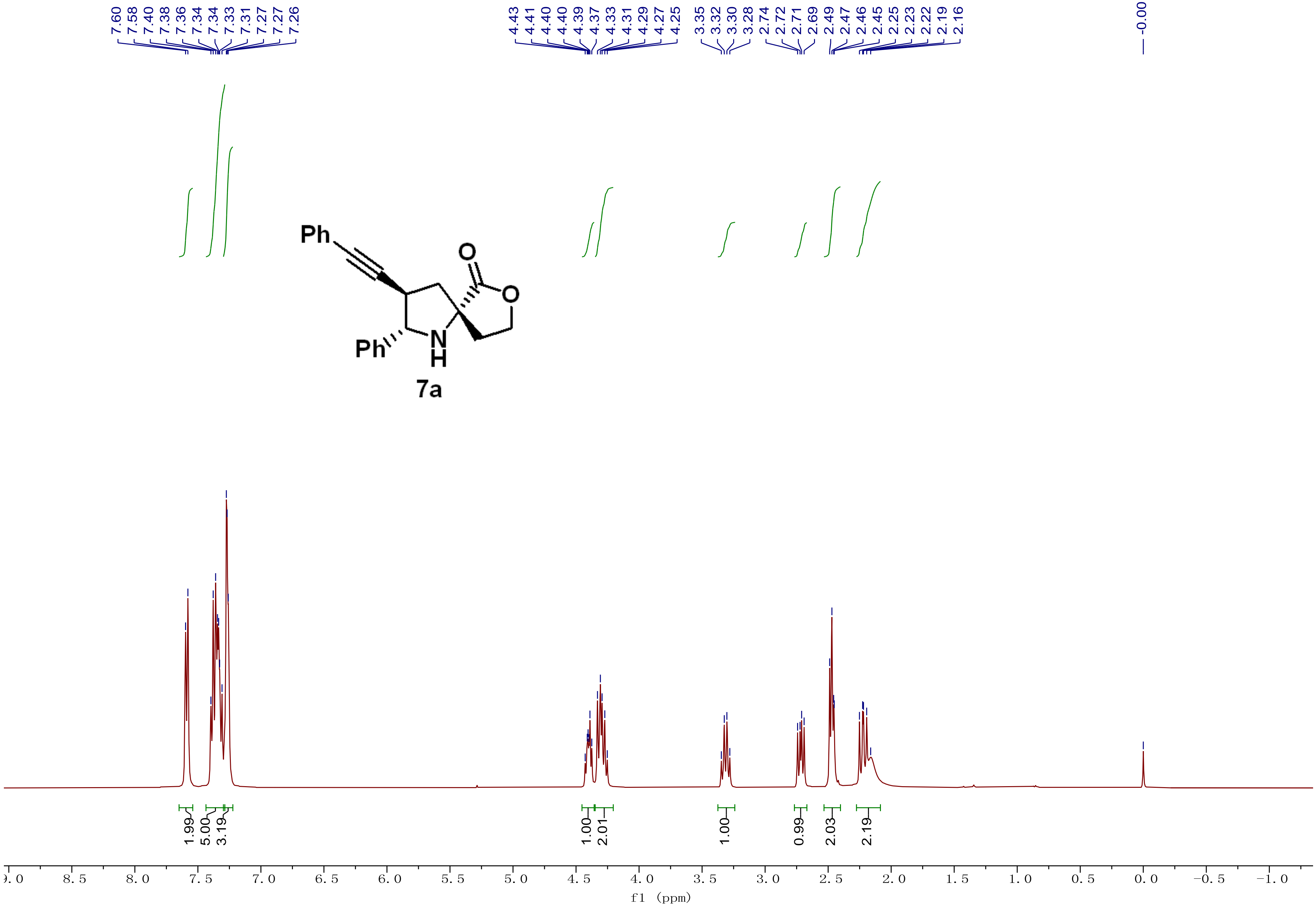
7.60 7.58 7.40 7.38 7.36 7.34 7.34 7.33 7.31 7.27 7.27 7.26
4.43 4.41 4.40 4.40 4.39 4.37 4.33 4.31 4.29 4.27 4.25
3.35 3.32 3.30 3.28
2.74 2.72 2.71 2.69
2.49 2.47 2.46 2.45
2.25 2.23 2.22 2.19 2.16
-0.00
Ph
O
O
Ph
N
H
7a
1.99 5.00 3.19 1.00 2.01 1.00 0.99 2.03 2.19
9.0 8.5 8.0 7.5 7.0 6.5 6.0 5.5 5.0 4.5 4.0 3.5 3.0 2.5 2.0 1.5 1.0 0.5 0.0 −0.5 −1.0
f1 (ppm)

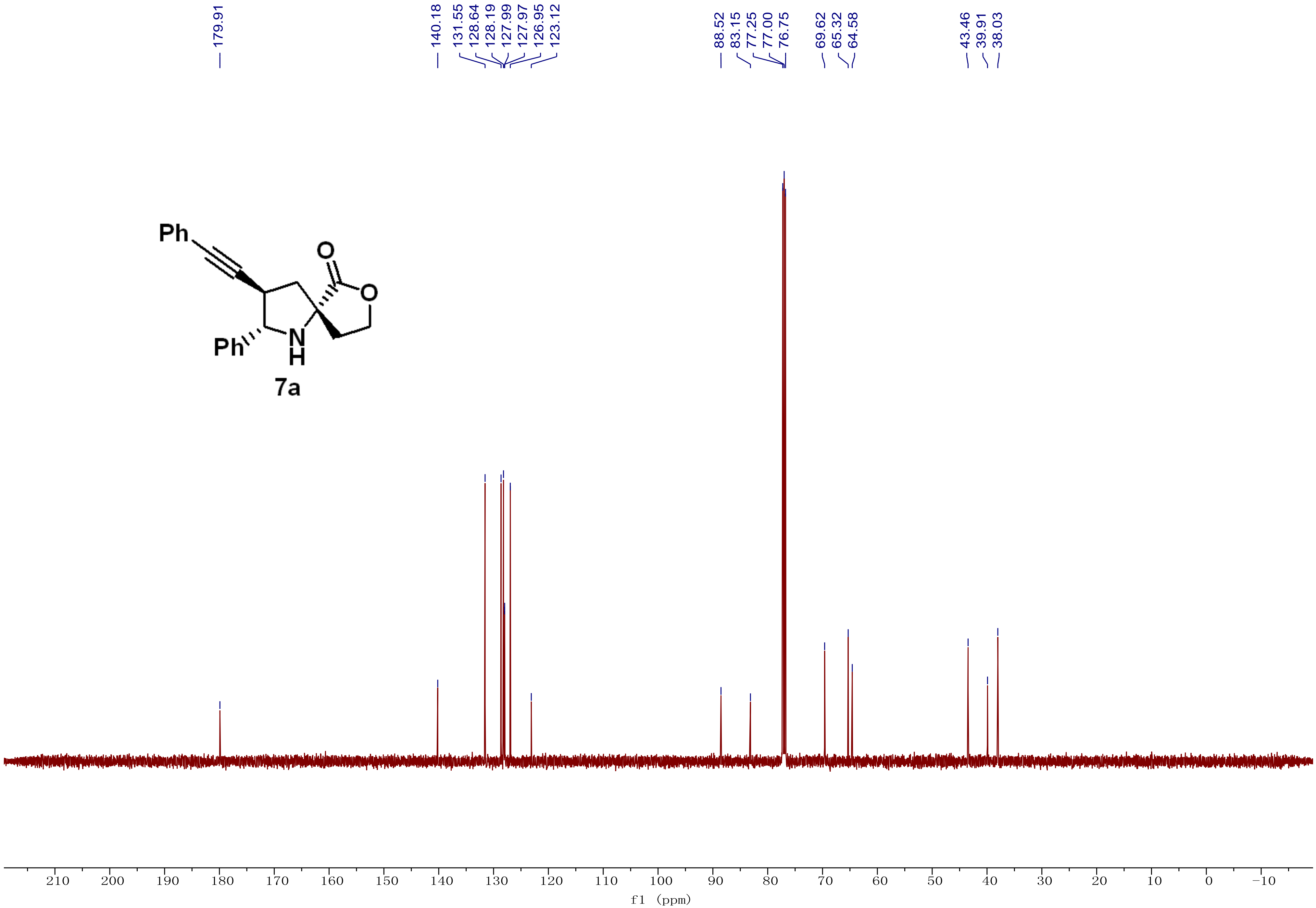
179.91
140.18
131.55
128.64
128.19
127.99
127.97
126.95
123.12
88.52
83.15
77.25
77.00
76.75
69.62
65.32
64.58
43.46
39.91
38.03
Ph
O
O
Ph
N
H
7a
210 200 190 180 170 160 150 140 130 120 110 100 90 80 70 60 50 40 30 20 10 0 −10
f1 (ppm)

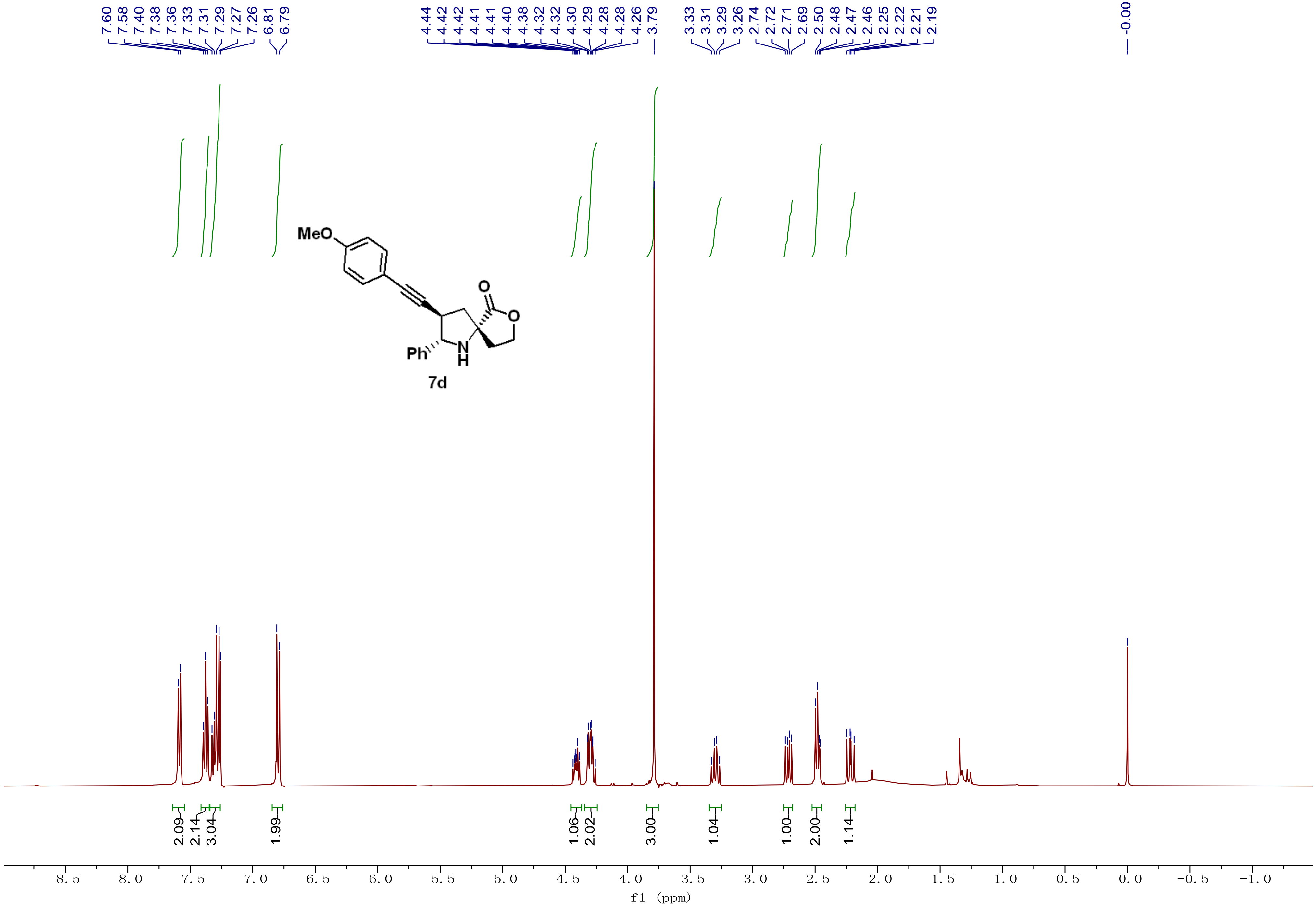
7.60
7.58
7.40
7.38
7.36
7.33
7.31
7.29
7.27
7.26
6.81
6.79
4.44
4.42
4.42
4.41
4.41
4.40
4.38
4.32
4.32
4.30
4.29
4.28
4.28
4.26
3.79
3.33
3.31
3.29
3.26
2.74
2.72
2.71
2.69
2.50
2.48
2.47
2.46
2.25
2.22
2.21
2.19
-0.00
MeO
O
O
Ph
N
H
7d
2.09
2.14
3.04
1.99
1.06
2.02
3.00
1.04
1.00
2.00
1.14
8.5
8.0
7.5
7.0
6.5
6.0
5.5
5.0
4.5
4.0
3.5
3.0
2.5
2.0
1.5
1.0
0.5
0.0
−0.5
−1.0
f1 (ppm)

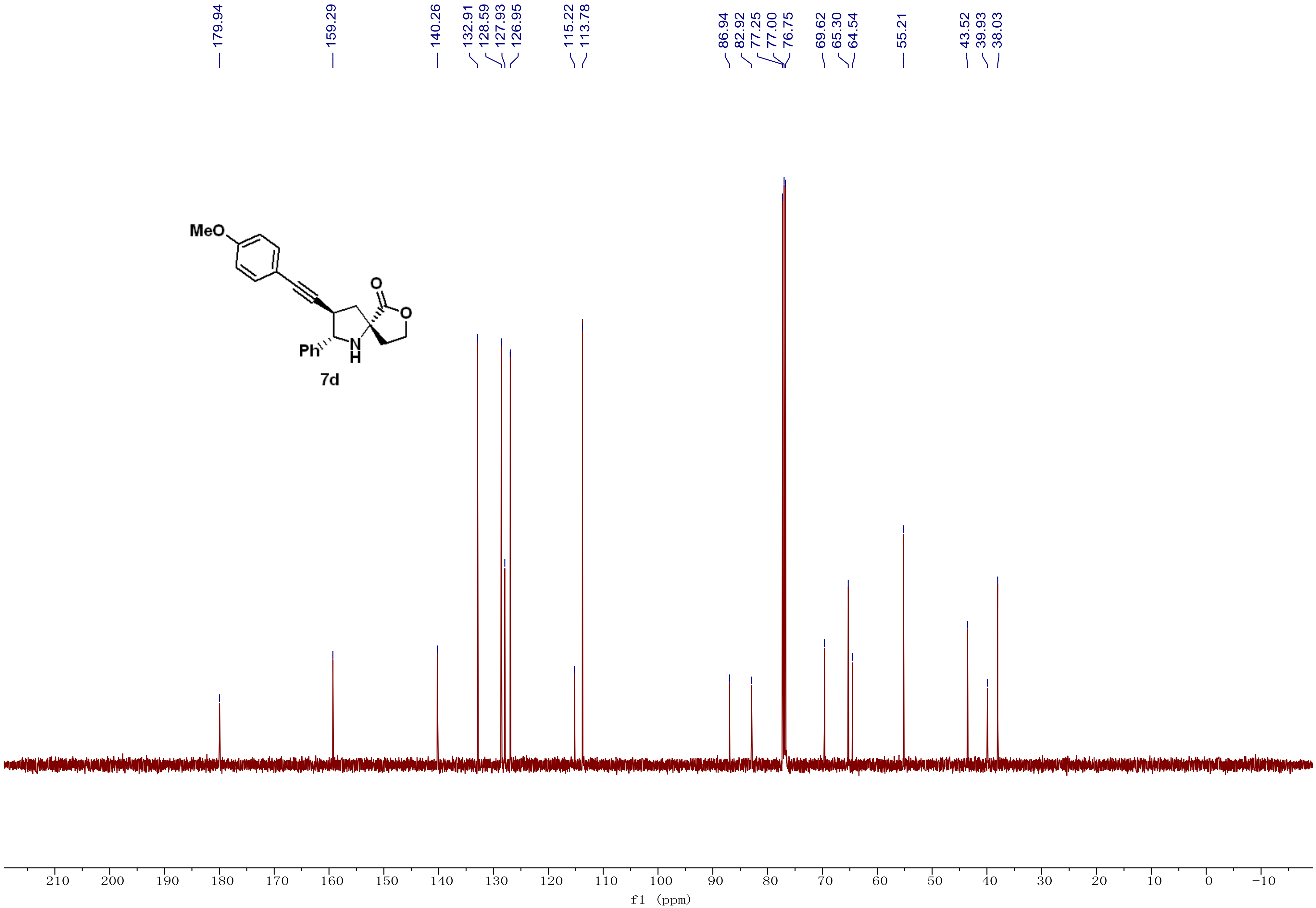
179.94
159.29
140.26
132.91
128.59
127.93
126.95
115.22
113.78
86.94
82.92
77.25
77.00
76.75
69.62
65.30
64.54
55.21
43.52
39.93
38.03
MeO
O
O
Ph
N
H
7d
210 200 190 180 170 160 150 140 130 120 110 100 90 80 70 60 50 40 30 20 10 0 −10
f1 (ppm)

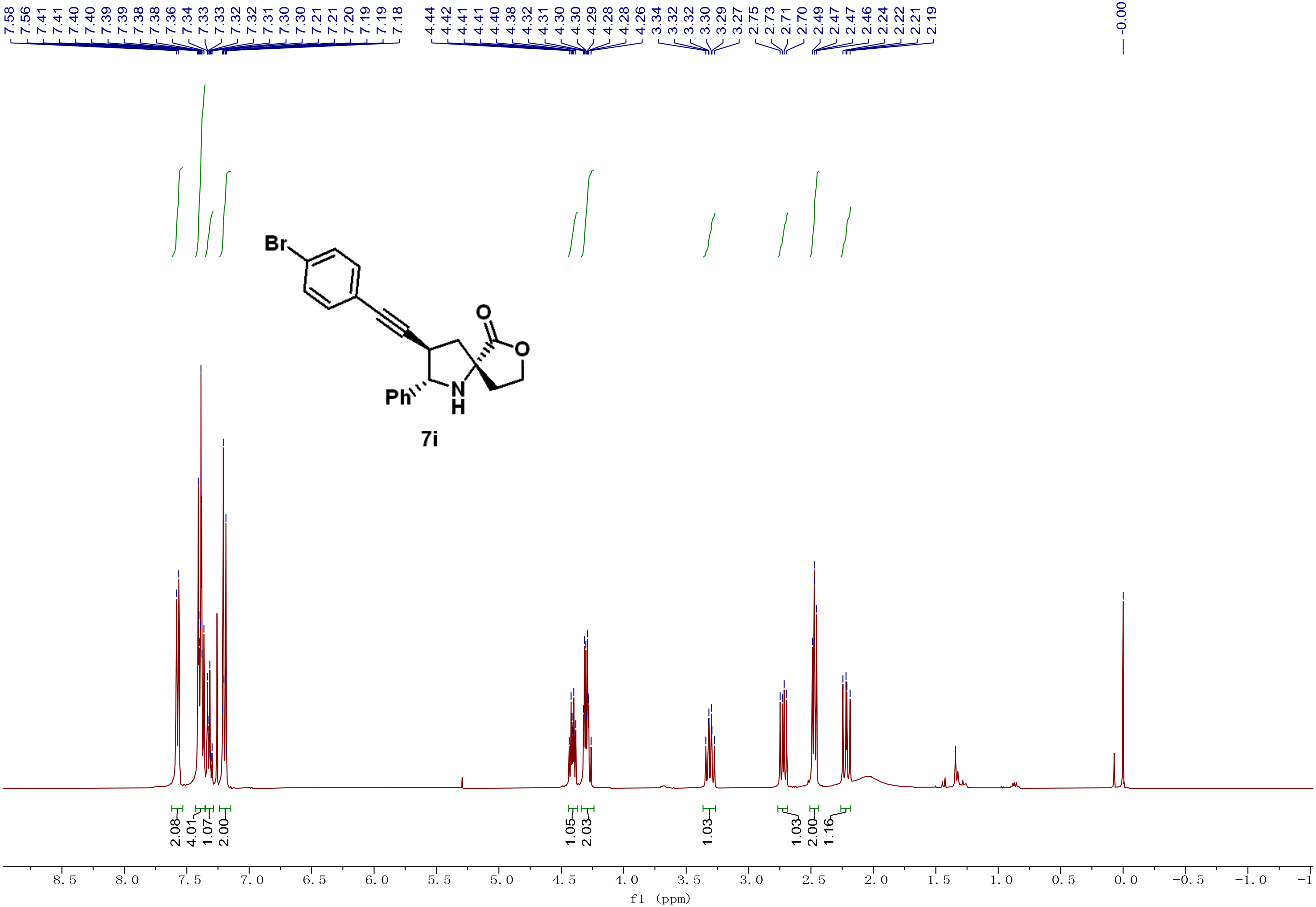
7.58
7.56
7.41
7.41
7.40
7.40
7.39
7.39
7.38
7.38
7.36
7.34
7.33
7.33
7.32
7.32
7.31
7.30
7.30
7.21
7.21
7.20
7.19
7.19
7.18
4.44
4.42
4.41
4.41
4.40
4.38
4.32
4.31
4.30
4.30
4.29
4.28
4.28
4.26
3.34
3.32
3.32
3.30
3.29
3.27
2.75
2.73
2.71
2.70
2.49
2.47
2.47
2.46
2.24
2.22
2.21
2.19
-0.00
Br
O
O
Ph
N
H
7i
2.08
4.01
1.07
2.00
1.05
2.03
1.03
1.03
2.00
1.16
8.5
8.0
7.5
7.0
6.5
6.0
5.5
5.0
4.5
4.0
3.5
3.0
2.5
2.0
1.5
1.0
0.5
0.0
−0.5
−1.0
−1
f1 (ppm)

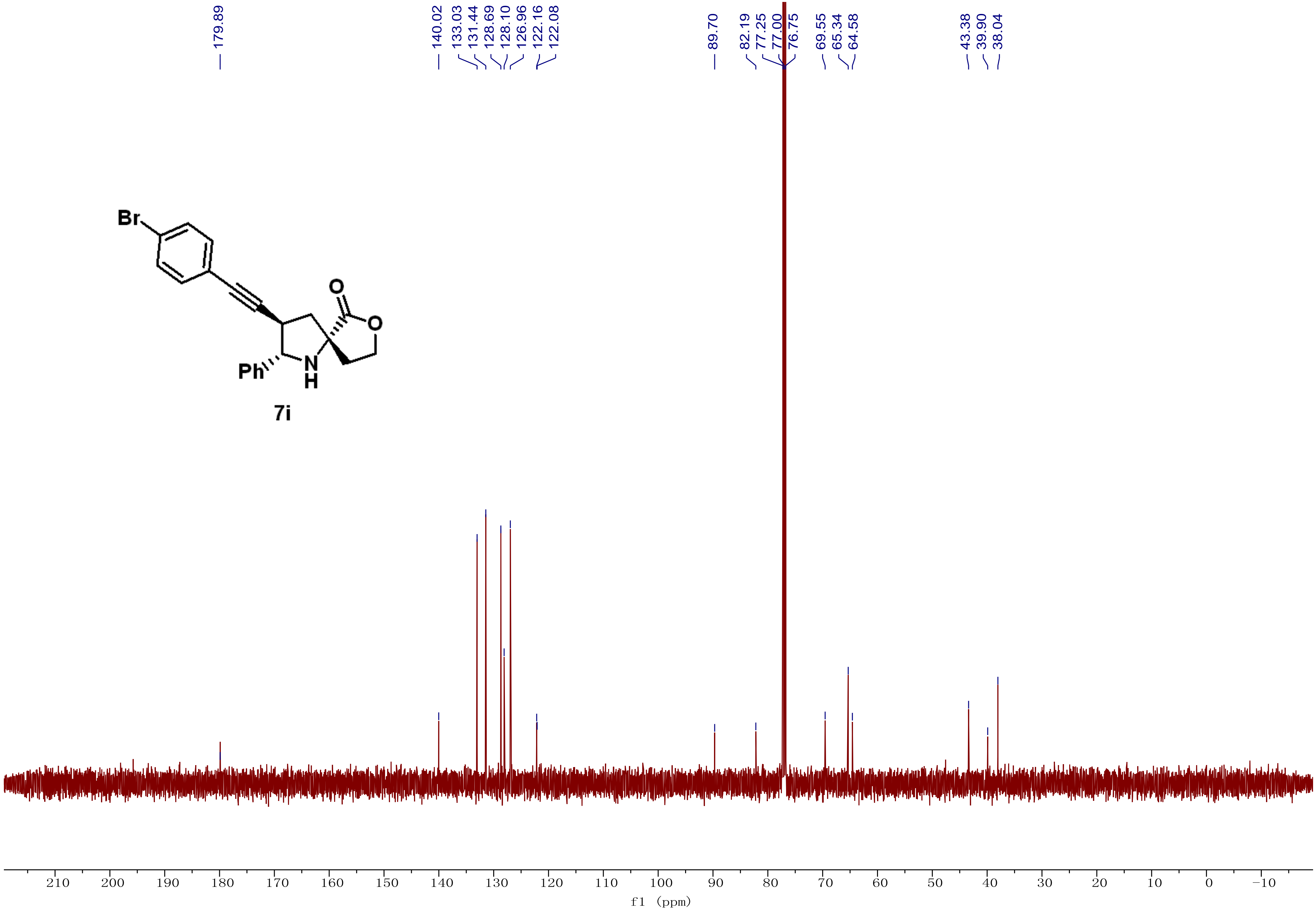
Br
O
O
Ph
N
H
179.89
140.02
133.03
131.44
128.69
128.10
126.96
122.16
122.08
89.70
82.19
77.25
77.00
76.75
69.55
65.34
64.58
43.38
39.90
38.04
210 200 190 180 170 160 150 140 130 120 110 100 90 80 70 60 50 40 30 20 10 0 −10
f1 (ppm)

7i

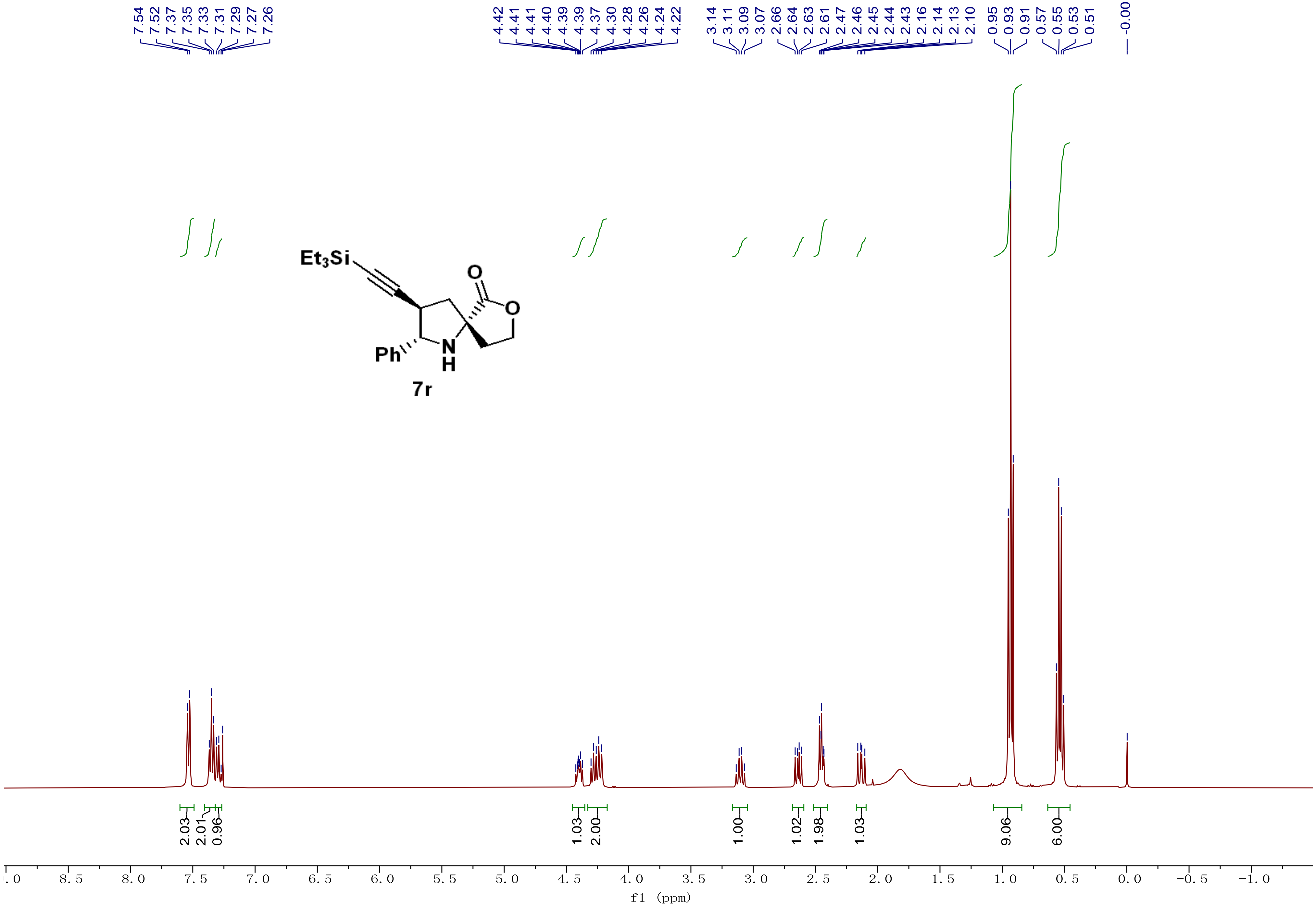
7.54 7.52 7.37 7.35 7.33 7.31 7.29 7.27 7.26
4.42 4.41 4.41 4.40 4.39 4.39 4.37 4.30 4.28 4.26 4.24 4.22
3.14 3.11 3.09 3.07 2.66 2.64 2.63 2.61 2.47 2.46 2.45 2.44 2.43 2.16 2.14 2.13 2.10
0.95 0.93 0.91 0.57 0.55 0.53 0.51
-0.00
Et3Si
O
O
Ph
N
H
7r
2.03 2.01 0.96 1.03 2.00 1.00 1.02 1.98 1.03 9.06 6.00
f1 (ppm)

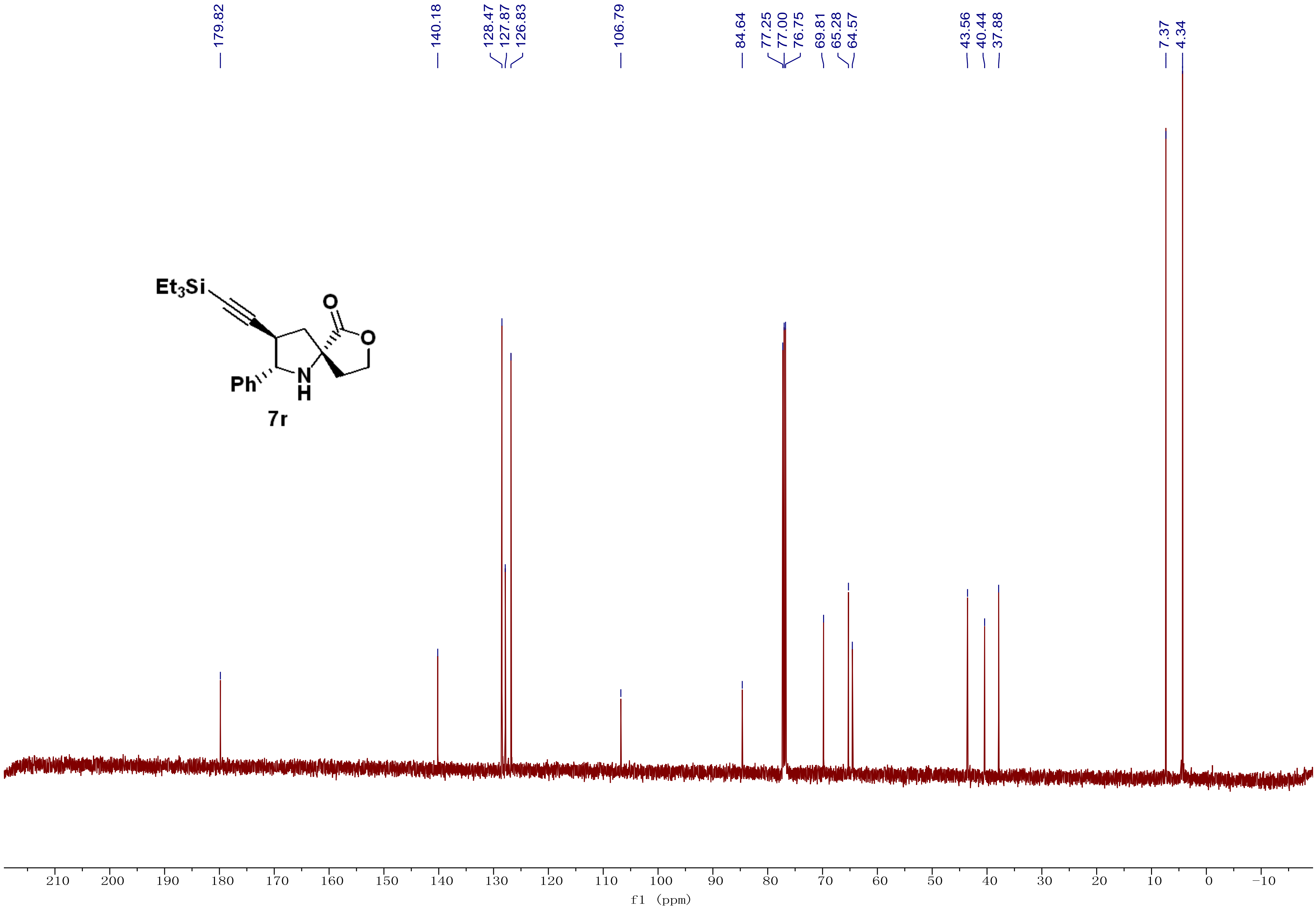
179.82
140.18
128.47
127.87
126.83
106.79
84.64
77.25
77.00
76.75
69.81
65.28
64.57
43.56
40.44
37.88
7.37
4.34
Et3Si
O
O
Ph
N
H
7r
210 200 190 180 170 160 150 140 130 120 110 100 90 80 70 60 50 40 30 20 10 0 -10
f1 (ppm)

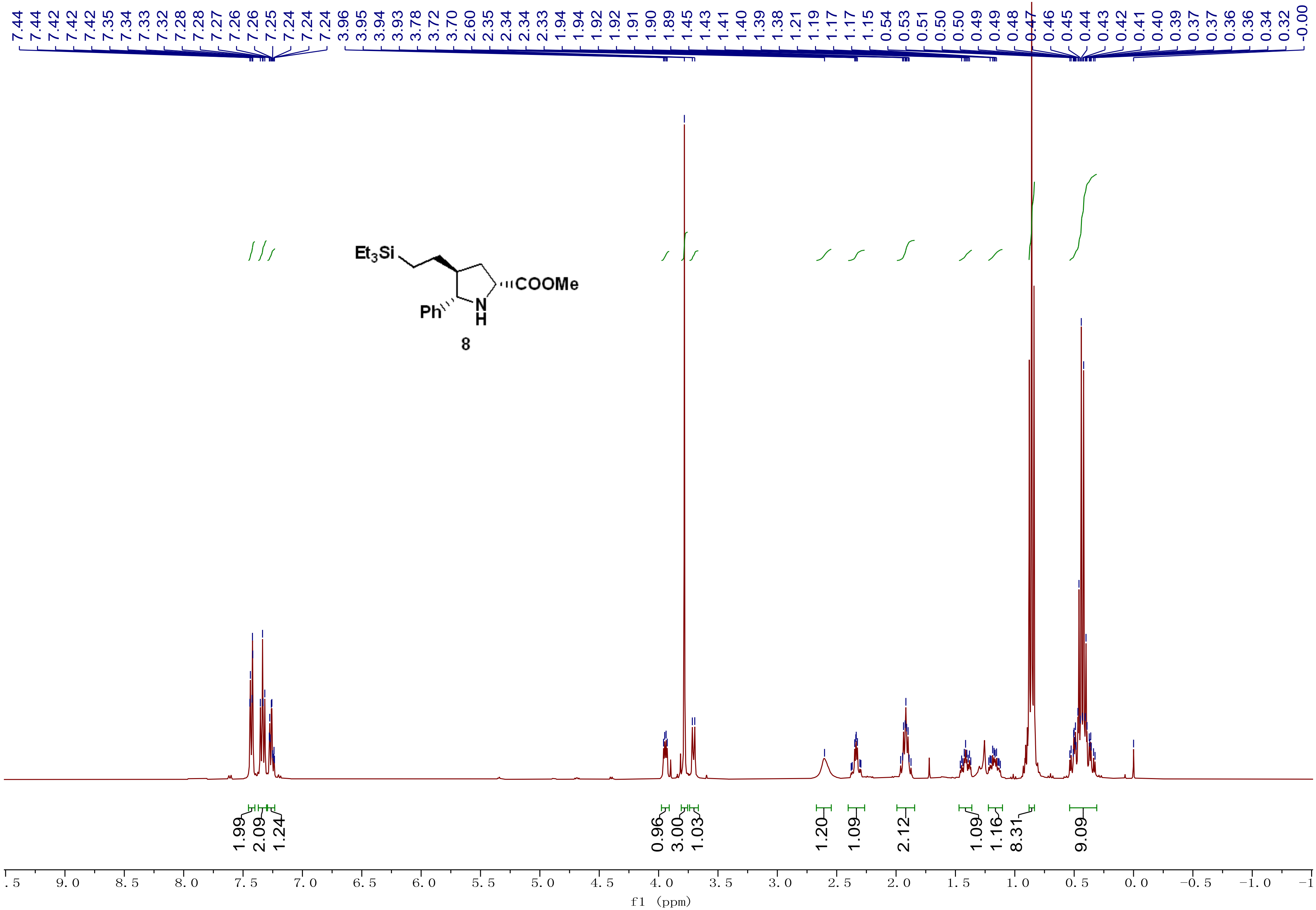
7.44 7.44 7.42 7.42 7.42 7.35 7.34 7.33 7.32 7.28 7.28 7.27 7.26 7.26 7.25 7.24 7.24 7.24
3.96 3.95 3.94 3.93 3.78 3.72 3.70 2.60 2.35 2.34 2.34 2.33 1.94 1.94 1.92 1.92 1.91 1.90 1.89 1.45 1.43 1.41 1.40 1.39 1.38 1.21 1.19 1.17 1.17 1.15 0.54 0.53 0.51 0.50 0.50 0.49 0.49 0.48 0.47 0.46 0.45 0.44 0.43 0.42 0.41 0.40 0.39 0.37 0.37 0.36 0.36 0.34 0.32 -0.00
Et3Si
COOMe
Ph
N
H
8
1.99 2.09 1.24
0.96 3.00 1.03
1.20 1.09 2.12 1.09 1.16 8.31 9.09
f1 (ppm)

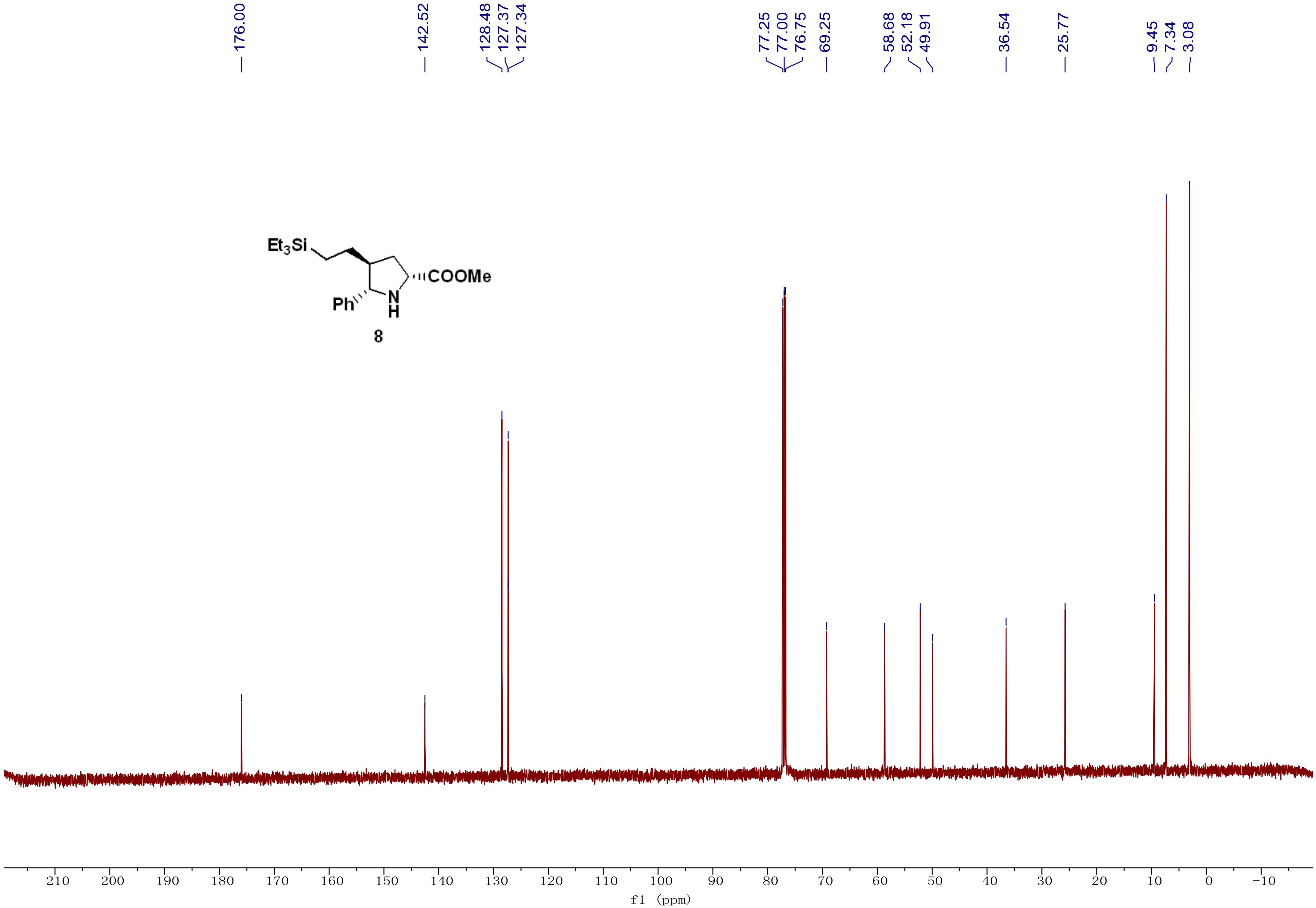
176.00
142.52
128.48
127.37
127.34
77.25
77.00
76.75
69.25
58.68
52.18
49.91
36.54
25.77
9.45
7.34
3.08
Et3Si
COOMe
Ph
N
H
8
210 200 190 180 170 160 150 140 130 120 110 100 90 80 70 60 50 40 30 20 10 0 −10
f1 (ppm)

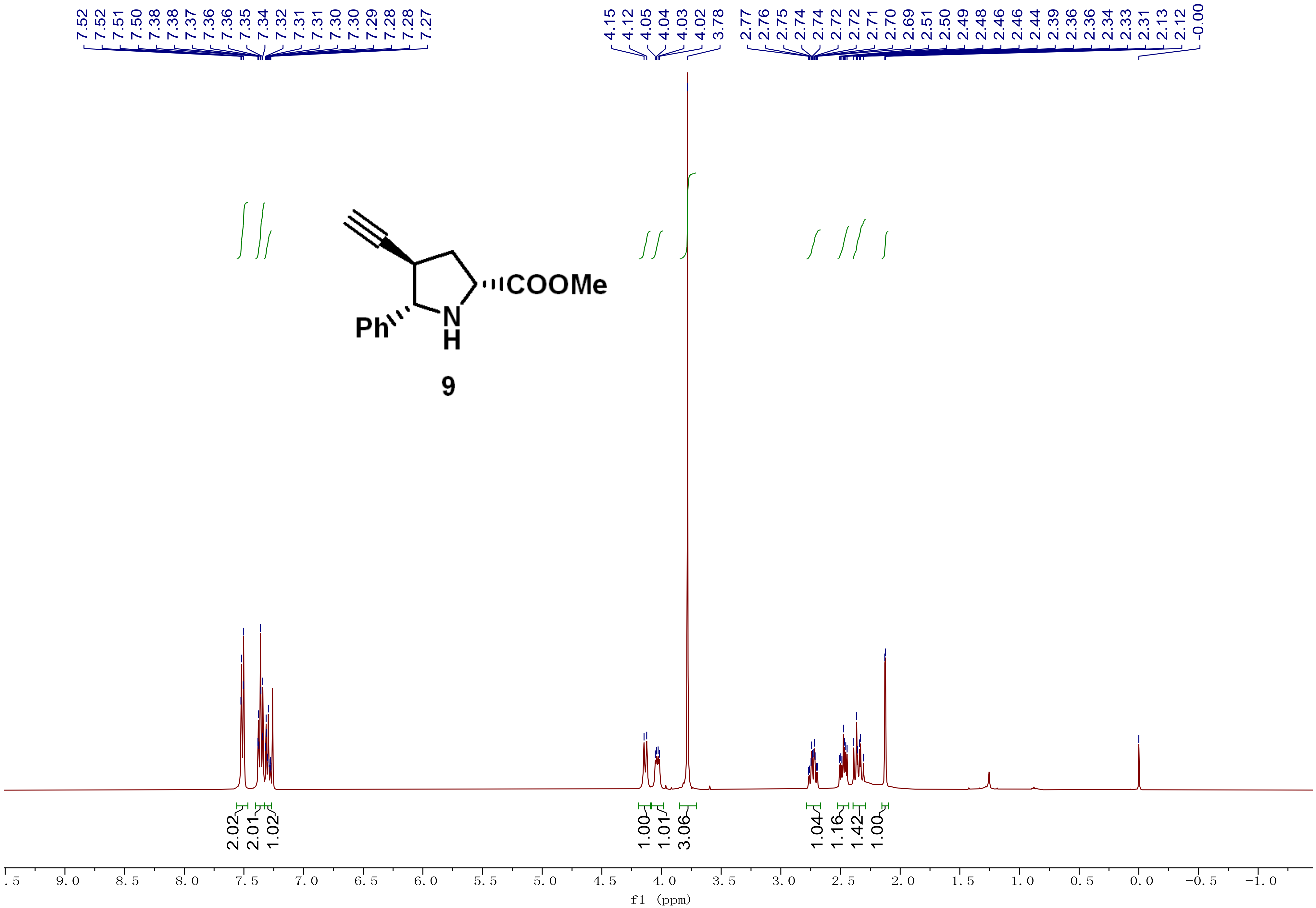
7.52 7.52 7.51 7.50 7.38 7.38 7.37 7.36 7.36 7.35 7.34 7.32 7.31 7.31 7.30 7.30 7.29 7.28 7.28 7.27
4.15 4.12 4.05 4.04 4.03 4.02 3.78
2.77 2.76 2.75 2.74 2.74 2.72 2.72 2.71 2.70 2.69 2.51 2.50 2.49 2.48 2.46 2.46 2.44 2.39 2.36 2.36 2.34 2.33 2.31 2.13 2.12 -0.00
COOMe
Ph
N
H
9
2.02 2.01 1.02
1.00 1.01 3.06
1.04 1.16 1.42 1.00
.5 9.0 8.5 8.0 7.5 7.0 6.5 6.0 5.5 5.0 4.5 4.0 3.5 3.0 2.5 2.0 1.5 1.0 0.5 0.0 -0.5 -1.0
f1 (ppm)

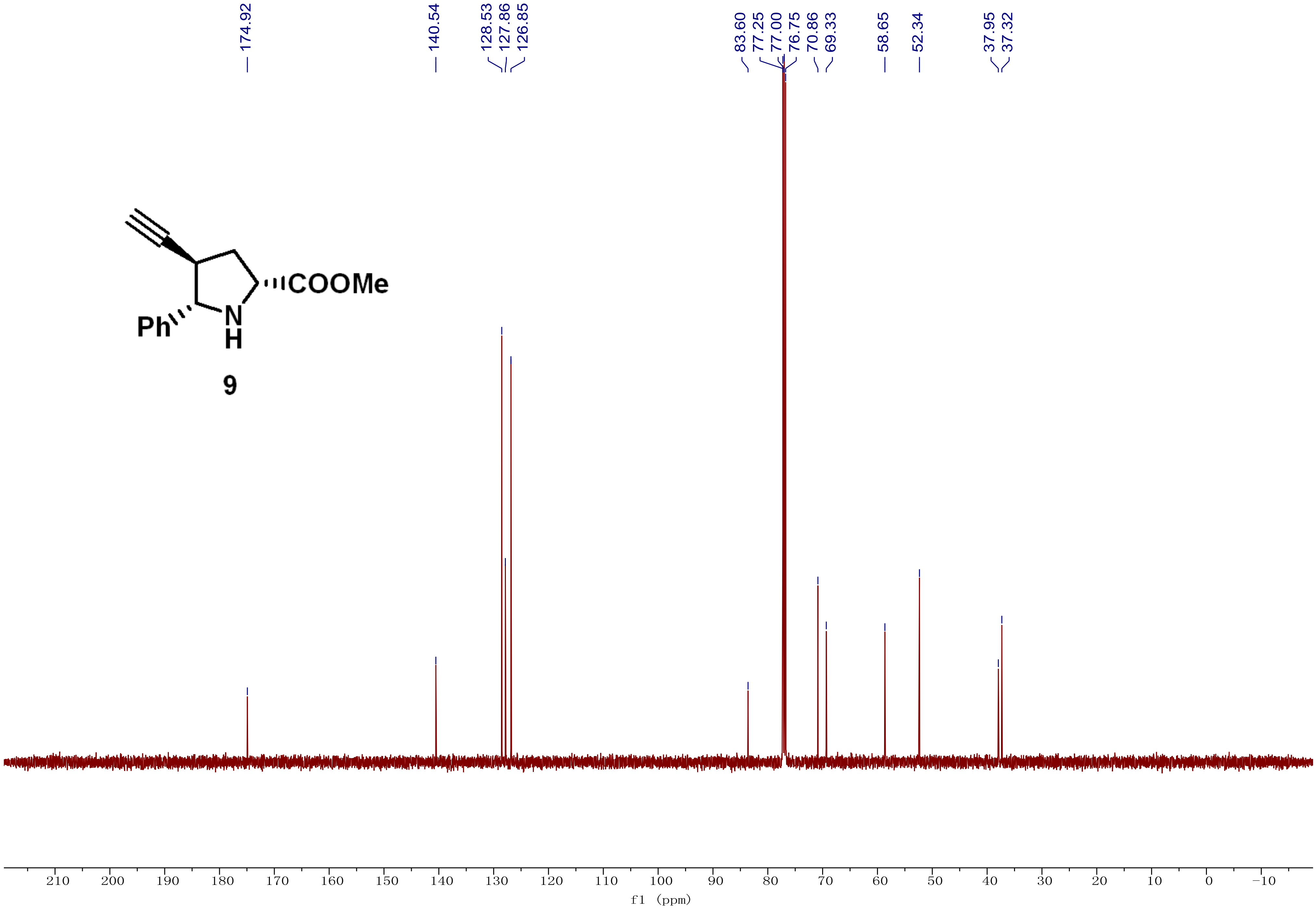
174.92
140.54
128.53
127.86
126.85
83.60
77.25
77.00
76.75
70.86
69.33
58.65
52.34
37.95
37.32
COOMe
Ph
N
H
9
210 200 190 180 170 160 150 140 130 120 110 100 90 80 70 60 50 40 30 20 10 0 −10
f1 (ppm)

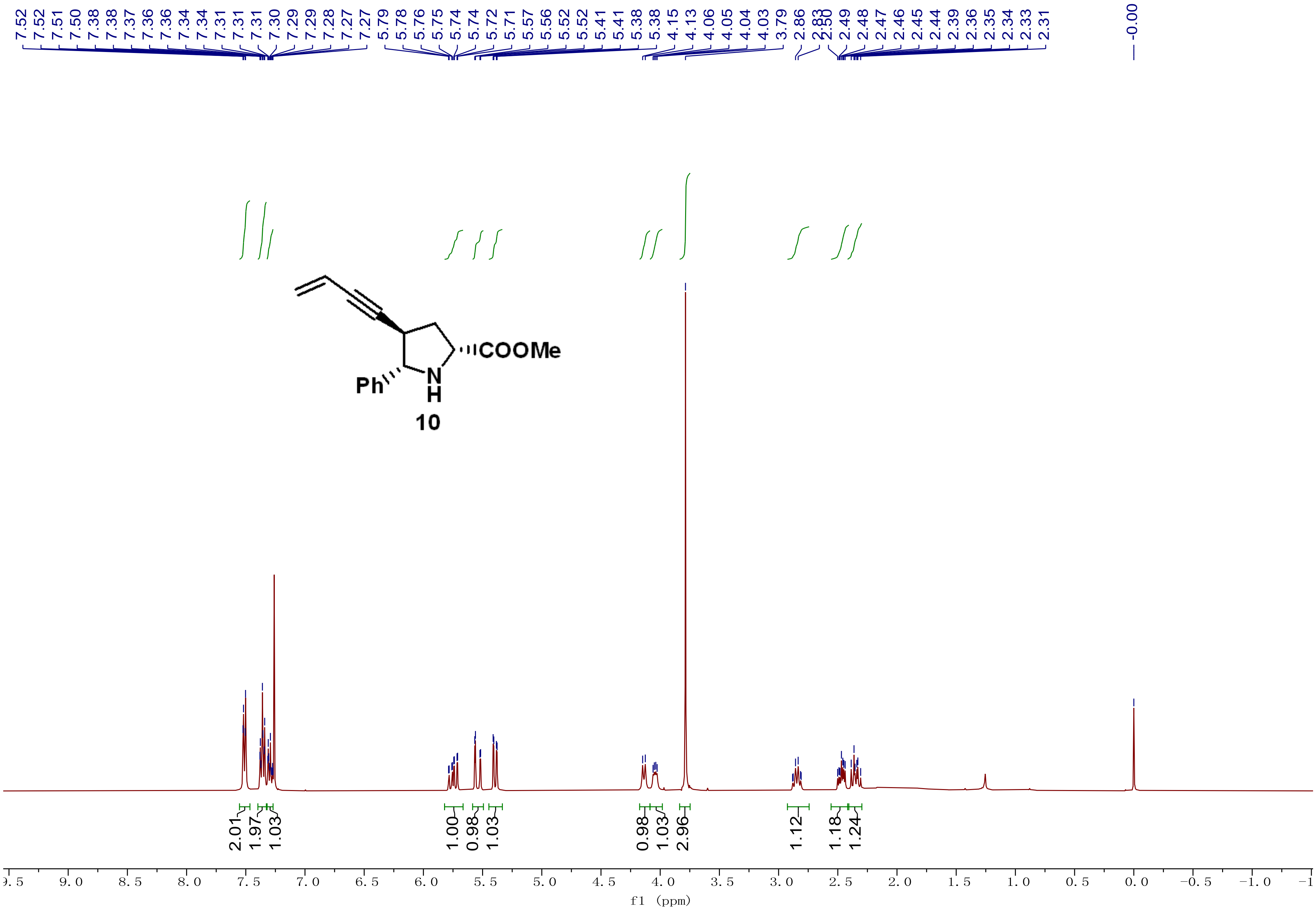
7.52 7.52 7.51 7.50 7.38 7.38 7.37 7.36 7.36 7.34 7.34 7.31 7.31 7.31 7.30 7.29 7.29 7.28 7.27 7.27 5.79 5.78 5.76 5.75 5.74 5.74 5.72 5.71 5.57 5.56 5.52 5.52 5.41 5.41 5.38 5.38 4.15 4.13 4.06 4.05 4.04 4.03 3.79 2.86 2.83 2.50 2.49 2.48 2.47 2.46 2.45 2.44 2.39 2.36 2.35 2.34 2.33 2.31
-0.00
COOMe
Ph
N
H
10
2.01 1.97 1.03 1.00 0.98 1.03 0.98 1.03 2.96 1.12 1.18 1.24
9.5 9.0 8.5 8.0 7.5 7.0 6.5 6.0 5.5 5.0 4.5 4.0 3.5 3.0 2.5 2.0 1.5 1.0 0.5 0.0 -0.5 -1.0 -1
f1 (ppm)

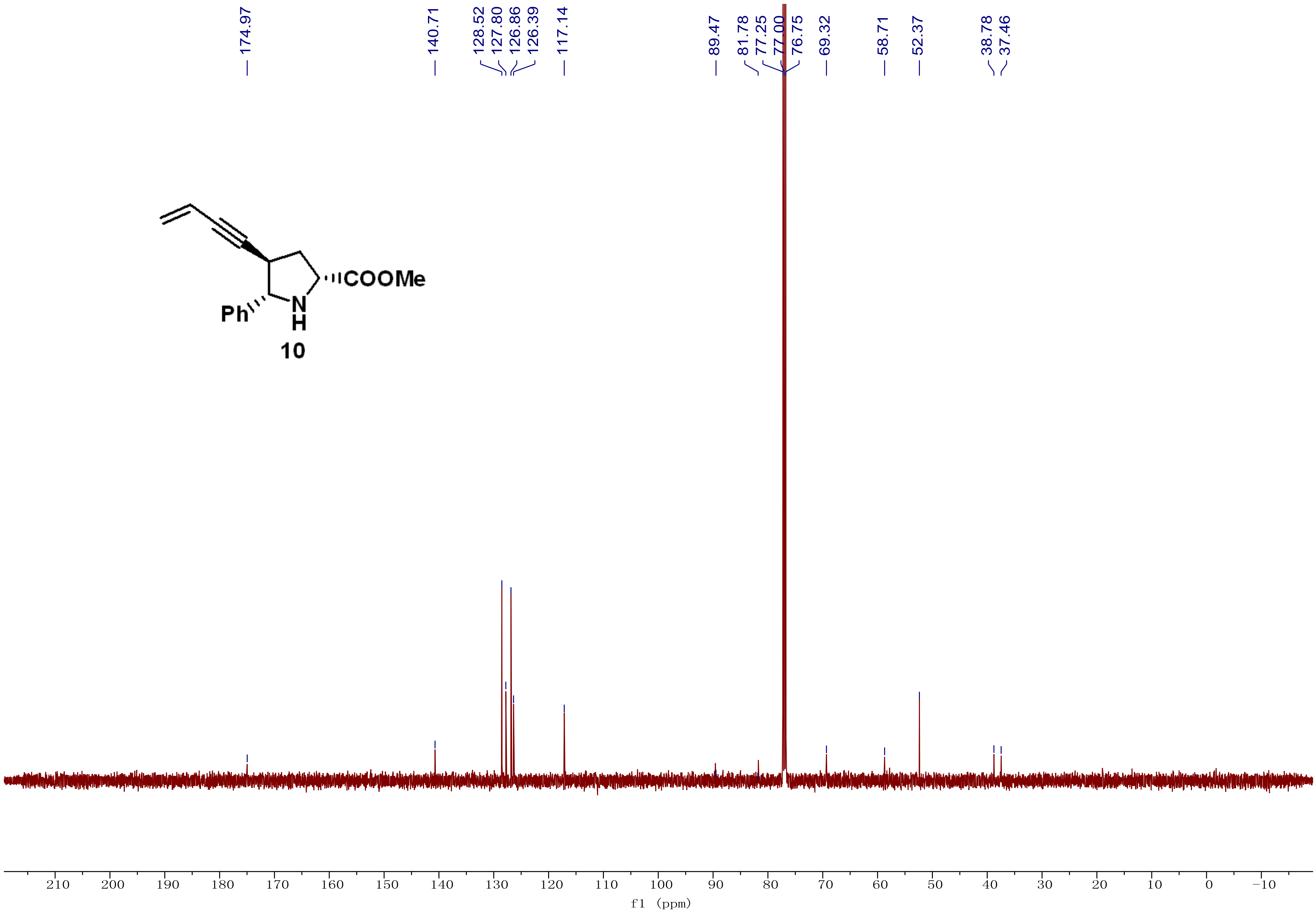

174.97
140.71
128.52
127.80
126.86
126.39
117.14
89.47
81.78
77.25
77.00
76.75
69.32
58.71
52.37
38.78
37.46
COOMe
Ph
N
H
10
210 200 190 180 170 160 150 140 130 120 110 100 90 80 70 60 50 40 30 20 10 0 −10
f1 (ppm)

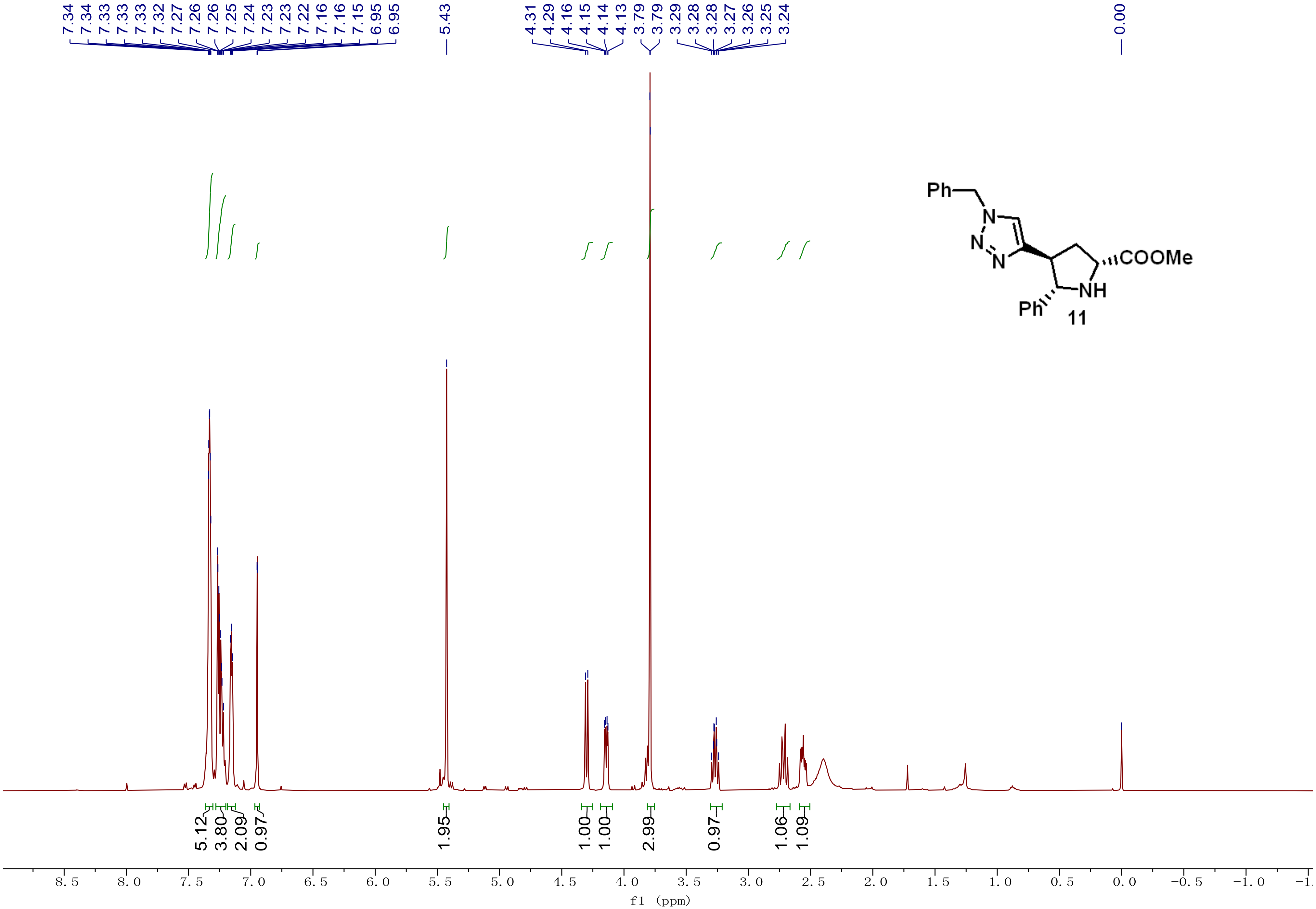
7.34
7.34
7.33
7.33
7.33
7.32
7.27
7.26
7.26
7.25
7.24
7.23
7.23
7.22
7.16
7.16
7.15
6.95
6.95
5.43
4.31
4.29
4.16
4.15
4.14
4.13
3.79
3.79
3.29
3.28
3.28
3.27
3.26
3.25
3.24
0.00
Ph
N
N
N
COOMe
NH
Ph
11
5.12
3.80
2.09
0.97
1.95
1.00
1.00
2.99
0.97
1.06
1.09
8.5
8.0
7.5
7.0
6.5
6.0
5.5
5.0
4.5
4.0
3.5
3.0
2.5
2.0
1.5
1.0
0.5
0.0
−0.5
−1.0
f1 (ppm)

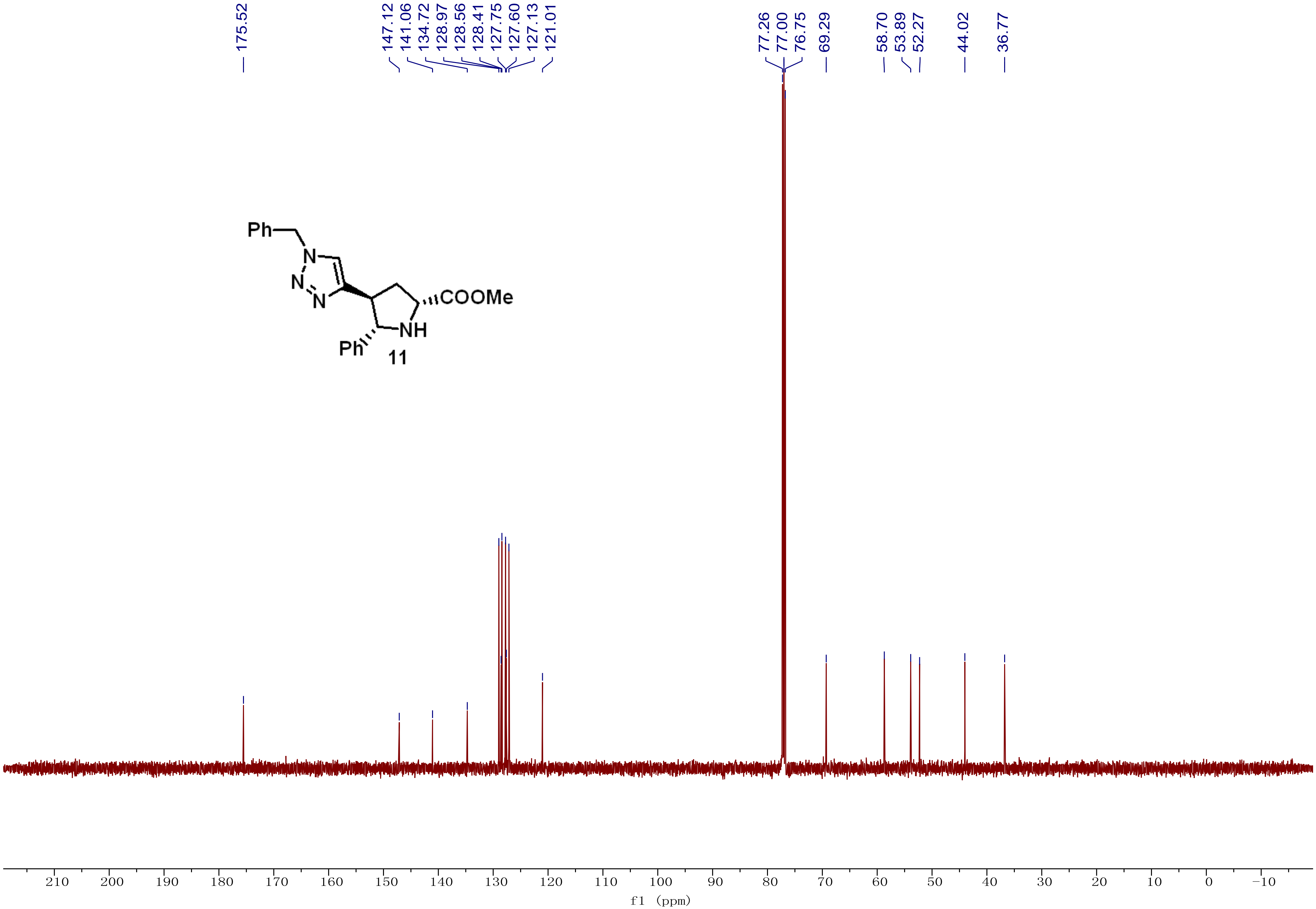

175.52
147.12
141.06
134.72
128.97
128.56
128.41
127.75
127.60
127.13
121.01
77.26
77.00
76.75
69.29
58.70
53.89
52.27
44.02
36.77
Ph
N
N
N
COOMe
NH
Ph
11
210 200 190 180 170 160 150 140 130 120 110 100 90 80 70 60 50 40 30 20 10 0 −10
f1 (ppm)

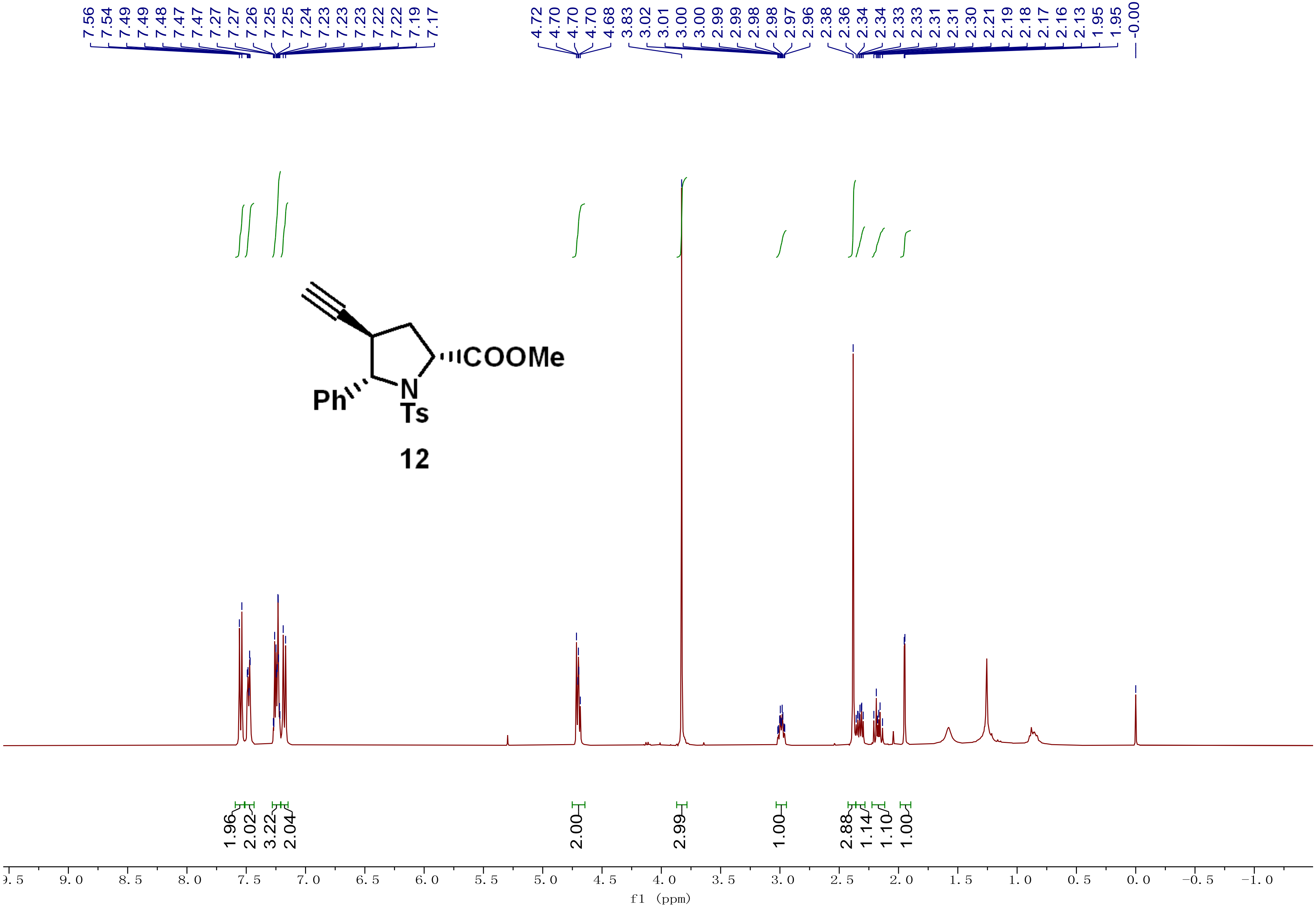
7.56 7.54 7.49 7.49 7.48 7.47 7.47 7.27 7.27 7.26 7.25 7.25 7.24 7.23 7.23 7.23 7.22 7.22 7.19 7.17
4.72 4.70 4.70 4.70 4.68 3.83 3.02 3.01 3.00 3.00 2.99 2.99 2.98 2.98 2.97 2.96 2.38 2.36 2.34 2.34 2.33 2.33 2.31 2.31 2.30 2.21 2.19 2.18 2.17 2.16 2.13 1.95 1.95
-0.00
COOMe
Ph
N
Ts
12
1.96 2.02 3.22 2.04 2.00 2.99 1.00 2.88 1.14 1.10 1.00
9.5 9.0 8.5 8.0 7.5 7.0 6.5 6.0 5.5 5.0 4.5 4.0 3.5 3.0 2.5 2.0 1.5 1.0 0.5 0.0 -0.5 -1.0
f1 (ppm)

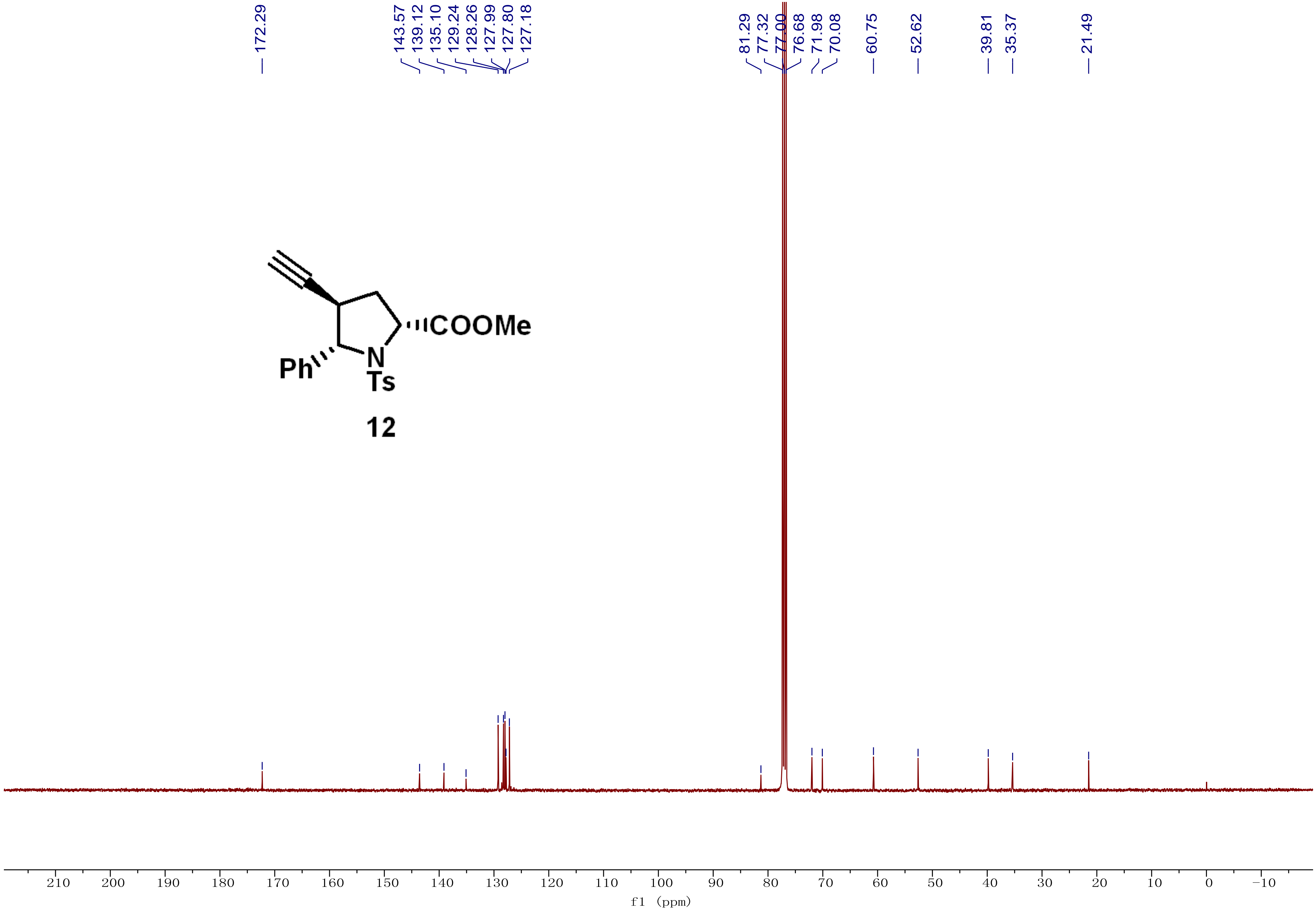
172.29
143.57
139.12
135.10
129.24
128.26
127.99
127.80
127.18
81.29
77.32
77.00
76.68
71.98
70.08
60.75
52.62
39.81
35.37
21.49
COOMe
Ph
N
Ts
12
210 200 190 180 170 160 150 140 130 120 110 100 90 80 70 60 50 40 30 20 10 0 −10
f1 (ppm)

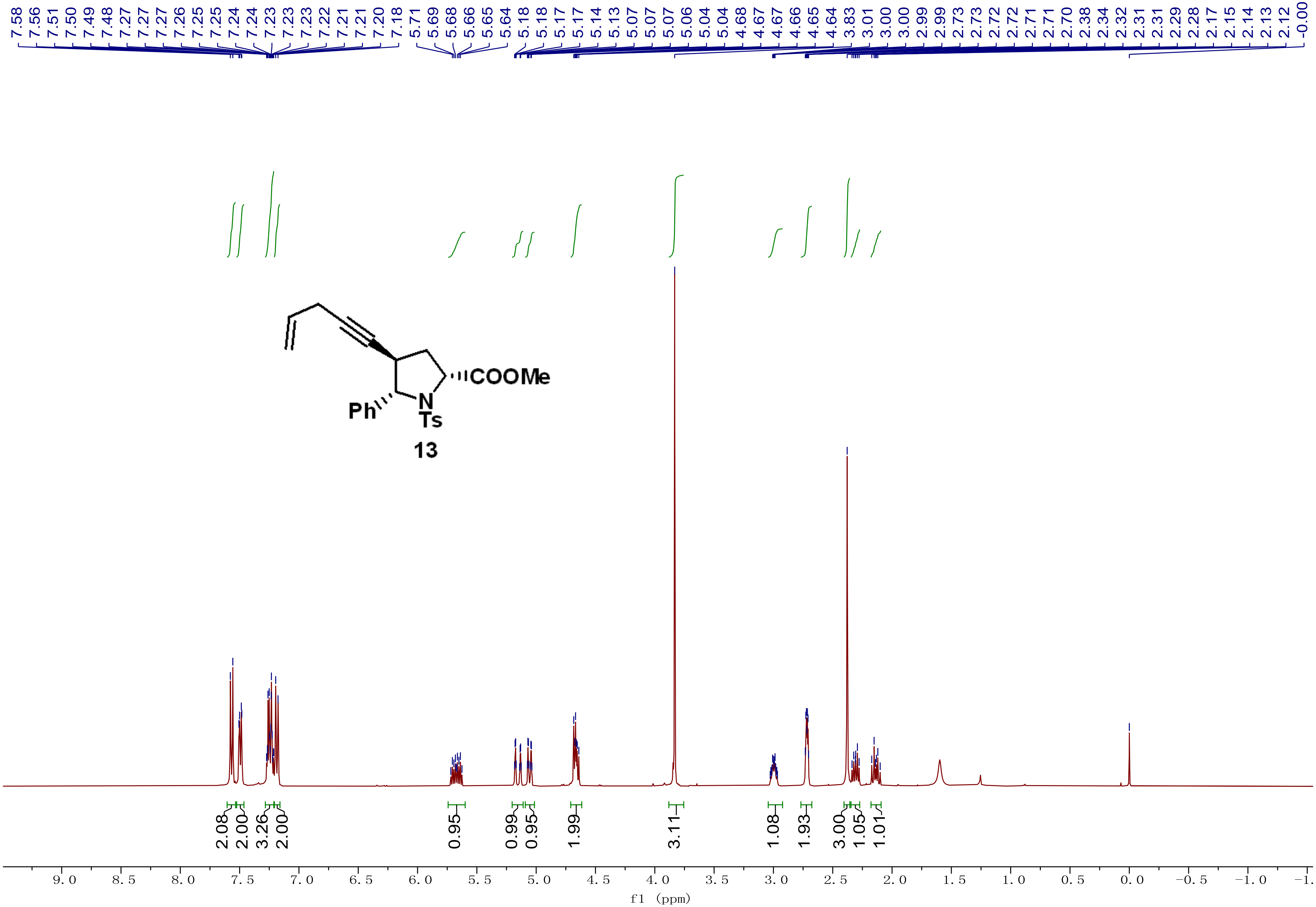
7.58 7.56 7.51 7.50 7.49 7.48 7.27 7.27 7.27 7.26 7.25 7.25 7.24 7.24 7.23 7.23 7.23 7.22 7.21 7.21 7.20 7.18 5.71 5.69 5.68 5.66 5.65 5.64 5.18 5.18 5.17 5.17 5.14 5.13 5.07 5.07 5.07 5.06 5.04 5.04 4.68 4.67 4.67 4.66 4.65 4.64 3.83 3.01 3.00 3.00 2.99 2.99 2.73 2.73 2.72 2.72 2.71 2.71 2.70 2.38 2.34 2.32 2.31 2.31 2.29 2.28 2.17 2.15 2.14 2.13 2.12 -0.00
COOMe
Ph
N
Ts
13
2.08 2.00 3.26 2.00 0.95 0.99 0.95 1.99 3.11 1.08 1.93 3.00 1.05 1.01
9.0 8.5 8.0 7.5 7.0 6.5 6.0 5.5 5.0 4.5 4.0 3.5 3.0 2.5 2.0 1.5 1.0 0.5 0.0 −0.5 −1.0 −1.
f1 (ppm)

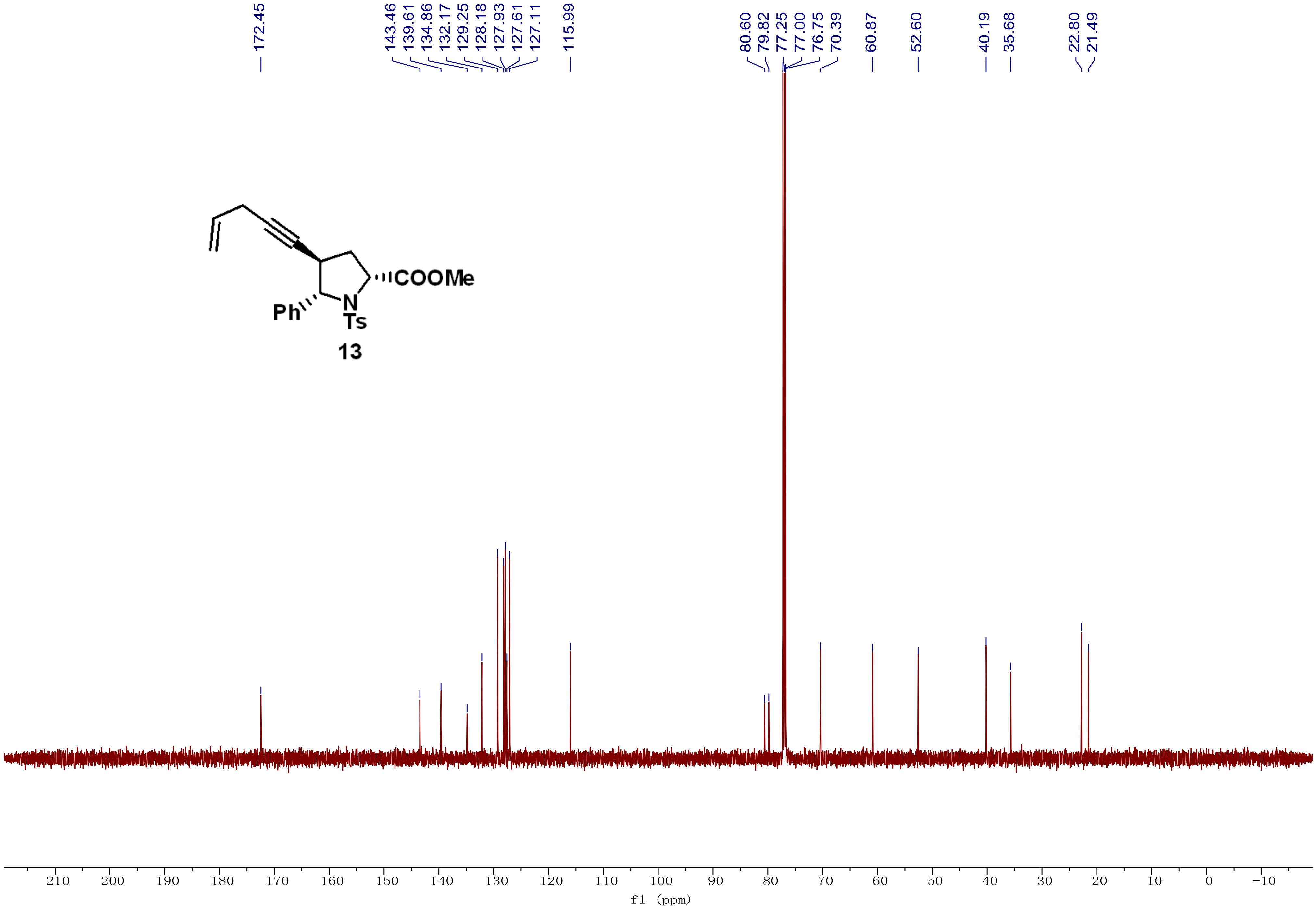
172.45
143.46
139.61
134.86
132.17
129.25
128.18
127.93
127.61
127.11
115.99
80.60
79.82
77.25
77.00
76.75
70.39
60.87
52.60
40.19
35.68
22.80
21.49
COOMe
Ph
N
Ts
13
210 200 190 180 170 160 150 140 130 120 110 100 90 80 70 60 50 40 30 20 10 0 −10
f1 (ppm)

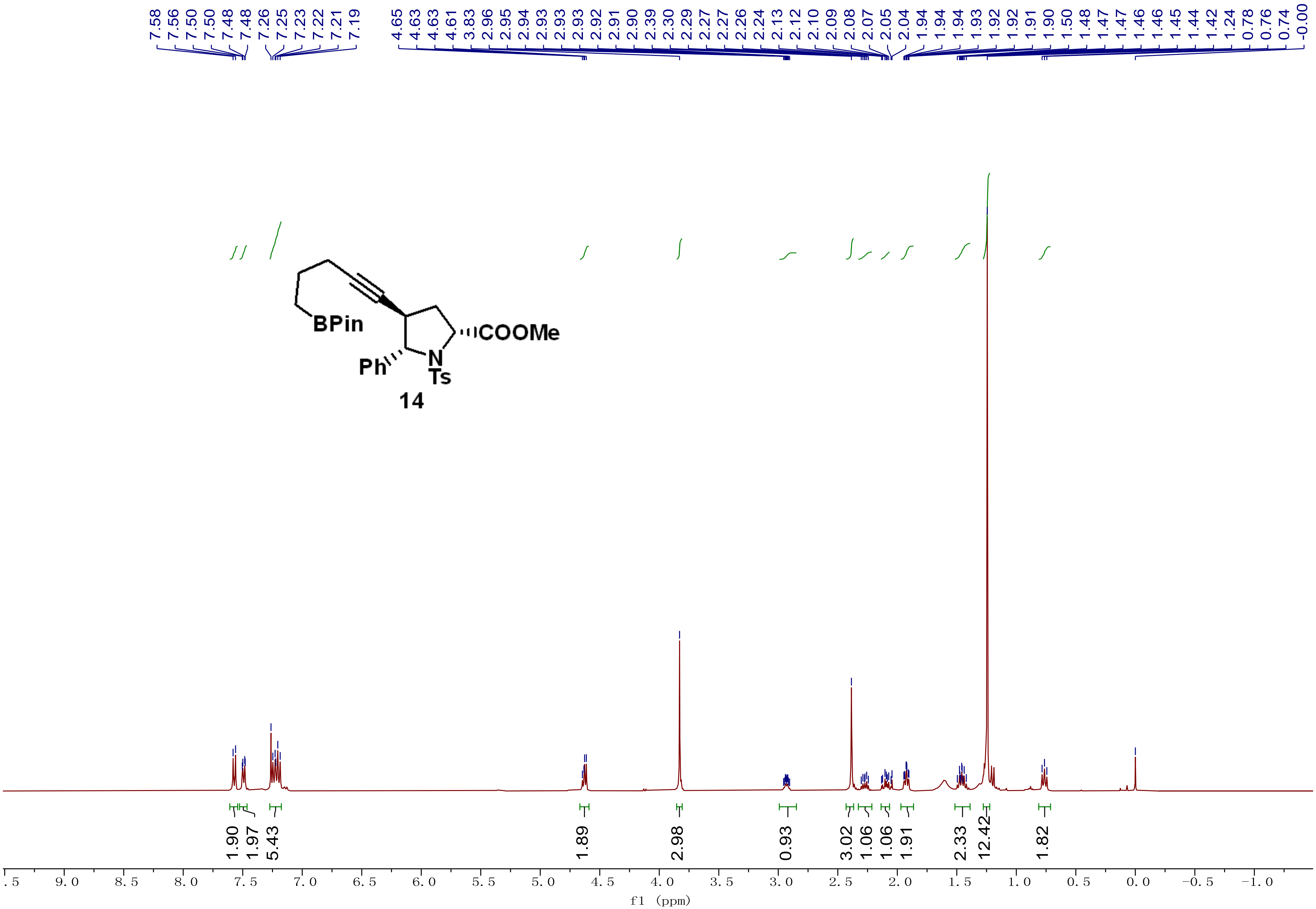
7.58 7.56 7.50 7.50 7.48 7.48 7.26 7.25 7.23 7.22 7.21 7.19
4.65 4.63 4.63 4.61 3.83 2.96 2.95 2.94 2.93 2.93 2.93 2.92 2.91 2.90 2.39 2.30 2.29 2.27 2.27 2.26 2.24 2.13 2.12 2.10 2.09 2.08 2.07 2.05 2.04 1.94 1.94 1.94 1.93 1.92 1.92 1.91 1.90 1.50 1.48 1.47 1.47 1.46 1.46 1.45 1.44 1.42 1.24 0.78 0.76 0.74 -0.00
BPin
COOMe
Ph
N
Ts
14
1.90 1.97 5.43 1.89 2.98 0.93 3.02 1.06 1.06 1.91 2.33 12.42 1.82
.5 9.0 8.5 8.0 7.5 7.0 6.5 6.0 5.5 5.0 4.5 4.0 3.5 3.0 2.5 2.0 1.5 1.0 0.5 0.0 −0.5 −1.0
f1 (ppm)

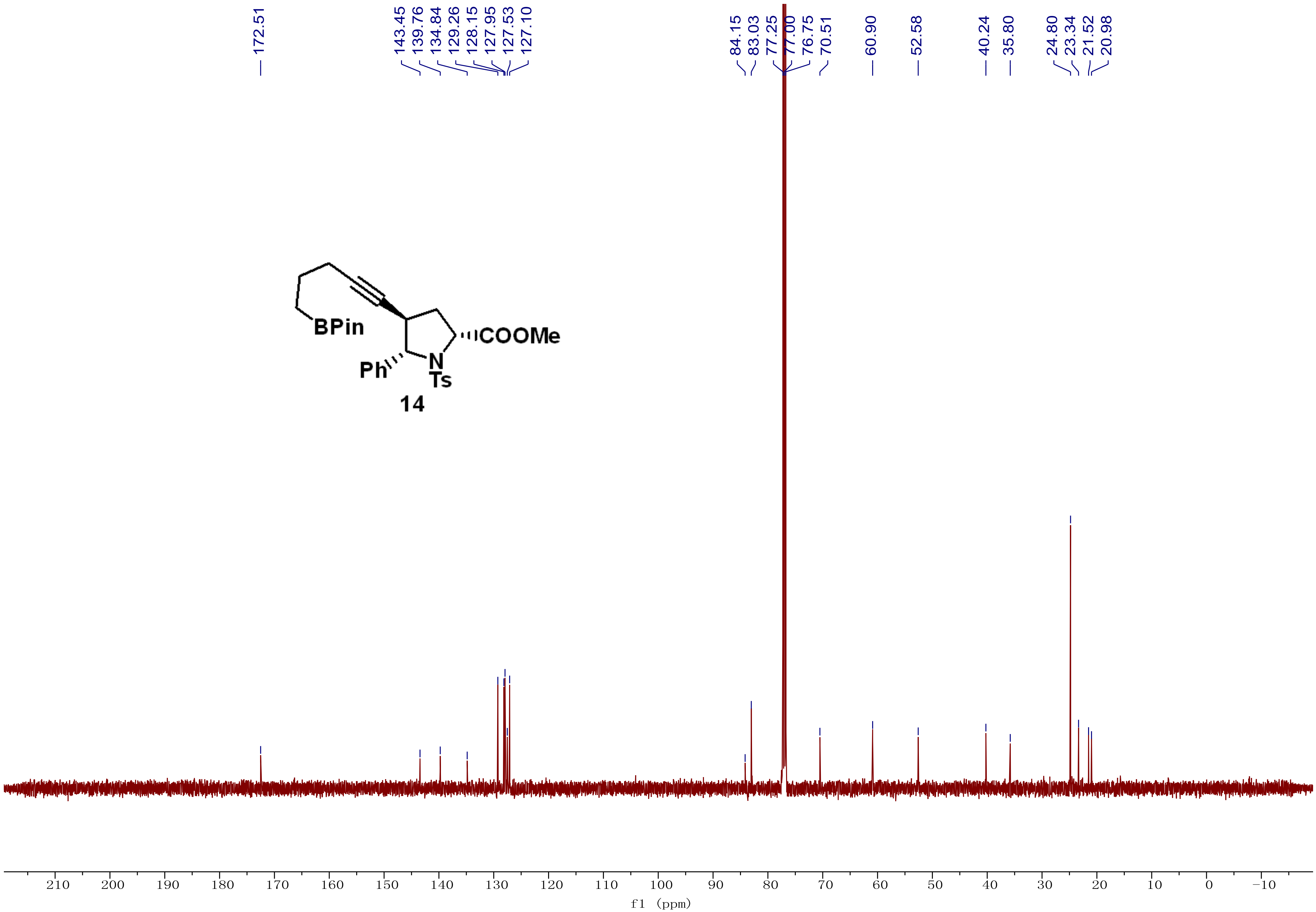
172.51
143.45
139.76
134.84
129.26
128.15
127.95
127.53
127.10
84.15
83.03
77.25
77.00
76.75
70.51
60.90
52.58
40.24
35.80
24.80
23.34
21.52
20.98
210 200 190 180 170 160 150 140 130 120 110 100 90 80 70 60 50 40 30 20 10 0 −10
f1 (ppm)
BPin
COOMe
Ph
N
Ts

14

4. Supplementary References

[1] Picher, M.-I.; Plietker, B. *Org. Lett.* **22**, 340−344 (2020).

[2] Zhang, Y.; Yun, B.; Gao, B.; Zhang, T.; Huang, H. *Org. Lett.* **21**, 535–539 (2019).

[3] Chang, X.-H.; Liu, Z.-L.; Luo, Y.-C.; Yang, C.; Liu, X.-W.; Da, B.-C.; Li, J.-J.; Ahmad, T.; Loh, T.-P.; Xu, Y.-H. *Chem. Commun.* **53**, 9344−9347 (2017).

[4] Wang, F.; Wang, D.; Zhou, Y.; Liang, L.; Lu, R.; Chen, P.; Lin, Z.; Liu, G. *Angew. Chem. Int. Ed.* **57**, 7140–7145 (2018).

[5] Adamson, N. J.; Jeddi, H.; Malcolmson, S. J. *J. Am. Chem. Soc.* **141**, 8574–8583 (2019).

[6] Sun, F.; Gao, J.; Fang, X. *Chem. Commun.* **56**, 6858−6861 (2020).

[7] Cheng, J.-K.; Loh, T.-P. *J. Am. Chem. Soc.* **137**, 42–45 (2015).

[8] Tang, X.; Woodward, S.; Krause, N. *Eur. J. Org. Chem*. **2009**, 2836–2844 (2009).

[9] Takayama, H.; Jia, Z.-J.; Kremer, L.; Bauer, J. O.; Strohmann, C.; Ziegler, S.; Antonchick, A. P.; Waldmann, H. *Angew. Chem. Int. Ed.* **52**, 12404–12408 (2013).

[10] Zhang, D.-J.; Xie, M.-S.; Qu, G.-R.; Gao, Y.-W.; Guo, H.-M. *Org. Lett.* **18**, 820–823 (2016).

[11] Wang, C.-J.; Liang, G.; Xue, Z.-Y.; Gao, F. *J. Am. Chem. Soc.* **130**, 17250–17251 (2008).

[12] Alemparte, C.; Blay, G.; Jørgensen, K. A. *Org. Lett*. **7**, 4569–4572 (2005).

[13] López-Pérez, A.; Adrio, J.; Carretero, J. C. *J. Am. Chem. Soc*. **130**, 10084–10085 (2008).

[14] Xiong, Y.; Du, Z.; Chen, H.; Yang, Z.; Tan, Q.; Zhang, C.; Zhu, L.; Lan, Y.; Zhang, M. *J. Am. Chem. Soc.* **141**, 961–971 (2019).

[15] Grigg, R.; Sarker, M. A. B. *Tetrahedron* **62**, 10332–10343 (2006).

[16] Kim, B.-S.; Gutierrez, O.; Kozlowski, M. C.; Walsh, P. J. *Adv. Synth. Catal.* **360**, 1426 – 1432 (2018).

[17] Turlington, M.; Du, Y.; Ostrum, S. G.; Santosh, V.; Wren, K.; Lin, T.; Sabat, M.; Pu, L. *J. Am. Chem. Soc.* **133**, 11780–11794 (2011).

[18] Pascual-Escudero, A.; de Cozar, A.; Cossio, F. P.; Adrio, J.; Carretero, J. C. *Angew. Chem. Int. Ed.* **55**, 15334-15338 (2016).

[19] Pan, Z.-Z.; Pan, D.; Li, J.-H.; Xue, X.-S.; Yin, L. *J. Am. Chem. Soc.* **145**, 1749–1758 (2023).

[20] Legault, C. Y. CYLView, 1.0b; Universitéde Sherbrooke: Québec, Montreal, Canada, 2009 (http://www.cylview.org).

[21] Lu, T.; Chen, F. *J. Comput. Chem.* **33**, 580-592 (2012).

[22] Lu, T.; Chen, Q. *J. Comput. Chem.* **43**, 539-555 (2022).

[23] Pracht, P.; Bohle, F.; Grimme, S. *Phys. Chem. Chem. Phys*. **22**, 7169 (2020).

[24] Grimme, S. *J. Chem. Theory Comput*. **15**, 2847 (2019).
