# Supplementary material for: Copper(I)-catalyzed asymmetric 1,3-dipolar cycloaddition of 1,3-enynes and azomethine ylides
Source: Nat Commun. 2023 Aug 4;14:4688. doi: 10.1038/s41467-023-40409-4 (PMC10403559; doi:10.1038/s41467-023-40409-4)
Supplement: Supplementary file 2 — Description of additional supplementary files [file 41467_2023_40409_MOESM2_ESM.pdf]

Description of Additional Supplementary Files

File Name: Supplementary Data 1
Description: Cartesian coordinates of all the optimized structures.
